# Efficacy and safety of human fibrinogen concentrate (BT524) in patients with major haemorrhage undergoing major orthopaedic or abdominal surgery (AdFIrst): a randomised, active-controlled, multicentre, partially blinded, phase 3 non-inferiority trial

**DOI:** 10.1016/j.eclinm.2025.103264

**Published:** 2025-06-07

**Authors:** Niels Rahe-Meyer, Ashok Roy, Hans-Heinrich Trouillier, Sonja Schimo, Judith Wessels-Kranz, Salomon Abraha, Alexander Staus, Ümniye Balaban, Thomas Häder, Jörg Schüttrumpf, Silke Aigner, Heike Böhm

**Affiliations:** a Franziskus Hospital Bielefeld, Kiskerstraße 26, 33615, Bielefeld, Germany; b Hampshire Hospitals NHS Foundation Trust, Aldermaston Road, RG24 9NA, Basingstoke, United Kingdom; c Orthopädie München-Schwabing, Kaiserstraße 26, 80801, München, Germany; d Biotest AG, Landsteiner Str. 5, 63303, Dreieich, Germany; e Ludwig-Maximilians-Universität München, Geschwister-Scholl-Platz 1, 80539, München, Germany; f Grifols S.A., Av. de la Genertalitat 152-158, Sant Cugat del Vallès, Barcelona, Spain

**Keywords:** Fibrinogen, Fibrinogen concentrate, Blood loss, Surgical, Haemorrhage, Haemostasis, Acquired fibrinogen deficiency, Cryoprecipitate, Fresh frozen plasma, Non-inferiority trial

## Abstract

**Background:**

Major haemorrhage is associated with considerable morbidity and mortality, but the optimal treatment remains disputed. This trial aimed to compare the efficacy and safety of human fibrinogen concentrate (FC) vs. either fresh frozen plasma (FFP) or cryoprecipitate (Cryo) as first-line treatment in patients with major bleeding during major orthopaedic or abdominal surgery, respectively.

**Methods:**

AdFIrst was a randomised, active-controlled, partially blinded, non-inferiority phase 3 trial conducted at 15 hospitals in Europe. Eligible patients (≥18 years), undergoing major spinal surgery or cytoreductive surgery for pseudomyxoma peritonei, with clinically relevant intraoperative blood loss were randomised by an interactive web response system (1:1) to receive intravenous FC (2–4 g) compared to FFP (15 mL/kg body weight) or Cryo (10 units); doses were repeated as needed. The primary endpoint was intraoperative blood loss from the time of decision to treat until the end of surgery with a non-inferiority margin of 150 mL, assessed in the per-protocol analysis set (PPS). Safety was assessed in all patients who received at least one dose of trial drug. The trial is complete (ClinicalTrials.gov: NCT03444324).

**Findings:**

Between February 12, 2018, and November 21, 2023, 222 patients (131 [59%] female; 91 [41%] male; 97% white) were enrolled and randomly assigned to receive either FC (n = 110) or FFP/Cryo (n = 112). The PPS included 201 patients (FC: n = 103; FFP/Cryo: n = 98). The primary endpoint was met; the least squares mean (LSM: adjusted mean estimated using ANOVA) intraoperative blood loss was 1381 mL (95% confidence interval [CI] 1187–1574) in the FC group and 1660 mL (95% CI 1461–1860) in the FFP/Cryo group. The LSM difference was −279 mL (95% CI −552 to −6; p < 0·0001), indicating a notable treatment difference beyond non-inferiority. In a post-hoc analysis, FC showed superiority to reduce intraoperative blood loss over FFP/Cryo in the PPS (p = 0·0087). Serious adverse events were reported in 28/110 patients (25%) in the FC and 41/112 patients (37%) in the FFP/Cryo group. Thromboembolic events occurred in 17 patients (FC: 4 (4%) and FFP/Cryo: 13 (12%). There were no treatment-related deaths.

**Interpretation:**

In patients undergoing major spinal or cytoreductive surgery for pseudomyxoma peritonei (PMP), FC was non-inferior to FFP/Cryo for the management of clinically relevant intraoperative bleeding. The efficacy and safety advantages observed in this trial support the emerging adoption of first-line use of FC in treatment guidelines.

**Funding:**

10.13039/501100025230Biotest AG, Germany.

**Research in context**

**Evidence before this study**

We searched PubMed on April 7, 2017, and updated it on August 4, 2020, using the terms “fibrinogen concentrate” [Title/Abstract] AND “plasma” [Title/Abstract] OR “cryoprecipitate” [Title/Abstract] OR “placebo” [Title/Abstract] OR “systematic review” [Title/Abstract] to identify clinical studies, meta-analyses, and systematic reviews comparing human fibrinogen concentrate (FC) with placebo, fresh frozen plasma (FFP), or cryoprecipitate in adults with surgical or trauma bleeding. Of the 55 results, we identified 8 systematic reviews, 1 meta-analysis, and 11 randomised controlled trials (RCTs) as being relevant, mostly in cardiac surgery, showing inconsistent results; limited or low-quality evidence was available for other types of surgery and trauma. To the best of our knowledge, no trials have directly compared FC with either FFP or cryoprecipitate, and robust evidence to guide optimal fibrinogen supplementation and product choice remains lacking.

**Added value of this study**

AdFIrst was the first multicentre, partially blinded, phase 3 trial to assess FC as a first-line treatment for major bleeding in major orthopaedic or abdominal surgery, and the largest to compare FC with either FFP or cryoprecipitate. The primary endpoint was met, showing FC was non-inferior to FFP/cryoprecipitate in reducing intraoperative blood loss, with statistically significant superiority observed in a post-hoc analysis of patients without major protocol deviations; a higher rate of successful correction of fibrinogen levels was also observed in the FC group. The safety profile of FC was favourable with a low incidence of thromboembolic events. Given the diverse range of surgical interventions and sources of bleeding included, the trial's results have broad applicability to all types of surgery with a high bleeding risk and trauma.

**Implications of all the available evidence**

The AdFirst trial showed that FC is non-inferior to FFP/cryoprecipitate for reducing clinically relevant bleeding in the intraoperative setting and expands the scope of surgical bleeding situations studied. The efficacy results, combined with the favourable safety profile and administration advantages of FC, support its use as a first-line treatment in patients with major haemorrhage.

## Introduction

Intraoperative bleeding occurs frequently in patients undergoing complex surgery and is associated with an increased risk of morbidity and mortality.^1^ In such patients, impaired haemostasis arising from the consumption and depletion of coagulation factors is common; moreover, this condition is exacerbated by haemodilution following allogeneic blood product (ABP) and volume administration.^2^

However, the optimal treatment strategy for the effective and safe management of coagulopathy in major bleeding remains elusive. Current guidelines are based on weak clinical evidence, as large randomised controlled trials (RCTs) are either missing or inconsistent in results. While massive transfusion protocols using fixed dose ratios of plasma, platelets and red blood cells are common, goal-directed bleeding management is steadily expanding in clinical practice. However, there is currently no consensus on the optimal approach.^2^, ^3^, ^4^, ^5^

Fibrinogen plays a central role in establishing and maintaining haemostasis in patients with major bleeding. Serving as the primary substrate in a clot and being essential to clot formation, it is also the first coagulation factor to drop below critical levels.^2^^,^^6^ Consequently, early supplementation of fibrinogen is considered important, with guidelines recommending repletion to levels of >1·5–2 g/L.^3^^,^^4^^,^^7^

Three different therapies are currently available for fibrinogen supplementation: fresh frozen plasma (FFP), cryoprecipitate (Cryo), and human fibrinogen concentrate (FC).^8^ FFP contains the lowest concentration of fibrinogen, but a near-physiological mixture of all coagulation and antithrombotic factors.^9^^,^^10^ Cryo, derived from FFP, contains higher concentrations of fibrinogen and other clotting factors, although the amounts vary.^8^^,^^11^ FC is a highly purified preparation with a defined fibrinogen content that can be used to rapidly and specifically increase fibrinogen levels without affecting other coagulation factors.^8^

The majority of previous studies on intraoperative bleeding management investigated cardiac surgery procedures with cardiopulmonary bypass, which feature surgery-specific conditions, including the administration of heparin/protamine and contact with foreign body surfaces and air.^12^ Early clinical data did not favour any particular fibrinogen-containing product^13^^,^^14^; however, the non-inferiority of FC to Cryo was later established in 735 cardiac surgery patients requiring fibrinogen supplementation post-bypass due to bleeding.^11^ A recent trial in patients undergoing major abdominal surgery for pseudomyxoma peritonei (PMP) confirmed this finding outside the cardiac setting,^15^ but there is little reliable evidence on the use of FC, FFP, and Cryo in other types of surgery. Most studies are retrospective with few patients.^13^^,^^16^^,^^17^ No single study has compared the efficacy of FC with either FFP or Cryo in major haemorrhage.

The lack of large, randomised, active-controlled studies has led to the emergence of a variety of clinical strategies worldwide. Although FFP is approved in all countries and has the broadest indications,^9^ Cryo predominates in the US and the UK, whereas FCs are favoured in Europe.^8^^,^^11^^,^^18^

Based on these considerations, we hypothesised that first-line treatment with FC would result in non-inferior haemostatic efficacy compared with FFP or Cryo in patients requiring fibrinogen supplementation, and that as a highly purified product, FC presents a safer option with a lower risk of thromboembolic complications.

The objectives of this phase 3 trial were to evaluate the efficacy and safety of a newly developed FC for the management of relevant intraoperative bleeding compared to standard of care (FFP or Cryo) in two types of surgery that are of general importance and encompass a wide range of bleeding sources, including bone, soft tissue, mucosal and tumour bleeding: The cytoreductive surgery for pseudomyxoma peritonei (PMP) and the spinal surgeries were chosen because they reliably induce significant blood loss. PMP tumors are mucinous tumors originating in the appendix that may spread to other abdominal organs. The extent of debulking surgery depends on the tumor's spread, potentially including right hemicolectomy, omentectomy, splenectomy, cholecystectomy, pelvic peritonectomy, and surgery for small bowel, diaphragm, or liver involvement. Spinal surgery (e.g., spondylodesis, release and scoliosis deformity correction, bony decompressions of the spinal canal, other complex spine reconstructions) requires extensive exposure of vertebrae, which are prone to bleeding. Types of bleeding include subcutaneous and muscle bleeding (diffuse and continuous), bone bleeding during resection, and the most problematic, epidural bleeding from the fragile venous plexus. Both types of surgery result in considerable peri-operative bleeding due to intravascular blood loss, hemodilution, and activation of the clotting cascade. This sequence of events can lead to a decrease in fibrinogen levels, necessitating replacement therapy. Since the underlying mechanism of bleeding and fibrinogen loss is consistent, these surgical models serve as valid representations for other procedures where significant blood loss is a risk.

The data obtained enhance our understanding of the management of bleeding across diverse surgical interventions, as well as trauma, which is difficult to study directly due to the wide variety of injuries and the nature of emergency settings in bleeding patients.

## Methods

### Trial design and patients

The AdFIrst trial (**Ad**justed **Fi**brinogen **r**eplacement **st**rategy implying to “Administer Fibrinogen First”) was a randomised, active-controlled, multicentre, partially blinded, phase 3, non-inferiority trial conducted at 15 hospitals (12 in the EU, two in Switzerland, and one in the UK). The aim was to demonstrate the efficacy and safety of FC as first-line treatment to reduce intraoperative blood loss in patients who experience uncontrolled major haemorrhage, whilst undergoing elective major spinal surgery or cytoreductive surgery for PMP. Adult patients (≥18 years of age) of either sex were recruited based on preoperative clinical assessments of the participating hospitals.

Major spinal surgery was chosen as an orthopaedic surgical model and PMP surgery as abdominal model. Patients undergoing spinal surgery were only eligible if they experienced intraoperative clinically relevant bleeding of approximately 1 L (Supplementary Material 1 pp 30–31). Patients undergoing PMP surgery were eligible if clinically relevant bleeding of ˃2 L was predicted intraoperatively (approximately 60 min after the start of surgery) (Supplementary Material 1 p 133). Trial drug administration was neither triggered solely by critical thresholds nor laboratory values. Instead, the treatment strategy was to prevent the need for additional hemostatic agents by implementing early fibrinogen supplementation and maintaining plasma fibrinogen levels. Main exclusion criteria were treatment with FC and/or any fibrinogen-containing product within 30 days before infusion of trial drug or presence/history of venous/arterial thrombosis or a thromboembolic event (TEE) within the preceding 6 months (for full criteria, see Supplementary Material 1 p 32).

The trial was conducted according to Good Clinical Practice guidelines, the Declaration of Helsinki, applicable regulatory requirements, and local regulations. Protocols and consent forms were approved by Independent Ethics Committees at each trial site. All patients provided written informed consent.

The trial was overseen by an independent data and safety monitoring board, which reviewed and assessed data in regular intervals throughout the trial.

### Randomisation and masking

Randomisation was stratified by site and surgery type. Patients were randomly assigned in a 1:1 ratio to receive either FC or FFP (for spinal surgery), or FC or Cryo (for PMP surgery) using balanced block randomisation with a block size of 4 within each site. Patients undergoing spinal surgery additionally underwent stratified randomisation by expected blood loss (˃1 L to ≤2 L and >2 L), which was recorded prior to surgery. Randomisation occurred intraoperatively, upon confirmation of trial eligibility, using an interactive web response system to retrieve the randomisation code.

The trial was partially blinded: the surgeon, surgical staff, and patients were blinded to treatment allocation; however, owing to different characteristics of the trial drugs, the anaesthesiologist responsible for administration could not be blinded. A sterile drape separated the surgical field from the anaesthetic field, covering the infusion stands (Supplementary Material 1 p 37).

### Procedures

The FC product used in this trial was BT524 (Biotest AG, Dreieich, Germany), a newly developed, lyophilized, heat-treated FC with a manufacturing process incorporating three virus inactivation steps: solvent/detergent treatment, ultraviolet C irradiation, and dry heat treatment. FC was supplied in single-use vials of 1 g and reconstituted with 50 mL of water for injection (20 mg/mL).

Spinal surgery: FC dose was calculated based on body weight (BW) and functional fibrinogen level from clot amplitude after 10 min (FIBTEM A10; measured intraoperatively by rotational thromboelastometry [ROTEM] using ROTEM delta and ROTEM sigma devices [Werfen GmbH, Munich, Germany]), with the aim to restore patient's individual baseline fibrinogen level. The formula used was: FC dose (g) = (baseline FIBTEM A10–actual FIBTEM A10) × BW/140.

The first FC dose was ≥2 g and the maximum total dose was 8 g. The recommended FFP dose was 15 mL/kg BW (FFP contains 2–5 mg/mL of fibrinogen),^1^ but ultimately determined by the extent of the patient's bleeding and their clinical condition as well as local standards. Repeated trial drug administration was permitted depending on the patient's clinical condition and FIBTEM A10 results (Supplementary Material 2 Fig. S1).

PMP surgery: the first dose of trial drug was a fixed dose of either 4 g FC or two pools of Cryo (10 units, equivalent to 4 g FC) (Supplementary Material 1 pp 138–139). Additional intraoperative doses (4 g FC or two pools of Cryo) were permitted based on the extent of the patient's bleeding, their clinical condition, and a FIBTEM A10 measurement of <12 mm (Supplementary Material 2 Fig. S2).

Trial drugs were administered by intravenous infusion with the aim of restoring individual baseline fibrinogen levels. The maximum infusion rate of FC was 250 mL/min. FFP was administered as rapidly as possible at the anaesthesiologist's discretion. Cryo was administered per site policies, usually 10–20 mL/kg/h.

Tranexamic acid was allowed to be administered prophylactically in all patients according to local standards. The administration of ABPs or autologous blood products, which effect coagulation and haemostatic agents was prohibited before and during the first administration of trial drug. No other restrictions were imposed; if required, further bleeding management was based on local hospital protocol.

Blood loss was continuously measured from the start of surgery up to 24 h after the end of surgery. The total blood loss was the sum of the blood quantified from the suction container and that absorbed by the surgical coths and swabs (for more detailed information see Supplementary Material 1 pp 55–56, 157–158). Functional fibrinogen levels determined by Clauss assay, FIBTEM A10 and maximum clot firmness (MCF) measured by ROTEM and coagulation activation tests were performed from the time of first trial drug administration through 24 h after surgery. FIBTEM was measured 15 and 90 min after the start of first trial drug administration, at the end of the surgery and one day after surgery. For the primary endpoint, the intraoperative blood loss was measured from the time of decision to treat until the end of surgery, blood from the blood suction unit, surgical cloths, and compresses was collected and measured using a standardised procedure. Rebleeding episodes were recorded starting at the end of surgery for up to eight days. The full schedule of assessments is provided in the Supplementary Material 1 pp 13−14, 115–116. The investigators and trial staff at all sites were trained on all protocol procedures prior to enrolment of any participant and on an ongoing basis throughout the trial, including the quantification of blood loss.

### Outcomes

The primary efficacy endpoint was intraoperative blood loss from the decision to treat the patient with trial drug (FC or FFP/Cryo) until the end of surgery. Secondary efficacy endpoints were: proportion of patients with successful correction of fibrinogen level, defined as achieving a restoration rate of ≥95% compared with baseline 15 min after the start of first trial drug administration (measured by FIBTEM A10); time to the first successful correction of fibrinogen level (≤15 min, >15 to ≤90 min, >90 min after trial drug administration, unsuccessful correction); proportion of patients with rebleeds after surgery until day 8; postoperative blood loss in the first 24 h; total amount of transfusion products or red blood cells (RBCs) infused after the start of the first trial drug administration until the end of surgery; length of hospital stay after surgery; and in-hospital mortality. Overall mortality was an exploratory efficacy endpoint.

Safety analysis was based on adverse events (AEs) and serious AEs (SAEs) that occurred during or after trial drug administration until the patient's last trial visit (treatment-emergent AEs/SAEs). AEs were continuously evaluated up to Day 36 following surgery. The final trial visit, which included the final safety examination, was scheduled for Day 36 but could be postponed up to Day 71 if required due to patient availability (e.g., if the patient was in a rehabilitation center). Frequency, severity, seriousness, and causality were assessed. AEs of special interest included TEEs, suspicion of transmission of infective agents, and relevant bleeding complications. Patients were observed closely for signs and symptoms of thrombosis, however no proactive monitoring using ultrasound or Doppler imaging were required. TEEs were defined as any arterial or venous TEEs per Medical Dictionary for Regulatory Activities (MedDRA) version 26·1 Standardised MedDRA Queries “embolic and thrombotic events, arterial” and “embolic and thrombotic events, venous”.

### Statistical analysis

An intra-operative blood loss of approximately 1 L initiated the decision to treat the patients with trial drug. Sample size was based on the assumption of an additional blood loss of approximately 500 mL in the FFP/Cryo group from the decision to treat until the end of surgery, a standard deviation of 375 mL, a non-inferiority margin of 150 mL, a one-sided alpha-level of 2·5%, and a t-test with at least 80% power. Assuming a 10% dropout rate, approximately 220 patients were randomised to have 200 evaluable patients (100 per group) in the per-protocol analysis set (PPS: all patients without major protocol deviations). Sample size was calculated using nQuery Advisor version 4·0 or higher.

The primary efficacy analysis tested non-inferiority in the PPS. A two-way analysis of variance (ANOVA) was performed with the intraoperative blood loss after decision to treat until the end of surgery as the dependent variable, and treatment group (FC vs. FFP/Cryo) and predictive blood loss (>1 L to = 2 L and >2 L) as the two factors, to evaluate the primary endpoint. Non-inferiority was demonstrated if the upper bound of the two-sided 95% confidence interval (CI) for the difference in the least squares mean (LSM) of blood loss between the FC and FFP/Cryo groups was less than the non-inferiority margin of 150 mL. In case of a significant violation to the assumptions for the ANOVA model for the primary endpoint, a non-parametric alternative method (van Elteren test) as sensitivity analysis was to be implemented. If non-inferiority was established, superiority was assessed using a hierarchical test procedure in the modified full analysis set (mFAS), comprising patients who received at least one dose of trial drug prior to the end of surgery and had at least one post-dose efficacy assessment. Secondary endpoints were analysed in the mFAS. Safety endpoints were analysed in all patients who received at least one dose of trial drug (safety set). Among the post-hoc analyses that were performed was the assessment of the superiority for the primary endpoint in the PPS, the calculation of p values for the length of hospital stay and risk ratio, 95% CI and p value for TEEs. For further details of analysis sets, subgroups, and post-hoc analyses, see Supplementary Material 2 p 2.

Endpoints were summarised descriptively and two-sided 95% CIs and statistical tests (Chi-square, Fisher's exact test, two-way analysis of variance (ANOVA), van Elteren test, independent two-sample t-test or Cochran-Mantel-Haenszel test) were added where appropriate and were interpreted on an exploratory basis. A p value of <0·05 was considered statistically significant.

Statistical analyses were performed using SAS version 9·4 (SAS Institute Inc., Cary, NC) or R for Statistical Computing (The R Foundation, Version 4·0, Vienna, Austria). CIs for median values were calculated using the ‘boot’ package in R software version 4·3·2. ClinicalTrials.gov registration: NCT03444324.

### Role of the funding source

The funder of the trial had a role in trial design; collection, analysis, and interpretation of data; and writing of the clinical trial report.

## Results

**Fig. 1:**
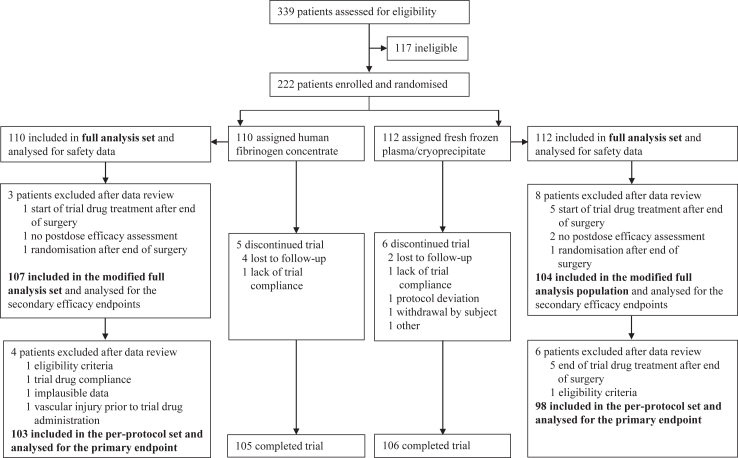
**Trial profile**.

Between February 12, 2018, and November 21, 2023, 339 patients who were to undergo spinal or PMP surgery were assessed for eligibility in the trial and gave informed consent: 222 were enrolled and randomised to receive either FC (n = 110) or FFP (spinal)/Cryo (PMP) (n = 112; FFP: 62, Cryo: 50); 117 were ineligible (Fig. 1). All 222 patients (100%, full analysis set [FAS]) received at least one dose of trial drug and were analysed for safety; 211 (95%) were in the mFAS, and 201 (91%; FC: 103; FFP/Cryo: 98) were in the PPS. Reasons for the exclusion of patients from each analysis set are provided in Fig. 1.

Demographics, baseline characteristics, and surgery types were balanced between treatment groups (Table 1). Overall, 98 (44%) patients underwent cytoreductive PMP surgery with hyperthermic intraperitoneal chemotherapy and 124 (56%) underwent spinal surgery, most commonly spondylodesis (40 [18%]) or release and scoliosis deformity correction (28 [13%]). Characteristics of surgery were consistent between treatment groups (Supplementary Material 2 Table S1). The median time between the decision to treat the patient with trial drug and the start of the first trial drug infusion, as well as the median duration of the first trial drug infusion, were both shorter in the FC group than in the FFP/Cryo group (33 min [interquartile range (IQR) 25–47] vs. 67 min [IQR 55–82], p < 0·0001, and 5 min (IQR 3–8) vs. 23 min (IQR 16–36), p < 0·0001, respectively). The total median trial drug volume administered was 150 mL (IQR 100–200) in the FC group and 915 mL (IQR 487–1076) in the FFP/Cryo group (p < 0·0001) (Table 2).

**Table 1:** Demographic and baseline characteristics of the trial population.

	FC (n = 110)	FFP/Cryo (n = 112)
Age, years	61 (54–70)	63 (51–73)
Sex	–	–
Female	67 (61)	64 (57)
Male	43 (39)	48 (43)
Race	–	–
Asian	1 (1)	2 (2)
White	109 (99)	107 (96)
Other	0 (0)	3 (3)
BMI, kg/m^2^	28 (24–31)	27 (23–31)
Expected blood loss	–	–
>1 L–≤2 L	44 (40)	48 (43)
>2 L	66 (60)	64 (57)
Laboratory values	–	–
Plasma fibrinogen, g/L^a^	3·7 (2·8–4·5)	3·7 (3·0–5·1)
FIBTEM A10, mm^b^	17·0 (13·0–21·0)	17·0 (15·0–22·0)
FIBTEM MCF, mm^b^	18·0 (13·0–22·0)	18·0 (15·0–25·0)
Platelet count, × 10^9^/L^c^	280 (226–329)	285 (241–349)
Haematocrit, fraction of 1^c^	0·40 (0·36–0·43)	0·40 (0·36–0·43)
Haemoglobin, g/L^c^	133 (120–143)	131 (123–145)
Creatinine, μmol/L^c^	70 (60–80)	69 (59–85)
Surgery type	–	–
Spinal surgery	62 (56)	62 (55)
Spinal surgery without tumour	53 (48)	57 (51)
Abdominal surgery (PMP)	48 (44)	50 (45)

Data are n (%) or median (IQR) for the safety set. BMI, body mass index; Cryo, cryoprecipitate; FC, human fibrinogen concentrate; FFP, fresh frozen plasma; FIBTEM A10, amplitude after 10 minutes as measured by ROTEM; FIBTEM MCF, maximum clot firmness as measured by ROTEM; n, number of patients; IQR, interquartile range; PMP, pseudomyxoma peritonei; ROTEM, rotational thromboelastometry.

a n = 106 in the FC group and n = 104 in the FFP/Cryo group.

b n = 106 in the FC group and n = 103 in the FFP/Cryo group.

c n = 109 in the FC group and n = 112 in the FFP/Cryo group.

**Table 2:** Comparison of exposure parameters for patients administered FC or FFP/Cryo.

	FC (n = 110)	FFP/Cryo (n = 112)	Difference (95% CI)	p value
Trial drug administrations during surgery	1^a^ (1–1)	1 (1–1)	0 (0–0)	0·43
Patients receiving 1 trial drug administration	95 (86)	93 (83)	3 (−6 to 13)	0·20
Patients receiving 2 trial drug administrations	15 (14)	15 (13)	0 (−9 to 9)	
Patients receiving 3 trial drug administrations	0	4 (4)	−4 (−7 to 0)	
Total trial drug administered, mL	150 (100–200)	915 (487–1076)	−765 (−830 to −600)	<0·0001
Time to start of first trial drug infusion, minutes	33 (25–47)^b^	67 (55–82)	−34 (−41 to −28)	<0·0001
Total trial drug administered for first infusion, mL	150 (100–200)	742 (473–1026)	−592 (−692 to −307)	<0·0001
Duration of first trial drug infusion, minutes	5 (3–8)	23 (16–36)	−18 (−21 to −15)	<0·0001
Patients with dose interruption	0 (0)	2 (2)	−2 (−4 to 1)	0·50

Data are n (%) or median (IQR). CI, confidence interval; Cryo, cryoprecipitate; FC, human fibrinogen concentrate; FFP, fresh frozen plasma; IQR, interquartile range; n, number of patients. Differences presented are percentage or median differences for FC—FFP/Cryo; a negative value indicates that less FC was administered than FFP/Cryo or that the infusion time for the FC group was shorter.

a Equivalent to 3 g FC.

b n = 108 in the FC group. Time to start of first trial drug infusion = Start time of first trial drug administration—Time to decision to treat patient with trial drug.

**Fig. 2:**
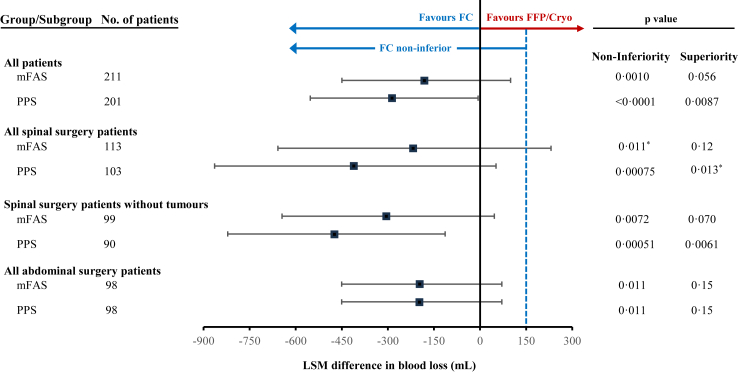
**Forest plot of the LSM difference (and associated CI) in intraoperative blood loss between patients treated with FC and FFP/Cryo**. LSM values are parametric, model-based estimates adjusted for blood loss and are presented with 95% CIs. Presented p values are derived from the nonparametric van Elteren test, which accounts for stratification by blood loss group (>1 L to ≤2 L and >2 L). PPS was used for analysis of the primary endpoint and mFAS was used for the sensitivity analysis. CI, confidence interval; Cryo, cryoprecipitate; FC, human fibrinogen concentrate; FFP, fresh frozen plasma; LSM, least squares mean; mFAS, modified full analysis set; PPS, per-protocol set. ∗Differences in the interpretation regarding the significance between confidence intervals and p values may be due to the distinct statistical methods employed by the LSM estimation and the van Elteren test.

Overall, the LSM of the primary efficacy endpoint of intraoperative blood loss from the decision to treat with trial drug until the end of surgery was 1381 mL (95% CI 1187–1574) in the FC group and 1660 mL (95% CI 1461–1860) in the FFP/Cryo group. The LSM difference was −279 mL (95% CI −552 to −6). As the primary endpoint was not normally distributed, the p values were calculated using the non-parametric van Elteren test. The upper bound of this CI (−6 mL) was below the predefined non-inferiority margin of 150 mL, establishing not only the non-inferiority of FC to FFP/Cryo in the PPS (p < 0·0001, Table 3; Fig. 2) but also indicating a notable treatment difference beyond non-inferiority.

**Table 3:** Summary of primary and secondary endpoints.

	FC	FFP/Cryo	Difference (95% CI)	p value
**Primary endpoint (intraoperative blood loss), mL**^a^	–	–	–	–
**Overall**	–	–	–	–
mFAS^b^	1433 (1237–1628)	1608 (1410–1806)	−175 (−449 to 99)	0·0010
PPS^c^	1381 (1187–1574)	1660 (1461–1860)	−279 (−552 to −6)	<0·0001
**Spinal surgery overall**	–	–	–	–
mFAS^d^	1723 (1402–2045)	1937 (1595–2279)	−213 (−658 to 231)	0·011
PPS^e^	1613 (1283–1943)	2019 (1665–2374)	−406 (−864 to 52)	0·00075
**Spinal surgery without tumours**	–	–	–	–
mFAS^f^	1458 (1187–1728)	1757 (1481–2032)	−299 (−645 to 47)	0·0072
PPS^g^	1394 (1119–1668)	1861 (1576–2146)	−467 (−821 to −113)	0·00051
**Abdominal surgery**	–	–	–	–
mFAS^h^	1517 (1331–1703)	1706 (1524–1889)	−190 (−450 to 71)	0·011
PPS^h^	1517 (1331–1703)	1706 (1524–1889)	−190 (−450 to 71)	0·011
**Secondary endpoints**^i^	–	–	–	–
Patients with first successful correction of fibrinogen level (by FIBTEM A10) intraoperatively^b^	–	–	–	–
≤15 min after trial drug start	59 (55)	19 (18)	37 (25–49)	<0·0001
>15 min or ≤90 min after trial drug start	17 (16)	11 (11)	5 (−4 to 14)	
>90 min after trial drug start	11 (10)	16 (15)	−5 (−14 to 4)	
Unsuccessful correction	20 (19)	58 (56)	−37 (−49 to −25)	
Patients with rebleeds after the end of surgery until day 8^b^	0 (0)	5 (5)	−5 (−9 to −1)	0·028
Post-operative blood loss in the first 24 h^b^	306 (234–378)	294 (221–367)	12 (−89 to 114)	0·81
Transfusion requirements^b^	–	–	–	–
Any type of transfusion product	79 (74)	72 (69)	5 (−10 to 19)	0·46
RBCs	65 (61)	61 (59)	2 (−11 to 15)	0·76
Length of hospital stay, days^j^	15 (10–19)	13 (10–20)	2 (0–3)	0·43
In-hospital mortality^b^	0 (0)	0 (0)	0 (0–0)	1·0

Data are least squares mean (95% CI), n (%), or median (IQR). CI, confidence interval; Cryo, cryoprecipitate; FC, human fibrinogen concentrate; FFP, fresh frozen plasma; FIBTEM A10, amplitude after 10 minutes as measured by ROTEM; IQR, interquartile range; mFAS, modified full analysis set; n, number of patients; PPS, per-protocol analysis set; RBC, red blood cell; ROTEM, rotational thromboelastometry.

a p values are derived from a non-inferiority test.

b n = 107 in the FC group and 104 in the FFP/Cryo group.

c n = 103 in the FC group and 98 in the FFP/Cryo group.

d n = 59 in the FC group and 54 in the FFP/Cryo group.

e n = 55 in the FC group and 48 in the FFP/Cryo group.

f n = 50 in the FC group and 49 in the FFP/Cryo group.

g n = 47 in the FC group and 43 in the FFP/Cryo group.

h n = 48 in the FC group and 50 in the FFP/Cryo group.

i p values are derived from a superiortiy test.

j n = 103 in the FC group and 100 in the FFP/Cryo group.

The non-inferiority of FC vs. FFP/Cryo for intraoperative blood loss was also indicated in the subgroups of patients who underwent spinal surgery (95% CI −864 to 52, p = 0·00075), spinal surgery without tumours (95% CI −821 to −113, p = 0·00051), and abdominal surgery (95% CI −450 to 71, p = 0·011) (Table 3). The results of the sensitivity analyses in the mFAS were consistent with those of the primary endpoint analysis.

The pre-specified superiority analysis of the primary endpoint revealed a tendency towards the superiority of FC compared with FFP/Cryo overall (p = 0·056). For the subgroups of spinal surgery, spinal surgery without tumours, and abdominal surgery, p values were 0·12, 0·070, and, 0·15, respectively. The post-hoc superiority analysis in the PPS revealed that FC was superior to FFP/Cryo in terms of intraoperative blood loss for all patients (p = 0·0087), for spinal surgery (p = 0·013), and spinal surgery in patients without tumours (p = 0·0061), but not for abdominal surgery (p = 0·15) (Fig. 2). Additional details on intraoperative blood loss are provided (Table 3, Fig. 2).

**Fig. 3:**
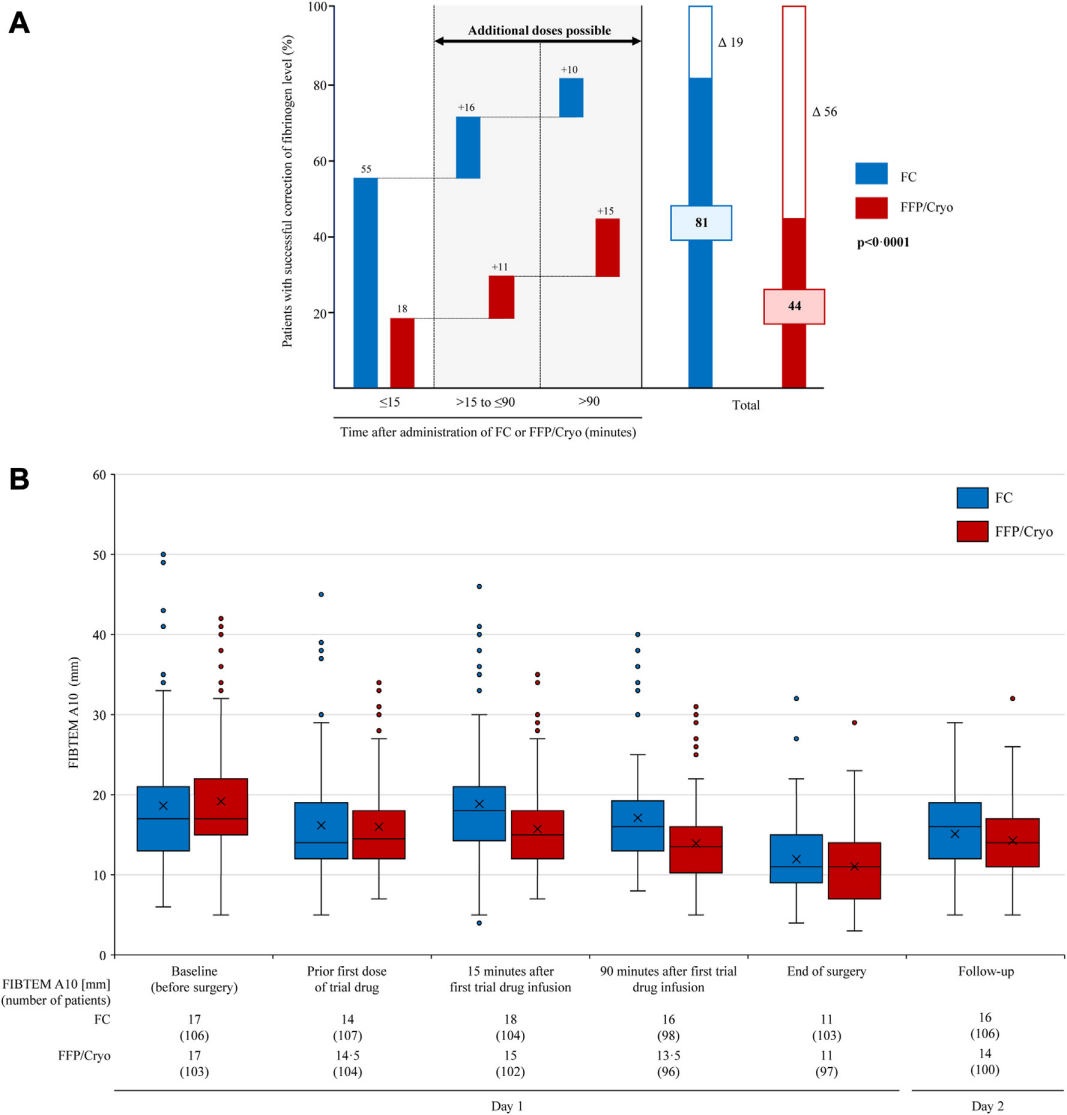
**Comparison of fibrinogen correction following administration of FC or FFP/Cryo**. (A) Percentage of patients with successful correction of functional fibrinogen level after the intraoperative administration of FC or FFP/Cryo. For patients with successful correction, the percentage with correction within the stated time period relative to the start of drug administration is also shown. The p value describes the difference between FC and FFP/Cryo over all defined time categories (B) Box-and-whisker plot depicting FIBTEM A10 as a measure of the functional fibrinogen level in patients as determined by ROTEM across different time points. Boxes represent the median, lower and upper quartiles (25th and 75th percentile, respectively), with whiskers indicating the minimum and maximum values. The whisker length is restricted to 1·5 times the IQR from the median; data points beyond this range are represented as individual dots. The mean value is indicated by a cross. End of surgery may have been before the 90-min time point. Cryo, cryoprecipitate; FC, human fibrinogen concentrate; FFP, fresh frozen precipitate; FIBTEM A10, functional fibrinogen level from clot amplitude after 10 minutes; ROTEM, rotational thromboelastometry.

Secondary efficacy endpoints are shown in Table 3. The time to first successful correction of functional fibrinogen levels by FIBTEM A10 compared to baseline was assessed in four categories relative to the start of the first trial drug administration. Patients in the FC group experienced faster correction (≤15 min: 55% vs. 18%; >15–≤90 min: 16% vs. 11%; >90 min: 10% vs. 15%; unsuccessful correction: 19% vs. 56%) and a significantly improved overall response rate (p < 0·0001, Fig. 3A). Additional details on fibrinogen correction are provided (Supplementary Material 2 Fig. S3 and Table S2).

The FIBTEM A10 values at pre-defined time points (from baseline to day 2) are depicted in Fig. 3B. The median functional fibrinogen level by FIBTEM A10 prior to first dose was similar in both groups: 14 mm in the FC group and 14·5 mm in the FFP/Cryo group; from the Clauss assay, fibrinogen concentrations were also similar (2·6 g/L and 2·8 g/L, respectively, Supplementary Material 2 Fig. S3). For a small number of patients with severely reduced functional fibrinogen levels, additional doses of trial drug were administered (Supplementary Material 2 p 3).

Rebleeds within 8 days after surgery occurred statistically more frequently in the FFP/Cryo group than in the FC group (5 [5%] vs. 0 [0%], p = 0·028) (Table 3). Owing to the nature of the surgical procedure, rebleeds only occurred in patients who underwent spinal surgery. The 24-h post-operative blood loss was similar between groups (LSM difference [95% CI]: 12 mL [−89, 114], p = 0·81).

The use of transfusion products after the start of first trial drug administration until the end of surgery was similar in both treatment groups (FC: 79 [74%] patients; FFP/Cryo: 72 [69%] patients, p = 0·46). Allogeneic RBCs were the most common transfusion products and usage was consistent across treatment groups, with median volumes of 329 mL (IQR 0–772) administered to 65 (61%) patients in the FC group and 328 mL (IQR 0–855) administered to 61 (59%) patients in the FFP/Cryo group (p = 0·76). No significant difference was found between treatment groups for the total amount of RBCs (allogeneic and autologous) consumed after the start of first trial drug administration until the end of surgery (FC median: 535 mL [IQR 0–850]; FFP/Cryo median: 534 mL [IQR 0–954], p = 0·83). Further details on transfusion product usage are provided (Supplementary Material 2 Table S3).

The median length of hospital stay was consistent across treatment groups (FC: 15 days [IQR 10–19]; FFP/Cryo: 13 days [IQR 10–20], p = 0·43 in a post-hoc analysis) and there was no difference in hospital stay length when analysed in weekly intervals (p = 0·065; Supplementary Material 2 Table S4). There were no in-hospital deaths in the trial.

The overall incidence and severity of AEs were comparable between treatment groups and related mainly to the surgical procedure (Table 4; Supplementary Material 2 Tables S5 and S6).

**Table 4:** Summary of adverse events and other outcomes (safety set).

	FC (n = 110)	FFP/Cryo (n = 112)
Any AE	96 (87)	100 (89)
Any adverse reaction	2 (2)	4 (4)
Any severe AE	17 (15)	18 (16)
Any severe adverse reaction	0	2 (2)
Any SAE	28 (25)	41 (37)
Any serious adverse reaction	1 (1)	4 (4)
Any AE leading to trial or treatment discontinuation	0	0
Any AE with outcome of death	0	1 (1)
Any adverse reaction with outcome of death	0	0
Any thromboembolic event	4 (4)	13 (12)
Pulmonary embolism	3 (3)	8 (7)
Deep vein thrombosis	1 (1)	5 (5)
Acute coronary syndrome	0	1 (1)
Any relevant bleeding complication	16 (15)	14 (13)
Any suspicion of transmission of infective agent	0	0

Data are n (%). Only treatment-emergent AEs are included. AE, adverse event; Cryo, cryoprecipitate; FC, human fibrinogen concentrate; FFP, fresh frozen plasma; n, number of patients; SAE, serious adverse event.

The most frequent AEs were different types of anaemia (25%), hallucination (25%), and hypotension (15%). Adverse reactions were rare, experienced by 2 (2%) patients in the FC group (1 [1%] patient experienced pulmonary embolism and thrombocytopenia; 1 [1%] patient experienced urticaria) and 4 (4%) patients in the FFP/Cryo group (all pulmonary embolism).

Most AEs were either mild or moderate. Severe AEs occurred in 35 (16%) patients, with a similar proportion of patients in each treatment group. None of them led to trial or treatment discontinuation. Overall, 103 SAEs were reported in 69 (31%) patients, with a lower incidence in the FC group than in the FFP/Cryo group (26% vs. 37%, respectively). No deaths were reported in the FC group, whereas one death (1%) occurred in the FFP/Cryo group. This fatal event (severe type 2 respiratory failure) occurred 28 days after Cryo administration and was deemed unrelated to the trial drug.

The incidence of TEEs was lower in the FC group than in the FFP/Cryo group: pulmonary embolism was reported in 3 (3%) and 8 (7%) patients and deep vein thrombosis was reported in 1 (1%) and 5 (5%) patients, respectively (Supplementary Material 2 Fig. S4 and Table S5). Post-hoc analyses confirmed that TEEs occurred significantly less often in the FC group. The risk of TEEs was lower in patients treated with FC compared with FFP/Cryo (risk ratio [RR] 0·31, 95% CI 0·11–0·93, p = 0·041).

There were no clinically meaningful differences in mean or median clinical laboratory values or vital signs across treatment groups.

## Discussion

The AdFIrst trial met the pre-specified endpoint, demonstrating the non-inferiority of FC to FFP/Cryo. To our knowledge, this is the first randomised trial to show significant superiority of FC in reducing intraoperative blood loss compared to FFP/Cryo (post-hoc analysis in the PPS). FC also demonstrated an advantageous safety profile and favourable administration characteristics compared to standard of care, which may position FC as the treatment of choice.

The broad clinical applicability of the data generated in this trial arises predominantly from the surgery types studied. Previous research was predominantly focused on cardiac surgery. In studies comparing FC to placebo^19^, ^20^, ^21^ or FFP,^10^ less ABP use^10^^,^^19^, ^20^, ^21^ and blood loss^10^^,^^21^ were reported with FC. Other studies could not confirm these outcomes, because either the advantage of FC was not statistically significant for the primary endpoint (ABP use^22^ or intraoperative blood loss^23^) or ABP usage was higher with FC.^24^ The most recent phase 3 trial, FIBRES, demonstrated the non-inferiority of FC to Cryo for bleeding management in cardiac surgery. Based on this phase 3 trial the first FC was approved in the US for the supplementation of fibrinogen in acquired fibrinogen deficiency.^11^

A limited number of studies, most of them retrospective and including only a small number of patients, have evaluated the use of FFP, Cryo and FC in other surgical settings, such as orthopaedic surgery.^13^^,^^15^, ^16^, ^17^ For FFP, the clinical evidence is inconsistent and weak across all assessed indications and outcomes.^9^^,^^25^ There is one published trial of major abdominal surgery, which indicated the non-inferiority of FC to Cryo in achieving successful overall haemostatic efficacy.^15^ Currently, the totality of the clinical data does not conclusively support any specific fibrinogen replacement therapy.^1^ This trial provided clinically meaningful data on the use of FC in orthopaedic and abdominal surgery, helping to close the knowledge gap outside cardiac surgery.

Our results showed that FC was non-inferior in reducing intraoperative blood loss compared with FFP/Cryo (LSM difference [95% CI]: −279 mL [−552 to −6]), with a notable treatment difference between the upper bound (−6 mL) and the non-inferiority margin (150 mL). Further, post-hoc analysis in the PPS indicated superior haemostatic efficacy of FC to FFP/Cryo (p = 0·0087), which to our knowledge has not been shown in other multicentre pivotal trials yet.

A primary goal in clinical trials of haemostatic agents is to control bleeding, and the amount of blood loss from coagulopathic bleeding is considered as a direct indicator of the haemostatic capability of the treatment. Previous randomised controlled trials have used highly variable or institution-dependent endpoints, such as number of ABPs administered,^11^^,^^21^^,^^24^ or subjective measures such as investigator's assessment,^15^ resulting in a lack of standardised trial outcomes. Although no measure is without limitation, a major strength of this trial was the use of an objective measurement to determine intraoperative blood loss.

Our trial evaluated FC in two surgery types. Major spinal surgery involves different types of bleeding associated with continuous oozing bleeding that is rarely high intensity but generally widespread and prolonged: subcutaneous and muscle bleeding, bone bleeding, and epidural bleeding.^26^ Inclusion of debulking PMP surgery provides data on constant oozing soft tissue bleeding and bleeding from mucosal wounds, glandular tissue, and abdominal tumours.^27^ Our results on intraoperative blood loss are similar to the data of the previous FORMA-05 phase 2 trial^15^ comparing FC with Cryo in patients who underwent PMP surgery. Both trials confirmed the efficacy and safety of FC in the bleeding management of patients, however, the FORMA-05 trial used a different primary endpoint. i.e. a four-point hemostatic efficacy scale comparing predicted and observed intra-operative blood loss according to investigator assessment.^15^ For spinal surgery, insufficient data exist for comparison. A recent trial by Chen et al. investigated a different population (children) and a different surgical setting, restricted to scoliosis.^28^ Based on the conserved function of fibrinogen in the clotting cascade, the diverse vascular sources of bleeding, and the multiple tissue types represented in this trial, our results represent broader applicability to all types of surgery with a high risk of bleeding and trauma.

The maintenance of haemostasis is acknowledged as a critical factor in the management of patients with major bleeding. Impairment of haemostasis is often associated with a rapid fall in plasma fibrinogen levels and consequently diminished clot quality. Hence, if fibrinogen levels remain low, it seems logical that blood loss will continue. The strategy of minimising further blood loss through the early supplementation and maintenance of plasma fibrinogen levels has been proposed as crucial for controlling bleeding.^7^

The results for our secondary endpoints support those of the primary endpoint, suggesting the efficacy of FC for maintaining fibrinogen levels. Prior to the first treatment step, laboratory measurements show a reduction in functional fibrinogen levels. Then, following trial drug administration, an adequate increase was achieved primarily in the fibrinogen group (≤15 min: 55% vs. 18%), confirming results in previous FC studies.^11^^,^^15^^,^^24^

To enable early haemostasis, this clinical trial focused on the timely supplementation of fibrinogen; the treatment decision was based on measurement and assessment of the bleeding situation and not based on critical levels of fibrinogen as currently recommended by guidelines.^1^ Future guidelines may also reflect this approach, supporting the first-line treatment with FC to elevate and maintain fibrinogen in clinical practice.

In addition, the secondary endpoints relating to the number of transfusion products used were consistent between groups. As we did not stipulate a standardised transfusion protocol across the sites, treatment decisions were subjective; hence, these secondary endpoints provided limited information.

The safety profile of FFP/Cryo is impacted by several concerns: risk of infectious disease transmission, transfusion-related acute lung injury, volume overload and associated complications, hypersensitivity and allergic reactions, and a risk of TEEs.^8^^,^^18^^,^^20^ The highly purified, triple virus-inactivated FC used in this trial provided relevant safety advantages over the alternatives: Our results showed the generally consistent safety profiles of FC and FFP/Cryo, but importantly there was a statistically significantly lower incidence of TEEs in the FC group. Although FFP and Cryo contained other coagulation-activating substances,^8^^,^^9^ their presence does not appear to increase therapeutic efficacy. Instead, coagulation activity may be unintentionally increased in different, non-bleeding areas of the vasculature, thereby increasing the risk of AEs such as TEEs. This aligns with findings from systematic and comprehensive reviews, which show a generally low incidence of TEEs in patients treated with FC and a lower risk of developing TEEs compared with controls.^29^^,^^30^ Additionally, a recent analysis of real-world data suggested no association between FC administration in a perioperative setting and the risk of TEEs, supporting FC's favourable safety profile with respect to thromboembolic risks.^31^

In general, no restrictions were applied in AE reporting and all untoward medical occurrences, regardless of their causal relationship to the trial drugs, were collected. As expected in these surgical settings, the overall incidence of AEs was high and comparable between treatment groups, reflecting the nature of the major surgical procedures and the underlying clinical condition of the trial population.

Our results confirmed the known advantages of FC in handling and targeted dosing. The administration of FC to a bleeding patient is logistically simpler: a standardised amount of purified fibrinogen can be instantly delivered in a small volume (thawing and blood type matching not required). This was reflected in the trial data: FC was delivered and administered more quickly after the decision to treat (median: 33 min [IQR 25–47] vs. 67 min [IQR 55–82], p < 0·0001) and the duration of administration was significantly shorter (median: 5 min [IQR 3–8] vs. 23 min [16–36], p < 0·0001).

Our trial had some limitations. Not all clinical conditions involving bleeding were studied. Cytoreductive PMP surgery is performed infrequently at only a few specialised centres: as the aim was to compare FC to Cryo in this surgery, recruitment in European countries (where the standard of care is FFP) was not possible. As the trial compared FC with two currently used therapies for fibrinogen replacement under typical clinical practice conditions, we did not include a placebo group to determine the efficacy of fibrinogen replacement per se. The multicentre design could not control for factors such as transfusion regimen, volume management, and postoperative coagulation management; hence, these were not standardised. However, stricter protocol regulations or more complex treatment algorithms to reduce variability might have increased the number of protocol violations^32^ or made it more difficult to demonstrate unambiguous discrimination for treatment-related endpoints.^24^

In conclusion, the AdFIrst trial was the largest trial conducted in patients undergoing major orthopaedic or abdominal surgery and the first pivotal trial to compare FC directly with both FFP and Cryo. This trial showed that FC was non-inferior to both FFP and Cryo and reduced intraoperative blood loss in patients undergoing major spinal or abdominal surgery, most likely as a direct result of the supplementation of FC as a haemostatic agent. This trial has made a contribution to the evidence on the use of FC in a broad range of bleeding situations and confirmed its favourable safety profile.

Given the demonstrated efficacy advantages of FC over FFP/Cryo, as well as safety and administration benefits, the results of this trial support the use of FC as a first-line treatment in patients with major haemorrhage.

## Contributors

NRM was Coordinating Investigator, AR and HHT were Principal Investigators each in one site of the trial. JS supervised the overall project. NRM, AR, HHT, HB and TH substantially contributed to the conception and design of the trial. HB, SiAi, TH and JWK were responsible for the management, oversight and reporting of the trial. NRM, AR, SiAi and HB led the interpretation of the trial data. SaAb led the safety-related project management and data interpretation. NRM, AR, JWK, HB, SiAi, SaAb, AS and ÜB had access to and verified the underlying data. AS and ÜB were involved in the statistical concept, analysis, and interpretation of the trial data. SS contributed to the data interpretation and wrote the initial draft of the manuscript. All authors reviewed and commented its previous versions critically. All authors gave final approval of the version to be published and agreed to be accountable for all aspects of the work in ensuring that questions related to the accuracy or integrity of any part of the work are appropriately investigated and resolved.

## Data sharing statement

Data collected for the trial, including individual participant data and a data dictionary defining each field in the dataset, will not be made available to others, as the patients’ informed consent form does not account for this. The trial protocol, statistical analysis plan, and master informed consent form are included in the Supplementary Material 1 accompanying this paper.

## Declaration of interests

All authors have completed the ICMJE uniform disclosure form and declare: NRM was coordinating investigator of AdFirst trial, received fees for study participation, consulting fees from Biotest AG, Dreieich and CSL-Behring, Marburg, Germany, and personal travel costs and time allowance for the participation in the final study meeting and analysis and interpretation of the data. AR was principal investigator in the Biotest Phase 3 trial reported, is principal investigator for Phase 3 study of CSL Behring and received honoraria from Takeda and Octapharma. HHT was principal investigator Biotest trial reported and has a leadershiprole in the Deutsche Gesellschaft für Wirbelsäulentherapie. SiAi, SaAb, HB, ÜB, TH, SS, AS, JWK are employees of Biotest AG, the sponsor of the trial. Until Aug 2024 JS was employee of Biotest AG, the sponsor of the trial and has been employee of Grifols S.A., since September 2023.
