# Supplementary material for: Efficacy and safety of human fibrinogen concentrate (BT524) in patients with major haemorrhage undergoing major orthopaedic or abdominal surgery (AdFIrst): a randomised, active-controlled, multicentre, partially blinded, phase 3 non-inferiority trial
Source: eClinicalMedicine. 2025 Jun 7;85:103264. doi: 10.1016/j.eclinm.2025.103264 (PMC12308311; doi:10.1016/j.eclinm.2025.103264)
Supplement: Supplementary Material 1 [file mmc1.pdf]

Supplementary Material 1

Efficacy and safety of human fibrinogen concentrate (BT524) in patients with major haemorrhage undergoing major orthopaedic or abdominal surgery (AdFIrst): a randomised, active-controlled, multicentre, partially blinded, phase 3 non-inferiority trial.

Prof Niels Rahe-Meyer, MD, Ashok Roy, MD, PD Dr Hans-Heinrich Trouillier, MD, Dr Sonja Schimo, Dr Judith Wessels-Kranz, Salomon Abraha, Dr Alexander Staus, Dr Ümniye Balaban, Dr Thomas Häder, Dr Jörg Schüttrumpf, MD, Dr Silke Aigner, Heike Böhm

CONTENTS

Supplementary Documents	Page
Document S1: Clinical study protocol for study sites in the EU and Switzerland—Study 995 CSP version 4.0 (995_CSP_V4.0_EU_final_04-Dec-2019_redacted)	2
Document S2: Clinical study protocol for study sites in the UK—Study 995 CSP version 4.3 (995_CSP_V4.3_UK_06-Sep-2021_redacted)	102
Document S3: Study 995 Statistical analysis plan—(995_Statistical Analysis Plan_V6_19-Jun-2023_redacted)	205
Document S4: Subject information sheet and Master informed consent form—(995 - Informed Consent Form _Master V5.0 Final-clean_ 12 Dec 2019_redacted)	246

Document S1: Clinical study protocol for study sites in the EU and Switzerland—Study 995 CSP version 4.0 (995_CSP_V4.0_EU_final_04-Dec-2019_redacted)

Clinical Study Protocol

Title: A randomized, active-controlled, multicenter, phase III study investigating efficacy and safety of intra-operative use of BT524 (human fibrinogen concentrate) in subjects undergoing major spinal or abdominal surgery (AdFIrst)

Short Title: AdFIrst - Adjusted **Fi**brinogen **r**eplacement **st**rategy

**Clinical Phase:**	III
**Version incl. date:**	Final 4.0 of 04-DEC-2019
**EudraCT Number:**	2017-001163-20
**Study No.:**	995

Sponsor PPD

Coordinating Investigator PPD

Confidentiality Statement

This protocol is property of Biotest AG, D-63303 Dreieich, and may not be circulated, reproduced or published - either in whole or in part - without the company's written permission.

Overview of Amendments integrated in the protocol text of Version 4.0 of 04-DEC-2019

Amendment No / CSP Version	Date	Sections concerned	Rationale
1 / CSP V2.0	26-APR-2018	Signature Page,<br>Synopsis,<br>2, 3, 4, 6, 7, 8, 9, 10	Introduction of a new Biostatistician.<br>Update with clarification of wording, corrections and formatting.<br>Revision of the protocol to eliminate inconsistencies between different protocol sections.
1 / CSP V2.0	26-APR-2018	Flowchart,<br>3, 7, 9	Clarification regarding the time-period between screening and baseline and the need of repeated assessments and diagnostic tests at these visits.
1 / CSP V2.0	26-APR-2018	Synopsis, 4	Incomplete exclusion criterion 4 was complemented.
1 / CSP V2.0	26-APR-2018	3	Clarification of treatment algorithm.
1 / CSP V2.0	26-APR-2018	6	Clarification of treatment algorithm resulted in a revised definition for prohibited medication (section 6.10).<br>Restructuring of section 6.11 'Warnings and Precautions'.
1 / CSP V2.0	26-APR-2018	9, 11	Update and clarification regarding the handling of laboratory samples and the assessment of results.
2 / CSP V3.0	07-JUN-2019	Signature page,<br>Introduction, 4	Introduction of a new BiostatisticianPPD<br>Update with clarification of wording, corrections and formatting.
2 / CSP V3.0	07-JUN-2019	Synopsis, Flowchart,<br>3, 4, 7, 9, 10	Intra-operative inclusion criterion was adapted. Based on patients' bodyweight and clinical condition, the clinical need of FFP transfusion during surgery can already occur after a clinically relevant bleeding of approximately 1 liter.<br>Instead of measurement and calculation of blood loss prior to the 'decision to treat', in case of a clinically relevant bleeding an estimation of blood loss will take place.
2 / CSP V3.0	07-JUN-2019	4.2	Inclusion criterion 4 aims to ensure that only patients without hereditary bleeding disorders are to be included in this study. Therefore, a footnote for clarification of wording was included.
2 / CSP V3.0	07-JUN-2019	Synopsis, Flowchart,<br>3, 6, 7, 9	The dosage was adapted in order to avoid under-dosing of subjects. The first BT524 treatment was changed to a minimum dose of 2 g. The option for repeated dosing with IMP was included. The wording of the

Amendment No / CSP Version	Date	Sections concerned	Rationale
			secondary endpoints was adapted accordingly.<br>The dose justification was updated.
2 / CSP V3.0	07-JUN-2019	Flowchart, 7	The time points '90 minutes after treatment start' and 'end of the surgery' can be close together. In this case, blood samples do not have to be taken at both times. Update with clarification of wording. The option for repeated dosing with IMP was included.
2 / CSP V3.0	07-JUN-2019	7	Clarification of wording regarding the re-screening of subjects.
2 / CSP V3.0	07-JUN-2019	9.3, Appendix 2	Update and clarification regarding the definition of Adverse Events of Special Interest (AESI) and the respective reporting procedures.
3 / CSP V4.0	04-Dec-2019	Cover Page, Signature page, 1, 9	Introduction of a new Biostatistician PPD . General update with clarification of wording, corrections and formatting.
3 / CSP V4.0	04-Dec-2019	Synopsis 2, 3, 5, 7.2, 8.1	Study synopsis, study objectives and study design have been modified to extend the target population of the study to allow inclusion of subjects undergoing pseudomyxoma peritonei surgery (only applicable in the United Kingdom) and to introduce the active comparator cryoprecipitate in this treatment group.
3 / CSP V4.0	04-Dec-2019	4.1 and 4.2, 6.6	Update in order to take particular account of subjects with pseudomyxoma peritonei.
3 / CSP V4.0	04-Dec-2019	Synopsis, 3, 4.2, 6.1, 6.6, 7	Clarification of wording of the intra-operative inclusion criterion.
3 / CSP V4.0	04-Dec-2019	Synopsis, 10	Update with clarification of wording and revision to include interim analyses.<br>Following the Data Monitoring, the assumed standard deviation has been adjusted and the power has been reduced.
3 / CSP V4.0	04-Dec-2019	19	Update of scientific literature mainly regarding the inclusion of subjects with pseudomyxoma peritonei.

I. SIGNATURE PAGE

PPD

I.I Signature Page for Investigators

Declaration of the Principal Investigator

I have read and understood this Clinical Study Protocol and agree to the following:

- To adhere to the ethical and scientific principles of Good Clinical Practice, and the principles of the Declaration of Helsinki, the local laws and regulations, and the applicable regulatory requirements.
- To conduct the clinical study as set out in the protocol.
  This includes:
  - To wait until I have received approval from the appropriate Independent Ethics Committee / Institutional Review Board (IEC/IRB) before enrolling any subject in this study.
  - To obtain informed consent for all subjects prior to any study-related measure performed.
  - To permit study-related monitoring, audits, IEC/IRB review, and regulatory authority inspections.
  - To provide direct access to all study-related records, source documents, and subject files for the monitor, auditor, IEC/IRB, or regulatory authority upon request.
  - To use the IMP and all study materials only within the framework of this Clinical Study Protocol.
  - To understand that changes to the Clinical Study Protocol must be made in the form of an amendment that has the prior written approval of Biotest and, as applicable, of the appropriate IEC/IRB and regulatory authority.
  - To comply with the reporting obligations for all Adverse Events

I understand that all documentation that has not been previously published will be kept in the strictest confidence. This documentation includes the Clinical Study Protocol, Investigator's Brochure, Case Report Forms, and other scientific data.

Principal Investigator
Name

____
Date, signature

Investigator stamp:

Please insert stamp of investigational site

II. STUDY SYNOPSIS

**Title**	A randomized, active-controlled, multicenter, phase III study investigating efficacy and safety of intra-operative use of BT524 (human fibrinogen concentrate) in subjects undergoing major spinal or abdominal surgery (AdFIrst)
**Clinical Phase**	III
**Coordinating Investigator**	PPD
**Study Objectives**	The main purpose of this phase III study is to demonstrate the efficacy of BT524 as a complementary therapy to management of uncontrolled severe hemorrhage in subjects undergoing elective major spinal or abdominal surgery.<br>The **primary objective** of this study is to demonstrate that BT524 is non-inferior that means not worse than fresh frozen plasma (FFP)/cryoprecipitate with a non-inferiority margin of 150 mL in reducing intra-operative blood loss by intravenous (IV) administration in subjects with acquired hypofibrinogenaemia undergoing elective major spinal or abdominal surgery.<br>If therapeutic equivalence (non-inferiority) has been demonstrated, therapeutic superiority of BT524 compared with FFP/cryoprecipitate will also be assessed.<br>**Secondary objectives** are to demonstrate the efficacy of BT524 by assessing the correction of the fibrinogen level intra-operatively, the transfusion requirements, post-operative blood loss in the first 24 hours, the number of subjects with rebleeds, the hospital length of stay and the in-hospital mortality. Secondary objectives also comprise the safety of BT524 by documenting the number of adverse events (AE) including changes in laboratory parameters, the virus status, and the frequency and severity of thrombosis and of thromboembolic events (TEEs).
**Study Design**	Prospective, randomized, active-controlled, multicenter, non-inferiority study
**Study Population**	Adult subjects (≥ 18 years) of both gender undergoing elective spinal or abdominal surgery with expected major blood loss
**Inclusion Criteria**	<u>**At screening:**</u><br>1. Written informed consent<br>2. Subjects scheduled for elective major spinal or cytoreductive pseudomyxoma peritonei (PMP)[1] surgery with expected major blood loss<br>3. Male or female, aged ≥ 18 years<br>4. No increased bleeding risk as assessed by standard coagulation tests and medical history

[1] Only applicable for subjects in the United Kingdom (UK)

	5. Intra-operative trigger for treatment:<br>a. **Subjects undergoing spinal surgery:** Intra-operative clinically relevant bleeding of approximately 1 L, requiring hemostatic treatment during surgery.<br>b. **Subjects undergoing cytoreductive PMP surgery[2]:** Intra-operative prediction of clinically relevant bleeding of > 2 L, requiring hemostatic treatment during surgery.
**Exclusion Criteria**	1. Pregnancy or unreliable contraceptive measures or breast feeding (women only)<br>2. Hypersensitivity to proteins of human origin or known hypersensitivity reactions to components of the Investigational Medicinal Products (IMP)<br>3. Participation in another clinical study within 30 days before entering the study or during the study and/or previous participation in this study<br>4. Treatment with any fibrinogen concentrate and/or fibrinogen-containing product within 30 days prior to infusion of IMP<br>5. Employee or direct relative of an employee of the Contract Research Organization (CRO), the study site, or Biotest<br>6. Inability or lacking motivation to participate in the study<br>7. Medical condition, laboratory finding (e.g. clinically relevant biochemical or hematological findings outside the normal range), or physical exam finding that in the opinion of the investigator precludes participation<br>8. Presence or history of venous/arterial thrombosis or TEE in the preceding 6 months
**Number of Subjects**	100 evaluable subjects per treatment arm
**Countries / Number of Study Sites**	Multicenter, multinational, Europe / 15-20 sites<br>**EU and Switzerland:** Spine surgery, FFP as comparator<br>**United Kingdom:** PMP surgery, cryoprecipitate as comparator
**Investigational Medicinal Products (IMP)**	**BT524** (human fibrinogen concentrate) and **FFP** (fresh frozen plasma) and **cryoprecipitate**<br>**BT524** is a heat-treated, lyophilized fibrinogen concentrate manufactured from human plasma.<br>BT524 is presented as a single-use vial containing 1 g of lyophilized fibrinogen. The lyophilisate is to be reconstituted with 50 mL of water for injections, resulting in a final concentration of 20 mg/mL for IV infusion.<br>**FFP** refers to the liquid portion of human blood that has been frozen and preserved after a blood donation and will be used for blood transfusion.

[2] Only applicable for subjects in the UK

	**Cryoprecipitate** is made from FFP which is frozen and repeatedly thawed in a laboratory to produce a source of concentrated clotting factors including fibrinogen, factor VIII, factor XIII, von Willebrand factor (vWF) and fibronectin and platelet microparticles.
**Dosage and Mode of Administration**	**Subjects undergoing spinal surgery:**<br>**BT524**, administered intravenously:<br>Dosage according to FIBTEM A10 values to restore baseline fibrinogen level:<br>**BT524 dose (g) = [baseline FIBTEM A10 - actual FIBTEM A10] x actual body weight (BW) /140**<br>**First dose at least 2 g**, subsequent intra-operative infusions as required.<br>**FFP**, administered intravenously:<br>Dosage according to local standards; the recommended dose of FFP is 15 mL per kg body weight (BW). Subsequent intra-operative infusions as required.
**Duration of Treatment**	**Single or repeated intra-operative administration of IMP.**<br>*Follow-up per subject:*<br>Each subject will be followed for at least 5 weeks, with clinical and laboratory data collected on visits scheduled on Days 2, 3, 5 and 8, and the closing visit, scheduled on Day 36* after the day of surgery (*+35, up to Day 71 if required).
**Criteria for Evaluation**<br>***- Efficacy***	***Primary Endpoint:***<br>• Intra-operative blood loss after decision to treat the subject with IMP until the end of surgery as measured by amount of blood from the blood suction unit and amount of blood from surgical cloths and compresses.<br><br>***Secondary Endpoints:***<br>• Proportion (%) of subjects with successful correction of fibrinogen level 15 minutes after start of first IMP administration<br>• Time to first successful correction of fibrinogen level<br>• Total amount of transfusion products (allogenic blood products) or autologous blood transfusion infused after start of first IMP administration until end of surgery<br>• Amount of red blood cells (allogenic and autologous RBCs) infused after start of first IMP administration until end of surgery<br>• Post-operative blood loss in the first 24 hours<br>• Proportion (%) of subjects with rebleeds after the end of surgery until Day 8<br>• Hospital length of stay after surgery<br>• In-hospital mortality

- Safety	***Secondary Endpoints:***<br>• AEs<br>• Changes in vital signs<br>• Changes in clinical laboratory assessments of hematology, clinical chemistry, and urinalysis<br>• Changes in clinical laboratory assessments of markers of coagulation<br>• Changes in clinical laboratory assessments of coagulation factors<br>• Frequency and severity of thrombosis and of TEEs<br>• Virus status
**Biostatistical Concept**	Assuming a blood loss of about 500 mL in the FFP-/cryoprecipitate-treatment arm after the decision to treat the subject with IMP until the end of surgery, a standard deviation of 375 mL, a non-inferiority margin of 150 mL, and an alpha-level of 2.5% (1-sided) 100 evaluable subjects per treatment arm are needed to demonstrate the non-inferiority of BT524 by using a t-test with 80% power. The sample size was calculated with nQuery Advisor Version 4.0 or higher.<br>The primary endpoint is intra-operative blood loss after the decision to treat the subject with IMP until the end of surgery. The primary analysis of this endpoint will test for non-inferiority. The final analysis will be performed using analysis of covariance (ANCOVA) with the predictive blood loss (> 1,000 mL to ≤ 2,000 mL and > 2,000 mL) as a covariate. Non-inferiority will be demonstrated if the upper confidence limit of the 2-sided 95% confidence interval for the difference in the least square means is less than the non-inferiority margin (150 mL). If non-inferiority is demonstrated, then superiority will be assessed.<br>**Interim analyses:**<br>In this study, 3 interim analyses with an alpha-adjustment according to Haybittle/Peto (Haybittle, 1971; Peto et al., 1976; Schulz and Grimes, 2005) are planned. This leads to local alpha levels of 0.001 for each interim analysis, a significance level of 0.05 for the final analysis, and to an overall global alpha level of 0.05.<br>All interim analyses will be based on the per-prototocl set.<br>The first interim analysis is planned with approximately 50 spine subjects, the second one with at least 40 PMP subjects and all other evaluable spine subjects at that time-point. The third interim analysis is planned with approximately 80% of subjects of the total sample size.<br>Aim of all interim analyses is to adapt the sample size according to the observed blood losses and the standard deviations:

	a.) Early termination due to non-inferiority of BT524 in comparison with the used standard therapies.<br>b.) Continuation with the sample size as initially planned.<br>c.) Adjustment of sample size to take into account changes from the previous assumptions on the additional blood loss.<br>d.) Stopping the study early due to futility if the sample size re-estimation indicates a much higher number than planned before.<br><br>All secondary efficacy endpoints will be summarized descriptively by treatment arm.<br>The secondary endpoints of proportion of subjects with a successful correction of fibrinogen level and proportion of subjects with rebleeds will be compared between the treatment arms using a Cochran-Mantel-Haenszel (CMH) approach stratified by predictive blood loss.<br>The secondary endpoint 'time to first successful correction of fibrinogen level' will be compared between the treatment arms using a Chi-Square test.<br>The secondary endpoints of consumption of transfusion products, amount of RBCs and post-operative blood loss in the first 24 hours will be analysed using ANCOVA with the predictive blood loss as a covariate.<br>Safety will be assessed based on AEs, laboratory data, vital signs data, frequency and severity of thrombosis and of TEE and virus status which will be summarized descriptively.<br>An independent Data Safety Monitoring Board (DSMB) will review unblinded safety data at regular intervals during the study.<br>After 40 subjects have completed, the overall mean and standard deviation of the primary endpoint will be derived using blinded data of all 40 subjects without separating according to treatment to assess if the sample size needs to be adjusted.
**First Subject In**	Q1 2018
**Last Subject Last Visit**<br>*(planned)*	Tbd

Confidentiality Statement

This protocol is the property of Biotest AG, and may not be circulated, reproduced or published - either in whole or in part - without the company's written permission.

III. FLOWCHART OF STUDY

**Study Schedule** Day	**D-42 to D-1**	**D-2 to D1**	**D1**	**D1**	**D1**	**D1**	**D2**	**D3**	**D5**	**D8**	**D36** (+35)
**Assessments** Visit	**Screening**	**Prior surgery** (Baseline)	**Surgery** prior 1st dose	pre-dose	post-dose	end of surgery	**Follow-up**	**Follow-up**	**Follow-up**	**Follow-up**	**Closing**
Informed Consent	•										
Check/re-check of inclusion / exclusion criteria	•	•[1]									
Demographic data	•										
Classification of type of spine surgery	•										
Recording expected blood loss		•									
Body weight	•	•[2]									
Physical examination	•	•[2]					•	•	•	•	•
Pregnancy test, only in females of childbearing potential	•	•[2]									
Medical and surgical history	•	•[3]									
Viral safety: Collection of retention sample	•										•
Virus serology (hepatitis B, hepatitis C, HIV)	•										•
Vital signs	•	•	•	•	•	•	•	•	•	•	•
Hematology and clinical chemistry	•	•[2]	•			•	•	•	•	•	•
Urinalysis	•	•[2]					•	•		•	•
Markers of coagulation (coagulation activation tests)	•	•[2]	•		•[4]	•[5]	•	•	•	•	•
Plasma concentration of fibrinogen activity (Clauss assay)	•	•[2]	•		•[4]	•[5]	•				
FIBTEM A10 (ROTEM)	•	•[2]	•	•	•[4]	•[5]	•				
Maximum clot firmness (MCF) (ROTEM)	•	•[2]	•	•	•[4]	•[5]	•				

[1] Diagnostic tests will be repeated at the investigator's discretion.
[2] Diagnostic tests have to be done prior to surgery. In case of short time-period between screening and baseline (≤ 2 days) these tests have only to be repeated based on medical judgment of the investigator. If not repeated, screening results will serve as baseline.
[3] Previous medication: change from screening.
[4] Tests have to be done only 15 and 90 minutes after start of 1st IMP administration.
[5] Tests for *markers of coagulation and plasma activity of fibrinogen (Clauss assay, FIBTEM A10, MCF)* have to be done '90 min after start of 1st IMP administration' and at the 'end of surgery'. In case of a short time-period between these two time-points (<30 min) these tests have only to be repeated based on medical judgment of the investigator.

**Study Schedule** **Day**	**D-42 to D-1**	**D-2 to D1**	**D1**	**D1**	**D1**	**D1**	**D2**	**D3**	**D5**	**D8**	**D36** (+35)
**Assessments** **Visit**	**Screening**	**Prior surgery** (Baseline)	**Surgery**				**Follow-up**	**Follow-up**	**Follow-up**	**Follow-up**	**Closing**
			**prior 1st dose**	**pre-dose**	**post-dose**	**end of surgery**					
Coagulation factors		•	•		•[6]						
Intra-operative inclusion criteria			•								
**Intravenous infusion(s) of IMP (BT524 or FFP)**				•[7]							
Recording start/end of surgery			•			•					
Continuous measurement of blood loss from start of surgery				•		•	•				
Calculation and recording of blood loss						•[8]	•[9]				
Recording time of decision to treat the subject with IMP			•								
Order of IMP			•	•							
Randomization			•								
Rebleeding episodes						•	•	•	•	•	
Concomitant medication or treatment				•		•	•	•	•	•	•
Transfusion products				•		•	•	•	•	•	•
Adverse events	•	•		•		•	•	•	•	•	•

[6] Test has to be done only 90 minutes after start of 1st IMP administration.
[7] Total volume and total infusion time (start and end of each infusion) to be recorded.
[8] Intra-operative blood loss from time-point of decision to treat the patient with IMP until end of surgery.
[9] Recording of blood loss until 24 hours after end of surgery.

IV. TABLE OF CONTENTS

I. SIGNATURE PAGE ... 4
I.I Signature Page for Investigators ... 5
II. STUDY SYNOPSIS ... 6
III. FLOWCHART OF STUDY ... 11
IV. TABLE OF CONTENTS ... 13
V. LIST OF ABBREVIATIONS ... 17
1 INTRODUCTION ... 19
2 STUDY OBJECTIVES ... 22
3 STUDY DESIGN ... 23
4 STUDY POPULATION ... 28
4.1 Study Population, Diagnosis and Number of Subject ... 28
4.1.1 Gender Distribution ... 29
4.2 Inclusion Criteria ... 29
4.3 Exclusion Criteria ... 30
4.4 Subjects Withdrawal Criteria and Replacements ... 30
4.5 Subjects Information ... 31
4.6 Declaration of Informed Consent ... 32
5 INVESTIGATIONAL MEDICINAL PRODUCTS ... 32
5.1 Investigational Medicinal Product BT524 ... 32
5.1.1 Description of Investigational Medicinal Product BT524 ... 33
5.1.2 Formulation, Packaging and Labelling ... 33
5.1.3 Storage Conditions and Stability ... 33
5.1.4 Preparation for Use ... 33
5.2 Investigational Medicinal Product Fresh Frozen Plasma (FFP) ... 33
5.2.1 Description of Investigational Medicinal Product FFP ... 34
5.2.2 Formulation, Packaging and Labelling ... 34
5.2.3 Storage Conditions and Stability ... 34
5.2.4 Preparation for Use ... 34
5.3 Investigational Medicinal Product Cryoprecipitate ... 34
6 STUDY TREATMENT ... 35

6.1 Dosage Regimen 35

6.2 Dosage and Administration 35

6.3 Compliance with Dosage Regimens 37

6.4 Dose Justification 37

6.5 Treatment of Overdose 38

6.6 Randomization Code 38

6.7 Procedures for Emergency Unblinding 39

6.8 Drug Accountability 39

6.9 Previous and Concomitant Medication or Treatment 40

6.10 Prohibited Medication or Treatment 40

6.11 Warnings and Precautions 40

7 COURSE OF THE CLINICAL STUDY 42

7.1 Visit Schedule 42

7.2 Duration of the Clinical Study 49
7.2.1 End of Study 49

7.3 Criteria for Premature Termination 50
7.3.1 Premature Termination of the Entire Clinical Study 50
7.3.2 Premature Termination of an Individual Study Site 50

7.4 Treatment and Care after the End of the Study 50

8 BENEFIT-RISK EVALUATION 51

8.1 Benefit of BT524 51

8.2 Foreseeable Risk and Discomfort Related to BT524 51

8.3 Other Sources of Possible Risk and Discomfort 51

8.4 Summary of Possible Risk and Discomfort 51

9 ASSESSMENT OF OBJECTIVES / CRITERIA FOR EVALUATION 52

9.1 Efficacy 52
9.1.1 Specification of Efficacy Parameters 52
9.1.1.1 Primary Efficacy Parameter 52
9.1.1.2 Secondary Efficacy Parameter 52
9.1.2 Methods for Assessing and Recording Efficacy Parameters 53
9.1.2.1 Methods for Assessing Primary Efficacy Parameter 53
9.1.2.2 Methods for Assessing Secondary Efficacy Parameter 54

9.1.3 Specification of Efficacy Endpoints 56
9.1.3.1 Specification of Primary Efficacy Endpoint 56
9.1.3.2 Specification of Secondary Efficacy Endpoints 56

9.2 Safety 57
9.2.1 Specification of Safety Parameters 57
9.2.1.1 Adverse Events 58
9.2.1.2 Physical Examination....58
9.2.1.3 Vital Signs 58
9.2.1.4 Laboratory Parameters 59
9.2.1.5 Retention Samples 63
9.2.2 Methods for Assessing and Recording Safety Parameter(s)....64
9.2.3 Safety Endpoints 64

9.3 Adverse Events 64
9.3.1 Definitions....64
9.3.2 Recording Adverse Events 66
9.3.3 Period of Observation 67
9.3.4 Assessment of Adverse Events....68
9.3.5 Immediate Reporting by Investigator to Sponsor 69
9.3.6 Use of IMP outside the Specifications of the Clinical Study Protocol 69
9.3.7 Investigational Medicinal Product Complaints....70
9.3.8 Special Situations Requiring Immediate Reporting 70
9.3.8.1 Pregnancy 70

9.4 Data Safety Monitoring Board.... 71

10 STATISTICS.... 72

10.1 Analysis Sets.... 72

10.2 Protocol Deviations 72

10.3 General Considerations 73

10.4 Efficacy Analyses 73

10.5 Primary Efficacy Analysis 74

10.6 Secondary Efficacy Analyses 74
10.6.1 Correction of Fibrinogen Level....75
10.6.2 Consumption of Transfusion Products....75
10.6.3 Amount of Red Blood Cells....75
10.6.4 Post-operative Blood Loss 75
10.6.5 Proportion of Subjects with Rebleeds 76
10.6.6 Hospital Length of Stay after Surgery....76
10.6.7 In-hospital Mortality....76

10.7 Safety Analysis 76

10.8 Interim Analyses 76

10.9 Determination of Sample Size.... 77
10.9.1 Data Monitoring 78

11 DATA MANAGEMENT 78

11.1 Data Collection 78

11.2 Correction of Data 79

11.3 Data Handling 79

12 QUALITY CONTROL AND QUALITY ASSURANCE 79

12.1 Study Initiation Activities 79

12.2 Training of site staff 79

12.3 Documentation and Filing 80

12.4 Monitoring 80

12.5 Audits and Inspections 81

12.6 Archiving 81

13 GENERAL REGULATIONS, AGREEMENTS AND ORGANISATIONAL PROCEDURES 81

13.1 Study Administrative Structure 81

13.2 Ethical and Regulatory Considerations 81

13.3 Committees / Monitoring Boards 82

13.4 Written Agreements 82

13.5 Insurance/Liability 82

13.6 Investigator's Brochure (IB) 82

13.7 Amendments to the Protocol 82

13.8 Confidentiality 82

13.9 Final Report and Publication 83

14 LIST OF REFERENCES 84

15 APPENDICES 91

Figure 1: Overview of Study Design 24
Figure 2: Quantification of Blood Loss Intra-operatively 25
Figure 3: Repeated IMP Administration 27
Figure 4: ROTEM Readout of Citrated Normal Blood 55

Table 1: Examples of Major Spine Surgeries with Expected Large Volume of Blood Loss 28
Table 2: Clinical Laboratory Parameters 60

V. LIST OF ABBREVIATIONS

ADR	Adverse Drug Reaction
AE	Adverse Event
AESI	Adverse Event of Special Interest
ALAT	Alanine aminotransferase
ANCOVA	Analysis of Covariance
AP	Alkaline phosphatase
aPTT	Activated partial thromboplastin time
ASAT	Aspartate aminotransferase
AT III	Antithrombin III
BDRM	Blind Data Review Meeting
BUN	Blood Urea Nitrogen
BW	Body Weight
CDS	Corporate Drug Safety
CHMP	Committee for Medicinal Products for Human Use
CMH	Cochran-Mantel-Haenszel
CSP	Clinical Study Protocol
CSR	Clinical Study Report
CRO	Contract Research Organization
DEVP	Drug Exposure Via Parent
DIC	Disseminated Intravascular Coagulation
DOAC	Direct Oral Anti-Coagulants
DSMB	Data Safety Monitoring Board
eCRF	electronic Case Report Form
EDC	Electronic Data Capture
EMA	European Medicines Agency
IEC	Ethics Committee
IRB	Institutional Review Board
FAS	Full Analysis Set
FII	Factor II
FV	Factor V
FVII	Factor VII
FVIII	Factor VIII
FIX	Factor IX
FX	Factor X
FXI	Factor XI
FXIII	Factor XIII
F_{1+2}	Prothrombin Fragments 1+2
FAS	Full Analysis Set
FFP	Fresh Frozen Plasma
GCP	Good Clinical Practice
ɣ-GT	Gamma glutamyltransferase
IB	Investigator’s Brochure
HAV	Hepatitis A Virus
HBV	Hepatitis B Virus
HCV	Hepatitis C Virus
HEV	Hepatitis E Virus

HIV	Human Immunodeficiency Virus
ICF	Informed Consent Form
IMP	Investigational Medicinal Product
ICH	International Conference on Harmonization
i.e.	id est
INR	International normalized ratio
IRAE(s)	Immediately Reportable Adverse Event(s)
IV	Intravenous
IWRS/IVRS	Interactive Web/Voice Response System
MCF	Maximum Clot Firmness
MedDRA®	Medical Dictionary for Regulatory Activities
NTEAE	Non-Treatment Emergent Adverse Event
NIMP	Non-Investigational Medicinal Product
PC	Protein C
PEI	Paul-Ehrlich-Institut, Germany
PHI	Protected Health Information
PPS	Per-Protocol Set
PS	Protein S
PMP	Pseudomyxoma peritonei
PT(INR)	Prothrombin Time (International Normalized Ratio)
QBL	Quantification of Blood Loss
RBC	Red Blood Cells
ROTEM	Rotational Thromboelastometry
SAE	Serious Adverse Event
SAF	Safety Analysis Set
SAP	Statistical Analysis Plan
SAS	Statistical Analysis Software
SmPC	Summary of Product Characteristics
SOC	System Organ Class
TAT	Thrombin-antithrombin III complex
TEAE	Treatment-Emergent Adverse Event
TEE	Thromboembolic Event
TMF	Trial Master File
TT	Thrombin Time
TXA	Tranexamic Acid
vWF	von Willebrand factor
WBC	White Blood Cells

1 INTRODUCTION

BT524 is a lyophilized, heat-treated fibrinogen concentrate manufactured from human plasma. BT524 is currently developed for the treatment and prophylaxis of bleeding in patients with congenital afibrinogenaemia or severe congenital hypofibrinogenaemia with bleeding tendency. Moreover, BT524 will be developed as complementary therapy to management of uncontrolled severe hemorrhage in acquired hypofibrinogenaemia.

To date, no clinical studies have previously been conducted with BT524 in subjects with acquired hypofibrinogenaemia. The pharmacokinetic properties of BT524 were investigated in the treatment and prophylaxis of bleeding in patients with congenital fibrinogen deficiency (afibrinogenaemia or severe hypofibrinogenaemia) in the ongoing prospective, open-label, phase I/III study PPD Details on the clinical pharmacology of BT524 and further information on non-clinical studies with BT524 are provided in the Investigator's Brochure.

In addition to the ongoing clinical development in congenital fibrinogen deficiency, the present prospective, multi-center, randomized, active-controlled, pivotal phase III study aims to demonstrate the efficacy and safety of BT524 in subjects with acquired hypofibrinogenaemia caused by major surgery associated with major blood loss.

Fibrinogen (coagulation factor I) is a soluble plasma glycoprotein synthesized by hepatic parenchymal cells. The normal blood fibrinogen concentration is between 2.0 and 4.5 g/L although this range can vary (Levy and Goodnough, 2015). Fibrinogen plays a central role by clot forming in wound healing and furthermore, is important in primary hemostasis as it contributes to blood platelet aggregation. In case of a fibrinogen deficiency the blood coagulation is disordered, which leads to (severe) hemorrhagic events.

Acquired fibrinogen deficiency is the most common type of fibrinogen deficiency. It is characterized by an impaired hemostatic function caused by fibrinogen concentrations below the normal ranges, classified as hypofibrinogenaemia. Hypofibrinogenaemia results from either reduced fibrinogen synthesis due to hepatic disorders, increased intravascular consumption due to a breakdown of fibrinogen, disseminated intravascular coagulation (DIC) or increased fibrinogen loss caused by certain medical conditions such as surgical procedures or uncontrolled life-threatening bleeding. Acquired fibrinogen deficiency can cause severe intra-operative bleeding. Depending on the severity and the extent of the event (i.e. trauma, surgery) and the patient's clinical condition fibrinogen plasma concentrations are highly variable in acquired fibrinogen deficiency.

Acquired fibrinogen deficiency is associated with increased morbidity and mortality. Thus, effective management of this hemostatic disorder is necessary to prevent potentially life-threatening bleeding, to reduce increased blood loss, transfusion requirements and the risk of surgery (Fenger-Eriksen et al., 2009).

Fibrinogen is an important contributor to clot strength and is the first coagulation factor to become critically reduced during intra-operative hemorrhage (Haas et al., 2012). Therefore, a rapid and accurate determination of fibrinogen level is important during hemorrhage to establish a timely hemostatic intervention. The rapid fibrinogen supplementation to restore plasma levels is an important component for normalizing clot formation in bleeding patients and maintaining fibrinogen levels is an important therapeutic target in bleeding, particular in intra-operative settings (Levy and Goodnough, 2015).

There is growing evidence that fibrinogen levels > 1.5 to 2 g/L are necessary to control major bleeding in the intra-operative settings (Haas et al., 2012; Levy and Goodnough, 2015). Accordingly, European trauma guidelines from 2013 (Spahn et al., 2013) and the guidelines from the European Society of Anaesthesiology (Kozek-Langenecker et al., 2013) recommend target levels of at least 1.5 to 2.0 g/L in intra-operative settings. Because of the large variability in fibrinogen concentrations among bleeding patients, individualized dosing of fibrinogen concentrate based upon both the level of bleeding and the plasma fibrinogen concentration are recommended (Levy et al., 2012). Fibrinogen concentrate infusion guided by point-of-care tests is recommended by the European Society of Anaesthesiology (Kozek-Langenecker et al., 2013) and the FIBTEM test has been used extensively in clinical studies to determine fibrinogen levels and calculate dosing (Levy and Goodnough, 2015).

Nevertheless, the optimal treatment level, the use of pre-emptive treatment and the preferred source of fibrinogen for acquired fibrinogen deficiency remain disputed. Fibrinogen concentrate is increasingly used and recommended for bleeding with acquired hemostatic deficiencies in several countries, but evidence is inconsistent regarding surgery settings, dosing and efficacy. Fresh frozen plasma (FFP) also contains fibrinogen and is available in all hospital settings and is comparatively cheap but requires the administration of large quantities of FFP to achieve a reasonable fibrinogen dose. Therefore, further clinical studies investigating fibrinogen replacement in acquired fibrinogen deficiency are needed.

Currently, conventional replacement therapy in fibrinogen deficiency consists of transfusion of allogenic blood products such as **FFP** and **cryoprecipitate.**

FFP is a blood product that has been available since 1941. Initially used as a volume expander, it is currently indicated for the management and prevention of bleeding in coagulopathic patients (Nascimento et al., 2010).

FFP is the liquid portion of blood that contains all the clotting factors, as well as other blood proteins and that is stored by freezing (WHO 2007). FFP is usually authorized nationally. Examples of FFP authorized in Germany are available from the website of the German authority Paul-Ehrlich-Institut (PEI). A solvent/detergent treated frozen plasma (PPD by PPD) is nationally approved in several EU countries (PPD).

Compared to fibrinogen concentrate FFP is stored frozen and must be thawed before use which is a limitation in time critical and potentially life-threatening situations such as severe bleeding. FFP contains 2.0 to 4.5 g/L of fibrinogen which is much lower than in fibrinogen concentrates (i.e. up to 10-fold lower than in BT524 with 20 g/L). Since FFP contains relatively low amounts of fibrinogen, it requires the administration of large volumes to provide meaningful increases in the fibrinogen plasma level. The large volumes carry the risk of hypervolemia, cardiac stress, circulatory overload, transfusion-related acute lung injury, hypothermia and metabolic complications once bleeding resolves (Bornikova et al., 2011; Elliott and Aledort, 2013; Mumford et al., 2014; Ofosu et al., 2008). Furthermore, the use of allogenic blood confers an additional risk for blood borne pathogens. Also noteworthy is the risk for transfusion related reactions, immune suppression, and a decrease in coagulation factors. There is also evidence that transfusion of allogenic blood is increasingly harmful as more blood is transfused (Verma et al., 2015).

As the concentration of fibrinogen in fibrinogen concentrates is markedly higher than in plasma the required dose to reach the fibrinogen target concentration can be administered in a minor volume. Furthermore, the amount of fibrinogen in FFP varies making it difficult to predict the increase in plasma fibrinogen concentration. The precisely defined fibrinogen content in fibrinogen concentrates allows accurate calculation of the amount of reconstituted concentrate needed for a targeted fibrinogen supplementation.

Cryoprecipitate is made from FFP and contains various proteins including fibrinogen. It contains higher concentrations of fibrinogen than FFP and some (but not all) coagulation factors, so less volume is needed. The minimum amount of fibrinogen required by standards of the American Association of Blood Banks is 150 mg per bag of cryoprecipitate; current preparations yield a median of 388 mg per bag, but the amount of fibrinogen is variable and cannot be determined accurately. A single unit of cryoprecipitate also contains variable amounts of FVIII, FXIII, von Willebrand factor (vWF), fibronectin and platelet microparticles (Callum et al., 2009). Therefore, the use of cryoprecipitate for fibrinogen replacement alone exposes the patient to potentially unneeded coagulation proteins which could increase the risk of thrombosis (Elliott and Aledort, 2013; Franchini and Lippi, 2012). Cryoprecipitate was withdrawn from most European countries some years ago because of safety concerns (Karkouti et al., 2018; Schochl et al., 2013) but remains available in Scandinavia, the UK and the USA, essentially for the purpose of fibrinogen replacement.

In summary fibrinogen concentrate seems to have certain advantages over other replacement therapies like FFP and cryoprecipitate, such as precisely determined high amounts of purified fibrinogen dissolved in a small volume, low risk of pathogen transmission and instant administration without need for thawing or testing AB0 blood group compatibility (Warmuth et al., 2012).

A number of clinical studies have been published in subjects with acquired hypofibrinogenaemia, such as following trauma, cardiothoracic surgery and obstetric hemorrhage, documenting that fibrinogen concentrate is able to improve clotting function and reduce blood loss. The results of these studies have shown that fibrinogen concentrate raises the levels of fibrinogen and improves clot firmness. Additionally, fibrinogen substitution has been reported to reduce bleeding and post-operative transfusion requirements. Published data from completed clinical studies in subjects undergoing major surgeries not only suggest that fibrinogen plays a critical role in achieving and maintaining hemostasis; in particular, they appear to show benefit of individualized dosing of fibrinogen concentrate using a target ROTEM/FIBTEM value integrating rapid diagnostic testing with appropriate therapeutic dosing of fibrinogen concentrate in line with patients' needs (Haas et al., 2015; Rahe-Meyer et al., 2013a; Rahe-Meyer et al., 2009a; Rahe-Meyer et al., 2013b; Rahe-Meyer et al., 2009b; Ranucci et al., 2015). In a prospective, randomised, single-center, controlled phase 2 study, published in 2019 (PPD), 45 adult subjects undergoing cytoreductive surgery for pseudomyxoma peritonei (PMP) were treated pre-emptively with fibrinogen concentrate or cryoprecipitate. Subjects were randomised to one of the two treatment groups (4 g fibrinogen concentrate or 2 pools cryoprecipitate), when assessment after the start of surgery predicted intra-operative blood loss ≥ 2 L without targeted fibrinogen replacement. Further intra-operative doses were based on thromboelastometry (FIBTEM A20 < 12 mm). Haemostatic efficacy was successful in 100% of subjects in both groups, with similar blood loss. No thromboembolic events (TEEs) occurred in subjects who received fibrinogen concentrate.

The published data indicate that fibrinogen concentrate is at least comparable with cryoprecipitate in terms of benefits for haemostatic therapy in the treatment of clinically relevant bleeding associated with acquired fibrinogen deficiency in subjects undergoing cytoreductive surgery for PMP (Roy et al., 2019).

As there is no guideline on the clinical investigation of fibrinogen in patients with uncontrolled severe hemorrhage in acquired hypofibrinogenaemia available, the EMA guideline on core SmPC for human fibrinogen products (EMA, 2015a) has been taken into account when planning this phase III study in acquired fibrinogen deficiency. Furthermore, currently ongoing clinical studies with fibrinogen concentrate as well as recently finalized clinical studies in this clinical setting (Roy et al., 2019) have been considered.

BT524 will be developed as complementary therapy to management of uncontrolled severe hemorrhage in acquired hypofibrinogenaemia caused by major surgeries associated with major blood loss. As it is expected that BT524 will show a safety advantage over the standard treatment with FFP/cryoprecipitate, an efficacy comparison to the standard is required to allow a risk-benefit assessment to be made for BT524.

Therefore, the present prospective, multi-center, randomized, active-controlled, pivotal phase III non-inferiority study aims to investigate efficacy and safety of BT524 in subjects with acquired hypofibrinogenaemia.

2 STUDY OBJECTIVES

The main purpose of this phase III study is to demonstrate the efficacy of BT524 as a complementary therapy to management of uncontrolled severe hemorrhage in acquired hypofibrinogenaemia in subjects undergoing elective major spinal or abdominal surgery.

The primary objective of this study is to demonstrate that BT524 is non-inferior that means not worse than FFP/cryoprecipitate with a non-inferiority margin of 150 mL in reducing intra-operative blood loss by IV administration in subjects with acquired hypofibrinogenaemia undergoing elective major spinal or abdominal surgery.

If therapeutic equivalence (non-inferiority) has been demonstrated, therapeutic superiority of BT524 compared with FFP/cryoprecipitate will also be assessed.

Secondary objectives are to demonstrate the efficacy of BT524 by assessing the correction of the fibrinogen level intra-operatively, the transfusion requirements, post-operative blood loss in the first 24 hours, the number of subjects with rebleeds, hospital length of stay and in-hospital mortality. Secondary objectives also comprise the safety of BT524 by documenting the number of AEs including changes in laboratory parameters, the virus status, and the frequency and severity of thrombosis and of TEEs.

3 STUDY DESIGN

This is a phase III, prospective, randomized, active-controlled, multicenter, non-inferiority clinical study in subjects undergoing major spinal or abdominal surgery to demonstrate the efficacy and the safety of intra-operative use of BT524 as a complementary therapy to management of uncontrolled severe hemorrhage in acquired hypofibrinogenaemia.

This non-inferiority study is focused on the **primary objective**, to demonstrate that BT524 is non-inferior that means not worse than FFP/cryoprecipitate with a non-inferiority margin of 150 mL in reducing intra-operative blood loss by IV administration in subjects with acquired hypofibrinogenaemia undergoing elective major spinal or abdominal surgery. If therapeutic equivalence has been demonstrated, therapeutic superiority of BT524 compared with FFP/cryoprecipitate will also be assessed.

Secondary objectives are to demonstrate the efficacy of BT524 by assessing the correction of fibrinogen level during surgery, the transfusion requirements, post-operative blood loss in the first 24 hours, the number of subjects with rebleeds, the hospital length of stay and the in-hospital mortality. Secondary objectives also comprise the safety of BT524 by documenting the number of AEs including changes in laboratory parameters, the virus status, and the frequency and severity of thrombosis and of TEEs.

The study comprises a screening visit within 42 days prior to surgery to assess subjects eligibility, a baseline visit on the day of the surgery prior anaesthesia (Day 1, but if required this could be Day -2 or Day -1 due to local hospital procedures), the surgery phase (including randomization, Day 1) and the follow-up phase of at least 5 weeks with 4 follow-up visits on Days 2, 3, 5 and 8 and the closing visit, including the final safety examination, scheduled on Day 36* after the day of surgery (*+35, up to Day 71 if required). The duration of individual study participation for eligible screened subjects is at least 5 weeks.

The study design for subjects undergoing spine surgery is shown in the following figure (Figure 1):

Figure 1: Overview of Study Design

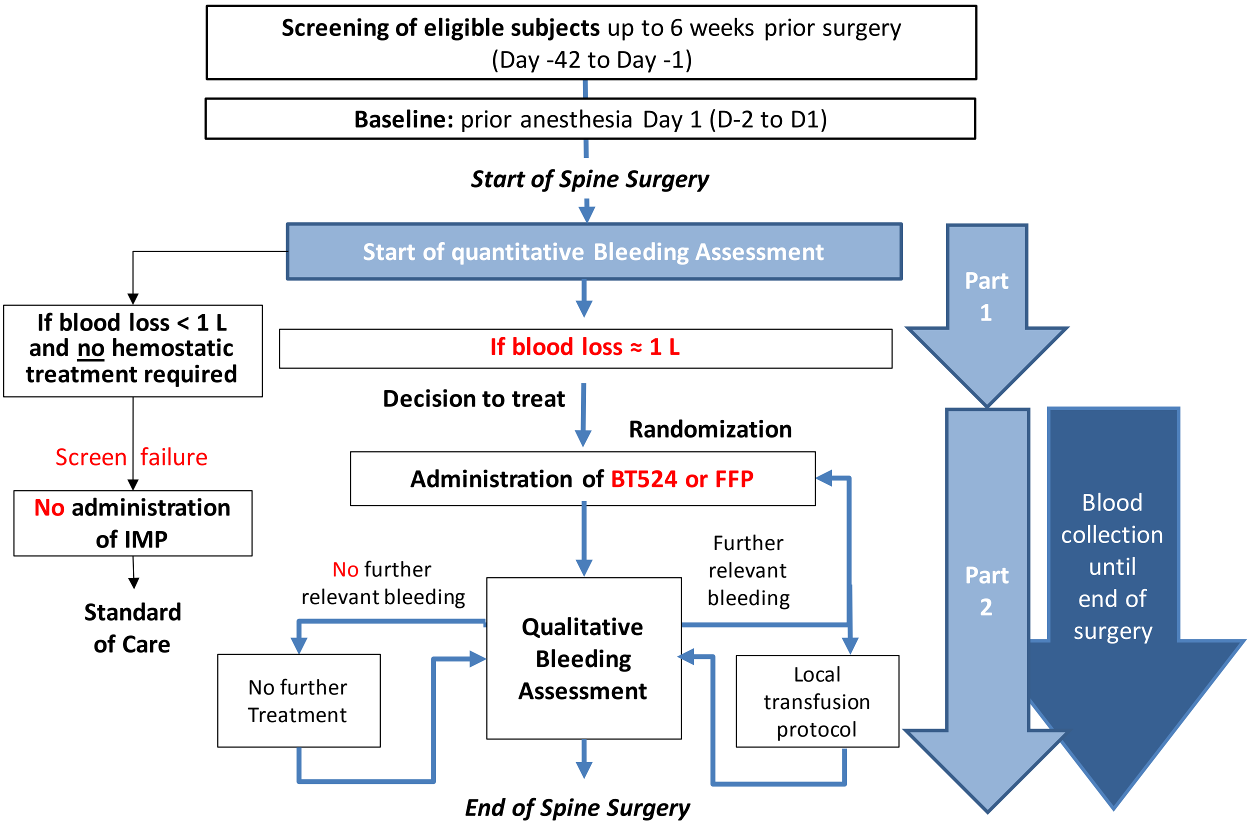

Further details on the assessment schedule (including the follow up period) that will be used for the assessment of the efficacy and safety parameters in this study are presented in the flowchart in section III and in the visit schedule section 7.1.

At least 200 subjects will be enrolled to ensure data are available for 100 evaluable subjects per treatment arm (BT524 or FFP/cryoprecipitate). The multicenter, multinational study will be conducted in approximately 15-20 sites in Europe (subjects undergoing major spinal surgery) and in one site in the United Kingdom (subjects undergoing cytoreductive PMP surgery).

Detailed Description of the Spinal Surgery Phase

The continuous determination/measurement of blood loss during the entire surgery will be separated into two parts:

- **First part:** blood loss will be determined from start of surgery until decision to treat by **estimating** the bleeding mass in the blood suction unit, taking the blood loss in surgical cloths and compresses into account. This is the prerequisite for the intra-operative inclusion criterion (clinically relevant blood loss of approximately 1 L, requiring hemostatic treatment) and the decision to treat the subject with IMP.
- **Second part:** blood loss will be **measured** after decision to treat until end of surgery and represents the blood loss considered for the primary objective. During this part of the surgery the blood loss will be quantified by measuring the continuous bleeding mass removed from the surgical field by a blood suction unit (and/or a cell saver) and by calculation of the amount of blood absorbed by surgical cloths and compresses. In a final step, the total blood loss will be calculated.

Prior to the start of surgery dry surgical cloths and compresses will be weighed (weight recorded), to be available in case a manual compression is required.

The **first part** of blood measurement starts at the start of surgery with the collection of blood in the blood suction unit: The blood is salvaged by a suction catheter from the operating field. This blood is suctioned into a reservoir which contains a heparinised saline solution (or citrate anticoagulant solution) used for anticoagulation during blood collection. The quantity of anticoagulant introduced into the blood collection system will be adapted continuously to the volume of blood loss.

In case a manual compression is necessary, dry surgical cloths and compresses will be applied to the surgical field, and - at the latest - prior to the decision to treat the subject with IMP removed. By wringing out surgical cloths, the blood can be caught in a kidney dish and finally also be collected in the suction container.

The anaesthesiologist will estimate the blood volume collected in the suction container continuously, taking the blood loss in surgical cloths and compresses into account. Allowance must be made for the presence of heparinised saline solution and irrigation solution. During the surgery, the anaesthesiologist must calculate the amount of solution suctioned into the container through irrigation of the wound. This will be done by knowing the capacity of the irrigation syringe in use and keeping track of the number of times it is used. The anaesthesiologist subtracts these amounts **to estimate the volume of blood in the container.**

The quantification of blood loss (QBL) intra-operatively is shown in Figure 2:

Figure 2: Quantification of Blood Loss Intra-operatively

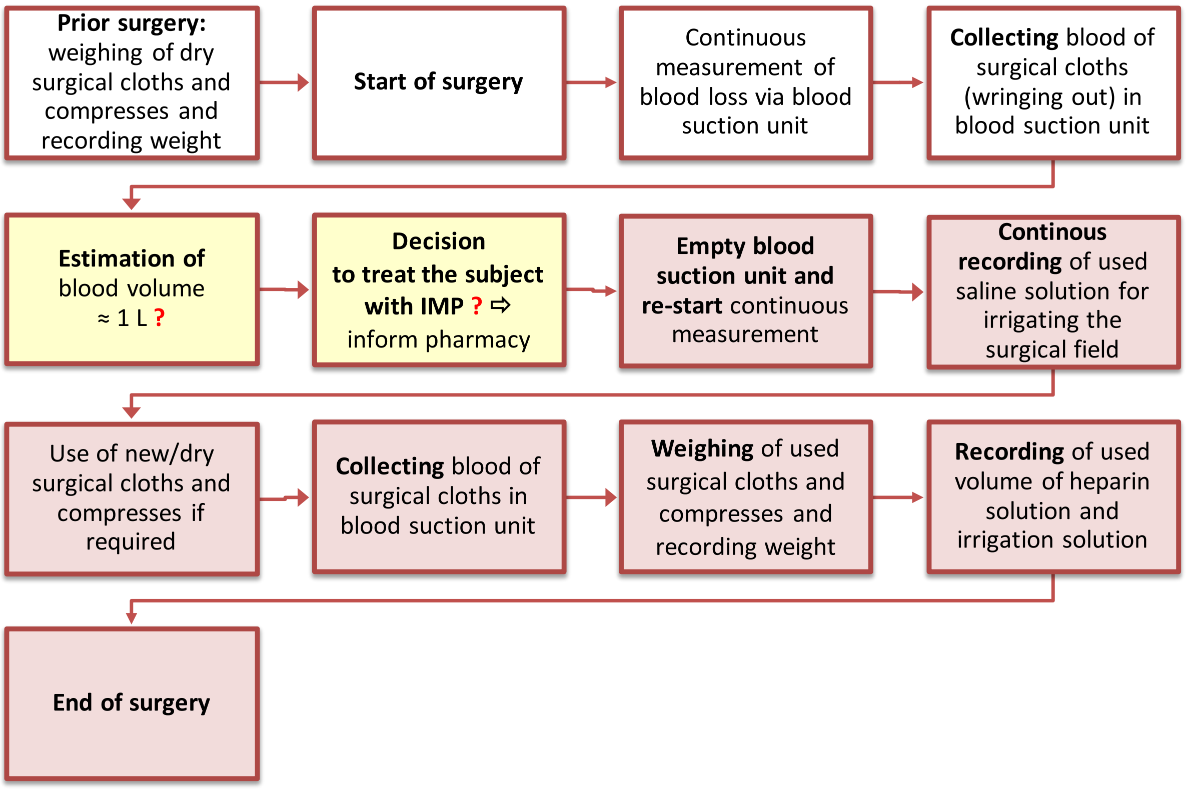

After an estimated blood loss of approximately 1 L, and the assessment that hemostatic treatment will be required during surgery (high risk for the need of fibrinogen supplementation, either with FFP or BT524), the decision to treat the subject with IMP can be made.

At this point the **time has to be documented**.

Immediately after an estimated blood loss of approximately 1 L, requiring haemostatic treatment during surgery, the pharmacy will be informed about the decision to treat the subject with IMP. The subject will be randomized to one of the treatment arms.

At the same time, the **suction container will be emptied** and all remaining surgical cloths and compresses will be removed from the surgical area.

Blood lost within the surgical field is collected into the blood suction container and the collection can be undertaken with or without further processing via cell saver and reinfusion. Cell savers separate the RBCs by centrifugation, and reinfuse the RBCs. The packed RBCs are collected in a separate bag. The collected RBCs can be reinfused according to institutional practice.

Cell salvage is not recommended by the manufacturers in patients undergoing surgery for malignancy because of the possibility of reinfusion of tumor cells.

The **second part of blood measurement** is initiated with collection of blood in the empty blood suction unit. In addition, in case a manual compression is necessary after decision to treat the subject with IMP, new surgical cloths and compresses will be applied to the surgical area.

Dosing of BT524 will be guided by FIBTEM A10 results and the dose will be calculated according to the predefined formula. However, the first BT524 dose is at least 2 g. The recommended therapeutic dose of FFP is 15 mL per kg BW. IMP dose will be prepared and delivered to the operating room by the pharmacy according to local standards and administered by the unblinded anaesthesiologist.

The content of each syringe with BT524 (1 g fibrinogen concentrate dissolved in 50 mL water for injection) can be administered in less than 20 seconds. FFP can be infused as rapidly as possible by the anaesthesiologist.

In the further course of the surgery, qualitative assessment of bleeding will be used by the spine surgeon to estimate if further bleeding intervention is required. Spine surgeon and surgical staff are blinded to therapy and will inform the anaesthesiologist regarding the bleeding assessment. In addition, FIBTEM A10 should be determined in order to calculate individual BT524 doses. If no further relevant bleeding occurred, surgery will be proceeded to completion.

If the bleeding remained unchanged and an ongoing relevant blood loss will be confirmed or the hemostatic control is not considered sufficient and requires further intervention or a new major blood loss occurred in the course of the surgery, subjects will be treated with repeated IMP administration according to their randomized treatment group or using a transfusion protocol according to local standards.

At the end of the surgery, all surgical cloths and compresses will be removed from the surgical area, wrung out until almost dry (blood will be collected) and weighed (weight will be recorded). The end of surgery is defined as time of last suture.

The fluid volume collected in the blood suction container will be measured and recorded, the proportion of blood, heparinised saline solution and irrigation solution will be calculated and also recorded.

Standardized Transfusion Algorithm

Tranexamic Acid (TXA) can be administered prophylactically in all subjects according to local standards, e.g. TXA 1 g at the start of surgery as a single IV infusion, followed by a further dose of 1 g TXA 6 hours after first dose, if required (in case the surgery is still ongoing).

Repeated IMP Administration

According to the study design and the described treatment algorithm BT524 or FFP will be administered as the primary haemostatic therapy if subjects have clinically relevant bleeding intra-operatively after completion of surgical haemostasis.

If bleeding continued after completion of IMP administration, a transfusion protocol according to local standards can be followed. Repeated intra-operative administration of IMP is possible depending on the subjects' clinical condition and their individual FIBTEM A10 results (Figure 3).

Figure 3: Repeated IMP Administration

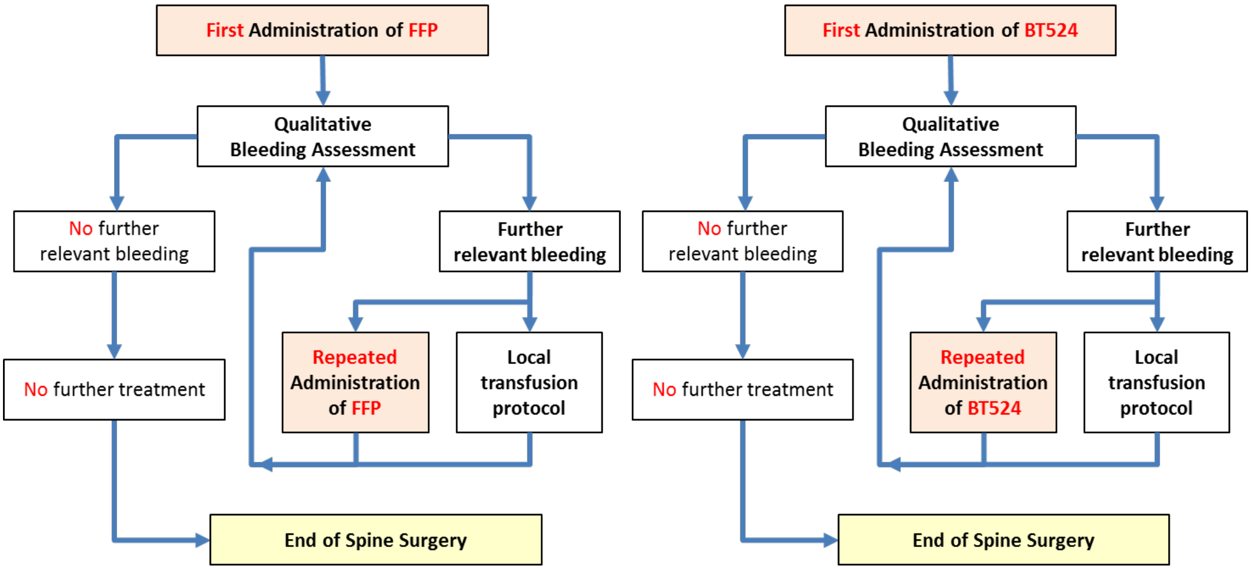

The decision to transfuse allogeneic or autologous blood products in the intra-operative period depends on the concentration of hemoglobin, the amount and speed of the blood loss and the clinical condition of the subject at the discretion of the surgeon and/or the anaesthesiologist.

The blood components that can be used for transfusion support are autologous RBC concentrates, derived from intra-operative salvage, allogeneic RBCs, platelet concentrates and allogeneic FFP.

The RBCs collected intra-operatively can be reinfused according to institutional practice.

Furthermore, plasma-derived drugs (e.g. albumin) can be used according to local standards. The total volume (number of units and volume per unit) of all blood components (e.g. RBCs, platelets, FFP) or plasma derivatives (such as albumin and coagulation factors) given intra-operatively will be recorded.

4 STUDY POPULATION

4.1 Study Population, Diagnosis and Number of Subject

A total of at least 200 adult subjects (≥ 18 years) of both gender undergoing elective spinal or abdominal surgery with expected major blood loss are planned to be treated within this study. Eligibility is defined by the inclusion and exclusion criteria as described below. The type of spine surgery is not restricted by the eligibility criteria to surgery for an expected major blood of approximately 1 L. The following table gives examples of the types of spine surgery often associated with severe hemorrhage (Table 1):

Table 1: Examples of Major Spine Surgeries with Expected Large Volume of Blood Loss

**Surgeries on spinal meninges and spinal cord**
• access to craniocervical junction and cervical spine, dorsal > 2 vertebral segments
• access to thoracic spine, dorsal > 2 vertebral segments
• upper thoracic spine ventral via sternotomy
• access to lumbar spine, dorsal > 2 vertebral segments
**Spondylodesis (Spinal Fusion)**
• spinal fusion, dorsal > 2 vertebral segments
• spinal fusion, dorsal and ventral approach > 3 vertebral segments
• spinal fusion, ventral approach > 3 vertebral segments
**Vertebral body replacement and complex spine reconstruction**
**Other complex spine reconstructions**
• corrective spinal fusion with instrumentation, dorsal
• corrective spinal fusion with instrumentation, ventral
• corrective spinal fusion with instrumentation, dorsal and ventral
**Release and scoliosis deformity correction**
**Complex 360°-reconstruction with fusion, ventrodorsal procedure**
**Complex 360°-reconstruction with fusion, ventrodorsal procedure after tumor resection**
**Bony decompression of the spinal canal, ≥ 4 vertebral segments**
**Re-operations**

4.1.1 Gender Distribution

There are no gender-based enrolment restrictions applicable for the study i.e., male and female subjects are intended to be included.

Since men and women undergoing elective surgery might suffer from acquired hypofibrinogenaemia, subjects of both genders should be included into the study. Equitable inclusion of both genders in research is important to ensure that both receive a proportionate share of benefits of research and that neither bears a disproportionate burden.

Women of childbearing potential are allowed to participate when using reliable/ effective contraceptive method(s) during the study and at least one month after the last administration of study drug. However, pregnant women are to be excluded (see general exclusion criterion 1).

4.2 **Inclusion Criteria**

Only subjects meeting all of the following inclusion criteria will be considered for study inclusion:

Inclusion Criteria	Rationale	Screening	Intra-operatively
1. Written informed consent obtained from subjects indicating that they understand the purpose of and procedures required for the study and are willing to participate in it	Ethical aspects	X	
2. Subjects scheduled for elective major spinal or cytoreductive PMP surgery with expected major blood loss	Disease requirement	X	
3. Male or female, aged ≥ 18	Study population requirement	X	
4. No increased bleeding risk as assessed by standard coagulation tests and medical history *	Pre-treatment requirement	X	
5. Intra-operative trigger for treatment<br>a. <u>Subjects undergoing spinal surgery:</u> Intra-operative clinically relevant bleeding of approximately 1 L, requiring hemostatic treatment during surgery **<br>b. <u>Subjects undergoing cytoreductive PMP surgery:</u> Intra-operative prediction of clinically relevant bleeding of > 2 L, requiring hemostatic treatment during surgery ***	Pre-treatment requirement		X

* Inclusion criterion no. 4 aims to ensure that only subjects <u>without hereditary bleeding disorders</u> are to be included in this study.

Subjects with continuous ‘aspirin intake’, subjects under Direct Oral Anti-Coagulants (DOACs), or with a hepatic disease are at higher bleeding risk. Nevertheless, these subjects can be included at the discretion of the investigator.

** A clinically relevant bleeding, resulting in a high risk for the need of fibrinogen supplementation with BT524 or FFP during surgery. The amount of clinically relevant blood loss depends on the subject’s clinical condition, e.g. underlying disease, significant comorbidities, low body weight, relevant change of a laboratory value.

*** Only applicable for subjects in the UK.

4.3 Exclusion Criteria

Subjects having any of the following criteria, either at screening and/or at baseline will not be included in the study:

Exclusion Criteria	Rationale	Screening	Baseline (prior start of surgery)
1. Pregnancy or unreliable contraceptive measures or breast feeding (women only)	Lack of suitability due to not yet established safety in a clinical study	X	
2. Hypersensitivity to proteins of human origin or known hypersensitivity reactions to components of the IMP	Lack of suitability for study due to safety reasons	X	
3. Participation in another clinical study within 30 days before entering the study or during the study and/or previous participation in this study	Lack of suitability for study	X	
4. Treatment with any fibrinogen concentrate and/or fibrinogen-containing product within 30 days prior to infusion of IMP	Lack of suitability for study	X	
5. Employee or direct relative of an employee of the CRO, the study site, or Biotest	Ethical aspects	X	
6. Inability or lacking motivation to participate in the study	Subject compliance	X	
7. Medical condition, laboratory finding (e.g. clinically relevant biochemical or hematological findings outside the normal range), or physical exam finding that in the opinion of the investigator precludes participation	Lack of suitability for study due to safety reasons	X	
8. Presence or history of venous/arterial thrombosis or TEE in the preceding 6 months	Lack of suitability for study due to safety reasons	X	

4.4 Subjects Withdrawal Criteria and Replacements

The participation of an individual subject may be terminated prematurely for reasons such as:

a. Withdrawal of written informed consent
b. Study discontinuation due to subject's own request (e.g. personal reasons)
c. Required treatment with any medication known or suspected to interfere with the IMP
d. Life threatening thrombosis or TEE or life threatening hypersensitivity or any AE, laboratory abnormality, or other medical condition or situation occurs suggesting that continued participation in the study would not be in the best interest of the subject.
e. Protocol deviation requiring discontinuation of study treatment
f. Evidence of exclusion criteria or inclusion criteria not met
g. Lack of study compliance
h. Recommendation of the Data Safety Monitoring Board (DSMB)

A subject is entitled to discontinue participation in the clinical study at their own request at any time without stating a reason.

The investigator can terminate a subject's participation in the study at any time if continuation could lead to disadvantages for the subject which cannot be justified by the investigator.

The reason for withdrawal of the subject must be documented by the investigator together with all data collected until the day of premature study termination including laboratory results and assessment of AE. All examinations foreseen for the subject's last study visit (closing visit) should be performed. Afterwards, the subject will be treated according to local standards at the discretion of the investigator.

In case a subject withdraws due to an AE or SAE please follow the instructions given in section 15 Appendix 2: Reporting Procedures of this protocol.

Withdrawn subjects will not be replaced.

For screening failures occurring during the screening period the following data need to be documented in eCRF only:

- Informed Consent
- Demographic data
- In-/Exclusion criteria
- End of Study (day and reason)
- AE information

For intra-operative screening failures (not randomized subjects) the following data need to be documented in eCRF only:

- Informed Consent
- Demographic data
- In-/Exclusion criteria
- Classification of type of planned spine surgery
- Estimated blood loss
- FIBTEM A10 at Baseline
- Duration of surgery
- End of Study (day and reason)
- AE information

4.5 Subjects Information

The subject will be informed about the clinical study according to the requirements of GCP and the legal requirements of the country in which the subject is recruited.

The clinical study, its objectives, possible benefits and risks, and its consequences will be verbally explained to the subject. Moreover, the subject is provided with written information about the clinical study. Sufficient time will be allowed for the information to be read and for questions to be asked. Attention should be paid to signs of undue distress in subjects who are unable to clearly articulate their distress. The subject must be told that refusal to participate in the clinical study does not cause any disadvantages to their treatment; similarly, withdrawal of written informed consent is possible at any time, without stating a reason and without prejudice to further medical management.

Subjects should be informed and should agree that medical data may be reviewed by authorized persons during monitoring and during an audit or an inspection by the

appointed regulatory authority or ethics committee, but that personal data will be treated with absolute confidentiality.

Upon request, the subject must be granted access to the insurance terms and conditions.

Any new and relevant information that evolves during the course of the clinical study concerning the IMP, alternative treatments, or the benefit/risk ratio will be communicated to the subject.

4.6 Declaration of Informed Consent

The subject must have given written consent to participate in the clinical study by signing and personally dating the Informed Consent Form (ICF). Informed consent to the proposed data handling and to data inspection must also be documented in written form. Written informed consent must be obtained from each subject before any study-related procedures are performed. The subject's written informed consent will be filed at the investigator's site.

A duplicate of the signed and dated written ICF must be handed over to the subject.

5 INVESTIGATIONAL MEDICINAL PRODUCTS

5.1 Investigational Medicinal Product BT524

BT524 is a lyophilized, heat-treated, virus and prion safe human fibrinogen concentrate manufactured from human plasma. Fibrinogen conversion to fibrin strands during blood clot formation is one of the major steps in the coagulation cascade to stop bleeding. In subjects with fibrinogen deficiencies, therapeutic substitution with human fibrinogen concentrate will help correct the hemostatic defect and arrest or prevent bleeding.

The manufacturing process of BT524 contains [redacted] steps that were shown to be effective for removal/inactivation of enveloped viruses such as HIV, HBV and HCV, and for the non-enveloped viruses such as Reo, HAV and parvovirus B19. Moreover, [redacted] re effectively removed during the production process.

Thus, BT524 is a virus and prion safe plasma-derived product fulfilling the requirements of the national German and European (CHMP) guidelines on a virus and prion safe pharmaceutical product.

BT524 is presented as a single-use vial with a nominal content of 1 g fibrinogen (lyophilized powder for solution for injection/infusion) to be reconstituted under aseptic conditions with 50 mL of water for injections, resulting in a final concentration of 20 mg/mL for infusion.

5.1.1 Description of Investigational Medicinal Product BT524

Substance code:	BT524
Active ingredients:	Fibrinogen concentrate from human plasma
Composition:	Lyophilized powder for solution for injection/infusion
Dosage form:	1 g
Concentration:	20 mg/mL after reconstitution with 50 mL water for injections
Container:	100 mL glass vial with rubber stopper
Manufacturer:	Biotest AG, D-63303 Dreieich, Germany

Batch number and expiry date are given in the applicable certificates of analysis.

5.1.2 Formulation, Packaging and Labelling

BT524 is a lyophilized, heat-treated fibrinogen concentrate manufactured from human plasma according to the description of Ph. Eur. monograph 0024 on Human Fibrinogen.

BT524 drug product is presented as a single-use 100 mL Type I glass vial with a nominal content of 1 g fibrinogen (lyophilized powder for solution for injection/infusion).

The labelling of BT524 will be performed according to local requirements. A sample label will be filed in the Trial Master File (TMF).

Batch number and vial number must be documented in the eCRF and the drug accountability log.

5.1.3 Storage Conditions and Stability

BT524 is to be stored in a cabinet or other enclosure which is security locked. Generally access should be restricted to the investigator and authorized personnel.

BT524 is to be stored at a temperature [redacted]. Continuous temperature recording should be documented on a temperature log.

5.1.4 Preparation for Use

BT524 is presented as a single-use glass vial of 100 mL with a nominal content of 1 g of lyophilisate (powder for solution for injection/ infusion). The lyophilisate is to be reconstituted under aseptic conditions with 50 mL of water for injections using an appropriate transfer device or syringe.

The vial is to be swirled gently until the product is fully dissolved. After reconstitution, the solution should be almost colorless and clear to slightly opalescent. Reconstituted products should be inspected visually for particulates and discoloration prior to administration. Do not use solutions that are cloudy or contain deposits.

BT524 will be delivered to the operating room by the pharmacy and/or according to local practice.

5.2 **Investigational Medicinal Product Fresh Frozen Plasma (FFP)**

<u>FFP will be used as active comparator to BT524 in subjects undergoing major spinal surgery.</u>

FFP is the standard of care in many European countries for replacement of coagulation factors during major bleeding in clinical settings such as surgery and trauma.

FFP is the liquid portion of blood that contains all the clotting factors, fibrinogen (400 to 900 mg/unit), plasma proteins (particularly albumin), electrolytes, physiological anticoagulants (protein C, protein S, antithrombin, tissue factor pathway inhibitor) and added anticoagulants, and that is stored by freezing (Nascimento et al., 2010). Standard FFP contains 2-5 mg fibrinogen per mL (American Society of Anesthesiologists Task Force on Perioperative Blood and Adjuvant, 2006; Stainsby et al., 2006). FFP is usually authorized nationally. FFP will be provided and used by the site according to local standards.

5.2.1 Description of Investigational Medicinal Product FFP

Substance code:	FFP
Active ingredients:	Human plasma proteins (including all coagulation factors)
Dosage form:	AB0-blood group specific solution for infusion, which appears (slightly) yellow
Container:	Blood bags containing approximately 200-250 mL frozen solution

5.2.2 Formulation, Packaging and Labelling

The labelling of FFP will be performed according to local requirements.

Bag number must be documented in the eCRF and the drug accountability log.

5.2.3 Storage Conditions and Stability

In general, FFP will be stored protected from light, at a temperature at ≤ -18°C. Continuous temperature recording should be documented on a temperature log.

5.2.4 Preparation for Use

FFP is to be thawed according to local standards. FFP should be inspected visually for particulate matter and discoloration prior to administration, whenever solution and container permit. Do not use if turbid. Avoid shaking.

5.3 Investigational Medicinal Product Cryoprecipitate

Cryoprecipitate will be used as active comparator to BT524 in subjects undergoing cytoreductive PMP surgery at one site in the UK.

6 STUDY TREATMENT

6.1 Dosage Regimen

After a quantitative bleeding assessment only subjects with an intra-operative clinically relevant bleeding of approximately 1 L, requiring hemostatic treatment (high risk for the need of fibrinogen supplementation with BT524 or FFP) during surgery, will be treated with BT524 or FFP according to the predefined treatment algorithm.

6.2 Dosage and Administration

In general, the dosage of IMP (BT524 or FFP) depends on the extent of bleeding and the subject's clinical condition. Therefore, the functional fibrinogen level should be determined intra-operatively on an individual subject basis by FIBTEM thromboelastometry, and the target level of fibrinogen FIBTEM A10 is defined as the baseline FIBTEM A10 measured prior anaesthesia.

Blinding

Study 995 will be partially blinded; surgeon, surgical staff and subjects will be blinded to treatment allocation throughout the entire surgery. The anaesthesiologist who will administer the IMP could not be blinded to treatment allocation because of the inherent characteristics of the IMPs BT524 and FFP. Whereas BT524 is administered by syringe with a small volume and fast infusion to rapidly supplement missing fibrinogen to restore haemostasis, FFP is administered as infusion bag with larger volume and longer infusion time. Due to the different methods of application, the different volume, resulting in different infusion times of the two IMPs a blinding technique at the level of the anaesthesiologist is considered not feasible (either impossible or heavily impractical) to allow the study to be conducted successfully.

The basis for the partial blinding in the operating room is the spatial separation between the anaesthetic field and the surgical, sterile field (operating field). As a basic principle, the anaesthesiologist remains outside of the operating field. By using a sterile drape with non-transparent material between these two fields, the surgeon will not be able to see which IMP is being administered by the anaesthesiologist. The sterile drape will especially cover up the infusion stands and therefore the administration of FFP. The anaesthesiologist has a professional and organizational responsibility regarding the maintenance of blinding of the surgeon and the surgical staff during the entire surgery. In general, the anaesthesiologist is responsible for administering all medications including IMP, and for monitoring and maintenance of vital functions (including e.g. heart rate, oxygen saturation) and laboratory values of the subject.

BT524

The dose of BT524 to be infused will be calculated based on the subject's BW and the measured FIBTEM A10 with the aim of restoring the individual baseline FIBTEM A10 values via the following formula:

BT524 dose (g) = (baseline FIBTEM A10 - actual FIBTEM A10) x BW/140

The calculated dose will be rounded to the nearest whole number of grams of fibrinogen. The first BT524 dose to be administered will be at least 2 g and the maximum dose of fibrinogen concentrate during surgery should not exceed 8 g. In case of repeated dosing,

regular monitoring of the plasma level of fibrinogen (FIBTEM A10) during therapy is indicated.

BT524 will be administered as IV infusion with a maximum infusion rate of [redacted]/min (1 g fibrinogen concentrate [redacted]). BT524 is to be administered preferably in the forearm vein. Alternatively, BT524 can be administered through a central venous line or a peripherally inserted central catheter (PICC). Other administration routes are only allowed after approval from the sponsor.

Fresh Frozen Plasma

In general FFP contains between 2 and 5 mg/mL fibrinogen. The usual FFP dose in major bleeding is 10 to 15 mL/kg . This should increase the subject's plasma coagulation factor levels by approximately 15-25%.

In general, the fibrinogen replacement using FFP should be guided by clinical situation and coagulation results. The volume of FFP to be transfused depends on the subject's BW and the recommended adult therapeutic dose of FFP is 15 mL per kg BW:

Subsequent intra-operative infusions as required.

Calculations for One Adult Therapeutic Dose FFP

Patient Weight (kg)	FFP dose – Volume/Units†	
	15mL/kg	Units FFP
50kg	750mL	
55kg	825mL	3
60kg	900mL	
65kg	975mL	
70kg	1,050mL	4
75kg	1,125mL	
80kg	1,200mL	
85kg	1,275mL	
90kg	1,350mL	5
95kg	1,425mL	
100kg	1,500mL	

†Volume of FFP in a unit is variable, mean FFP unit volume = 273mLs(3).

Source: (NHS Blood and Transplant, November 2013. Access Date: 29-Jun-2016.)

FFP will be administered after thawing using an infusion set with a filter. FFP will be administered as IV infusion as rapidly as possible and at the discretion of the treating anaesthesiologist.

6.3 Compliance with Dosage Regimens

As the IMP (BT524 or FFP) will be administered IV to each subject under the supervision of the unblinded anaesthesiologist, the compliance is expected to be 100%. In addition, the assessment of plasma fibrinogen concentrations may also serve as an adherence measure.

If a subject's treatment deviates from the dosage regimen (e.g., a dosing interruption occurs due to the occurrence of an AE), this will be recorded in the eCRF.

6.4 Dose Justification

In general, the dosage of IMP (BT524 or FFP) depends on the clinical situation and the extent of bleeding during surgery.

The recommended therapeutic dose of FFP is a guide to the appropriate adult dose, it is not a directive, and should not be used in place of clinical assessment (NHS Blood and Transplant, November 2013. Access Date: 29-Jun-2016.).

Fibrinogen is the first coagulation factor to become critically reduced during major surgical blood loss and the observation that patients with higher fibrinogen levels experience fewer bleeding complications than those with low levels highlights the importance of fibrinogen in the maintenance of hemostasis (Charbit et al., 2007; Ucar et al., 2007). Consequently, fibrinogen replacement therapy targeting a high-normal level of plasma fibrinogen and fibrin-based clot formation may be an important first step in restoring hemostasis during major bleeding (Rahe-Meyer et al., 2013a).

The normal blood fibrinogen concentration is between 2.0 and 4.5 g/L although this range can vary (Levy and Goodnough, 2015). Restoring the individual physiological fibrinogen level seems reasonable to ensure sufficient fibrinogen supplementation without risk of overdosing.

Therefore, the repeated IMP dosing in this study will be guided by determination of the functional fibrinogen level via ROTEM/FIBTEM thromboelastometry intra-operatively with the aim to restore patient's specific baseline fibrinogen level obtained prior to start of surgery.

Instead of using a fixed fibrinogen target level for all patients, the patient specific baseline level prior to start of surgery was chosen to take the individual physiological fibrinogen level of each patient into account. The patient's individual baseline fibrinogen level is defined as therapeutic target level to assure that the patients are treated with fibrinogen supplementation according to their specific clinical situation and their individual needs.

Rotational thromboelastometry (ROTEM) is an established viscoelastic method for hemostasis testing in whole blood, and ROTEM/FIBTEM that measures the fibrin or fibrinogen contribution to clot strength can be used to determine the most appropriate therapeutic dose of fibrinogen concentrate (Levy et al., 2014).

The ROTEM/FIBTEM test will be used in the intra-operative setting to quickly identify deficits in fibrin quality, and to guide hemostatic therapy. In contrast to conventional laboratory tests (measurement of fibrinogen concentration via Clauss assay), ROTEM/FIBTEM can measure early variables describing the clot firmness, such as clot amplitude obtained after 10 minutes (A10), and provide a forecast on the expected Maximum Clot Firmness (MCF) value at an earlier stage already. This early variable allows for a more rapid decision about therapeutic interventions. Owing to its rapid assessment of fibrinogen, ROTEM is frequently used to guide transfusion therapy (Schochl et al., 2011), and the data from Schochl and colleagues revealed the effective

use of ROTEM-guided coagulation management in trauma patients by reducing the amount of allogeneic blood product transfusion (Schochl et al., 2010).

Accordingly, the IMP treatment in this clinical study will be based on ROTEM/FIBTEM to obtain results more quickly and the dosing is targeted on the correction of the plasma fibrinogen level to the patient's individual fibrinogen FIBTEM A10 baseline level prior surgery.

According to the investigators' feedback, there is a certain amount of time between ‘decision to treat’ and start of IMP treatment. The FIBTEM value determined at decision to treat applies to the dose calculation of BT524. If treatment starts only about 30 to 45 minutes later after measuring the FIBTEM, there is a great risk of under-dosing the patients. In the time-window between decision to treat (measuring FIBTEM) and start of IMP treatment, patients' blood loss continues and fibrinogen levels continue to decline. The BT524 dose required at start of IMP treatment to restore fibrinogen plasma level is likely to be higher than the dose calculated at the time of treatment decision. Therefore, the first BT524 dose administered should be a dose of at least 2 g. This is in line with the dose recommendation given in the core SmPC for human fibrinogen concentrate (EMA, 2015b).

The FIBTEM guided dosing is only possible for the human fibrinogen concentrate BT524 with a defined concentration of fibrinogen. The content of fibrinogen in FFP varies between 2 and 5 mg/L. Therefore, the fibrinogen replacement using FFP will be performed according to treatment guidelines and local standards. The volume of FFP to be transfused depends on the patient's BW and the recommended adult therapeutic dose of FFP is 15 mL per kg BW (NHS Blood and Transplant, November 2013. Access Date: 29-Jun-2016.).

6.5 Treatment of Overdose

In order to avoid overdosage in subjects with acquired hypofibrinogenaemia undergoing major surgery, point-of-care FIBTEM guided dosing according to fibrinogen plasma levels will be used for the fibrinogen concentrate BT524 during surgery. The fibrinogen content in FFP is much lower and the amount of FFP will be applied based on BW according to local standards. Monitoring of the plasma level of fibrinogen intra-operatively will be performed as defined in section III.

In case of overdosage, the risk of development of thromboembolic complications is enhanced (EMA, 2015a).

6.6 Randomization Code

There will be a stratified randomization per surgery type. Subjects undergoing spine surgery are to be randomized on a 1:1 basis to receive either BT524 or FFP, and subjects undergoing cytoreductive PMP surgery are to be randomized separately on a 1:1 basis to receive either BT524 or cryoprecipitate.

For subjects undergoing spine surgery there will be a stratified randomization according to the expected blood loss: > 1,000 mL to ≤ 2,000 mL and > 2,000 mL. The expected blood loss will be recorded prior surgery.

Randomization of subjects to treatment will occur intra-operatively (predose) when eligibility for the clinical study has been confirmed. After an intra-operative blood loss of approximately 1 L, requiring hemostatic treatment (high risk for the need of fibrinogen supplementation with BT524 or FFP) during surgery, the randomization request will be

sent to the pharmacy (if applicable). The pharmacy retrieves the randomization code via Interactive Web Response System (IWRS) and provides the prepared IMP to the unblinded anaesthesiologist. In case the anaesthesiologist retrieves the randomization code via IWRS, the pharmacy will be informed accordingly and can provide the IMP.

Subject Identification

For the coherent assignment of the study documents all subjects having signed the informed consent and having entered the screening period will receive a subject number. The subject number comprises a five digit number of which the first two digits define the investigational site and the last three digits the subject enrolled at the corresponding site. Subject numbers are assigned consecutively per site. Subject numbers are assigned unique and will not be replaced i.e., in case of a screening failure.

An interactive web/voice response system (IWRS/IVRS) will be implemented and used for randomization and re-supply. Detailed instructions for the use of IWRS systems are provided in a separate document that will be filed in the Investigator Site File.

The random allocation of treatments to subjects will be done using a computerized randomization program. Subjects will receive a randomization number, which will be recorded along with the date of randomization in the eCRF.

6.7 Procedures for Emergency Unblinding

In the event of an emergency, each study site will be able to unblind subject treatment allocation via IWRS (either the principal investigator or a designated medic sub-investigator at the study site) without undue delay.

However, unblinding shall only be carried out if a medical emergency requires the identification of the IMP for that particular participant. If possible and time allows, investigators should make every effort to discuss the subject's case with the sponsor or representative prior to breaking the blind. If the code is broken for a subject (via the IWRS), Biotest must be informed immediately. The reason for opening the code break must be documented on the appropriate eCRF page along with the date and the initials of the person who broke the code.

Any subject for whom the blind is broken will be discontinued from the clinical study, however will be followed-up.

6.8 Drug Accountability

The IMP BT524 will be supplied to the investigator at the time of site initiation under the assumption that all required regulatory documents are in place. The investigator or his/her designee should maintain records that document adequately that the subjects were provided the doses specified in the protocol and reconcile all IMPs received for the clinical study. The investigator has to ensure that consignments of IMP are received correctly by a dedicated person (e.g. pharmacy) and that the IMP is safely and appropriately handled and stored.

The investigator or designee is obliged to keep sufficient documentation of the delivery, use, and destruction or return of unused, used or partially used packages of IMP. The investigator must allow the monitor to perform drug reconciliation before any IMP is returned or destroyed. The documentation must include dates, quantities, subject numbers, batch numbers and expiry dates.

The entries in the eCRF as well as the documentation kept in the Investigator Site File will be compared with the returned and residual IMPs, with clarification of any discrepancies or inconsistencies.

6.9 Previous and Concomitant Medication or Treatment

All previous medication and treatment in the previous 4 weeks prior to the elctive surgery and the administration of IMP are to be recorded in the eCRF.

Concomitant medication therapeutically required is allowed during the study. If a change in concomitant medication is necessary during the study, it is the responsibility of the investigator to ensure that details regarding the medication are recorded in full in the eCRF (i.e., identity of all medications, dosage and route of administration, frequency, duration of administration, and indication for use at each visit).

6.10 Prohibited Medication or Treatment

The administration of allogeneic or autologous blood products interacting relevantly with the coagulation system (e.g. platelet concentrates, cryoprecipitate) and of hemostatic agents (including coagulation factor concentrates) is not allowed prior to start of or during the first IMP administration within this study. In such instances, the subject can not be randomized or will be withdrawn from the study.

RBCs can be infused according to institutional practice.

6.11 Warnings and Precautions

Brief Summary

A Guideline for Core SmPC for Human Fibrinogen Products (EU Core SmPC) is in place which also describes the well established benefit-risk profile of the authorized fibrinogen concentrates [Guideline on Core SmPC for Human Fibrinogen Products (EMA, 2015a)].

It provides the following AEs as undesirable effects considered established for all authorized and marketed human fibrinogen concentrate formulations:

In the MedDRA System Organ Class (SOC) "Immune system disorders": Allergic or anaphylactic-type reactions; in the MedDRA SOC "Vascular disorders": Thromboembolic episodes (including myocardial infarction and pulmonary embolism); and in the MedDRA SOC "General disorders and administration site conditions": Increase in body temperature.

Description of Potential Risks

There is a risk of thrombosis when subjects, with either congenital or acquired deficiency, are treated with human fibrinogen particularly with high dose or repeated dosing. Subjects given human fibrinogen should be observed closely for signs or symptoms of thrombosis. In subjects with a history of coronary heart disease or myocardial infarction, in subjects with liver disease, in peri- or post-operative subjects, in neonates, or in subjects at risk of TEEs or DIC, the potential benefit of treatment with human plasma fibrinogen should be weighed against the risk of thromboembolic complications. Caution and close monitoring should also be performed.

Acquired hypofibrinogenaemia is associated with low plasma concentrations of all coagulation factors (not only fibrinogen) and inhibitors and so treatment with blood products containing coagulation factors should be considered. Careful monitoring of the coagulation system is necessary.

If allergic or anaphylactic-type reactions occur, the injection/infusion should be stopped immediately. In case of anaphylactic shock, standard medical treatment for shock should be implemented.

There is currently no data on fibrinogen inhibitors available with human fibrinogen.

Standard measures to prevent infections resulting from the use of medicinal products prepared from human blood or plasma include selection of donors, screening of individual donations and plasma pools for specific markers of infection and the inclusion of effective manufacturing steps for the inactivation/removal of viruses. Despite this, when medicinal products prepared from human blood or plasma are administered, the possibility of transmitting infective agents cannot be totally excluded. This also applies to unknown or emerging viruses and other pathogens.

The measures taken are considered effective for enveloped viruses such as human immunodeficiency virus (HIV), hepatitis B virus (HBV) and hepatitis C virus (HCV) and for the non-enveloped hepatitis A and parvovirus B19 viruses. It is strongly recommended that every time that fibrinogen is administered to a patient, the name and batch number of the product are recorded in order to maintain a link between the patient and the batch of the product.

Special precautions for BT524 administration

All plasma-derived human products may lead to allergic or anaphylactic reactions. Thus, the administration of BT524 will be performed under medical supervision where proper medical care for allergic or anaphylactic reactions can be provided. If allergic or anaphylactic-type reactions occur, the infusion must be stopped immediately. In case of anaphylactic shock, standard medical treatment for shock must be applied.

Prothrombin time PT(INR), activated partial thromboplastin time (aPTT), prothrombin fragments F_{1+2}, thrombin-antithrombin III complex (TAT), D-dimer, PS, PC, antithrombin III (AT III) activity and thrombin time (TT) and further coagulation parameters are monitored regularly to detect a hypercoagulable state.

Changes in vital signs (including pulse [heart rate], blood pressure, respiratory rate, body temperature) are monitored during the surgery and according to the flow chart (see section III).

<u>Incompatibilities</u>

Fibrinogen must not be mixed with other medicinal products and should be administered by a separate injection/infusion line.

7 COURSE OF THE CLINICAL STUDY

7.1 Visit Schedule

Screening Visit Day -42 through Day -1:

All subjects will attend a screening visit (if required more than one visit) between Day -42 and Day -1 prior to elective major spine surgery, where the following procedures will be performed:

- **Written informed consent** will be obtained.
- In general, eligibility to take part in the study will be assessed against the **inclusion and exclusion criteria**.
- **Demographic data** (including sex, year of birth, race) will be recorded.
- Classification of **type of the planned spine surgery** will be recorded.
- **Physical examination** (including body weight and body height) will be performed.
- **Body weight** will be recorded.
- A serum **pregnancy test** (human chorionic gonadotropin) will be performed for all females of child-bearing potential (a woman of child bearing potential is one that has NOT had a hysterectomy and/or a bilateral oophorectomy, or has NOT been naturally postmenopausal for at least 24 consecutive months).
- **Medical and surgical history** with regard to the subjects' drug history, previous medication and treatment, disease history, and other medical and surgical history will be recorded. Previous medication taken up to 4 weeks before enrollment will be recorded. The type of previous medication, dose schedule, duration, and the indication the previous medication was given for will be documented.
- **Vital signs** (including pulse [heart rate], blood pressure, respiratory rate, body temperature) will be recorded.
- Samples for **clinical laboratory** parameters (hematology, clinical chemistry, urinalysis) will be taken:
  - **Hematology** (RBC, WBC, platelet count, hemoglobin, hematocrit)
  - **Clinical chemistry** (ALAT, ASAT, γ-GT, AP, total bilirubin, creatinine, creatinine clearance, BUN/Urea, potassium, sodium, calcium, chloride)
  - **Urinalysis** (pH, blood, WBC, protein, glucose, ketone bodies, nitrite, bilirubin, urobilinogen)

  Some of these tests for clinical laboratory parameters may have been performed by the investigator as standard of care prior to the subject signing the Informed Consent Form. If test(s) were done within 42 days before the first scheduled treatment with IMP (BT524 or FFP), the investigator may use the results obtained from standard of care for the purpose of this study.
- Sample for **coagulation activation tests** (PT(INR), aPTT, TAT, F_{1+2}, D-dimer, PS, PC, AT III, TT) will be taken.
- Sample for **plasma activity of fibrinogen** (Clauss assay) will be taken.
- **FIBTEM A10 and Maximum clot firmness (MCF)** (ROTEM) will be performed.
- **Retention samples for viral safety** laboratory parameters will be taken.

- **Samples for virus serology laboratory parameters** (hepatitis B, hepatitis C, HIV) will be taken.
- **AEs** will be documented.

If a subject does not fulfill the eligibility criteria, re-assessment is allowed within the 42 days screening period.

Baseline Visit Day 1 (or Day -2 or Day -1, prior to surgery):

All subjects will attend a baseline visit on Day 1, the day of spine surgery. The baseline visit can be scheduled one or two days prior to spine surgery (Day -2 or Day -1) if required due to local hospital procedures.

The following assessments will be performed **prior to start of surgery, before** the potential IMP administration (BT524 or FFP):

- **Re-check of inclusion and exclusion criteria**: Eligibility to take part in the study will be confirmed against the inclusion and exclusion criteria, as appropriate (i.e., diagnostic tests will be repeated at the investigator's discretion).
- Any **changes in medical and surgical history** since the screening visit will be recorded. The type of previous medication, dose schedule, duration, and the indication the previous medication was given for, will be documented.
- **Expected blood loss** will be recorded.
- **Physical examination** will be performed.
- **Pregnancy test** (urine or serum) will be performed for all females of child-bearing potential (a woman of child bearing potential is one that has NOT had a hysterectomy and/or a bilateral oophorectomy, or has NOT been naturally postmenopausal for at least 24 consecutive months).
- **Body weight** will be recorded to calculate subject dose.
- **Vital signs** (including pulse [heart rate], blood pressure, respiratory rate, body temperature) will be recorded.
- Samples for **clinical laboratory** parameters (hematology, clinical chemistry, urinalysis) will be taken:
  - **Hematology** (RBC, WBC, platelet count, hemoglobin, hematocrit)
  - **Clinical chemistry** (ALAT, ASAT, γ-GT, AP, total bilirubin, creatinine, creatinine clearance, BUN/Urea, potassium, sodium, calcium, chloride)
  - **Urinalysis** (pH, blood, WBC, protein, glucose, ketone bodies, nitrite, bilirubin, urobilinogen)
- Sample for **coagulation activation tests** (PT(INR), aPTT, TAT, F_{1+2}, D-dimer, PS, PC, AT III, TT) will be taken.
- Sample for **coagulation factors** (FII, FV, FVII, FVIII, FIX, FX, FXI, FXIII) will be taken.
- Sample for **plasma activity of fibrinogen** (Clauss assay) will be taken and
- **FIBTEM A10 and MCF** (ROTEM) will be performed.
- **AEs** will be documented.

Please note: *The following tests and assessments have to be done prior to surgery. In case of a short time-period between screening and baseline (≤ 2 days) these tests have only to be repeated based on medical judgment of the investigator. If not repeated, screening results will serve as baseline.*

- Physical examination
- Pregnancy test
- Body weight
- Hematology
- Clinical chemistry
- Urinalysis
- Coagulation activation tests
- Plasma activity of fibrinogen (Clauss assay)
- FIBTEM A10 and MCF (ROTEM)

Surgery Day 1 (pre-dose):

- **Time of start of surgery** will be recorded.
- Continuous **collection and measurement of blood loss** from start of surgery.
- Amount of blood in blood suction unit and surgical cloths and compresses will be estimated.
- **Eligibility** to take part in the study will be assessed against the intra-operative inclusion criteria:

  an intra-operative clinically relevant blood loss of approximately 1 L, requiring hemostatic treatment (high risk for the need of fibrinogen supplementation with BT524 or FFP) during surgery.

In subjects who do not meet the intra-operative inclusion criteria no IMP administration will take place and no further assessments intra-operatively will be performed. These subjects will be considered as screening failures and will be treated with standard of care.

Only for subjects considered to be eligible for IMP administration with BT524 or FFP the following assessments will be performed:

- **Time of decision** to treat the subject with IMP will be recorded.
- **Amount of blood** from blood suction unit and from surgical cloths and compresses after decision to treat the subject with IMP until end of surgery will be measured.
- Sample for plasma **activity of fibrinogen** (Clauss assay) will be taken.
- **FIBTEM A10 and MCF** (ROTEM) will be performed.
- **Calculation** of FIBTEM A10 guided dose for BT524 (first dose should be at least 2 g) or weight-based dose of FFP.
- **Randomization** will be initiated **with order of IMP.**
- Sample for **coagulation activation tests** (PT(INR), aPTT, TAT, F_{1+2}, D-dimer, PS, PC, AT III, TT) will be taken prior start of first IMP administration.

- Sample for **coagulation factors** (FII, FV, FVII, FVIII, FIX, FX, FXI, FXIII) will be taken prior start of first IMP administration.
- Samples for **clinical laboratory parameters** (hematology, clinical chemistry) will be taken prior start of first IMP administration:
    - **Hematology** (RBC, WBC, platelet count, hemoglobin, hematocrit)
    - **Clinical chemistry** (ASAT, ALAT, creatinine, creatinine clearance, BUN/Urea, γ-GT, AP, total bilirubin, potassium, sodium, calcium, chloride)
- **Vital signs** (pulse [heart rate], blood pressure, respiratory rate, body temperature) will be recorded prior start of IMP administration.
- **Transfusion products** (allogenic blood products or autologous blood transfusion/cell salvage) given intra-operatively will be documented.
- **Concomitant medication or treatment** will be recorded. The type of concomitant medication/treatment, dose/schedule, duration, and the indication the concomitant medication was given for will be documented.
- **AEs** will be documented.
- **IMP** will be infused IV.

  The total volume and the total infusion time of each IMP administration (start and end of infusion) will be recorded.

__Surgery Day 1 (post-dose):__

- Continuous **collection and measurement of blood loss**
- **Vital signs** (pulse [heart rate], blood pressure, respiratory rate, body temperature) will be recorded **15 minutes** and **90 minutes** after start of IMP administration.
- Sample for **coagulation activation tests** (PT(INR), aPTT, TAT, F_{1+2}, D-dimer, PS, PC, AT III, TT) will be taken **15 minutes** and **90 minutes** after start of first IMP administration.
- Sample for **coagulation factors** (FII, FV, FVII, FVIII, FIX, FX, FXI, FXIII) will be taken **90 minutes** after start of first IMP administration.
- Sample for plasma **activity of fibrinogen** (Clauss assay) will be taken **15 minutes** and **90 minutes** after start of first IMP administration.
- **FIBTEM A10 and MCF** (ROTEM) will be performed **15 minutes** and **90 minutes** after start of first IMP administration.
- **Transfusion products** (allogenic blood products or autologous blood transfusion/cell salvage) given intra-operatively will be documented.
- **Concomitant medication or treatment** will be recorded. The type of concomitant medication/treatment, dose/schedule, duration, and the indication the concomitant medication/treatment was given for will be documented.
- **AEs** will be documented.

Please note: *The following tests have to be done 90 minutes after start of first IMP treatment. In case this time-point is within a short timeframe (< 30 min) after the 'end of surgery', these tests have only to be repeated based on medical judgment of the investigator.*

- Markers of coagulation (Coagulation activation tests)
- Plasma activity of fibrinogen (Clauss assay)
- FIBTEM A10 and MCF (ROTEM)

Surgery Day 1 (prior to repeated IMP administration):

- **Vital signs** (pulse [heart rate], blood pressure, respiratory rate, body temperature) will be recorded prior start of each IMP administration.
- **FIBTEM A10 and MCF** (ROTEM) will be performed.
- **FIBTEM A10 guided dose for BT524** or weight-based dose of FFP will be calculated.
- **IMP** will be ordered**.**
- **IMP** will be administered. The total volume and the total infusion time of each IMP administration (start and end of infusion) will be recorded.
- **Amount of blood** from blood suction unit and from surgical cloths and compresses will be collected and measured.
- **Transfusion products** (allogenic blood products or autologous blood transfusion/cell salvage) given intra-operatively will be documented.
- **Concomitant medication or treatment** will be recorded.
- **AEs** will be documented.

Surgery Day 1 (end of surgery):

- **Vital signs** (including pulse [heart rate], blood pressure, respiratory rate, body temperature) will be recorded at the end of the surgery.
- Samples for **clinical laboratory parameters** (hematology, clinical chemistry) will be taken at the end of the surgery:
  - **Hematology** (RBC, WBC, platelet count, hemoglobin, hematocrit)
  - **Clinical chemistry** (ASAT, ALAT, creatinine, creatinine clearance, BUN/Urea, γ-GT, AP, total bilirubin, potassium, sodium, calcium, chloride)
- Sample for **coagulation activation tests** (PT(INR), aPTT, TAT, F_{1+2}, D-dimer, PS, PC, AT III, TT) will be taken at the end of the surgery.
- Sample for plasma **activity of fibrinogen** (Clauss assay) will be taken at the end of the surgery.
- **FIBTEM A10 and MCF** (ROTEM) will be performed at the end of the surgery.
- Start continuous **measurement of blood loss** until 24 hours post-operative.
- **Amount of blood** from blood suction unit and from surgical cloths and compresses will be calculated and recorded (blood loss after the decision to treat the subject with IMP until end of surgery).
- **Transfusion products** (allogenic blood products or autologous blood transfusion/cell salvage) given intra-operatively will be documented.

- **Concomitant medication or treatment** will be recorded. The type of concomitant medication/treatment, dose/schedule, duration, and the indication the concomitant medication//treatment was given for will be documented.
- **AEs** will be documented.
- **Time of end of surgery** (time of last suture) will be recorded.
- **Rebleeding episodes** will be recorded.

Please note: *The following tests have to be done at the 'end of surgery'. In case the end of surgery is within a short timeframe (< 30 min) after the blood draw '90 minutes after start of first IMP treatment', these tests have only to be repeated based on medical judgment of the investigator.*

- Markers of coagulation (Coagulation activation tests)
- Plasma activity of fibrinogen (Clauss assay)
- FIBTEM A10 and MCF (ROTEM)

Follow-up Visit Day 2:

All subjects treated with IMP will attend the follow-up visit one day after surgery (Day 2). The following procedures will be performed:

- Continuous measurement of post-operative blood loss and recording until 24 hours after end of surgery.
- **Physical examination** will be performed.
- **Vital signs** (including pulse [heart rate], blood pressure, respiratory rate, body temperature) will be recorded.
- Sample for **coagulation activation tests** (PT(INR), aPTT, TAT, F_{1+2}, D-dimer, PS, PC, AT III, TT) will be taken.
- Samples for **clinical laboratory parameters** (hematology, clinical chemistry, urinalysis) will be taken:
  - **Hematology** (RBC, WBC, platelet count, hemoglobin, hematocrit)
  - **Clinical chemistry** (ASAT, ALAT, creatinine, creatinine clearance, BUN/Urea, γ-GT, AP, total bilirubin, potassium, sodium, calcium, chloride)
  - **Urinalysis** (pH, blood, WBC, protein, glucose, ketone bodies, nitrite, bilirubin, urobilinogen)
- Sample for plasma **activity of fibrinogen** (Clauss assay) will be taken and
- **FIBTEM A10 and MCF** (ROTEM) will be performed.
- **Transfusion products** (allogenic blood products or autologous blood transfusion/cell salvage) will be documented.
- **Concomitant medication or treatment** will be documented. The type of concomitant medication/treatment, dose/schedule, duration, and the indication the concomitant medication/treatment was given for will be documented.
- **AEs** will be documented.
- **Rebleeding episodes** will be recorded.

Follow-up Visits Day 3, Day 5 and Day 8:

All subjects treated with IMP will attend the follow-up visits 2, 4 and 7 days after surgery (Day 3, 5 and 8):

The following procedures will be performed:

- **Physical examination** will be performed.
- **Vital signs** (including pulse [heart rate], blood pressure, respiratory rate, body temperature) will be recorded.
- Sample for **coagulation activation tests** (PT(INR), aPTT, TAT, F_{1+2}, D-dimer, PS, PC, AT III, TT) will be taken.
- Samples for **clinical laboratory parameters** (hematology, clinical chemistry, urinalysis) will be taken:
    - **Hematology** (RBC, WBC, platelet count, hemoglobin, hematocrit)
    - **Clinical chemistry** (ASAT, ALAT, creatinine, creatinine clearance, BUN/Urea, γ-GT, AP, total bilirubin, potassium, sodium, calcium, chloride)
    - **Urinalysis** (pH, blood, WBC, protein, glucose, ketone bodies, nitrite, bilirubin, urobilinogen)
- **Transfusion products** (allogenic blood products or autologous blood transfusion/cell salvage) will be documented.
- **Concomitant medication or treatment** will be documented. The type of concomitant medication/treatment, dose/schedule, duration, and the indication the concomitant medication/treatment was given for will be documented.
- **AEs** will be documented.
- **Rebleeding episodes** will be recorded.

Discharge from Hospital:

- If the discharge from hospital will take place on **Day 6** or **Day 7**, the follow-up visit on **Day 8** will be performed prior to the scheduled visit on the day of discharge.
- The day of the discharge from hospital will be recorded at the **closing visit D36**.

Closing Visit Day 36 (+35, up to Day 71 if required):

All subjects treated with IMP will attend a closing visit including the final safety examination at least 5 weeks after the surgery, scheduled on Day 36 (+35, up to Day 71 if required). The following procedures will be performed:

- A **physical examination** will be performed.
- **Vital signs** (including pulse [heart rate], blood pressure, respiratory rate, body temperature) will be recorded.
- Sample for **coagulation activation tests** (PT(INR), aPTT, TAT, F_{1+2}, D-dimer, PS, PC, AT III, TT) will be taken.
- Samples for **clinical laboratory parameters** (hematology, clinical chemistry, urinalysis) will be taken:
    - **Hematology** (RBC, WBC, platelet count, hemoglobin, hematocrit)
    - **Clinical chemistry** (ASAT, ALAT, creatinine, creatinine clearance, BUN/Urea, γ-GT, AP, total bilirubin, potassium, sodium, calcium, chloride)

- **Urinalysis** (pH, blood, WBC, protein, glucose, ketone bodies, nitrite, bilirubin, urobilinogen)

- **Retention samples for viral safety laboratory parameters** will be taken.
- **Samples for virus serology laboratory parameters** (hepatitis B, hepatitis C, HIV) will be taken.
- **Transfusion products** (allogenic blood products or autologous blood transfusion/cell salvage) will be documented.
- **Concomitant medication or treatment**: type of concomitant medication/treatment, dose/schedule, duration, and the indication the concomitant medication/treatment was given for will be documented continuously up to Day 36.
- **AEs** will be recorded continuously up to Day 36.

Further details on the visit schedule that will be used for the assessment of the efficacy and safety parameters in this study are presented in section III, Flowchart of Study.

7.2 Duration of the Clinical Study

First Subject in (planned)	Q1 2018
Last Subject Last Visit *(planned)*	tbd

Individual Subject

Regular duration of individual study participation for eligible screened subjects is at least 5 weeks plus time between screening (signed Informed Consent) and surgery.

Each subject fulfilling the intra-operative inclusion criteria will be administered BT524 or FFP/cryoprecipitate during surgery. The usual stay in hospital after elective surgery varies depending on the type of surgery and subjects overall conditions. Each subject will have follow-up visits 1, 2, 4 and 7 days after surgery (Day 2, 3, 5 and 8) and the day of discharge from hospital will be recorded. Subsequent, each subject has to return into the hospital for the closing visit including the final safety examination at least 5 weeks after surgery, scheduled on Day 36. The closing visit can be postponed up to Day 71 (Day 36 +35) if required due to subject's availability (e.g. stay in rehabilitation center).

Subjects not meeting inclusion criteria or meeting exclusion criteria are screen failures and will end study participation at the day of screen failure. Subjects that do not meet the intra-operatively inclusion criterion are also considered as screen failure and will end study participation on the day of the surgery.

A subject is considered to have completed the study when he/she is presumed to have followed the protocol (i.e., completed visits approximately 5 weeks after surgery). If for any reason, a subject discontinues involvement in the study early, every effort should be made to ensure the subject attends a closing visit (section 4.4).

For each subject date and reason for the end of the study participation will be recorded.

7.2.1 End of Study

The end of clinical study will be defined as the Last Visit of the Last Subject.

7.3 Criteria for Premature Termination

7.3.1 Premature Termination of the Entire Clinical Study

The clinical study as a whole may be stopped by Biotest after consultation with the Coordinating Investigator if there are reasons for which continuation of clinical study is no longer justified, such as:

a) Unacceptable delay of study completion
b) Low recruitment rate
c) A large number of subjects with premature termination
d) Changed benefit-risk ratio according to the efficacy and/or safety results from this or parallel studies
e) Lack of efficacy
f) Recently emerged information suggest that the study population can be offered a more advantageous study design

In case of premature termination of the entire clinical study, the sponsor has to notify the appropriate Ethics Committees and Regulatory Authorities as soon as possible but at the latest within 15 days.

7.3.2 Premature Termination of an Individual Study Site

The clinical study may be stopped at an individual study site for reasons such as:

a) Determination of unexpected, significant, or unacceptable risk to subjects.
b) Low recruitment rate,
c) Lack of co-operation,
d) Severe deviations from study protocol,
e) Manipulation of study data,
f) Violation of other ethical or legal principles.

7.4 Treatment and Care after the End of the Study

For subjects who have finished the clinical study and for all subjects who drop out prematurely it is the responsibility of the investigator to choose adequate therapeutic measurements.

After termination of the clinical study, any unexpected safety issue that changes the benefit-risk evaluation and is likely to have an impact on the subjects who have participated in it, should be reported as soon as possible to the sponsor. Sponsor expeditiously reports the event to the competent authority(ies) concerned (See also section 9.3.4).

8 BENEFIT-RISK EVALUATION

8.1 Benefit of BT524

Fibrinogen plays a critical role in achieving and maintaining hemostasis in patients undergoing major surgeries and the observation that patients with higher fibrinogen levels experience fewer bleeding complications than those with low levels highlights the importance of fibrinogen in the maintenance of hemostasis. Consequently, fibrinogen replacement therapy targeting a high-normal level of plasma fibrinogen and fibrin-based clot formation may be an important first step in restoring hemostasis during major bleeding.

Major spinal and abdominal surgeries represent elective surgeries often associated with significant intra- and post-operative blood loss resulting in decreased fibrinogen levels. An accurate and rapid determination of (functional) fibrinogen level is important during hemorrhage to establish a timely hemostatic intervention. Individualized dosing of fibrinogen concentrate using a target ROTEM/FIBTEM value integrates rapid diagnostic testing with appropriate therapeutic dosing of fibrinogen concentrate according to patients' needs.

The principal benefit for the participating subjects randomized to the BT524 treatment arm will be to receive the required individualized fibrinogen replacement therapy in a goal-directed treatment algorithm as a timely hemostatic intervention if significant bleeding is accompanied by acquired hypofibrinogenaemia. Moreover, fibrinogen concentrate seems to have certain advantages over the standard replacement therapy with FFP/cryoprecipitate, such as precisely determined, high amount of purified fibrinogen dissolved in a small volume, low risk of pathogen transmission and instant administration without need for thawing or testing AB0 blood group compatibility.

8.2 Foreseeable Risk and Discomfort Related to BT524

At that point of time the pre-clinical and clinical study data are still considered too scarce to finally define risks as "identified" in the context of BT524. Therefore at present all signals as derived from class-labelling, currently remain to be regarded as potential risks. Please also refer to section 6.11 Warnings and Precautions.

8.3 Other Sources of Possible Risk and Discomfort

Please refer to section 6.11 Warnings and Precautions.

8.4 Summary of Possible Risk and Discomfort

Human fibrinogen concentrate is a well-known substance, established for decades in the treatment of congenital fibrinogen deficiency as well as of various indications of acquired fibrinogen deficiency. Fibrinogen concentrates have shown to be safe and well-tolerated (Fenger-Eriksen et al., 2009; Henselmans et al., 1999).

A Guideline for Core SmPC for Human Fibrinogen Products (EU Core SmPC) is in place which also describes the well established benefit-risk profile of the authorized fibrinogen concentrates (EMA, 2015a).

It provides the following AEs as undesirable effects considered established for all authorized and marketed human fibrinogen concentrate formulations: In the MedDRA SOC "Immune system disorders": Allergic or anaphylactic-type reactions; in the MedDRA SOC "Vascular disorders": Thromboembolic episodes (including myocardial infarction

and pulmonary embolism); and in the MedDRA SOC "General disorders and administration site conditions": Increase in body temperature.

Currently, there is no data with human fibrinogen products in regard to inhibitor formation.

With regard to transmissible agents refer to section 6.11 Warnings and Precautions.

The safety profile of BT524 is anticipated to be in line with the marketed fibrinogen concentrates and the content of the EU Core SmPC [Guideline on Core SmPC for Human Fibrinogen Products (EMA/CHMP/BPWP/691754/2013 Rev 1)].

The safety and tolerability and the benefit-risk profile of BT524 (fibrinogen concentrate from human plasma) is considered favorable.

9 ASSESSMENT OF OBJECTIVES / CRITERIA FOR EVALUATION

9.1 Efficacy

9.1.1 Specification of Efficacy Parameters

9.1.1.1 Primary Efficacy Parameter

The primary efficacy parameter is determined from the intra-operative blood loss after the decision to treat the subject with IMP until the end of the surgery as measured by the amount of blood from the blood suction unit and the amount of blood from surgical cloths and compresses.

9.1.1.2 Secondary Efficacy Parameter

Secondary efficacy will be determined using the following parameters:

- Proportion (%) of subjects with successful correction of fibrinogen level (FIBTEM A10) 15 minutes after start of first IMP administration
- Time to first successful correction of fibrinogen level (15 minutes or 90 minutes after start of first IMP administration, end of surgery, not within surgery)
- Total amount (volume in mL and number of units) of transfusion products (allogenic blood products) or autologous blood transfusion infused after start of first IMP administration until end of surgery
- Amount (volume in mL and number of units) of RBCs (allogenic and autologous) infused after start of first IMP administration until end of surgery
- Post-operative blood loss in the first 24 hours
- Proportion (%) of subjects with rebleeds after the end of surgery until Day 8
- Hospital length of stay after surgery
- In-hospital mortality

9.1.2 Methods for Assessing and Recording Efficacy Parameters

9.1.2.1 Methods for Assessing Primary Efficacy Parameter

Amount of Blood Loss

The blood loss will be quantified by measuring the continuous bleeding mass with a **blood suction unit** (and/or a **cell saver**) and by calculation of the amount of blood from **surgical cloths and compresses** (see section 3).

The **blood suction unit** will be used to remove the blood from the area being operated. The blood is salvaged by a suction catheter from the operating field and suctioned into a reservoir which contains a **heparinised saline solution** (or citrate anticoagulant solution) used for anticoagulation during blood collection. The quantity of anticoagulant introduced into the blood collection system will be adapted continuously to the volume of blood loss.

Allowance must be made also for the presence of **irrigation solution**. The amount of solution suctioned into the container through irrigation of the wound must be recorded. This will be done by knowing the capacity of the irrigation syringe in use and keeping track of the number of times it is used.

The total volume of fluid collected in the suction container will be recorded. Then the amount of heparinised saline solution and of irrigation solution must be subtracted **from the entire fluid volume in the suction container to** determine the actual blood lost.

Prior to the start of surgery **dry surgical cloths and compresses** will be weighed (weight recorded), to be available in case a manual compression is required. In case a **manual compression** is necessary during surgery, dry surgical cloths and compresses will be applied to the surgical area, afterwards removed and wrung out until almost dry. By wringing out surgical cloths, the blood can be caught in a kidney dish and finally collected in the suction container. Subsequently, all cloths and compresses will be weighed (before they dry out) and the weight will be recorded.

For the primary endpoint the amount of blood loss will be measured from the time of decision to treat the subject with IMP until the end of the surgery:

Immediately after an estimated blood loss of approximately 1 L, and the assessment that hemostatic treatment will be required during surgery (high risk for the need of fibrinogen supplementation, either with FFP or BT524 during surgery), the pharmacy will be informed about the decision to treat the subject with IMP. At the same time, the **suction container will be emptied and the blood measurement for the primary endpoint is initiated** with collection of blood in the empty blood suction unit.

In addition, in case a manual compression is necessary after decision to treat the subject with IMP, new surgical cloths and compresses will be applied to the surgical area.

At the end of the surgery, all surgical cloths and compresses will be removed from the surgical area, wrung out until almost dry (blood will be collected) and weighed. The weight will be recorded. The end of surgery is defined as time of last suture.

The fluid volume collected in the blood suction container will be measured and documented, the proportion of blood, heparinised saline solution and irrigation solution will be calculated and also recorded.

In a final step the **total blood loss will be calculated**. The calculation of blood loss based on the volume of blood removed from the surgical field by the blood suction unit and that absorbed by surgical cloths and compresses.

9.1.2.2 Methods for Assessing Secondary Efficacy Parameter

Correction of Fibrinogen Level via FIBTEM A10 (mm)

The correction of the fibrinogen level will be measured via thromboelastometry (ROTEM/FIBTEM A10). ROTEM is an established viscoelastic method for hemostasis testing in whole blood and ROTEM/FIBTEM that measures the fibrin or fibrinogen contribution to clot strength, can be used to determine the most appropriate therapeutic dose of fibrinogen concentrate (Levy et al., 2014).

Whole blood viscoelastic tests such as the fibrin-based thromboelastometry (ROTEM) test FIBTEM will be used in the intra-operative setting to quickly identify deficits in fibrin quality, and to guide hemostatic therapy. In contrast to conventional laboratory tests (measurement of fibrinogen concentration via Clauss assay), ROTEM/FIBTEM can measure early variables describing the clot firmness, such as clot amplitude obtained after 5 minutes (A5) or 10 minutes (A10), and provide a forecast on the expected MCF value at an earlier stage already. These early variables allow for a more rapid decision about therapeutic interventions.

The fibrinogen activity will be measured by FIBTEM A10, MCF and Clauss assay at screening, prior surgery (baseline), predose, 15 and 90 minutes after the start of first IMP administration, at the end of the surgery and one day after surgery.

For the evaluation of the secondary efficacy endpoint successful correction of the fibrinogen level is defined as restoring fibrinogen FIBTEM A10 baseline levels measured by ROTEM 15 minutes after start of first IMP administration.

Accordingly, the time to first successful correction of fibrinogen level is defined as the time point (15 minutes or 90 minutes after start of first IMP administration, end of surgery, not within surgery) the fibrinogen FIBTEM A10 baseline levels measured by rotational thromboelastometry are restored the first time.

However, correction of fibrinogen will also be analysed based on results obtained from MCF and Clauss assay to perform sensitivity analyses.

Clauss Assay

The most widely used technique for determination of fibrinogen concentration is the Clauss (FIBClauss) assay (Clauss, 1957). In this method, dilutions of a plasma standard of known fibrinogen concentration are clotted by addition of a high concentration of thrombin, and a standard curve is prepared. Because the clotting time is inversely proportional to the fibrinogen concentration, the clotting time of diluted patient plasma is used to read the fibrinogen concentration from the standard curve. This method is reliable, accurate, and precise, and easily adapted to automated coagulation analyzers.

Maximum Clot Firmness (MCF, mm)

MCF addresses the clot integrity and will be measured by ROTEM® (ROTEM delta or ROTEM sigma).

MCF will be determined locally from whole blood or at the central laboratory by means of the Fib-tem S assay (tissue factor activation and platelet inhibition), a ready-to-use ROTEM system reagent (PPD , Mar 2011) for the ROTEM delta/sigma which allows the assessment of the fibrinogen level and the quality of the fibrin polymerization in citrated blood by inhibiting the platelets. For ROTEM sigma the FIBTEM C system reagent will be used accordingly.

Fibtem measures the viscoelastic properties of the clot and provides information on the speed of coagulation initiation, kinetics of clot growth (MCF, mm), clot strength, and breakdown(Lang et al., 2009). **Figure 4** shows an example of the ROTEM readout of citrated normal blood.

Figure 4: ROTEM Readout of Citrated Normal Blood

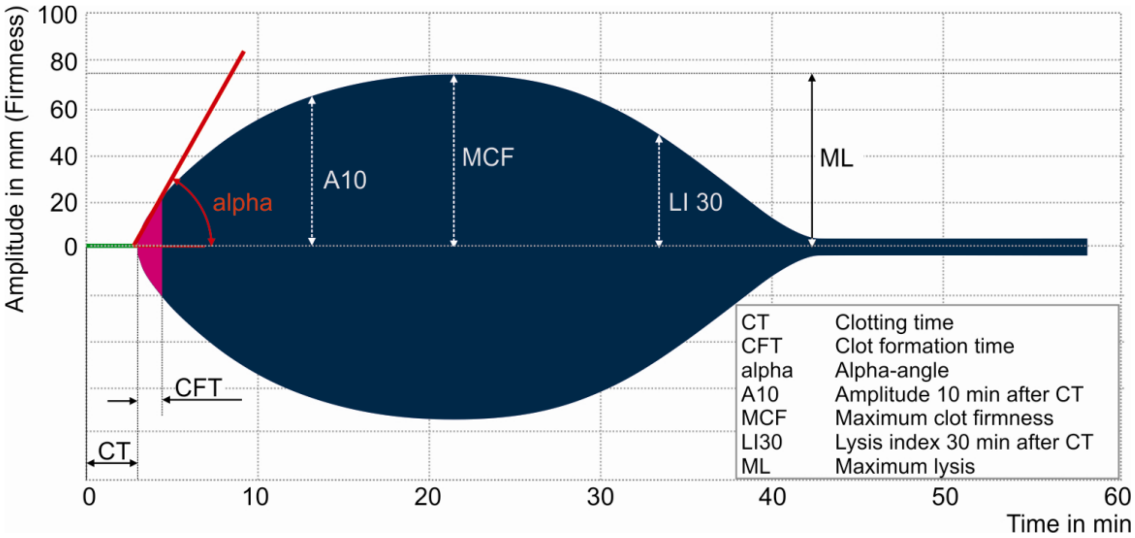

The ROTEM® analyses: FibTEM® test (fibrin clot obtained by platelet inhibition with cytochalasin D). The clotting time (CT (seconds)) represents the time from the start of the test until a clot firmness of 2 mm is detected; maximum clot firmness (MCF (mm)) represents the total amplitude of the clot (Schöchl et al., 2010***)***.

In the ROTEM read out of Fib-tem S test expected reference (normal range) values for the amplitude of MCF are 9-25 mm for fibrinogen levels (Fib-tem S 2011).

Consumption of Transfusion Products

The **amount of transfusion products** (allogenic blood products or autologous blood transfusion) given after start of first IMP administration (BT524 or FFP) until end of surgery to counteract hemodynamic instability will be documented (volume in mL and number of units if applicable) and evaluated.

The **amount of RBCs** (allogenic and autologous) required intra-operatively after start of first IMP administration (BT524 or FFP) until end of surgery will be documented (volume in mL and number of units if applicable) and evaluated.

Intra-operative Blood Salvage (Cell Salvage)

Intra-operative blood salvage is the collection and reinfusion of blood lost during and immediately after surgery. The blood from the surgical field is recovered, mixed with an anticoagulant and pumped through a cell salvage machine where it is centrifuged and washed. The resulting RBCs are then pumped into a transfer bag. The RBCs may be reinfused to the patient immediately or at a later time.

Autologous RBCs obtained by blood salvage are usually transfused immediately; in special circumstances they can be stored, for a maximum of 6 hours, at 4±2 °C, but must be identified unequivocally.

Autologous RBCs which are re-transfused to the subject after start of first administration of IMP until end of surgery have to be included in the amount of RBCs to be defined for the secondary efficacy parameter.

Postoperative Drainage Volume

The plasma drainage tube and disposable drainage bag will be emptied 24 hours after the end of the surgery. The end of surgery is defined as time of last suture. The drainage volume will be measured and recorded.

Rebleeds

Any bleed judged by the surgeon and/or the anaesthesiologist as requiring haemostatic treatment (including reoperation) after the end of the surgery until follow-up Day 8 will be recorded. The end of surgery is defined as time of last suture.

Hospital Length of Stay after Surgery

The day of the surgery and the day of the discharge from hospital will be recorded.

The hospital length of stay after surgery is used to compare the number of days patients stayed in the hospital after surgery. The hospital length of stay after surgery is calculated by the following formula:

Length of stay after surgery = date of discharge - date of surgery

In-hospital Mortality

In-hospital mortality is defined as death occurring during the hospital stay. A death during hospitalization will be recorded as an SAE.

9.1.3 Specification of Efficacy Endpoints

9.1.3.1 Specification of Primary Efficacy Endpoint

Intra-operative blood loss as measured by amount of blood from the blood suction unit and amount of blood from surgical cloths and compresses after the decision to treat the subject with IMP until end of surgery. The end of surgery is defined as time of last suture.

9.1.3.2 Specification of Secondary Efficacy Endpoints

- Proportion (%) of subjects with successful correction of fibrinogen level 15 minutes after start of first IMP administration.

Successful correction of fibrinogen level in a subject is defined as restoring fibrinogen FIBTEM A10 baseline level to at least 95% measured by ROTEM 15 minutes after start of first IMP administration. A correction of at least 95% is considered successful, as measurement methods has a coefficient of variation of about 5% (Solomon et al., 2015).

- Time to first successful correction of fibrinogen level (15 minutes or 90 minutes after start of first IMP administration, end of surgery, not within surgery).
- Total amount of transfusion products (allogenic blood products) or autologous blood transfusion infused after start of first IMP administration until end of surgery. The end of surgery is defined as time of last suture.
- Amount of RBCs (allogenic and autologous) infused after start of first IMP administration until end of surgery. The end of surgery is defined as time of last suture.
- Post-operative blood loss in the first 24 hours.
- Proportion (%) of subjects with rebleeds.
  Rebleeds are defined as any bleed requiring haemostatic treatment (including reoperation) after the end of surgery until Day 8.
- Hospital length of stay after surgery, defined as the date of discharge minus the date of surgery.
- In-hospital mortality.

9.2 **Safety**

9.2.1 Specification of Safety Parameters

Safety and tolerability in this clinical study will be addressed by the following safety parameters:

- AEs
- Changes in vital signs (including pulse [heart rate], blood pressure, respiratory rate, body temperature)
- Change in clinical laboratory assessments of hematology, clinical chemistry and urinalysis.
- Change in clinical laboratory assessments of markers of coagulation:
  PT(INR), aPTT, TAT, F_{1+2},D-dimer, PS, PC, AT III, TT
- Change in clinical laboratory assessments of coagulation factors:
  FII, FV, FVII, FVIII, FIX, FX, FXI, FXIII
- Frequency and severity of thrombosis and of TEEs
- Virus status.

For the timing of individual safety parameters refer to section 7.1 Visit Schedule and section III FLOWCHART OF STUDY. Any abnormal observation or finding detected during the screening procedures after subject's informed consent that is considered clinically relevant must be documented as concomitant disease (ongoing medical history) or as past medical history. All medications other than the study medications should also be documented.

9.2.1.1 Adverse Events

After informed consent has been signed, AE data are obtained by the investigator through observation of the subject (including examinations and investigations) before, during and after surgery, from any information volunteered by the subject, and through active questioning. At each visit, subjects will be asked about AE that occurred since the last visit, by questioning them with regard to their well-being by 'non-leading' questions. All AEs should be recorded and reported to safety department (see section 9.3.2). This includes AEs occurring after informed consent has been signed (before, during or after surgery), as well as changes in concomitant diseases (i.e., ongoing medical history).

Occurrence, frequency, nature and severity of AEs will be recorded. This includes observations or abnormalities in physical examination, vital signs, laboratory or other investigations reported as AEs. Any treatments for AEs will also be recorded.

For further details regarding AEs including definitions and reporting procedures refer to Appendix 1: Safety Definitions.

9.2.1.2 Physical Examination

For each subject, a complete physical examination will be performed at the time points specified in III Flowchart of Study (before and after surgery). Physical examination includes inspection of general appearance, skin, neck (including thyroid) eyes, ears, nose, throat, heart, lungs, abdomen, lymph nodes, vascular system, extremities, musculoskeletal system and nervous system. Clinical findings and existing diseases at screening are to be documented as medical and surgical history. A new appearance of an abnormal finding or worsening of a concomitant disease that occurs after signature of informed consent must be documented as AE.

- BW is measured in kilograms (kg) only at screening visit and at baseline.
- Body height is measured in cm once at screening visit for the purpose of calculation of BMI.

9.2.1.3 Vital Signs

Before and after surgery (Screening, baseline, follow-up Day 2 to Day 36) vital signs are measured with the following methods/units:

- Blood pressure is measured in mmHg according to the Riva Rocci method while the subject has been resting in supine position for at least 15 minutes. The same arm should be used for blood pressure measurements throughout the study. The size of the cuff has to be chosen appropriately in relation to the subject's arm circumference.
- Heart rate and pulse is measured in beats per minute (either electronically and/or by palpation for 1 minute) while the subject is supine and has been resting for at least 15 minutes. When heart rate is of concern, cardiac monitors are used to determine not only rate, but also rhythm.
- Respiratory rate per minute.
- Body temperature is measured in °C.

<u>During surgery (Day 1):</u> Surgery/anaesthesia standard methods should be used for monitoring of vital parameters including standard measurement methods for blood pressure, pulse, heart rate and rhythm, respiratory rate and temperature. These

monitoring results are to be reviewed by the investigator, under consultation with a specialist if necessary.

Results must be summarized in writing and classified as 'normal' or 'abnormal'. Abnormal monitoring results must in addition be classified as 'abnormal, clinically not relevant' or 'abnormal, clinically relevant'. Abnormal, clinically relevant findings have to be reported as AE (unless already pre-existing at baseline with the same severity).

In addition, vital signs will be assessed and recorded in the eCRF at the following time-points: prior IMP treatment, 15 and 90 min after start of first IMP administration and end of surgery.

Measurements outside the normal range (according to the age and gender of the subject) or even changed values within the normal range showing a trend have to be assessed for clinical relevance by the investigator, and reported as AE if considered to represent a clinically significant change as compared to pre-treatment values.

9.2.1.4 Laboratory Parameters

All laboratory results have to be evaluated either in the eCRF or in the web-base tool of the central lab by the investigator/subinvestigator (anaesthesiologist) according to the following pattern:

a) Within reference range (normal range)
b) Outside reference range but not clinically relevant (e.g. marginal deviation only, due to underlying medical history diseases in the study population)
c) Outside reference range and clinically relevant

Laboratory values occurring (by date of blood sampling) after signature of informed consent, which are outside the reference range and assessed as clinically relevant (as determined by investigator), have to be documented as AE.

An abnormal laboratory value that is a sign of an AE (e.g. increased leukocytes due to bacterial infection) that has already been reported during the present clinical study, has to be documented as a symptom together with the diagnosis (bacterial infection) and does not constitute a separate AE.

For the routine laboratory parameters, the total volume of blood that will be drawn from a subject who completes the study is defined in the Laboratory Manual.

The laboratory parameters to be assessed are summarized in Table 2:

Table 2: Clinical Laboratory Parameters

Clinical Chemistry	Assessment to be done locally or centrally
• Alanine aminotransferase (ALAT)	• locally
• Aspartate aminotransferase (ASAT)	• locally
• Gamma-glutamyltransferase (ɣ-GT)	• locally
• Alkaline phosphatase (AP)	• locally
• Bilirubin (direct and indirect if total bilirubin is elevated)	• locally
• Creatinine / creatinine clearance	• locally
• Blood Urea Nitrogen (BUN) or Urea (UREA)	• locally
• Sodium	• locally
• Chloride	• locally
• Potassium	• locally
• Calcium	• locally
**Hematology**	
• Hematocrit	• locally
• Hemoglobin	• locally
• Red blood cells (RBCs)	• locally
• White blood cells (WBC)	• locally
• Differential white blood cells	• locally
• Platelets	• locally
**Coagulation**	
**Markers of Coagulation**	
• Prothrombin time (PT) / international normalized ratio (INR)	• locally
• Activated partial thromboplastin time (aPTT)	• locally
• Thrombin-antithrombin III complex (TAT)	• centrally
• Prothrombin fragments (F_{1+2})	• centrally
• D-Dimer	• centrally
• Protein S (PS)	• centrally
• Protein C (PC)	• centrally
• Antithrombin III (AT III) activity	• centrally
• Thrombin Time (TT)	• centrally
**Coagulation Factors**	
• Factor II (FII)	• centrally
• Factor V (FV)	• centrally
• Factor VII (FVII)	• centrally
• Factor VIII (FVIII)	• centrally
• Factor IX (FIX)	• centrally
• Factor X (FX)	• centrally
• Factor XI	• centrally
• Factor XIII (FXIII)	• centrally
**Urinalysis**	
• pH	• locally
• Qualitative for blood	• locally
• White blood cells (WBC)	• locally
• Protein	• locally
• Glucose	• locally
• Ketone bodies	• locally
• Bilirubin	• locally
• Urobilinogen	• locally
• Nitrites	• locally

The following markers of coagulation will be assessed at the time points specified in section III, Flowchart of clinical study:

- Prothrombin Time (PT) / International Normalized Ratio (INR)

  PT is a measure of the integrity of the extrinsic and final common pathways of the procoagulant cascade. PT presents the time for patient plasma to clot after the addition of calcium and thromboplastin as an activator of the extrinsic pathway. Therefore, deficiencies or inhibitors of clotting factors within the extrinsic (factor VII) and final common pathways (factors V, X, II, I [fibrinogen]) result in prolongation of the PT.

  The INR is a mathematical conversion of a patient's PT that accounts for the sensitivity of thromboplastin used by factoring in the international sensitivity index (ISI) value supplied by its manufacturer (Kamal et al., 2007).

- Activated Partial Thromboplastin Time (aPTT)

  aPTT measures the integrity of the intrinsic and final common pathways of the coagulation cascade and represents the time for patient plasma to clot after the addition of phospholipid (intrinsic pathway activator) and calcium (Kamal et al., 2007).

- Thrombin-Antithrombin III Complex (TAT)

  TATs develop during the inactivation of thrombin – the central enzyme of the coagulation system – via complexion with anti-thrombin. TATs are also an indirect measure of thrombin generation. In combination with F_{1+2} a hypercoagulable state can be detected (Wagner C, 2008).

- Prothrombin Fragments 1 and 2 (F1+2)

  During the activation process of prothrombin to thrombin F_{1+2} are split off and represent an indirect measure of thrombin generation. They are useful to detect and follow-up a hypocoagulable state (Wagner C, 2008).

- D-dimer

  D-dimer is a fibrin degradation product, a small protein fragment present in the blood after a blood clot is degraded by fibrinolysis. D-dimer contains two crosslinked D fragments of the fibrinogen protein. D-dimers are produced when fibrin is cleaved by plasmin. The presence of D-dimer may be used to assist with the diagnosis of DIC, Deep Venous Thrombosis (DVT) or Pulmonary Embolism (PE).

- Antithrombin III (AT III) Activity (functional ATIII level)

  Antithrombin is a glycoprotein and is a natural anticoagulant that inhibits the activated coagulation factors thrombin (factor IIa), factor Xa, and, to a lesser extent, factor XIa and factor IXa. Heparin significantly increases the inhibition rate. The antithrombin level does not influence the results of screening coagulation tests such as partial thromboplastin time (PTT), PT, and TT (Teruya and Kostousov, Updated: Jan 30, 2014. Access Date: 15-Jun-2016.).

- Thrombin Time (TT)

  TT is a screening coagulation test designed to assess fibrin formation from fibrinogen in plasma. TT is performed as the next step in the evaluation of abnormally prolonged aPTT or PT (Rodgers and Lehman, 2007).

- Protein C (PC)

  PC is an inactive protein. When activated, it plays a significant part in blood clot, inflammation, and cell death regulation, as well as in maintenance of blood vessel cell wall permeability. The inactive form of PC circulates in blood plasma and is a vitamin K–dependent glycoprotein. When inactive PC binds to thrombin, it becomes activated (Beckmann et al., 1985; Foster et al., 1985; Mather et al., 1996).

  PC has a crucial role as an anticoagulant, and individuals with a PC deficiency or with some type of P activation dysfunction are at much greater risk of thrombosis (Beckmann et al., 1985).

- Protein S (PS)

  PS is a vitamin K-dependent plasma glycoprotein. The two forms of PS are a free form that is active and a complex form (65%) that is inactive (Lundwall et al., 1986).

  PS plays a significant role in the anticoagulation cascade, where it functions as a cofactor to the serine protease activated PC in the inactivation of factors Va and VIIIa. Through direct binding to factors Va, Xa, and VIII, PS exerts activated PC–independent anticoagulant activity (Francis, 1988). If the amount of PC or PS is inadequate or if either one is not functioning properly, thrombin generation essentially remains undeterred, which may promote inappropriate or excessive clotting, with resultant blockage of blood flow in the veins and, rarely, the arteries (thrombosis) (Esmon, 2003; Mosnier et al., 2007). (Bonhomme and Fontana, 2015; Castoldi and Rosing, 2011)

The following coagulation factors will be assessed at the time points specified in section III, Flowchart of clinical study:

- Factor II (FII)

  Clotting factor II, or prothrombin, is a vitamin K–dependent proenzyme that functions in the blood coagulation cascade (Harel et al., 2016).

- Factor V (FV)

  Factor V is an essential component in the blood coagulation cascade. The factor V protein is a catalyst, accelerating the process by which prothrombin is converted to thrombin, the initial step in clot formation.

- Factor VII (FVII)

  Factor VII is a vitamin K-dependent serine protease glycoprotein. The physiological activator of factor VII is thought to be factor Xa. The factor VIIa/tissue factor complex activates both factors IX and X (Roberts et al., 2001).

- Factor VIII (FVIII)

  Factor VIII (antihemophilic factor) is a key factor of the intrinsic clotting cascade. Normal hemostasis requires at least a quarter (25%) of factor VIII activity (Bishnu Prasad Devkota, Updated: Jan 17, 2014. Access Date: Jun 15, 2016.).

- Factor IX (FIX)

  Factor IX, or Christmas factor, is one of the serine proteases of the coagulation system. Factor IX can be activated either by factor XIa or by the factor VIIa/tissue factor complex. In complex with its cofactor, factor VIIIa, factor IXa activates factor X (Roberts et al., 2001).

- Factor X (FX)

  Clotting factor X, or Stuart-Prower factor, is a vitamin K–dependent serine protease that serves as the first enzyme in the common pathway of thrombus formation (Schwartz, Updated: Mar 01, 2017. Access Date: 15-Mar-2017.).

- Factor XI (FXI)

  Factor XI or plasma thromboplastin antecedent is the zymogen form of factor XIa, one of the enzymes of the coagulation cascade. It is a serine protease. Deficiencies of factor XI may lead to a bleeding tendency reflecting the significant role of factor XI in hemostasis (Roberts et al., 2001).

- Factor XIII (FXIII)

  Factor XIII (FXIII), which was initially termed fibrin stabilizing factor, is involved in clot preservation (Shanbhag et al., 2016). Thrombin, generated by reactions initiated by activated tissue factor VII/factor IX pathways, leads to clot formation. Fibrin monomers polymerize spontaneously; this is followed by development of a complex branching clot as a result of the actions of activated FXIII (FXIIIa) (Andersen et al., 2009; Casadio et al., 1999; Fox et al., 1999). Several controls in the complex activation process focus the actions of FXIIIa on fibrin rather than on fibrinogen. Cross-linking of polymerized soluble fibrin by FXIIIa is the final step in hemostasis (Lorand, 2000; 2001). (Sadler, 1998; Shahidi, 2017; Taylor, 2015)

In addition, the following specific safety laboratory parameters will be assessed at the time points specified in section III, Flowchart of clinical study:

- Pregnancy Test

  In females of childbearing potential, a pregnancy human chorionic gonadotropin test in serum will be performed at screening as specified in section III, Flowchart of clinical study. In addition, a second pregnancy test in urine (or serum) will be performed at baseline as specified in section III, Flowchart of clinical study. Pregnancy tests will be performed in the local lab.

- Virus Serology

  The immunological status of viral infections will be assessed at screening and closing visits for HIV, HBV and HCV, section III, Flowchart of clinical study. The following tests will be performed in the local lab:

  - **HIV** (anti HIV1 and anti HIV2)
  - **hepatitis B** (total hepatitis B core antibody [anti HBc], hepatitis B surface antibody [anti HBs], hepatitis B surface antigen [HBsAg])
  - **hepatitis C** (anti HCV)

9.2.1.5 Retention Samples

In order to respond rapidly to any reports on additional viral infections, a pre-treatment serum sample (5 mL) from each subject included in the study must be taken pre-dose and stored at -70 °C for possible future testing (screening visit).
At closing visit (D36) an additional serum sample (5 mL) must be taken and stored up to 6 months after study end.
Viral safety retention samples will be analysed in the central lab (if applicable).

9.2.2 Methods for Assessing and Recording Safety Parameter(s)

The following safety parameters are recorded in the eCRF: AEs, vital signs (including pulse [heart rate], blood pressure, respiratory rate, body temperature), clinical laboratory values of hematology, clinical chemistry and urinalysis. Clinical laboratory assessments of markers of coagulation and coagulation factors: PT(INR), aPTT, TAT, F1+2, D-dimer, PS, PC, AT III, TT, FII, FV, FVII, FVIII, FIX, FX, FXI, FXIII. Virus status.

For documentation of abnormal, clinically relevant findings refer to the respective sections above: section 9.2.1.4.

The results of all analyses performed by the central laboratory PPD , will be transferred to the investigational site, CRO's Data Management, and Biotest CCR&D Medical Advisor in a timely manner. All laboratory analyses performed by the local laboratory using standard assay methods will be transcribed to the eCRF by the investigator also in a timely manner.

For alert processes in the situation of abnormal results refer to Laboratory Manual.

9.2.3 Safety Endpoints

The following variables are defined as secondary safety endpoints:

- AEs
- Changes in vital signs
- Changes in clinical laboratory assessments of hematology, clinical chemistry, and urinalysis
- Changes in clinical laboratory assessments of markers of coagulation
- Changes in clinical laboratory assessments of coagulation factors
- Frequency and severity of thrombosis and of TEEs
- Virus status

9.3 **Adverse Events**

9.3.1 Definitions

(also refer to Appendix 1: Safety Definitions)

- **Adverse Event (AE)**
  Any untoward medical occurrence in a patient or clinical study subject administered an IMP and which does not necessarily have a causal relationship with this treatment. An AE may be any aggravation or new unfavorable and unintended sign, symptom, or disease temporally associated with the use of an IMP, whether or not considered related to the IMP (ICH Guideline for Good Clinical Practice E6(R2)).

- **Adverse drug reaction of an investigational medicinal product (ADR):**
  All untoward and unintended responses to an IMP related to any dose administered. All AEs judged by either the reporting investigator or the sponsor as

having a reasonable causal relationship to a medicinal product qualify as adverse reactions. The expression reasonable causal relationship means to convey in general that there is evidence or argument to suggest a causal relationship. All non-serious AE (related and not related) should also be entered in eCRF as soon as possible but not later than one month after occurrence.

- **Serious Adverse Event (SAE)**

  An SAE is any untoward medical occurrence or effect that at any dose*:
  - results in death
  - is life-threatening
  - requires hospitalization or prolongation of existing hospitalization
  - results in persistent or significant disability / incapacity
  - is a congenital anomaly / birth defect
  - is another important medical event

  * "At any dose" does not necessarily imply that the subject is receiving the study drug at the time of the event.

  Reporting requirements are detailed in Appendix 2.

- **Adverse Event of Special Interest (AESI)**

  An AE of special interest (serious or non-serious) is one of scientific and medical concern specific to the sponsor's IMP or development program, for which ongoing monitoring and immediate communication by the investigator to the sponsor may be appropriate. Such events may require further investigation in order to characterize and understand them. Reporting requirements are detailed in Appendix 2.

  The following AEs have been defined as AESI for this study:
  - Thrombosis or TEE
  - Relevant bleeding complication:

    Relevant changes of a vital sign or laboratory value (e.g. severe tachycardia, severe hypotension, hypovolemic shock, severe anemia) occurring after IMP administration, during and/or after surgery that require immediate corrective action/treatment and are caused by bleeding.

    Note: Corrective treatment includes e.g. the administration of unplanned blood products or other drugs.
  - Suspicion of transmission of infective agents (viral safety).

- **Immediately Reportable Adverse Event (IRAE)**

  An AE that must be reported to the sponsor **within 24 hours** of the study site being aware of the AE. Reporting requirements are detailed in Appendix 2.

  For this clinical study, IRAEs include
  - all SAEs
  - all AESIs (serious and **non-serious**)
  - all AEs that result in a subject's withdrawal from the study (including suspected allergic reaction)
  - Medication error (incl. overdose)

    Pregnancy

- **Adverse Events Leading to Subject's Withdrawal from the Clinical Study**
  An AE, serious or non-serious, resulting in subject's withdrawal from the clinical study, i.e. permanent treatment discontinuation (see section 4.4). Reporting requirements by the investigator are detailed in section Appendix 2.

- **Medication Error**
  A medication error is an unintended failure in the drug treatment process that leads to, or has the potential to harm the patient (EMA Good Practice Guide definition).
  - A 'failure in the drug treatment process' does not refer to lack of efficacy of the drug, rather to human or process mediated failures.
  - The error is unintended. The concepts of intentional overdose, off-label use, misuse and abuse as defined in GVP (good pharmacovigilance practices) Module VI.A.2.1.2 are outside scope and should be clearly distinguished from medication errors.
  - 'Drug treatment process' includes prescribing, storing, dispensing, preparation for administration and administration of a medicine in clinical practice.

  The dose and administration of the study medication is described in section 6 Study Treatment. Any deviation from the study medication (wrong medication, wrong dose, wrong route of administration, wrong patient) is a medication error. Any higher administered dose of the study medication, than described in section 6 Study Treatment, is an overdose.

9.3.2 Recording Adverse Events

All AEs, serious and non-serious, that occur during the period of observation defined for the clinical study (section III, Flowchart of Study) have to be fully documented in the eCRF according to the provisions given in this section of the study protocol and eCRF completion guidelines, as well as in the subject's source data. This applies also to AEs in subjects who signed the informed consent but never received the study drug. AEs considered not related to study medication observed after Day 36 (defined closing visit) will not be recorded in the eCRF.

In addition, for a subset of AEs (SAE, AESI, AE leading to withdrawal) immediate reporting from investigator to sponsor is required (Immediately Reportable Adverse Events, IRAE). This is further detailed in Appendix 2. The following information is necessary:

- **Diagnosis vs. Signs/Symptoms**
  The investigator should provide a diagnosis rather than individual signs and symptoms, wherever possible and appropriate. However, if there is not enough information to provide a diagnosis, individual signs and symptoms are to be recorded. If a diagnosis is accompanied by unusual symptoms, the diagnosis itself and the unusual symptoms have to be reported separately. For serious and other IRAEs the investigator shall provide any other supporting information that may be required for the assessment of the events.

  A complication of an AE constitutes another AE. For example in diarrhea leading to dehydration, diarrhea and dehydration would be captured as separate AEs.

  The eCRF provides for a number of items to be completed for each AE. This includes the onset date, end date, intensity/severity, seriousness, action taken with

study medication, treatment for the AE, outcome, and causal relationship of the AE with the study medication, other drugs, or study procedures (Appendix 2: Reporting Procedures).

Severe vs. Serious: The severity is used to describe the intensity of an event. This is not the same as seriousness, which is based on subject/event outcome or action criteria usually associated with events that pose a threat to subject's life or functioning. Seriousness, not severity, serves as the guide for defining regulatory reporting obligations.

- **Causal Relationship of AE**

  The causal relationship with the study medication has to be reported for each AE. It refers to the presence or absence of a reasonable possibility of a causal relationship between the study medication and the AE.

  The investigator is asked to use medical judgment and take into account the nature of the AE, subject's medical and surgical history, temporal relation, response to withdrawal or interruption of study drug (dechallenge), response to re-introduction of study drug (rechallenge), any alternative explanations such as underlying or concomitant diseases, concomitant drugs, study procedures.

  The investigator should also provide the causality assessment with the NIMP (e.g. background medication, concomitant medication - See Appendix 1 for definition).

  The following categories are used:

  - Related: There is a reasonable possibility of a causal relationship between the study medication and the AE.
  - Not related: There is no reasonable possibility of a causal relationship between the study medication and the AE.

  For serious and other immediately reportable AEs the investigator is asked to specify if there are alternative and/or additional explanations for the occurrence of the event, e.g. concomitant drugs, study procedures, or concomitant/underlying disease and should provide this information already with the initial case report.

9.3.3 Period of Observation

The period of observation for collection of AEs extends from the time the subject signs the ICF until the last study visit (D36), which is scheduled 5 weeks after the day of the surgery (day of IMP administration).

Abnormal, clinically relevant findings or observations made prior to signature of informed consent are to be recorded as medical history/concomitant disease but not as AEs.

AEs (including any change in severity or trait of the concomitant disease/ medical history) occurring in the pre-treatment period between signature of informed consent until first administration of study medication are non-treatment emergent AEs (NTEAEs).

AEs (including any change in severity or trait of the concomitant disease/ medical history) occurring from the administration of study medication until the subject's last study visit are treatment-emergent AEs (TEAEs).

If an SAE occurs in a subject after the period of observation, i.e. after the last study visit, which is considered by the investigator to be related to the study medication, this should be recorded as SAE and follow the immediate reporting process for SAEs as described

in Appendix 2. If the eCRF has been closed for the subject, the investigator should contact the sponsor to determine how to report the SAE (see also section 7.4).

9.3.4 Assessment of Adverse Events

- **Responsibilities of Investigator**

  AEs are assessed by the investigator in a standardized manner including, but not limited to the seriousness, severity, outcome, and causality. This has to be performed in line with the definitions and provisions given in Appendix 1.

  Laboratory values outside the reference range have to be assessed for clinical relevance taking into account the pre-treatment values. For reporting of abnormal laboratory values as AEs refer to section 9.2.1.4.

  If an AE meets the definition of any of the mandatory AE related stopping rules (section 9.3.1), the investigator must withdraw the subject and report the AE as IRAE according to section Appendix 2. If no mandatory AE related stopping rules defined for the study, delete this sentence.

  For all AEs the causality assessment has to be provided in the eCRF, even if based on preliminary data. Once more information is available, the investigator may change a preliminary causality assessment.

  During and after participation of a subject in a clinical study the investigator/institution has to ensure that adequate medical care is provided to the subject for any ongoing AEs including clinically significant abnormal laboratory values. The investigator has to inform the subject when medical care is needed for any intercurrent disease of which the investigator becomes aware.

- **Responsibilities of Sponsor**

  AEs are reviewed and assessed by the sponsor during ongoing safety monitoring activities throughout the study, as well as medical evaluation and regulatory assessment for reportability of SAEs and IRAEs. For the purpose of regulatory reporting, the causality assessment given by the investigator will not been downgraded by the sponsor. If the sponsor disagrees with the investigator's causality assessment, both the opinion of the investigator and the sponsor will be recorded.

  Regulatory assessment of an AE by the sponsor comprises further the assessment of expectedness. For BT524, Fibrinogen Concentrate from Human Plasma it is based on section 7 of the IB . For FFP it is based on the Summary of Product Characteristics of a reference FFP product PPD , solution for infusion, PPD).

- **Follow-up of Adverse Events**

  AEs should be followed up to determine the outcome.

  AEs that are serious or severe or considered related to the study medication or study procedures must be followed up by the investigator until the AE is resolved or resolved with sequelae, and until all queries related to the AE have been

clarified. If the subject had an AE with fatal outcome, an autopsy report should be provided if possible.

If AEs that are serious or severe or considered related to study medication or study procedures are ongoing at the time of the subject's last study visit, or if the subject has clinically relevant laboratory parameter abnormalities at the last study visit, one or more safety follow-up visit should be scheduled for those subjects. The investigator should set the interval to the additional Safety follow-up visit according to his/her medical judgment. Follow-up activities should be continued until the investigator considers it medically justifiable to stop further follow-up.

All other AEs must be followed up by the investigator until the AE is resolved or resolved with sequelae or the end of the period of observation (= last study visit), whichever comes first.

The investigator should respond to any queries raised by the sponsor in relation to AEs, including provision of supporting documentation for SAEs or other IRAEs (e.g. ECG data, laboratory results, hospital summary, autopsy report) within the requested timeline. In case of fatal or life-threatening SAEs the sponsor may request urgent clarification within one calendar day. In general, if for AEs requiring immediate reporting from investigator to sponsor (IRAE/SAE) follow-up information becomes available, this must be reported to the sponsor **within 24 h** of becoming aware of this information (i.e. the same timeframe as for initial IRAE/SAE reports). Any supporting documents have to be identified by the subject ID, and personal data (e.g. subject name, address or phone number) obliterated prior to sending to the sponsor. For details on reporting IRAE/SAE see Appendix 2.

AE data in the eCRF must be updated accordingly when follow-up information is received.

All efforts to collect follow-up information must be documented in the subject's source data.

Subjects who were treated with the study medication but did not complete the study as per protocol, should receive all the examinations and investigations scheduled for the last study visit. The investigator should make all efforts to contact subjects lost to follow-up and document the attempts in the subject's source data.

9.3.5 Immediate Reporting by Investigator to Sponsor

(also refer to Appendix 2: Reporting Procedures)

9.3.6 Use of IMP outside the Specifications of the Clinical Study Protocol

Situations may occur where the IMP is used outside the specifications of the protocol, which may or may not be associated with an AE. These special situations comprise

- Medication errors (including overdose)
- Abuse/ misuse of the IMP.

Such situations, whether or not associated with an AE, are documented in the eCRF on dedicated pages. Any AE that occurred in association with such a special situation has to be cross-referenced on the dedicated eCRF page. An IRAE/SAE occurring in conjunction with a medication error or abuse/ misuse of the IMP has to follow the

immediate reporting process for IRAE/SAE as described in Appendix 2 in addition to its documentation in the eCRF (also refer to sections 9.3.8 and 10.2).

9.3.7 Investigational Medicinal Product Complaints

IMP complaints must be recorded in the eCRF and in addition reported to the sponsor on the "Investigational Medicinal Product Complaint Report Form" **within 24 hours** of the investigator becoming aware of the IMP complaint. If the IMP complaint is associated with an AE, the AE must be entered in the eCRF also.

Any complaint samples should be provided to the sponsor upon request.

9.3.8 Special Situations Requiring Immediate Reporting

Special situations may occur that may or may not be associated with AEs. For these situations special reporting provisions apply.

Special situations comprise:

- Pregnancy in a female study subject or the partner of a male study subject
- Investigational Medicinal Product Complaint.
- Use of IMP outside the specifications of the Clinical Study Protocol (CSP) (e.g. Medication errors, overdose, misuse and abuse (also refer to sections 9.3.6 and 10.2).
- Protocol deviations (refer to section 10.2)

If such a situation occurs, the investigator should contact the sponsor immediately. A special paper report form has to be completed always in these situations and sent to the sponsor immediately, but not later **than 24 hours** after the investigator becoming aware of the situation.

9.3.8.1 Pregnancy

Pregnant women are excluded from the study, and female study subjects of child-bearing potential undergo pregnancy testing at screening and regularly during the study (section III, Flowchart of study). If pregnancy is suspected in a study subject during treatment with the IMP, the IMP must be immediately withheld until the result of a confirmatory test is available. If confirmed, the subject must be withdrawn from the study.

Although not an AE per se, pregnancy in a female study subject or the partner of a male study subject must be recorded if it occurs during the period of observation of the study (see definition in section 9.3.3). The investigator must contact the sponsor immediately in such a situation.

The pregnancy must be documented on a "Drug Exposure Via Parent" (DEVP) Report Form and reported to the sponsor **within 24 hours** of the investigator becoming aware of the pregnancy. If an AE occurs in relation to the pregnancy, it has to be noted on the DEVP form and recorded in the eCRF. If an IRAE/SAE occurs in relation to the pregnancy, it has to be noted on the DEVP form and recorded in the eCRF and follow the immediate reporting process for IRAE including SAE as described in Appendix 2.

The investigator must make all reasonable efforts to follow up the pregnancy until its end and will report all outcomes associated with the pregnancy to the sponsor. In the situation of pregnancy of the female partner of a male study subject, consent for the release of

medical data should be obtained from the female partner to allow collection of information on the outcome of the pregnancy.

9.4 **Data Safety Monitoring Board**

A DSMB will independently monitor the study.

The DSMB will independently review and assess the unblinded safety data throughout the entire study at regular intervals. The DSMB consists of three voting members: an expert in pharmacovigilance, an expert in the field of hematology/hemostaseology and an expert in anaesthesiology. Two members of the DSMB constitute a quorum.

In addition, a statistician without a vote will be responsible for adequate data supply. Prior to the data safety monitoring phase, a meeting will be held to familiarize the DSMB with all relevant procedures. The DSMB members are unblinded during both evaluation periods and will be provided with the following information: reports of SAEs and AEs, data on markers of coagulation and coagulation factors, clinical laboratory assessments of hematology, clinical chemistry and urinalysis, and vital signs.

DSMB meetings will take place at regular intervals. The DSMB will be provided with data covering the screening visit, the day of surgery plus 4 additional follow-up visits (Day 2, 3, 5 and 8) and will evaluate the subjects' risks at a formal DSMB meeting with regards to the relevant parameters and outcome criteria. In addition, subject's data from the closing visit will also be evaluated if data already available at the time of the DSMB meeting. After each meeting treatment of the following subjects can continue unless the DSMB has not actively disapproved it.

Minutes of the DSMB will describe the proceedings from all sessions of the DSMB meeting, and will summarize all recommendations, which will also be reported to Biotest and the principal investigator.

The DSMB members can propose to stop the study at any time after a scheduled or unscheduled meeting in case of major safety concerns related to study treatment.

Further details will be provided in the DSMB Charter.

10 STATISTICS

The statistical planning and evaluation of the clinical study will be carried out by a qualified biostatistician in accordance with the ICH-guidelines and adequate biostatistical SOPs in SAS version 9.4 or later. A detailed Statistical Analysis Plan (SAP), providing details about the statistical methods for the analyses, will be finalized before unblinding. This ensures that the integrity of the analyses is maintained.

Any deviations from the planned analyses will be described and justified in the Clinical Study Report (CSR).

10.1 Analysis Sets

The following analysis sets will be defined:

All Subjects Enrolled Set:
The All Subjects Enrolled Set includes all subjects who have given informed consent to this study.

Safety Set (SAF):
The SAF comprises all subjects who have received at least one dose of IMP. Subjects will be analyzed according to the treatment received.

Full Analysis Set (FAS):
The FAS comprises all subjects who received at least one dose of IMP prior to the 'end of surgery' and have at least one post dose efficacy assessment.

Subjects will be analyzed as randomized.

Per-protocol Set (PPS):
The PPS includes all subjects who are compliant with the study protocol without any major protocol deviations thought to have the potential to impact the results of the efficacy analysis, e.g. no treatment with IMP, treatment with IMP after the 'end of surgery', no post dose efficacy assessment. Classification of protocol deviations as major or minor will be agreed upon at the Blind Data Review Meeting (BDRM) prior to database lock.

Subjects will be analyzed according to the treatment received.

10.2 Protocol Deviations

Deviations from the protocol will be documented on an on-going basis during conduct of the clinical study based on monitoring reports (e.g. failure of eligibility criteria), data management checks and statistical programming (e.g. prohibited medications based on drug codes). Protocol deviations will be discussed and agreed in the BDRM to find protocol deviations with major impact on subject safety or the validity of the study data. Subjects with major protocol deviations will be excluded from the PPS under the assumption that the deviation may have an impact on the efficacy analysis.

The investigator should not implement any deviation from, or changes of the protocol without agreement by the sponsor and prior review and documented approval/ favorable opinion from the IRB/IEC of an amendment, except where necessary to eliminate an immediate hazard(s) to study subjects. The investigator, or person designated by the investigator, should document and explain any deviation from the approved protocol.

10.3 General Considerations

BT524 will be compared with standard treatment (FFP/cryoprecipitate) whereas FFP and cryoprecipitate will be considered as one treatment group.

The global significance level will be 2.5% (one-sided), confidence intervals will be 95% (two-sided). All statistical tests will be two-sided, unless otherwise stated.

Quantitative (continuous) data - absolute values and differences from baseline, where appropriate - will be summarized with number of observations (n), arithmetic mean, standard deviation, median, minimum, and maximum.

Qualitative (categorical) data will be summarized using number of observations (n), frequency and percentages of subjects. Unless stated otherwise the calculation of percentages will be based on the total number of subjects in the population of interest. Thus counts of missing observations will be included in the denominator and presented as a separate category.

Definition of Baseline
If not stated otherwise, the last non-missing valid observation prior to surgery will serve as the baseline measurement.

Missing Data Conventions
In this short study, not many missing values are expected. Therefore, data will not be imputed for safety analyses or continuous efficacy endpoints.

For binary endpoints, an observed case analysis (excluding missing data) will be considered to be the primary analysis method and a non-responder analysis (treating missing values as non-responders or the worst case) may be performed as a sensitivity analysis if deemed necessary. Details will be given in the SAP.

Pooling of Centers
In case of low number of subjects per center, summaries of data by center would be unlikely to be informative. Therefore, data from all centers per country/region (if applicable) and in total will be pooled prior to analysis.

Subgroups
A subgroup analysis according to the predictive blood loss is planned at least for the primary efficacy variable blood loss.

Disposition
The number of subjects screened and who failed screening prior to surgery or during surgery will be summarized. The number of subjects randomized; number and percentage of subjects treated with IMP; and number and percentage of subjects who prematurely withdrew from the study with the reason for withdrawal will be summarized by treatment arm. The number and percentage of subjects in each of the analysis sets will also be summarized by treatment arm.

Demographic and Baseline Data
Demographic and baseline data will be summarized descriptively.

10.4 Efficacy Analyses

Analyses of the efficacy parameters will be based on the PPS and FAS as appropriate and defined in the SAP.

10.5 Primary Efficacy Analysis

The primary endpoint / efficacy variable is the intra-operative blood loss after decision to treat the subject with IMP until the end of surgery as measured and calculated by amount of blood from the blood suction unit and amount of blood from surgical cloths and compresses.

The primary analysis of this endpoint will test for non-inferiority in the PPS.

The null hypothesis for the primary analysis is that the degree of inferiority of BT524 compared to standard treatment (FFP/cryoprecipitate) is greater than or equal to the non-inferiority margin. The alternative hypothesis is that the degree of inferiority of BT524 compared to FFP/cryoprecipitate is less than the non-inferiority margin.

H_0: $\mu_1 - \mu_2 \geq \delta$
H_1: $\mu_1 - \mu_2 < \delta$

where

μ_1 = mean intra-operative blood loss after the decision to treat the subject with IMP in the BT524 treatment arm
μ_2 = mean intra-operative blood loss after the decision to treat the subject with IMP in the FFP-/cryoprecipitate-treatment arm
δ = non-inferiority margin = 150 mL

The final analysis will be performed using ANCOVA with the intra-operative blood loss after the decision to treat the subject with IMP until the end of surgery as the dependent variable and the predictive blood loss (>1,000 mL to ≤ 2,000 mL and > 2,000 mL) as a covariate. The least square means and difference in least square means will be presented with the corresponding 95% confidence intervals and 2-sided p-value. Non-inferiority will be demonstrated if the upper confidence limit of the 2-sided 95% confidence interval for the difference in the least square means is less than the non-inferiority margin (150 mL).

This analysis will also be performed in the FAS as a sensitivity analysis.

If non-inferiority is demonstrated, then superiority will be assessed in the FAS with superiority demonstrated if the 2-sided p-value is less than 0.05 (i.e. the upper confidence interval is less than 0 mL) using the analysis performed to assess Non-Inferiority.

No imputation for missing values will be applied.

10.6 Secondary Efficacy Analyses

All secondary efficacy analyses will be conducted with the FAS. The secondary endpoints / efficacy variables are:

- Proportion (%) of subjects with successful correction of fibrinogen level 15 minutes after start of first IMP administration. Successful correction of fibrinogen level in a subject is defined as restoring fibrinogen FIBTEM A10 baseline levels measured by ROTEM 15 minutes after start of first IMP administration. A correction of at least 95% is considered successful.
- Time to first successful correction of fibrinogen level (15 minutes or 90 minutes after start of first IMP administration, end of surgery, not within surgery).

- Total amount of transfusion products (allogenic blood products) or autologous blood transfusion infused after start of first IMP administration until end of surgery.
- Amount of RBCs (allogenic and autologous) infused after start of first IMP administration until end of surgery.
- Post-operative blood loss in the first 24 hours.
- Proportion (%) of subjects with rebleeds after the end of the surgery until Day 8.
- Hospital length of stay after surgery.
- In-hospital mortality.

10.6.1 Correction of Fibrinogen Level

The proportion of subjects with a successful correction of fibrinogen level will be compared between the treatment arms using a CMH approach stratified by predictive blood loss (> 1,000 mL to ≤ 2,000 mL and > 2,000 mL). The number and percentage of subjects with a successful correction of fibrinogen level will be presented with corresponding 95% confidence intervals. The estimated treatment effect (i.e., the difference in correction rate between the treatment arms), corresponding 95% confidence interval, and 2-sided p-value for the difference will be presented.

The time to first successful correction of fibrinogen level will be compared between the treatment arms using a Chi-Square test. The number and percentage of subjects reaching a successful correction at each time point (15 minutes or 90 minutes after start of first IMP administration, end of surgery, not within surgery) will be presented together with the p-value for differences between treatment arms.

Absolute values and change from baseline in fibrinogen levels will be presented descriptively over time by treatment arm.

10.6.2 Consumption of Transfusion Products

The total amount of transfusion products (allogenic blood products or autologous blood transfusion or cell salvage) infused until end of surgery will be descriptively summarized by type of transfusion product and treatment arm.

10.6.3 Amount of Red Blood Cells

The amount of RBCs required intra-operatively will be descriptively summarized by treatment arm.

An ANCOVA analysis will be performed with the amount of RBCs required as the dependent variable and the predictive blood loss (> 1,000 mL to ≤ 2,000 mL and > 2,000 mL) as a covariate. The least square means and difference in least square means will be presented with the corresponding 95% confidence intervals and 2-sided p-value.

10.6.4 Post-operative Blood Loss

The post-operative blood loss in the first 24 hours will be descriptively summarized by treatment arm.

An ANCOVA analysis will be performed with the post-operative blood loss in the first 24 hours as the dependent variable and the predictive blood loss (> 1,000 mL to ≤ 2,000 mL and > 2,000 mL) as a covariate. The least square means and difference in least square

means will be presented with the corresponding 95% confidence intervals and 2-sided p-value.

10.6.5 Proportion of Subjects with Rebleeds

The proportion of subjects with rebleeds after the end of surgery (until Day 8) will be compared between the treatment arms using a CMH approach stratified by predictive blood loss (> 1,000 mL to ≤ 2,000 mL and > 2,000 mL). The number and percentage of patients with a rebleed will be presented with corresponding 95% confidence intervals. The estimated treatment effect (i.e., the difference in rebleed rate between the treatment arms), corresponding 95% confidence interval, and 2-sided p-value for the difference will be presented.

10.6.6 Hospital Length of Stay after Surgery

The hospital length of stay after surgery will be descriptively summarized by treatment arm.

10.6.7 In-hospital Mortality

The number and percentage of subjects died during hospital stay will be presented by treatment arm.

10.7 **Safety Analysis**

AEs will be coded using the most current version of Medical Dictionary for Regulatory Activities (MedDRA®). The version used will be defined in the SAP. Incidence rates (i.e. number and percentage of affected subjects) will be calculated for the coding levels *SOC* and *preferred term* and will be presented by treatment arm. Further analyses of AEs will focus on seriousness, intensity, causal relationship to IMP, and outcome. IRAE including SAEs etc. will be displayed in detail.

Safety laboratory assessments (hematology, clinical chemistry, urinalysis and coagulation parameters) will be categorized with respect to the laboratory specific reference ranges as normal/abnormal. Abnormal values will be further classified with respect to clinical relevance. Changes over time will be described by means of “shift-tables” by treatment arm as well as summarized with descriptive statistics by time point and treatment arm.

Vital signs data will be summarized descriptively before and after surgery by treatment arm.

The frequency of thrombosis and of TEEs, virus status, exposure data and concomitant medication will be summarized by treatment arm.

All safety analyses will be based on the SAF.

10.8 **Interim Analyses**

In this study, 3 interim analyses of the observed blood losses are planned to have the option of adjusting the sample size needed (primary: PPS, secondary: FAS).

For this purpose, an alpha-adjustment according to Haybittle/Peto (Haybittle, 1971; Peto et al., 1976; Schulz and Grimes, 2005) is planned. This leads to local alpha levels of 0.001 for each interim analysis and a significance level of 0.05 for the final analysis to reach a global significance level of 5%.

All interim analyses will be based on the PPS.

The first interim analysis is planned with approximately 50 spine subjects, the second one with at least 40 PMP subjects and all other evaluable spine subjects at that time-point. The third interim analysis is planned after approximately 80% of subjects of the total sample size.

Aim of all interim analyses is to adapt the sample size according to the observed blood losses and the standard deviations:

a.) Early termination due to non-inferiority of BT524 in comparison with the used standard therapies.
b.) Continuation with the sample size as initially planned.
c.) Adjustment of sample size to take into account changes from the previous assumptions on the additional blood loss.
d.) Stopping the study early due to futility if the sample size re-estimation indicates a much higher number than planned before.

10.9 Determination of Sample Size

The non-inferiority margin is defined as 150 mL blood loss, as such difference in blood loss after the decision to treat the subjects with IMP is considered as clinically not relevant.

The intra-operative blood loss of approximately 1 L, requiring hemostatic treatment during surgery, is defined as the time of decision to treat the subjects with IMP. Based on the assumption of an additional blood loss of about 500 mL in the FFP-/cryoprecipitate-treatment arm after the decision to treat, a further intra-operative blood loss of approximately 150 mL would not lead to a further transfusion of ≥ 1 unit of packed RBCs, ≥ 1 unit of FFP/cryoprecipitate, or ≥ 1 unit of whole blood.

Whole blood contains RBCs and plasma components of circulating blood. A single whole blood donation contains approximately 500 mL of blood with a minimum hematocrit of 38%. When the plasma is removed, RBCs remain and have a hematocrit of > 80% and a volume of 225-350 mL. Additive solutions mixed with the red cells result in a hematocrit of 55-65% and a volume of 300-400 mL. One unit of whole blood or one unit of RBCs can be expected to result in a hemoglobin increase of 1 g/dL or a hematocrit increase of 3% in a typical adult. Therefore, one unit of RBCs can replace a blood loss of 500 mL (Avery and Avery, Spring 2010; Liumbruno et al., 2009). Accordingly, a volume of 150 mL (after an assumed blood loss of 500 mL) is still below a clinically relevant blood loss and would not trigger an additional administration of transfusion products.

It is assumed that BT524 is non-inferior that means not worse than FFP/cryoprecipitate with a non-inferiority margin of 150 mL in reducing intra-operative blood loss. Assuming a blood loss of about 500 mL in the FFP-/cryoprecipitate-treatment arm after the decision to treat the subject with IMP until end of surgery, a standard deviation of 375 mL, a non-inferiority margin of 150 mL, an alpha-level of 2.5% (1-sided) 100 evaluable subjects per treatment arm are needed to demonstrate the Non-Inferiority of BT524 by using a t-test (equivalence) with 80% power.

The sample size will be recalculated at the interim analyses as defined in section 10.8. Sample size estimations will be performed by using nQuery Advisor Version 4.0 or higher.

With 100 subjects per treatment arm superiority of BT524 can also be tested with a power of >80% (t-test, alpha=0.05 2-sided, effect size Δ=0.5).

10.9.1 Data Monitoring

After 40 subjects have completed the study, the overall mean and standard deviation for the primary efficacy variable intra-surgery blood loss after decision to treat the subject with IMP will be derived using blinded aggregate data of all 40 subjects without separating according to treatment.

If the assumed mean and standard deviation blood loss are not reflected in these subjects, then a sample size adjustment will be considered to ensure that a sufficient number of subjects has been randomized to maintain a power of 90%.

If an adaption of the sample size is intended, this will be documented in a protocol amendment.

This data monitoring is not an interim analysis because the analysis is performed with all subjects without separating the subjects according to treatment. Therefore, no alpha-adjustment is necessary.

11 DATA MANAGEMENT

11.1 Data Collection

Electronic Case report form (eCRF)

The eCRF is the primary data collection instrument for the clinical study. All data to be recorded according to this CSP must be documented. Entries in the eCRF must only be made by the investigator or persons authorized by the investigator. A list of all persons who are allowed to make entries in the eCRF must be available in each study site.

eCRF completion guidelines will be provided as electronic version with the eCRF as a link on the dashboard.

Clinical study data will be directly entered via eCRF into the study database on a central server by authorized investigator and/or study personnel.

It is ensured that the *electronic data capture (*EDC) system (including the eCRF) is built up with following requirements: validated system, functionality of different user roles and access administration, password protection, given traceability, record keeping, and availability of audit trail functionality as well appropriate standard operation procedures are maintained.

The investigator and/or assigned study personnel at each site will enter data from source documents corresponding to a subject's visit into the protocol-specific eCRF. Subjects will be identified by a unique study specific number and/or code in any database. The subject's name and any other identifying detail will not be included in any study data-electronic file.

Laboratory samples (e.g. safety lab, clinical immunology, pharmacokinetic, other study specific laboratory data) will be collected and analyzed in local labs at each site or shipped to central lab for analysis (section 9.2.1.4). The results of local lab samples will be sent back to the investigator to be entered into the eCRF. The central lab data will be available for evaluation by the investigator via the web-based tool provided by the vendor.

Data will be sent to the CRO on a regular basis to join with the clinical data entered via eCRF.

The complete data management activities (data entry, data validation, query handling, data editing after entry, coding, data base closure, etc.) will be defined in advance within a data management plan together with a description of the personnel responsible for data correction, performance, and controlling as well as specific data handling procedures.

MedDRA® dictionary will be used for coding of AE, concomitant diseases and medical history. Concomitant medication will be coded using the Word Health Drug Dictionary (WHO-DD). Details are provided in the data management plan and the study specific safety manual.

11.2 Correction of Data

After data have been entered into the clinical study database, a system of computerized data validation checks will be implemented and applied to the database on a regular basis.

Definition and details are provided in the data validation specifications.

Queries are entered, tracked, and resolved through the eCRF system directly. If a correction is required for an eCRF, the time and date stamps tracking function creates an electronic audit trail for the person entering/updating the eCRF data.

11.3 Data Handling

The data will be entered into a validated database. The Data Management personnel will be responsible for data processing, in accordance with procedural documentation. Database lock will occur once all data entered are clean and quality assurance procedures have been completed.

All procedures for the handling and analysis of data will be conducted according to available ICH-GCP guidelines for the handling and analysis of data for clinical studies.

12 QUALITY CONTROL AND QUALITY ASSURANCE

12.1 Study Initiation Activities

The investigator(s) is/are informed about objectives and methods of the study, the inclusion and exclusion criteria, the time-schedule, and the applied procedures by means of a Pre-Study Visit by the monitor (if necessary), an investigators' meeting prior to start of the study, and during the Site Initiation Visit by the monitor.

12.2 Training of site staff

The Principal Investigator needs to ensure that all persons assisting with the clinical study are adequately informed about the protocol, the investigational product(s) and their study related duties and functions. Furthermore the Principal Investigator is requested to maintain a list of appropriately qualified persons to whom the investigator has delegated significant study-related duties.

12.3 Documentation and Filing

List of Subjects (subject identification log)

The investigator is asked to keep a confidential list of names of all subjects participating in the study, giving reference to the subjects' records.

With the help of this list it must be possible to identify the subjects and their medical records. In addition, the investigator is asked to keep a list of all subjects screened on a screening log to document identification of subjects who entered pre-study screening. In case of non-eligibility a reason is to be provided.

Source Data

Source data is all information in original records and certified copies of original records of clinical findings, observations, or other activities in a clinical study necessary for the reconstruction and evaluation of the study. Source data are contained in source documents which comprise clinical documentation, data, and records (e.g. hospital records, clinical and office charts, laboratory notes, memoranda, subjects' diaries or evaluation checklists, pharmacy dispensing records, recorded data from automated instruments, copies or transcriptions certified after verification as being accurate copies, microfiches, photographic negatives, microfilm or magnetic media, x-rays, subject files, and records kept at the pharmacy, at the laboratories and at medico-technical departments involved in the clinical study). Any data recorded directly in the eCRFs (i.e. no prior written or electronic record of data) will also be considered to be source data.

Investigator Site File / Regulatory Binder

Before site initiation the CRO will provide an Investigator Site File / Regulatory Binder to each study site. The Investigator Site File will include essential documents as defined by the ICH GCP guideline and applicable local requirements.

The investigator will be responsible for the continual update and maintenance of the investigator site file, which will be periodically reviewed by the monitor(s). In case of an audit by the sponsor or an inspection by the Regulatory Authorities these documents will be reviewed.

All study related documents are to be archived and stored according to legal requirements, but at **least for 25 years** after completion of the study.

Prior to destruction the investigator will contact Biotest AG for approval and conformation of such.

12.4 Monitoring

The monitor is responsible for checking the quality of data and adherence to the study protocol and to legal and ethical requirements according to local laws and GCP.

The interval between monitoring visits will be dependent on the recruitment rate and the complexity of the study.

Source data verification is an essential part of the monitoring process and the investigator must grant direct access to the subjects' source data.

The extent and nature of monitoring will be described in detail within the monitoring plan.

12.5 Audits and Inspections

Audits will be performed according to the corresponding audit program, including the possibility that a member of the sponsor's quality assurance function may arrange to visit the investigator in order to audit the performance of the study at the study site, as well as all study documents originating there. Audits may also be performed by contract auditors. In this case, the sponsor's quality assurance function will agree with the contract auditor regarding the timing and extent of the audit(s). In case of audits at the investigational site, the monitor, PM-CRO (Project Manager CRO) or CCR (Clinical Manager (cM) Biotest) will usually accompany the auditor(s).

Inspections by regulatory authority representatives and IECs/IRBs are possible at any time, even after the end of study. The investigator has to notify the sponsor immediately of any such inspection. The investigator and institution will permit and support study-related monitoring, audits, reviews by the IEC/IRB and/or Regulatory Authorities, and will allow direct access to source data and source documents for monitoring, audits, and inspections. The principal investigator shall personally participate in all audits and inspections.

12.6 Archiving

After evaluation and reporting of the study data, all documents relating to the clinical study will be kept in the archives of the sponsor or of a contracted service provider and the study site(s) according to applicable regulatory requirements.

13 GENERAL REGULATIONS, AGREEMENTS AND ORGANISATIONAL PROCEDURES

13.1 Study Administrative Structure

Details for the study administrative structure are kept as a separate list filed in the Trial Master File.

13.2 Ethical and Regulatory Considerations

This CSP and any amendments will be submitted to a properly constituted Independent Ethics Committee (IEC) / Institutional Review Board (IRB) and/or Regulatory Authorities (RA), in agreement with applicable regulatory requirements, for formal approval of the study conduct. A copy of these approvals must be submitted to Biotest before initiation of the clinical study and each site needs to keep a copy of these documents.

Changes to the CSP must be made in the form of an amendment that has the prior written approval of Biotest. Substantial CSP amendments need to be notified to/approved by IEC/IRB and/or Competent Authorities (CA) / Regulatory Authorities (RA) prior to implementation as required by applicable regulations.

The clinical study will be performed according to the applicable regulatory requirements taking into account the principles of GCP and the latest version of the Declaration of Helsinki.

13.3 Committees / Monitoring Boards

Safety data from the clinical study will be evaluated by a DSMB at regular intervals during the study, to ensure that the continuation of the study is appropriate and to make recommendations to the sponsor. The DSMB will consist of permanent members who are not associated with the sponsor or with the operative conduct of the study. A description of the scope of work and operating procedures for the DSMB is provided in the DSMB Charter. The composition of the DSMB will also be outlined in the DSMB Charter.

13.4 Written Agreements

A written agreement will be set up between Biotest and each investigator setting out any arrangements on delegation and distribution of tasks and obligations and, if appropriate, on financial matters.

13.5 Insurance/Liability

In accordance with the relevant national regulations, the sponsor has taken out a subject liability insurance for all subjects who have given their consent to the clinical study. The subjects are insured against injury caused by study medication or participation. The subjects will be informed about the insurance and their own responsibilities and duties.

The insurance company issuing the policy is defined by the Insurance Certificate for Clinical Trials for the respective county. This certificate will comply with the country-specific legal requirements.

13.6 Investigator's Brochure (IB)

The investigator will be informed about current knowledge concerning the study medication BT524 through an Investigator´s Brochure (IB). All investigators will be informed immediately about relevant new information available.

13.7 Amendments to the Protocol

Changes to the CSP must be made in the form of a CSP Amendment that has the prior written approval of Biotest. Substantial changes to the protocol need to be notified to/approved by IEC/IRB and/or Regulatory Authorities prior to implementation, as required by applicable regulations.

Amendments in order to eliminate immediate hazard to subjects may be implemented before the approval of the IEC/IRB and/or Regulatory Authorities after consultation with Biotest.

In the event that a significant deviation from the protocol is anticipated based on the subjects status, or occurs due to an accident or mistake, the investigator or his/her designee must contact Biotest or PPD (CRO) at the earliest possible time. This will allow an early joint decision to be made as to whether or not the subject should continue in the study. This decision will be documented by both the investigator and Biotest or PPD (CRO).

13.8 Confidentiality

The objectives and contents of this clinical study as well as its results are to be treated as confidential and may not be made accessible to third parties.

Information about study subjects will be kept confidential and managed according to the requirements of the Health Insurance Portability and Accountability Act of 1996. Those regulations require a signed subject authorization informing the subject of the following:

- What protected health information (PHI) will be collected from subjects in this study
- Who will have access to that information and why
- Who will use or disclose that information
- The rights of a research subject to revoke their authorization for use of their PHI.

In the event that a subject revokes authorization to collect or use PHI, the investigator, by regulation, retains the ability to use all information collected prior to the revocation of subject authorization. For subjects that have revoked authorization to collect or use PHI, attempts should be made to obtain permission to collect at least vital status (i.e. that the subject is alive) at the end of their scheduled study period.

13.9 Final Report and Publication

For each study an integrated final report according to ICH-requirements will be produced. At the end of the study the sponsor will provide the competent authority and IEC/IRB with a summary of the CSR **within < 1 year** after the end of the study, where required.

It is generally recommended that the results of clinical studies be presented at congresses and symposia and/or published in scientific journals. Prior to their publication, all results of medical tests with the sponsor's products, and/or publications or lecture manuscripts concerning such results, are to be reviewed and discussed by the coordinating investigator and the sponsor by mutual agreement.

Each investigator is obligated to keep data pertaining to the study secret. He/she must consult with the sponsor before any study data are published.

The legitimate interests of the sponsor, such as acquiring optimum patent protection, coordinating submissions to the health authorities or coordination with other studies in the same field that are underway, protection of confidential data and information, etc. will be given due consideration by all partners involved.

14 LIST OF REFERENCES

PPD [redacted]

[redacted]

[redacted]

[redacted]

[redacted]

[redacted]

[redacted]

[redacted]

[redacted]

[redacted]

[redacted]

PPD [redacted]

[redacted]

[redacted]

[redacted]

[redacted]

[redacted]

[redacted]

[redacted]

[redacted]

[redacted]

[redacted]

[redacted]

[redacted]

PPD [redacted]

[redacted]

[redacted]

[redacted]

[redacted]

[redacted]

[redacted]

[redacted]

[redacted]

[redacted]

[redacted]

[redacted]

PPD [redacted]

[redacted]

[redacted]

[redacted]

[redacted]

[redacted]

[redacted]

[redacted]

[redacted]

[redacted]

[redacted]

[redacted]

PPD [redacted]

[redacted]

[redacted]

[redacted]

[redacted]

[redacted]

[redacted]

[redacted]

[redacted]

[redacted]

[redacted]

PPD [redacted]

[redacted]

[redacted]

[redacted]

[redacted]

[redacted]

[redacted]

[redacted]

[redacted]

[redacted]

[redacted]

[redacted]

PPD [redacted]

[redacted]

15 APPENDICES

Appendix 1: Safety Definitions

Appendix 2: Reporting Procedures

Appendix 1: Safety Definitions

- **Adverse Event (AE)**

  Any untoward medical occurrence in a patient or clinical study subject administered an IMP and which does not necessarily have a causal relationship with this treatment. An AE may be any aggravation or new unfavorable and unintended sign, symptom, or disease temporally associated with the use of an IMP, whether or not considered related to the IMP.

  This includes abnormal laboratory and other investigation results which are considered clinically relevant by the investigator (unless already pre-existing at baseline). However, if an abnormal laboratory value is a sign of an already reported AE (e.g. infection), the respective abnormal laboratory value does not constitute a separate AE.

  A surgical or invasive procedure is not an AE in itself. Instead, the condition for which the surgical or invasive procedure is performed may be an AE. Planned or elective surgery or procedures (i.e. planned prior to signature of informed consent) for a pre-existing condition and the pre-existing condition leading to surgery or procedure are not AEs. However, if the pre-existing condition worsened after signature of informed consent, the worsening of the condition constitutes an AE.

  Worsening of the disease under study (underlying disease): This will be captured by efficacy parameters and should not usually be recorded as AE, unless one or more of the following criteria are met:

  - The worsening of the disease under study constitutes a serious AE
  - A deterioration exceeding the usual fluctuations of the disease under study has occurred in the opinion of the investigator
  - The worsening leads to discontinuation of the study medication
  - Additional treatment is required for the worsening, e.g. concomitant medication is added or changed.

  No causal relationship with the investigational drug, or comparator drug, or study procedures is implied by the use of the term “Adverse Event”.

- **Adverse reaction of an investigational medicinal product:**

  All untoward and unintended responses to an IMP related to any dose administered. All AEs judged by either the reporting investigator or the sponsor as having a reasonable causal relationship to a medicinal product qualify as adverse reactions. The expression reasonable causal relationship means to convey in general that there are facts, evidence or arguments to suggest a causal association with the drug.

- **Serious Adverse Event (SAE)**

  An SAE is any untoward medical occurrence or effect that at any dose*:

  - results in death
    - *Death is an outcome of an AE and not an AE in itself. All deaths, regardless of cause or relationship must be reported for study subjects.*
  - is life-threatening

  - *"Life-threatening" refers to an event in which the subject was at risk of death at the time of the event; it does not refer to an event which hypothetically might have caused death if it were more severe.*
- requires hospitalization or prolongation of existing hospitalization
  - *In-subject hospitalization means that the subject has been formally admitted to a hospital for medical reasons, for any length of time, which may or may not be overnight. It does not include presentation and care within an emergency department.*
  - *A complication that occurs during hospitalization and prolongs the existing hospitalization is an SAE. Complications that occur during hospitalization but do not prolong the existing hospitalization and do not meet any other seriousness criteria are non-serious AEs.*
  - *Elective or pre-planned (prior to signature of informed consent) hospitalization for investigations, medical or surgical treatment does not meet this seriousness criterion. However, if the underlying condition for which hospital treatment or surgery had been planned worsened during the study, the worsening of the condition is to be reported as SAE.*
- results in persistent or significant disability / incapacity
- is a congenital anomaly / birth defect
- is another important medical event
  - *Adverse events that may not be immediately life-threatening or result in death or hospitalization but my jeopardize the subject or may require medical or surgical intervention to prevent one of the other outcomes listed above, should be reported as serious. Medical and scientific judgment must be exercised in deciding whether an event is serious.*

* "At any dose" does not necessarily imply that the subject is receiving the study drug at the time of the event.

- **Diagnosis vs. Signs/Symptoms**

The investigator should provide a diagnosis rather than individual signs and symptoms, wherever possible and appropriate. However, if there is not enough information to provide a diagnosis, individual signs and symptoms are to be recorded. If a diagnosis is accompanied by unusual symptoms, the diagnosis itself and the unusual symptoms have to be reported separately. For serious and other IRAEs the investigator shall provide any other supporting information that may be required for the assessment of the events, specifically in the free text narrative description of the case. This is of particular importance in situations where a diagnosis cannot (yet) be made. Any subject identifying data on supporting documents (e.g. name, address, phone number) have to be obliterated prior to sending them to the sponsor.

A complication of an AE constitutes another AE. For example in diarrhea leading to dehydration, diarrhea and dehydration would be captured as separate AEs.

The eCRF provides for a number of items to be completed for each AE. This includes the onset date, end date, intensity/severity, seriousness, action taken with study medication, treatment for the AE, outcome, and causal relationship of the AE with the study medication, other drugs, or study procedures.

- **Onset date, end date**

If an AE started during the study but did not end before the final follow-up visit, the investigator must make a reasonable effort to establish the outcome and the end date. If this is not possible, e.g. because the AE is still ongoing, or the subject is lost to follow-up, there will be no end date for the AE.

For all AEs that resolve, resolve with sequelae, or have a fatal outcome, an end date must be provided.

If an AE stops and restarts later, all occurrences have to be recorded separately.

If an AE starts as non-serious AE and becomes serious at a later point in time, the following applies in regard to onset and end dates:

- **Intensity/Severity**

Refers to the extent to which an AE affects the subject's daily activities. Severity will be categorized according to the following criteria:

Table: Adverse Event Severity

**Mild**	The AE does not interfere with the subject's routine activities.
**Moderate**	The AE interferes with the subject's daily routine, but usual routine activities can still be carried out.
**Severe**	The AE results in inability to perform routine activities.

Severe vs. Serious: The severity is used to describe the intensity of an event. This is not the same as seriousness, which is based on subject/event outcome or action criteria usually associated with events that pose a threat to subject's life or functioning. Seriousness, not severity, serves as the guide for defining regulatory reporting obligations.

Example: While an event may be of "severe" intensity, it may be of relatively minor medical significance, such as severe headache. On the other hand, a myocardial infarction would be usually regarded as serious, even if its intensity is "mild".

If formal severity classifications are used, such as the NCI CTC classification used in oncology studies, provisions should be made regarding the reporting of events of defined grading (e.g. ≥ 3) as serious.

AEs with changes in severity: If an AE changes in severity, this will be captured as one AE with the highest severity grade recorded.

- **Seriousness**

  For definition of seriousness criteria refer to section 9.3.1. For a serious AE all seriousness criteria that apply have to be reported. Reporting requirements by the investigator are detailed in Appendix 2.

  If a list of always serious terms is to be used in the study, this should be mentioned here, as well as its use by the investigator. For example a PT-based auto-seriousness in the eCRF, or an urgent query process once a verbatim is coded to a PT on the list.

- **Action Taken with Study Medication**

  The action taken with study medication as a result of the AE has to be documented. In the situation that the AE leads to permanent discontinuation of the study medication, this meets the definition of an AE leading to subject's withdrawal from the study, which is an immediately reportable AE (IRAE). Reporting requirements by the investigator are detailed in section Appendix 2.

- **Treatment for the AE**

  It has to be specified in the eCRF if counteractive treatment was given for the AE. Any treatment for an AE, whether pharmacological or other (e.g. surgical) treatment, has to be recorded in the eCRF.

- **Outcome**

  The following categories are used:

  - Resolved
    - Indicates that the event has fully resolved.
  - Resolving
    - Indicates that the event is in the process of recovery but has not yet fully resolved.
  - Not resolved
    - Indicates that the event is ongoing and there has been no recovery.
  - Resolved with sequelae
    - Indicates that there is a residual, possibly permanent consequence of the event (e.g. residual hemiparesis subsequent to stroke).
  - Fatal
    - Indicates that the subject died due to the event. The outcome "fatal" applies only to the event(s) that were the cause(s) of death. For other AEs that were ongoing at the time of death, the outcome must not be "fatal" but "not resolved".

- **Causal Relationship of AE**

  The causal relationship with the study medication has to be reported for each AE. It refers to the presence or absence of a reasonable possibility of a causal relationship between the study medication and the AE. The investigator is asked to use medical judgment and take into account the nature of the AE, subject's

medical history, temporal relation, response to withdrawal or interruption of study drug (dechallenge), response to re-introduction of study drug (rechallenge), any alternative explanations such as underlying or concomitant diseases, concomitant drugs, study procedures.

The following categories are used:

- Related: There is a reasonable possibility of a causal relationship between the study medication and the AE.
- Not related: There is no reasonable possibility of a causal relationship between the study medication and the AE.

For serious and other immediately reportable AEs the investigator is asked to specify if there are alternative and/or additional explanations for the occurrence of the event, e.g. concomitant drugs, study procedures, or concomitant/underlying disease and should provide this information already with the initial case report.

- **Concomitant medication**

Concomitant medication will be documented according to categories of medicinal products which are normally used in clinical studies as NIMPs (European Commission, 18/03/2011):

(1) Rescue medication
(2) Challenge agents
(3) Concomitant medicinal products systematically prescribed to the study patients
(4) Background treatment

Definitions adapted from the Guidance on IMPs and NIMPs (European Commission, 18/03/2011):

- **Rescue medication**

Rescue medications are medicines identified as those that may be administered to the patients when the efficacy of the IMP is not satisfactory, or the effect of the IMP is too great and is likely to cause a hazard to the patient, or to manage an emergency situation. Rescue medication allows patients to receive effective treatment, e.g. where a standard treatment is available.

- **Challenge agents**

Challenge agents are usually given to study subjects to produce a physiological response that is necessary before the pharmacological action of the IMP can be assessed.

- **Concomitant medicinal products systematically prescribed to the study patients**

This type of NIMP is given to clinical study participants as required in the protocol as part of their standard care for a condition which is not the indication for which the IMP is being tested, and is therefore not the object of the study.

- **Background treatment**

This type of medicinal product is administered to each of the clinical study subjects, regardless of randomization arm, to treat the indication which is the object of the study. Background treatment is generally considered to be the current standard care for the particular indication. In these studies, the IMP is given in addition to the background treatment and safety and efficacy are assessed. The protocol may require that the IMP plus the background treatment is compared to an active comparator or to placebo plus background treatment.

Appendix 2: Reporting Procedures

Reporting procedure:

All IRAE information has to be recorded in the IRAE/SAE form and reported to PPD Corporate Drug Safety (CDS) immediately (i. e. within 24 hours) by e-mail or fax (see addresses below in the box) after the investigational site becoming aware of the IRAE.

In addition, this IRAE has to be recorded on the AE page of the eCRF and the following eCRF pages have to be updated or completed at the same time as necessary: Study drug documentation, subject demographics, medical history, concomitant medication, and study completion/termination (in case of an AE leading to withdrawal).

Entry of an IRAE/SAE into the eCRF will trigger an alert message to PPD and Biotest CDS.

PPD	[redacted]
[redacted]	[redacted]
[redacted]	[redacted]
[redacted]	[redacted]

For questions regarding IRAEs including SAEs or to notify the sponsor of an IRAE including SAE in the event of technical failure of the e-mail or fax system, the investigator should contact PPD [redacted]

Whenever follow-up information becomes available to a previously recorded IRAE/SAE, this has to be captured in the IRAE/SAE form and should be send by e-mail or fax together with any supporting documents (e.g. medical records, autopsy report, ECG or laboratory reports) as part of the follow-up information accompanied by a cover page within max. 24 hours of the investigator becoming aware of the follow-up information to the reporting contact above.

In addition, the new information should be captured in the eCRF on the AE page within max. 24 hours after becoming aware of the follow-up information.

The investigator has to undertake active follow-up for subjects with IRAE/SAEs. The investigator shall respond to queries raised by the sponsor with regard to IRAE/SAEs within the timelines stipulated in the query, and provide all necessary information as requested. In case of a fatal or life-threatening SAE, the sponsor will contact the investigator urgently to obtain required additional information within one business day. If supporting documents are requested by the sponsor (e.g. copies of medical records, laboratory reports, ECG tracings, autopsy report), the investigator must ensure that subject identifying data are obliterated prior to sending to the sponsor. The supporting documents should carry the subject ID for identification.

If required the investigator is responsible to inform local IECs/IRBs of safety reports in compliance with applicable regulatory requirements. Copies of all correspondence

relating to reporting of safety reports to IEC/IRB should be maintained in the Investigator Site File/ Regulatory Binder.

The sponsor is responsible for fulfilling all obligations regarding notification of regulatory authorities, ethics committees according to applicable regulatory requirements, in regard to expedited reporting (e.g. serious unexpected suspected adverse reactions) and periodic reporting (e.g. development Safety Update Report). In addition, the sponsor is responsible for information of investigators according to the current legislation.

Document S2: Clinical study protocol for study sites in the UK—Study 995 CSP version 4.3 (995_CSP_V4.3_UK_06-Sep-2021_redacted)

Biotest
From Nature for Life

Clinical Study Protocol

Title: A randomized, active-controlled, multicenter, phase III study investigating efficacy and safety of intra-operative use of BT524 (human fibrinogen concentrate) in subjects undergoing major spinal or abdominal surgery (AdFIrst)

Short Title: AdFIrst - Adjusted **Fi**brinogen **r**eplacement **st**rategy

**Clinical Phase:**	III
**Version incl. date:**	Final 4.3 of 06-SEP-2021<br>Country specific amendment for United Kingdom
**EudraCT Number:**	2017-001163-20
**Study No.:**	995

Sponsor PPD

PPD

Coordinating Investigator PPD

Confidentiality Statement

This protocol is property of PPD*, and may not be circulated, reproduced or published - either in whole or in part - without the company's written permission.*

Overview of Amendments integrated in the protocol text of Version 4.1 of 04-MAR-2020

Amendment No / CSP Version	Date	Sections concerned	Rationale
1 / CSP V2.0	26-Apr-2018	Signature Page,<br>Synopsis, 2, 3, 4, 6, 7, 8, 9, 10	Introduction of a new Biostatistician.<br>Update with clarification of wording, corrections and formatting.<br>Revision of the protocol to eliminate inconsistencies between different protocol sections.
1 / CSP V2.0	26-Apr-2018	Flowchart, 3, 7, 9	Clarification regarding the time-period between screening and baseline and the need of repeated assessments and diagnostic tests at these visits.
1 / CSP V2.0	26-Apr-2018	Synopsis, 4	Incomplete exclusion criterion 4 was complemented.
1 / CSP V2.0	26-Apr-2018	3	Clarification of treatment algorithm.
1 / CSP V2.0	26-Apr-2018	6	Clarification of treatment algorithm resulted in a revised definition for prohibited medication (section 6.10).<br>Restructuring of section 6.11 'Warnings and Precautions'.
1 / CSP V2.0	26-Apr-2018	9, 11	Update and clarification regarding the handling of laboratory samples and the assessment of results.
2 / CSP V3.0	07-Jun-2019	Signature page, Introduction, 4	Introduction of a new Biostatistician PPD .<br>Update with clarification of wording, corrections and formatting.
2 / CSP V3.0	07-Jun-2019	Synopsis, Flowchart, 3, 4, 7, 9, 10	Intra-operative inclusion criterion was adapted. Based on patients' bodyweight and clinical condition, the clinical need of FFP transfusion during surgery can already occur after a clinically relevant bleeding of approximately 1 liter.<br>Instead of measurement and calculation of blood loss prior to the 'decision to treat', in case of a clinically relevant bleeding an estimation of blood loss will take place.
2 / CSP V3.0	07-Jun-2019	4.2	Inclusion criterion 4 aims to ensure that only patients without hereditary bleeding disorders are to be included in this study. Therefore, a footnote for clarification of wording was included.
2 / CSP V3.0	07-Jun-2019	Synopsis, Flowchart, 3, 6, 7, 9	The dosage was adapted in order to avoid under-dosing of subjects. The first BT524 treatment was changed to a minimum dose of 2 g. The option for repeated dosing with IMP was included. The wording of the

Amendment No / CSP Version	Date	Sections concerned	Rationale
			secondary endpoints was adapted accordingly.<br>The dose justification was updated.
2 / CSP V3.0	07-Jun-2019	Flowchart, 7	The time points ‘90 minutes after treatment start’ and ‘end of the surgery’ can be close together. In this case, blood samples do not have to be taken at both times. Update with clarification of wording. The option for repeated dosing with IMP was included.
2 / CSP V3.0	07-Jun-2019	7	Clarification of wording regarding the re-screening of subjects.
2 / CSP V3.0	07-Jun-2019	9.3, Appendix 2	Update and clarification regarding the definition of Adverse Events of Special Interest (AESI) and the respective reporting procedures.
3 / CSP V4.0	04-Dec-2019	Cover Page, Signature page, 1, 9	Introduction of a new Biostatistician PPD .<br>General update with clarification of wording, corrections and formatting.
3 / CSP V4.0	04-Dec-2019	Synopsis, Flowchart 2, 3, 5, 7.2, 8.1	Study synopsis, study objectives and study design have been modified to extend the target population of the study to allow inclusion of subjects undergoing pseudomyxoma peritonei surgery (only applicable in the United Kingdom) and to introduce the active comparator cryoprecipitate in this treatment group.
3 / CSP V4.0	04-Dec-2019	4.1 and 4.2, 6.6	Update in order to take particular account of subjects with pseudomyxoma peritonei.
3 / CSP V4.0	04-Dec-2019	Synopsis, 3, 4.2, 6.1, 6.6, 7	Clarification of wording of the intra-operative inclusion criterion.
3 / CSP V4.0	04-Dec-2019	Synopsis, 10	Update with clarification of wording and revision to include interim analyses.<br>Following the Data Monitoring, the assumed standard deviation has been adjusted and the power has been reduced.
3 / CSP V4.0	04-Dec-2019	19	Update of scientific literature mainly regarding the inclusion of subjects with pseudomyxoma peritonei.
4 / CSP V4.1	04-Mar-2020	Cover Page, Signature page	Introduction of a new Biostatistician PPD .
4 / CSP V4.1	04-Mar-2020	Synopsis, Flowchart, 3, 4, 5, 6, 7.1, 9, 10.9	Study synopsis, study design and study treatment have been modified to describe in detail the treatment of subjects undergoing pseudomyxoma peritonei surgery in the United Kingdom.

Amendment No / CSP Version	Date	Sections concerned	Rationale
			General update with clarification of wording, corrections and formatting.
4 / CSP V4.1	04-Mar-2020	Study Population 4.1	Update in order to take particular account of subjects with pseudomyxoma peritonei.
4 / CSP V4.1	04-Mar-2020	IMP 5.3	Detailed description of the active comparator cryoprecipitate.
4 / CSP V4.1	04-Mar-2020	Methods for Assessing Primary Efficacy Parameter, 9.1.2	Adaption of process for quantification of blood loss due to cytoreductive PMP surgery.
4 / CSP V4.1	04-Mar-2020	Laboratory Parameters, 9.2.1, Table 1	Introduction of two additional laboratory parameters to be analysed in PMP subjects, update of Table 1.
4 / CSP V4.1	04-Mar-2020	Assessment of Adverse Events, 9.3.4	Update of section “Responsibilities of Sponsor” to clearly specify where to find the Reference Safety Information of both, BT524 and cryoprecipitate.
5 / CSP V4.2	04-Aug-2020	Synopsis,<br>2, 3, 4.1, 4.2, 5.2, 6.6, 7.2, 9.2.1.4, 10.3	General update to clarify that in the United Kingdom (UK) only one site will be involved and only subjects undergoing cytoreductive surgery for PMP can be included.<br>Removal of information regarding the treatment of subjects undergoing spinal surgery.
5 / CSP V4.2	04-Aug-2020	Procedures for Emergency Unblinding, 6.7	Clarification of wording to emphasize that the investigator has the final decision and unilateral right for unblinding.
5 / CSP V4.2	04-Aug-2020	Assessment of Adverse Events, 9.3.4	Update of section “Responsibilities of Sponsor” to change the Reference Safety Information (RSI) for cryoprecipitate.<br>Reference to the new Reference Safety Information for cryoprecipitate (section 4.8 of the SmPC of PPD solution for infusion).
5 / CSP V4.2	04-Aug-2020	Pregnancy, 9.3.8.1<br>(Gender Distribution 4.1.1)	Adding of a list of reliable birth control methods.
6 / CSP V4.3	06-Sep-2021	III Flowchart	Reintroduction of urinalysis on follow-up day 5 in the flowchart as this item was deleted in the CSP V3.0 by mistake.<br>Adaption of footnotes:<br>• Option for rescheduling tests and procedures that require an in-person visit from Day 36 (closing visit) to the day of discharge from hospital.<br>• Inclusion of missing footnote for coagulation factors on day 1, end of surgery.

Amendment No / CSP Version	Date	Sections concerned	Rationale
6 / CSP V4.3	06-Sep-2021	7.1 Visit Schedule for PMP subjects<br>7.2 Duration of the Clinical Study	Option for using a phone call instead of an in-person closing visit is depicted.<br>Option for rescheduling tests and procedures that require an in-person visit from Day 36 (closing visit) to the day of discharge from hospital is depicted.
6 / CSP V4.3	06-Sep-2021	9.2.1.4 Laboratory Parameters	Clarification regarding increased laboratory parameter values (liver function tests) and the documentation as adverse events.
6 / CSP V4.3	06-Sep-2021	3, 5.2.1, 7.1, 9.1.2, 10.1	Editorial changes.

I. SIGNATURE PAGE

This clinical study is carried out in accordance with the international guidelines on Good Clinical Practice (ICH-GCP) and in compliance with applicable regulatory authority requirements. It is confirmed that the clinical study will be carried out and documented in accordance with this study protocol.

PPD [redacted]

I.I Signature Page for Investigators

Declaration of the Principal Investigator

I have read and understood this Clinical Study Protocol and agree to the following:

- To adhere to the ethical and scientific principles of Good Clinical Practice, and the principles of the Declaration of Helsinki, the local laws and regulations, and the applicable regulatory requirements.
- To conduct the clinical study as set out in the protocol.
  This includes:
  - To wait until I have received approval from the appropriate Independent Ethics Committee / Institutional Review Board (IEC/IRB) before enrolling any subject in this study.
  - To obtain informed consent for all subjects prior to any study-related measure performed.
  - To permit study-related monitoring, audits, IEC/IRB review, and regulatory authority inspections.
  - To provide direct access to all study-related records, source documents, and subject files for the monitor, auditor, IEC/IRB, or regulatory authority upon request.
  - To use the IMP and all study materials only within the framework of this Clinical Study Protocol.
  - To understand that changes to the Clinical Study Protocol must be made in the form of an amendment that has the prior written approval of Biotest and, as applicable, of the appropriate IEC/IRB and regulatory authority.
  - To comply with the reporting obligations for all Adverse Events

I understand that all documentation that has not been previously published will be kept in the strictest confidence. This documentation includes the Clinical Study Protocol, Investigator's Brochure, Case Report Forms, and other scientific data.

Principal Investigator
Name

Date, signature

Investigator stamp:

Please insert stamp of investigational site

II. STUDY SYNOPSIS

**Title**	A randomized, active-controlled, multicenter, phase III study investigating efficacy and safety of intra-operative use of BT524 (human fibrinogen concentrate) in subjects undergoing major spinal or abdominal surgery (AdFIrst)
**Clinical Phase**	III
**Coordinating Investigator**	PPD
**Study Objectives**	The main purpose of this phase III study is to demonstrate the efficacy of BT524 as a complementary therapy to management of uncontrolled severe hemorrhage in subjects undergoing elective major spinal or abdominal surgery.<br>The **primary objective** of this study is to demonstrate that BT524 is non-inferior that means not worse than fresh frozen plasma (FFP)/cryoprecipitate with a non-inferiority margin of 150 mL in reducing intra-operative blood loss by intravenous (IV) administration in subjects with acquired hypofibrinogenaemia undergoing elective major spinal or abdominal surgery.<br>If therapeutic equivalence (non-inferiority) has been demonstrated, therapeutic superiority of BT524 compared with FFP/cryoprecipitate will also be assessed.<br>**Secondary objectives** are to demonstrate the efficacy of BT524 by assessing the correction of the fibrinogen level intra-operatively, the transfusion requirements, post-operative blood loss in the first 24 hours, the number of subjects with rebleeds, the hospital length of stay and the in-hospital mortality. Secondary objectives also comprise the safety of BT524 by documenting the number of adverse events (AE) including changes in laboratory parameters, the viral status, and the frequency and severity of thrombosis and of thromboembolic events (TEEs).
**Study Design**	Prospective, randomized, active-controlled, multicenter, non-inferiority study
**Study Population**	*In the United Kingdom:*<br>Adult subjects (≥ 18 years) of both gender undergoing elective abdominal surgery with expected major blood loss
**Inclusion Criteria**	**<u>At screening:</u>**<br>1. Written informed consent<br>2. Subjects scheduled for elective cytoreductive pseudomyxoma peritonei (PMP) surgery with expected major blood loss<br>3. Male or female, aged ≥ 18 years<br>4. No increased bleeding risk as assessed by standard coagulation tests and medical history

	5. **Intra-operative trigger for treatment:**[1]<br>Intra-operative prediction of clinically relevant bleeding of > 2 L, requiring hemostatic treatment during surgery.
**Exclusion Criteria**	1. Pregnancy or unreliable contraceptive measures or breast feeding (women only)<br>2. Hypersensitivity to proteins of human origin or known hypersensitivity reactions to components of the Investigational Medicinal Products (IMP)<br>3. Participation in another clinical study within 30 days before entering the study or during the study and/or previous participation in this study<br>4. Treatment with any fibrinogen concentrate and/or fibrinogen-containing product within 30 days prior to infusion of IMP<br>5. Employee or direct relative of an employee of the Contract Research Organization (CRO), the study site, or Biotest<br>6. Inability or lacking motivation to participate in the study<br>7. Medical condition, laboratory finding (e.g. clinically relevant biochemical or hematological findings outside the normal range), or physical exam finding that in the opinion of the investigator precludes participation<br>8. Presence or history of venous/arterial thrombosis or TEE in the preceding 6 months
**Number of Subjects**	100 evaluable subjects per treatment arm
**Countries / Number of Study Sites**	Multicenter, multinational, Europe / 15-20 sites<br>**EU and Switzerland:** Spine surgery, FFP as comparator<br>**United Kingdom** (one site)**:** PMP surgery, cryoprecipitate as comparator
**Investigational Medicinal Products (IMP)**	**BT524** (human fibrinogen concentrate) and **cryoprecipitate**<br>**BT524** is a heat-treated, lyophilized fibrinogen concentrate manufactured from human plasma.<br>BT524 is presented as a single-use vial containing 1 g of lyophilized fibrinogen. The lyophilisate is to be reconstituted with 50 mL of water for injections, resulting in a final concentration of 20 mg/mL for IV infusion.<br>FFP refers to the liquid portion of human blood that has been frozen and preserved after a blood donation and will be used for blood transfusion.<br>**Cryoprecipitate** is made from FFP which is frozen and repeatedly thawed in a laboratory to produce a source of concentrated clotting factors including fibrinogen, factor VIII, factor XIII, von Willebrand factor (vWF) and fibronectin and platelet microparticles.

[1] Approximately 60 minutes after the start of cytoreductive PMP surgery.

**Dosage and Mode of Administration**	**Subjects undergoing cytoreductive PMP surgery:**<br>**BT524,** administered intravenously:<br>Fixed first dose of 4 g, subsequent intra-operative infusions (4 g) as required.<br>**Cryoprecipitate,** administered intravenously:<br>Therapeutic dose is 2 pools, each pool consisting of 5 units (10 units, dose-equivalent to 4 g fibrinogen concentrate), subsequent intra-operative infusions of 2 pools as required.
**Duration of Treatment**	**Single or repeated intra-operative administration of IMP.**<br>*Follow-up per subject:*<br>Each subject will be followed for at least 5 weeks, with clinical and laboratory data collected on visits scheduled on Days 2, 3, 5 and 8, and the closing visit, scheduled on Day 36* after the day of surgery (*+35, up to Day 71 if required).
**Criteria for Evaluation**<br>***- Efficacy***	***Primary Endpoint:***<br>• Intra-operative blood loss after decision to treat the subject with IMP until the end of surgery as measured by amount of blood from the blood suction unit and amount of blood from swabs, surgical cloths and compresses.<br><br>***Secondary Endpoints:***<br>• Proportion (%) of subjects with successful correction of fibrinogen level 15 minutes after start of first IMP administration<br>• Time to first successful correction of fibrinogen level<br>• Total amount of transfusion products (allogenic blood products) or autologous blood transfusion infused after start of first IMP administration until end of surgery<br>• Amount of red blood cells (allogenic and autologous RBCs) infused after start of first IMP administration until end of surgery<br>• Post-operative blood loss in the first 24 hours<br>• Proportion (%) of subjects with rebleeds after the end of surgery until Day 8<br>• Hospital length of stay after surgery<br>• In-hospital mortality
- Safety	***Secondary Endpoints:***<br>• AEs<br>• Changes in vital signs<br>• Changes in clinical laboratory assessments of hematology, clinical chemistry, and urinalysis<br>• Changes in clinical laboratory assessments of markers of coagulation

	• Changes in clinical laboratory assessments of coagulation factors<br>• Frequency and severity of thrombosis and of TEEs<br>• Viral status
**Biostatistical Concept**	Assuming a blood loss of about 500 mL in the FFP[2]-/ cryoprecipitate-treatment arm after the decision to treat the subject with IMP until the end of surgery, a standard deviation of 375 mL, a non-inferiority margin of 150 mL, and an alpha-level of 2.5% (1-sided) 100 evaluable subjects per treatment arm are needed to demonstrate the non-inferiority of BT524 by using a t-test with 80% power. The sample size was calculated with nQuery Advisor Version 4.0 or higher.<br>***Primary Endpoint:***<br>The primary endpoint is intra-operative blood loss after the decision to treat the subject with IMP until the end of surgery. The primary analysis of this endpoint will test for non-inferiority. The final analysis will be performed using analysis of covariance (ANCOVA) with the predictive blood loss (> 1,000 mL to ≤ 2,000 mL and > 2,000 mL) as a covariate. Non-inferiority will be demonstrated if the upper confidence limit of the 2-sided 95% confidence interval for the difference in the least square means is less than the non-inferiority margin (150 mL). If non-inferiority is demonstrated, then superiority will be assessed.<br>***Interim analyses:***<br>In this study, 3 interim analyses with an alpha-adjustment according to Haybittle/Peto (Haybittle, 1971; Peto et al., 1976; Schulz and Grimes, 2005) are planned. This leads to local alpha levels of 0.001 for each interim analysis, a significance level of 0.05 for the final analysis, and to an overall global alpha level of 0.05.<br>All interim analyses will be based on the per-prototocl set.<br>The first interim analysis is planned with approximately 50 spine subjects, the second one with at least 40 PMP subjects and all other evaluable spine subjects at that time-point. The third interim analysis is planned with approximately 80% of subjects of the total sample size.<br>Aim of all interim analyses is to adapt the sample size according to the observed blood losses and the standard deviations:<br>a.) Early termination due to non-inferiority of BT524 in comparison with the used standard therapies.<br>b.) Continuation with the sample size as initially planned.<br>c.) Adjustment of sample size to take into account changes from the previous assumptions on the additional blood loss.

[2] FFP treatment is not applicable for subjects in the UK.

	d.) Stopping the study early due to futility if the sample size re-estimation indicates a much higher number than planned before.<br>***Secondary Endpoints:***<br>All secondary efficacy endpoints will be summarized descriptively by treatment arm.<br>The secondary endpoints of proportion of subjects with a successful correction of fibrinogen level and proportion of subjects with rebleeds will be compared between the treatment arms using a Cochran-Mantel-Haenszel (CMH) approach stratified by predictive blood loss.<br>The secondary endpoint 'time to first successful correction of fibrinogen level' will be compared between the treatment arms using a Chi-Square test.<br>The secondary endpoints of consumption of transfusion products, amount of RBCs and post-operative blood loss in the first 24 hours will be analysed using ANCOVA with the predictive blood loss as a covariate.<br>Safety will be assessed based on AEs, laboratory data, vital signs data, frequency and severity of thrombosis and of TEE and viral status which will be summarized descriptively.<br>***Data Safety Monitoring Board:***<br>An independent Data Safety Monitoring Board (DSMB) will review unblinded safety data at regular intervals during the study.<br>***Data Monitoring:***<br>After 40 subjects have completed, the overall mean and standard deviation of the primary endpoint will be derived using blinded data of all 40 subjects without separating according to treatment to assess if the sample size needs to be adjusted.
**First Subject In**	Q1 2018
**Last Subject Last Visit** *(planned)*	Tbd

Confidentiality Statement

This protocol is the property of Biotest AG, and may not be circulated, reproduced or published - either in whole or in part - without the company's written permission.

III. FLOWCHART OF STUDY

**Study Schedule** Day	**D-42 to D-1**	**D-2 to D1**	**D1**	**D1**	**D1**	**D1**	**D2**	**D3**	**D5**	**D8**	**D36** (+35)
**Assessments** Visit	**Screening**	**Prior surgery** (Baseline)	**Surgery**				**Follow-up**	**Follow-up**	**Follow-up**	**Follow-up**	**Closing**
			**prior 1st dose**	**pre-dose**	**post-dose**	**end of surgery**					
Informed consent	•										
Check/re-check of inclusion / exclusion criteria	•	•[1]									
Demographic data	•										
Classification of type of surgery	•										
Recording expected blood loss		•									
Body weight	•	•[2]									
Physical examination	•	•[2]					•	•	•	•	•[3]
Pregnancy test, only in females of childbearing potential	•	•[2]									
Medical and surgical history	•	•[4]									
Viral safety: Collection of retention sample	•										•[3]
Virus serology (hepatitis B, hepatitis C, HIV)	•										•[3]
Vital signs	•	•	•	•	•	•	•	•	•	•	•[3]
Hematology and clinical chemistry	•	•[2]	•			•	•	•	•	•	•[3]
Urinalysis	•	•[2]					•	•	•	•	•[3]
Markers of coagulation (coagulation activation tests)	•	•[2]	•		•[5]	•[6]	•	•	•	•	•[3]
Plasma concentration of fibrinogen activity (Clauss assay)	•	•[2]	•		•[5]	•[6]	•				
FIBTEM A10 (ROTEM)	•	•[2]	•	•	•[5]	•[6]	•				
Maximum clot firmness (MCF) (ROTEM)	•	•[2]	•	•	•[5]	•[6]	•				

[1] Diagnostic tests will be repeated at the investigator's discretion.
[2] Diagnostic tests have to be done prior to surgery. In case of short time-period between screening and baseline (≤ 2 days) these tests have only to be repeated based on medical judgment of the investigator. If not repeated, screening results will serve as baseline.
[3] Diagnostic tests are required as part of the closing visit. In case the closing visit is scheduled as a phone call, these tests can be performed on the day of hospital discharge.
[4] Previous medication: change from screening.
[5] Tests have to be done only 15 and 90 minutes after start of 1st IMP administration.
[6] Tests (*Markers of coagulation, Clauss assay, FIBTEM A10, MCF, coagulation factors*) have to be done '90 min after start of 1st IMP administration' and at the 'end of surgery'. In case of a short time-period between these two time points (<30 min) these tests have only to be repeated based on medical judgment of the investigator.

PPD

**Study Schedule** **Day**	**D-42 to D-1**	**D-2 to D1**	**D1**	**D1**	**D1**	**D1**	**D2**	**D3**	**D5**	**D8**	**D36** (+35)
**Assessments** **Visit**	**Screening**	**Prior surgery** (Baseline)	**Surgery**				**Follow-up**	**Follow-up**	**Follow-up**	**Follow-up**	**Closing**
			**prior 1st dose**	**pre-dose**	**post-dose**	**end of surgery**					
Coagulation factors (including vWF)		•	•		•[7]	•[6]					
Intra-operative inclusion criteria			•								
**Intravenous infusion(s) of IMP (BT524 or cryoprecipitate)**				•[8]							
Recording start/end of surgery			•			•					
Continuous measurement of blood loss from start of surgery				•		•	•				
Calculation and recording of blood loss						•[9]	•[10]				
Recording time of decision to treat the subject with IMP			•								
Order of IMP			•	•							
Randomization			•								
Rebleeding episodes						•	•	•	•	•	
Concomitant medication or treatment				•		•	•	•	•	•	•
Transfusion products				•		•	•	•	•	•	•
Adverse events	•	•		•		•	•	•	•	•	•

[7] Test has to be done only 90 minutes after start of 1st IMP administration.
[8] Total volume and total infusion time (start and end of each infusion) to be recorded.
[9] Intra-operative blood loss from time point of decision to treat the patient with IMP until end of surgery.
[10] Recording of blood loss (drainage volume) until 24 hours after end of surgery.

PPD

IV. TABLE OF CONTENTS

I. SIGNATURE PAGE 6

I.I Signature Page for Investigators 7

II. STUDY SYNOPSIS 8

III. FLOWCHART OF STUDY 13

IV. TABLE OF CONTENTS 15

V. LIST OF ABBREVIATIONS 19

1 INTRODUCTION 21

2 STUDY OBJECTIVES 24

3 STUDY DESIGN 25

4 STUDY POPULATION 30

4.1 Study Population, Diagnosis and Number of Subject 30
4.1.1 Gender Distribution 30

4.2 Inclusion Criteria 31

4.3 Exclusion Criteria 31

4.4 Subjects Withdrawal Criteria and Replacements 32

4.5 Subjects Information 33

4.6 Declaration of Informed Consent 33

5 INVESTIGATIONAL MEDICINAL PRODUCTS 34

5.1 Investigational Medicinal Product BT524 34
5.1.1 Description of Investigational Medicinal Product BT524 34
5.1.2 Formulation, Packaging and Labelling 34
5.1.3 Storage Conditions and Stability 35
5.1.4 Preparation for Use 35

5.2 Investigational Medicinal Product Cryoprecipitate 35
5.2.1 Description of Investigational Medicinal Product Cryoprecipitate 35
5.2.2 Formulation, Packaging and Labelling 35
5.2.3 Storage Conditions and Stability 36
5.2.4 Preparation for Use 36

6 STUDY TREATMENT 36

6.1 Dosage Regimen 36

6.2 Dosage and Administration 36

6.3 Compliance with Dosage Regimens 38

6.4 Dose Justification 38

6.5 Treatment of Overdose 39

6.6 Randomization Code 39

6.7 Procedures for Emergency Unblinding 40

6.8 Drug Accountability 40

6.9 Previous and Concomitant Medication or Treatment 40

6.10 Prohibited Medication or Treatment 41

6.11 Warnings and Precautions 41

7 COURSE OF THE CLINICAL STUDY 43

7.1 Visit Schedule for PMP subjects 43

7.2 Duration of the Clinical Study 51
7.2.1 End of Study 51

7.3 Criteria for Premature Termination 51
7.3.1 Premature Termination of the Entire Clinical Study 51
7.3.2 Premature Termination of an Individual Study Site 52

7.4 Treatment and Care after the End of the Study 52

8 BENEFIT-RISK EVALUATION 53

8.1 Benefit of BT524 53

8.2 Foreseeable Risk and Discomfort Related to BT524 53

8.3 Other Sources of Possible Risk and Discomfort 53

8.4 Summary of Possible Risk and Discomfort 53

9 ASSESSMENT OF OBJECTIVES / CRITERIA FOR EVALUATION 54

9.1 Efficacy 54
9.1.1 Specification of Efficacy Parameters 54
9.1.1.1 Primary Efficacy Parameter 54
9.1.1.2 Secondary Efficacy Parameter 54
9.1.2 Methods for Assessing and Recording Efficacy Parameters 55
9.1.2.1 Methods for Assessing Primary Efficacy Parameter 55
9.1.2.2 Methods for Assessing Secondary Efficacy Parameter 56
9.1.3 Specification of Efficacy Endpoints 58
9.1.3.1 Specification of Primary Efficacy Endpoint 58

9.1.3.2 Specification of Secondary Efficacy Endpoints 58

9.2 Safety 59
9.2.1 Specification of Safety Parameters 59
9.2.1.1 Adverse Events 59
9.2.1.2 Physical Examination....60
9.2.1.3 Vital Signs 60
9.2.1.4 Laboratory Parameters 61
9.2.1.5 Retention Samples 66
9.2.2 Methods for Assessing and Recording Safety Parameter(s)....67
9.2.3 Safety Endpoints 67

9.3 Adverse Events 67
9.3.1 Definitions....67
9.3.2 Recording Adverse Events 69
9.3.3 Period of Observation 70
9.3.4 Assessment of Adverse Events....71
9.3.5 Immediate Reporting by Investigator to Sponsor 72
9.3.6 Use of IMP outside the Specifications of the Clinical Study Protocol 72
9.3.7 Investigational Medicinal Product Complaints....73
9.3.8 Special Situations Requiring Immediate Reporting 73
9.3.8.1 Pregnancy 73

9.4 Data Safety Monitoring Board.... 74

10 STATISTICS.... 75

10.1 Analysis Sets.... 75

10.2 Protocol Deviations 75

10.3 General Considerations 76

10.4 Efficacy Analyses 76

10.5 Primary Efficacy Analysis 77

10.6 Secondary Efficacy Analyses 77
10.6.1 Correction of Fibrinogen Level....78
10.6.2 Consumption of Transfusion Products....78
10.6.3 Amount of Red Blood Cells....78
10.6.4 Post-operative Blood Loss 78
10.6.5 Proportion of Subjects with Rebleeds 79
10.6.6 Hospital Length of Stay after Surgery....79
10.6.7 In-hospital Mortality....79

10.7 Safety Analysis 79

10.8 Interim Analyses 79

10.9 Determination of Sample Size.... 80
10.9.1 Data Monitoring 81

11 DATA MANAGEMENT.... 81

11.1 Data Collection 81

11.2 Correction of Data 82

11.3 Data Handling 82

12 QUALITY CONTROL AND QUALITY ASSURANCE 82

12.1 Study Initiation Activities 82

12.2 Training of site staff 82

12.3 Documentation and Filing 83

12.4 Monitoring 83

12.5 Audits and Inspections 84

12.6 Archiving 84

13 GENERAL REGULATIONS, AGREEMENTS AND ORGANISATIONAL PROCEDURES 84

13.1 Study Administrative Structure 84

13.2 Ethical and Regulatory Considerations 84

13.3 Committees / Monitoring Boards 85

13.4 Written Agreements 85

13.5 Insurance/Liability 85

13.6 Investigator’s Brochure (IB) 85

13.7 Amendments to the Protocol 85

13.8 Confidentiality 85

13.9 Final Report and Publication 86

14 LIST OF REFERENCES 87

15 APPENDICES 94

Figure 1: Overview of Study Design 26
Figure 2: Quantification of Blood Loss Intra-operatively 27
Figure 3: Repeated IMP Administration 29
Figure 4: ROTEM Readout of Citrated Normal Blood 57

Table 1: Clinical Laboratory Parameters 62

V. LIST OF ABBREVIATIONS

ADR	Adverse Drug Reaction
AE	Adverse Event
AESI	Adverse Event of Special Interest
ALAT	Alanine aminotransferase
ANCOVA	Analysis of Covariance
AP	Alkaline phosphatase
aPTT	Activated partial thromboplastin time
ASAT	Aspartate aminotransferase
AT III	Antithrombin III
BDRM	Blind Data Review Meeting
BUN	Blood Urea Nitrogen
BW	Body Weight
CDS	Corporate Drug Safety
CHMP	Committee for Medicinal Products for Human Use
CMH	Cochran-Mantel-Haenszel
CSP	Clinical Study Protocol
CSR	Clinical Study Report
CRO	Contract Research Organization
DEVP	Drug Exposure Via Parent
DIC	Disseminated Intravascular Coagulation
DOAC	Direct Oral Anti-Coagulants
DSMB	Data Safety Monitoring Board
eCRF	electronic Case Report Form
EDC	Electronic Data Capture
EMA	European Medicines Agency
IEC	Ethics Committee
IRB	Institutional Review Board
FAS	Full Analysis Set
FII	Factor II
FV	Factor V
FVII	Factor VII
FVIII	Factor VIII
FIX	Factor IX
FX	Factor X
FXI	Factor XI
FXIII	Factor XIII
F_{1+2}	Prothrombin Fragments 1+2
FAS	Full Analysis Set
FFP	Fresh Frozen Plasma
GCP	Good Clinical Practice
γ-GT	Gamma glutamyltransferase
IB	Investigator’s Brochure
HAV	Hepatitis A Virus
HBV	Hepatitis B Virus
HCV	Hepatitis C Virus
HEV	Hepatitis E Virus

HIV	Human Immunodeficiency Virus
ICF	Informed Consent Form
IMP	Investigational Medicinal Product
ICH	International Conference on Harmonization
i.e.	id est
INR	International normalized ratio
IRAE(s)	Immediately Reportable Adverse Event(s)
IV	Intravenous
IWRS/IVRS	Interactive Web/Voice Response System
MCF	Maximum Clot Firmness
MedDRA®	Medical Dictionary for Regulatory Activities
NTEAE	Non-Treatment Emergent Adverse Event
NIMP	Non-Investigational Medicinal Product
PC	Protein C
PEI	Paul-Ehrlich-Institut, Germany
PHI	Protected Health Information
PPS	Per-Protocol Set
PS	Protein S
PMP	Pseudomyxoma peritonei
PT(INR)	Prothrombin Time (International Normalized Ratio)
QBL	Quantification of Blood Loss
RBC	Red Blood Cells
ROTEM	Rotational Thromboelastometry
SAE	Serious Adverse Event
SAF	Safety Analysis Set
SAP	Statistical Analysis Plan
SAS	Statistical Analysis Software
SmPC	Summary of Product Characteristics
SOC	System Organ Class
TAT	Thrombin-antithrombin III complex
TEAE	Treatment-Emergent Adverse Event
TEE	Thromboembolic Event
TGT	Thrombin Generation Test
TMF	Trial Master File
TT	Thrombin Time
TXA	Tranexamic Acid
vWF	von Willebrand factor
WBC	White Blood Cells

1 INTRODUCTION

BT524 is a lyophilized, heat-treated fibrinogen concentrate manufactured from human plasma. BT524 is currently developed for the treatment and prophylaxis of bleeding in patients with congenital afibrinogenaemia or severe congenital hypofibrinogenaemia with bleeding tendency. Moreover, BT524 will be developed as complementary therapy to management of uncontrolled severe hemorrhage in acquired hypofibrinogenaemia.

To date, no clinical studies have previously been conducted with BT524 in subjects with acquired hypofibrinogenaemia. The pharmacokinetic properties of BT524 were investigated in the treatment and prophylaxis of bleeding in patients with congenital fibrinogen deficiency (afibrinogenaemia or severe hypofibrinogenaemia) in the ongoing prospective, open-label, phase I/III study PPD. Details on the clinical pharmacology of BT524 and further information on non-clinical studies with BT524 are provided in the Investigator's Brochure.

In addition to the ongoing clinical development in congenital fibrinogen deficiency, the present prospective, multi-center, randomized, active-controlled, pivotal phase III study aims to demonstrate the efficacy and safety of BT524 in subjects with acquired hypofibrinogenaemia caused by major surgery associated with major blood loss.

Fibrinogen (coagulation factor I) is a soluble plasma glycoprotein synthesized by hepatic parenchymal cells. The normal blood fibrinogen concentration is between 2.0 and 4.5 g/L although this range can vary (Levy and Goodnough, 2015). Fibrinogen plays a central role by clot forming in wound healing and furthermore, is important in primary hemostasis as it contributes to blood platelet aggregation. In case of a fibrinogen deficiency the blood coagulation is disordered, which leads to (severe) hemorrhagic events.

Acquired fibrinogen deficiency is the most common type of fibrinogen deficiency. It is characterized by an impaired hemostatic function caused by fibrinogen concentrations below the normal ranges, classified as hypofibrinogenaemia. Hypofibrinogenaemia results from either reduced fibrinogen synthesis due to hepatic disorders, increased intravascular consumption due to a breakdown of fibrinogen, disseminated intravascular coagulation (DIC) or increased fibrinogen loss caused by certain medical conditions such as surgical procedures or uncontrolled life-threatening bleeding. Acquired fibrinogen deficiency can cause severe intra-operative bleeding. Depending on the severity and the extent of the event (i.e. trauma, surgery) and the patient's clinical condition fibrinogen plasma concentrations are highly variable in acquired fibrinogen deficiency.

Acquired fibrinogen deficiency is associated with increased morbidity and mortality. Thus, effective management of this hemostatic disorder is necessary to prevent potentially life-threatening bleeding, to reduce increased blood loss, transfusion requirements and the risk of surgery (Fenger-Eriksen et al., 2009).

Fibrinogen is an important contributor to clot strength and is the first coagulation factor to become critically reduced during intra-operative hemorrhage (Haas et al., 2012). Therefore, a rapid and accurate determination of fibrinogen level is important during hemorrhage to establish a timely hemostatic intervention. The rapid fibrinogen supplementation to restore plasma levels is an important component for normalizing clot formation in bleeding patients and maintaining fibrinogen levels is an important therapeutic target in bleeding, particular in intra-operative settings (Levy and Goodnough, 2015).

There is growing evidence that fibrinogen levels > 1.5 to 2 g/L are necessary to control major bleeding in the intra-operative settings (Haas et al., 2012; Levy and Goodnough, 2015). Accordingly, European trauma guidelines from 2013 (Spahn et al., 2013) and the guidelines from the European Society of Anaesthesiology (Kozek-Langenecker et al., 2013) recommend target levels of at least 1.5 to 2.0 g/L in intra-operative settings. Because of the large variability in fibrinogen concentrations among bleeding patients, individualized dosing of fibrinogen concentrate based upon both the level of bleeding and the plasma fibrinogen concentration are recommended (Levy et al., 2012). Fibrinogen concentrate infusion guided by point-of-care tests is recommended by the European Society of Anaesthesiology (Kozek-Langenecker et al., 2013) and the FIBTEM test has been used extensively in clinical studies to determine fibrinogen levels and calculate dosing (Levy and Goodnough, 2015).

Nevertheless, the optimal treatment level, the use of pre-emptive treatment and the preferred source of fibrinogen for acquired fibrinogen deficiency remain disputed. Fibrinogen concentrate is increasingly used and recommended for bleeding with acquired hemostatic deficiencies in several countries, but evidence is inconsistent regarding surgery settings, dosing and efficacy. Fresh frozen plasma (FFP) also contains fibrinogen and is available in all hospital settings and is comparatively cheap but requires the administration of large quantities of FFP to achieve a reasonable fibrinogen dose. Therefore, further clinical studies investigating fibrinogen replacement in acquired fibrinogen deficiency are needed.

Currently, conventional replacement therapy in fibrinogen deficiency consists of transfusion of allogenic blood products such as **FFP** and **cryoprecipitate.**

FFP is a blood product that has been available since 1941. Initially used as a volume expander, it is currently indicated for the management and prevention of bleeding in coagulopathic patients (Nascimento et al., 2010).

FFP is the liquid portion of blood that contains all the clotting factors, as well as other blood proteins and that is stored by freezing (WHO 2007). FFP is usually authorized nationally. Examples of FFP authorized in Germany are available from the website of the German authority Paul-Ehrlich-Institut (PEI). A solvent/detergent treated frozen plasma (PPD by PPD is nationally approved in several EU countries (PPD PPD).

Compared to fibrinogen concentrate FFP is stored frozen and must be thawed before use which is a limitation in time critical and potentially life-threatening situations such as severe bleeding. FFP contains 2.0 to 4.5 g/L of fibrinogen which is much lower than in fibrinogen concentrates (i.e. up to 10-fold lower than in BT524 with 20 g/L). Since FFP contains relatively low amounts of fibrinogen, it requires the administration of large volumes to provide meaningful increases in the fibrinogen plasma level. The large volumes carry the risk of hypervolemia, cardiac stress, circulatory overload, transfusion-related acute lung injury, hypothermia and metabolic complications once bleeding resolves (Bornikova et al., 2011; Elliott and Aledort, 2013; Mumford et al., 2014; Ofosu et al., 2008). Furthermore, the use of allogenic blood confers an additional risk for blood borne pathogens. Also noteworthy is the risk for transfusion related reactions, immune suppression, and a decrease in coagulation factors. There is also evidence that transfusion of allogenic blood is increasingly harmful as more blood is transfused (Verma et al., 2015).

As the concentration of fibrinogen in fibrinogen concentrates is markedly higher than in plasma the required dose to reach the fibrinogen target concentration can be administered in a minor volume. Furthermore, the amount of fibrinogen in FFP varies making it difficult to predict the increase in plasma fibrinogen concentration. The precisely defined fibrinogen content in fibrinogen concentrates allows accurate calculation of the amount of reconstituted concentrate needed for a targeted fibrinogen supplementation.

Cryoprecipitate is made from FFP and contains various proteins including fibrinogen. It contains higher concentrations of fibrinogen than FFP and some (but not all) coagulation factors, so less volume is needed. The minimum amount of fibrinogen required by standards of the American Association of Blood Banks is 150 mg per bag of cryoprecipitate; current preparations yield a median of 388 mg per bag, but the amount of fibrinogen is variable and cannot be determined accurately. A single unit of cryoprecipitate also contains variable amounts of FVIII, FXIII, von Willebrand factor (vWF), fibronectin and platelet microparticles (Callum et al., 2009). Therefore, the use of cryoprecipitate for fibrinogen replacement alone exposes the patient to potentially unneeded coagulation proteins which could increase the risk of thrombosis (Elliott and Aledort, 2013; Franchini and Lippi, 2012). Cryoprecipitate was withdrawn from most European countries some years ago because of safety concerns (Karkouti et al., 2018; Schochl et al., 2013) but remains available in Scandinavia, the UK and the USA, essentially for the purpose of fibrinogen replacement.

In summary fibrinogen concentrate seems to have certain advantages over other replacement therapies like FFP and cryoprecipitate, such as precisely determined high amounts of purified fibrinogen dissolved in a small volume, low risk of pathogen transmission and instant administration without need for thawing or testing AB0 blood group compatibility (Warmuth et al., 2012).

A number of clinical studies have been published in subjects with acquired hypofibrinogenaemia, such as following trauma, cardiothoracic surgery and obstetric hemorrhage, documenting that fibrinogen concentrate is able to improve clotting function and reduce blood loss. The results of these studies have shown that fibrinogen concentrate raises the levels of fibrinogen and improves clot firmness. Additionally, fibrinogen substitution has been reported to reduce bleeding and post-operative transfusion requirements. Published data from completed clinical studies in subjects undergoing major surgeries not only suggest that fibrinogen plays a critical role in achieving and maintaining hemostasis; in particular, they appear to show benefit of individualized dosing of fibrinogen concentrate using a target ROTEM/FIBTEM value integrating rapid diagnostic testing with appropriate therapeutic dosing of fibrinogen concentrate in line with patients' needs (Haas et al., 2015; Rahe-Meyer et al., 2013a; Rahe-Meyer et al., 2009a; Rahe-Meyer et al., 2013b; Rahe-Meyer et al., 2009b; Ranucci et al., 2015). In a prospective, randomized, single-center, controlled phase 2 study, published in 2019 PPD, 45 adult subjects undergoing cytoreductive surgery for pseudomyxoma peritonei (PMP) were treated pre-emptively with fibrinogen concentrate or cryoprecipitate. Subjects were randomized to one of the two treatment groups (4 g fibrinogen concentrate or 2 pools cryoprecipitate), when assessment after the start of surgery predicted intra-operative blood loss ≥ 2 L without targeted fibrinogen replacement. Further intra-operative doses were based on thromboelastometry (FIBTEM A20 < 12 mm). Hemostatic efficacy was successful in 100% of subjects in both groups, with similar blood loss. No thromboembolic events (TEEs) occurred in subjects who received fibrinogen concentrate.

The published data indicate that fibrinogen concentrate is at least comparable with cryoprecipitate in terms of benefits for hemostatic therapy in the treatment of clinically relevant bleeding associated with acquired fibrinogen deficiency in subjects undergoing cytoreductive surgery for PMP (Roy et al., 2020).

As there is no guideline on the clinical investigation of fibrinogen in patients with uncontrolled severe hemorrhage in acquired hypofibrinogenaemia available, the EMA guideline on core SmPC for human fibrinogen products (EMA (Committee for Medicinal Products for Human Use), 2015) has been taken into account when planning this phase III study in acquired fibrinogen deficiency. Furthermore, currently ongoing clinical studies with fibrinogen concentrate as well as recently finalized clinical studies in this clinical setting (Roy et al., 2020) have been considered.

BT524 will be developed as complementary therapy to management of uncontrolled severe hemorrhage in acquired hypofibrinogenaemia caused by major surgeries associated with major blood loss. As it is expected that BT524 will show a safety advantage over the standard treatment with FFP/cryoprecipitate, an efficacy comparison to the standard is required to allow a risk-benefit assessment to be made for BT524.

Therefore, the present prospective, multi-center, randomized, active-controlled, pivotal phase III non-inferiority study aims to investigate efficacy and safety of BT524 in subjects with acquired hypofibrinogenaemia.

2 STUDY OBJECTIVES

The main purpose of this phase III study is to demonstrate the efficacy of BT524 as a complementary therapy to management of uncontrolled severe hemorrhage in acquired hypofibrinogenaemia in subjects undergoing elective major spinal or abdominal surgery.

The primary objective of this study is to demonstrate that BT524 is non-inferior that means not worse than FFP/cryoprecipitate with a non-inferiority margin of 150 mL in reducing intra-operative blood loss by IV administration in subjects with acquired hypofibrinogenaemia undergoing elective major spinal or abdominal surgery.

If therapeutic equivalence (non-inferiority) has been demonstrated, therapeutic superiority of BT524 compared with FFP/cryoprecipitate will also be assessed.

Secondary objectives are to demonstrate the efficacy of BT524 by assessing the correction of the fibrinogen level intra-operatively, the transfusion requirements, post-operative blood loss in the first 24 hours, the number of subjects with rebleeds, hospital length of stay and in-hospital mortality. Secondary objectives also comprise the safety of BT524 by documenting the number of AEs including changes in laboratory parameters, the viral status, and the frequency and severity of thrombosis and of TEEs.

Since this is a **country specific clinical study protocol (CSP) version for subjects undergoing cytoreductive PMP surgery at one site in the UK only**, the treatment of subjects undergoing spinal surgery (with BT524 or FFP) is not described in this protocol version.

3 STUDY DESIGN

This is a phase III, prospective, randomized, active-controlled, multicenter, non-inferiority clinical study in subjects undergoing major spinal *(not applicable for subjects in the UK)* or abdominal surgery to demonstrate the efficacy and the safety of intra-operative use of BT524 as a complementary therapy to management of uncontrolled severe hemorrhage in acquired hypofibrinogenaemia.

This non-inferiority study is focused on the **primary objective**, to demonstrate that BT524 is non-inferior that means not worse than FFP/cryoprecipitate with a non-inferiority margin of 150 mL in reducing intra-operative blood loss by IV administration in subjects with acquired hypofibrinogenaemia undergoing elective major spinal or abdominal surgery. If therapeutic equivalence has been demonstrated, therapeutic superiority of BT524 compared with FFP/cryoprecipitate will also be assessed.

Secondary objectives are to demonstrate the efficacy of BT524 by assessing the correction of fibrinogen level during surgery, the transfusion requirements, post-operative blood loss in the first 24 hours, the number of subjects with rebleeds, the hospital length of stay and the in-hospital mortality. Secondary objectives also comprise the safety of BT524 by documenting the number of AEs including changes in laboratory parameters, the viral status, and the frequency and severity of thrombosis and of TEEs.

The study comprises a screening visit within 42 days prior to surgery to assess subjects eligibility, a baseline visit on the day of the surgery prior anaesthesia (Day 1, but if required this could be Day -2 or Day -1 due to local hospital procedures), the surgery phase (including randomization, Day 1) and the follow-up phase of at least 5 weeks with 4 follow-up visits on Days 2, 3, 5 and 8 and the closing visit, including the final safety examination, and a safety follow-up scheduled on Day 36* after the day of surgery (*+35, up to Day 71 if required). The duration of individual study participation for eligible screened subjects is at least 5 weeks.

Since **this is a country specific clinical study protocol (CSP) version for subjects undergoing cytoreductive PMP surgery only**, the study design for subjects undergoing spinal surgery is not outlined here.

The **study design for subjects undergoing cytoreductive PMP surgery is shown in the following figure** (Figure 1):

Figure 1: Overview of Study Design

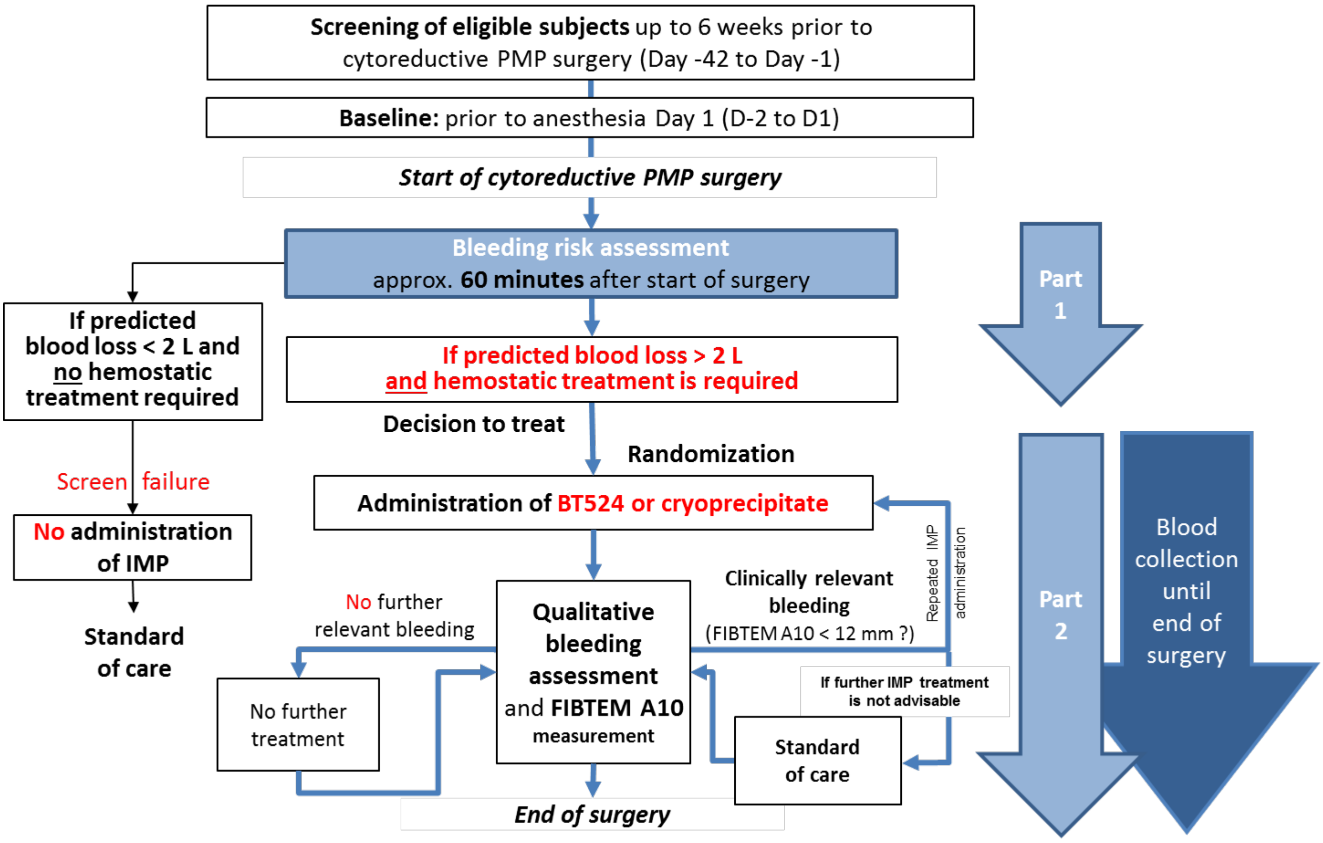

Further details on the assessment schedule (including the follow-up period) that will be used for the assessment of the efficacy and safety parameters in this study are presented in the flowchart in section III and in the visit schedule section 7.1.

At least 200 subjects will be enrolled to ensure data are available for 100 evaluable subjects per treatment arm (BT524 or FFP[1]/cryoprecipitate). The multicenter, multinational study will be conducted at approximately 15-20 sites in the EU and Switzerland with subjects undergoing major spinal surgery, **and at one site in the United Kingdom with subjects undergoing cytoreductive PMP surgery and treated with BT524 and cryoprecipitate.**

Detailed Description of the **PMP Surgery Phase**

The continuous determination/measurement of blood loss during the entire surgery will be separated into two parts:

- **First part:** blood loss will be determined from start of surgery until decision to treat by **estimating** the bleeding mass in the blood suction unit, taking the blood loss in swabs, surgical cloths and compresses into account. This is the prerequisite for the intra-operative inclusion criterion (intra-operative prediction of clinically relevant bleeding of > 2 L, requiring hemostatic treatment during surgery) and the decision to treat the subject with IMP.
- **Second part:** blood loss will be **measured** after decision to treat until end of surgery and represents the blood loss considered for the primary objective. During

[1] FFP treatment is not applicable for subjects in the UK.

this part of the surgery the blood loss will be quantified by measuring the continuous bleeding mass removed from the surgical field by a blood suction unit and by calculation of the amount of blood absorbed by swabs, surgical cloths and compresses. In a final step, the total blood loss will be calculated.

Prior to the start of surgery the weight of dry swabs, surgical cloths and compresses will be recorded (weighing of swabs is required if weight is not specified on the packaging).

The **first part** of blood measurement starts at the start of surgery with the collection of blood in the blood suction unit: The blood is salvaged by a suction catheter from the operating field. In case a manual compression is necessary, dry swabs, surgical cloths and compresses will be applied to the surgical field, and - at the latest - prior to the decision to treat the subject with IMP removed.

The anaesthesiologist will estimate the blood volume collected in the suction container continuously, taking the blood loss in swabs, surgical cloths and compresses into account. Allowance must also be made for the presence of mucinous ascites and washout. During the surgery, the anaesthesiologist must calculate these volumes suctioned into the container. The anaesthesiologist subtracts these amounts **to estimate the volume of blood in the container.**

The quantification of blood loss (QBL) intra-operatively is shown in Figure 2:

Figure 2: Quantification of Blood Loss Intra-operatively

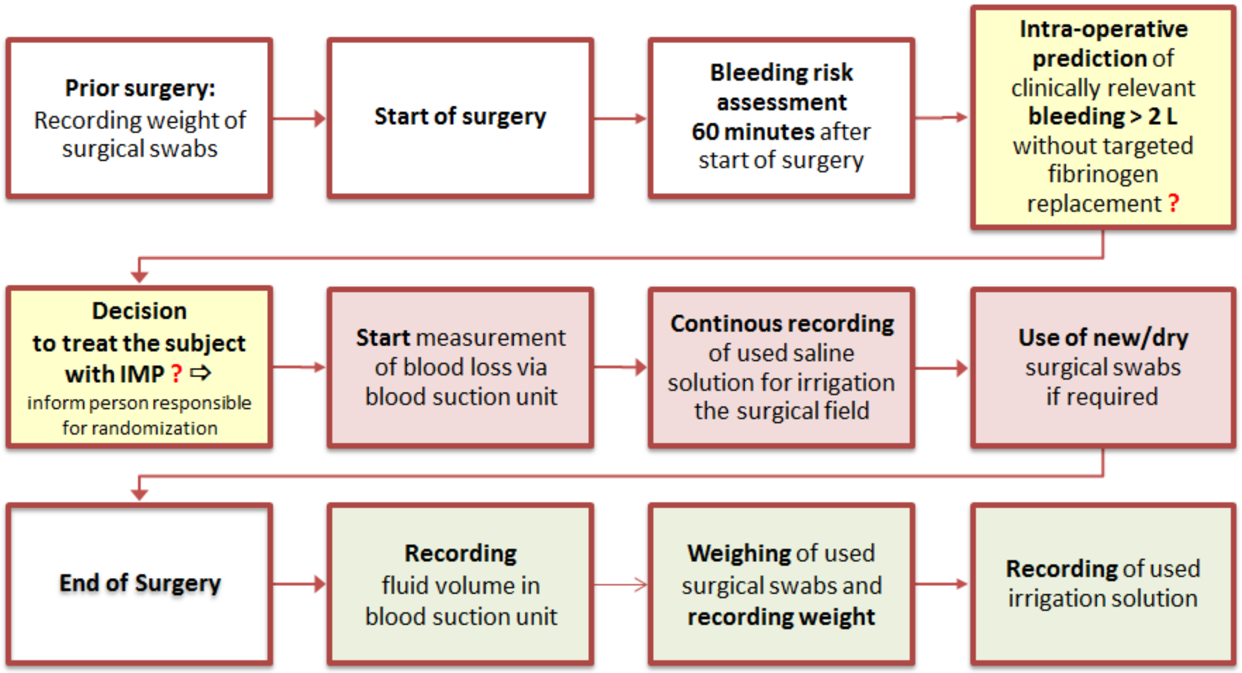

After opening and inspection of the abdomen the inside of the abdominal cavity will be washed out with a certain amount of irrigation solution (physiological saline solution) to remove mucinous ascites. Afterwards, approximately 60 minutes after the start of cytoreductive PMP surgery, the subject's intra-operative bleeding risk has to be assessed by the surgeon and the anaesthesiologist. In case of a predicted clinically relevant blood

loss of more than 2 L, and the assessment that hemostatic treatment will be required during surgery (high risk for the need of fibrinogen supplementation, either with cryoprecipitate or BT524), the decision to treat the subject with IMP can be made.

At this point the **time has to be documented**.

Immediately after the prediction of clinically relevant bleeding of > 2 L, requiring hemostatic treatment during surgery, the person responsible for randomization will be informed about the decision to treat the subject with IMP. The subject will be randomized to one of the treatment arms.

At the same time, the **suction container will be emptied** and all remaining swabs, surgical cloths and compresses will be removed from the surgical field.

The **second part of blood measurement** is initiated with collection of blood in the empty blood suction unit. In addition, in case a manual compression is necessary after decision to treat the subject with IMP, new swabs, surgical cloths and compresses will be applied to the surgical field.

The first dose of BT524 (4 g) or cryoprecipitate (2 pools, each pool consisting of 5 units/donations, dose-equivalent to 4 g fibrinogen concentrate) will be administered pre-emptively, based on clinical judgement (prediction of clinically relevant bleeding of more than 2 L intra-operatively).

IMP will be delivered to the operating room by the blood bank (cryoprecipitate) or by the pharmacy (BT524) according to local standards, and administered by the unblinded anaesthesiologist.

The content of each syringe with BT524 (1 g fibrinogen concentrate dissolved in 50 mL water for injection) can be administered in less than 20 seconds. Cryoprecipitate can be infused as rapidly as possible by the anaesthesiologist.

In the further course of the surgery, qualitative assessment of bleeding will be used by the surgeon to estimate if further bleeding intervention is required. Surgeon and surgical staff are blinded to therapy and will inform the anaesthesiologist regarding the bleeding assessment. In addition, FIBTEM A10 should be determined in order to guide further IMP doses. In case of a further intra-operative clinically relevant bleeding, a FIBTEM A10 < 12 mm (known or presumed hypofibrinogenaemia) may trigger the administration of IMP. If no further relevant bleeding occurs, the surgery will proceed to completion.

If the bleeding remained unchanged and an ongoing relevant blood loss will be confirmed or the hemostatic control is not considered sufficient and requires further intervention or a new major blood loss occurred in the course of the surgery, subjects can be treated with repeated IMP administration according to their randomized treatment group. If further IMP treatment is not advisable a transfusion protocol according to local standards (standard of care) should be followed (at the discretion of the surgeon and anesthesiologist).

At the end of the surgery, all swabs, surgical cloths and compresses will be removed from the surgical field, wrung out (blood will be collected in the suction container) until almost dry and weighed (weight will be recorded). The end of surgery is defined as time of last suture.

The fluid volume collected in the blood suction container will be measured and recorded, the proportion of blood, and irrigation solution will be calculated and also recorded.

Standardized Transfusion Algorithm

Tranexamic Acid (TXA) can be administered prophylactically in all subjects according to local standards, e.g. TXA 1.5 g at the start of surgery as a single IV infusion, followed by a further dose of 1 g TXA 4 hours after first dose, if required (in case the surgery is still ongoing).

Repeated IMP Administration

According to the study design and the described treatment algorithm BT524 or cryoprecipitate will be administered pre-emptively as the primary hemostatic therapy if clinical judgement approximately 60 minutes after the start of cytoreductive PMP surgery predicts a clinically relevant bleeding of > 2 L intra-operatively, requiring hemostatic treatment during surgery.

Repeated intra-operative administration of IMP is possible depending on the subjects' clinical condition and their individual FIBTEM A10 results (Figure 3).

If bleeding continued after completion of IMP administration, and further IMP treatment is not advisable a **transfusion protocol according to local standards** (standard of care) should be followed.

Figure 3: Repeated IMP Administration

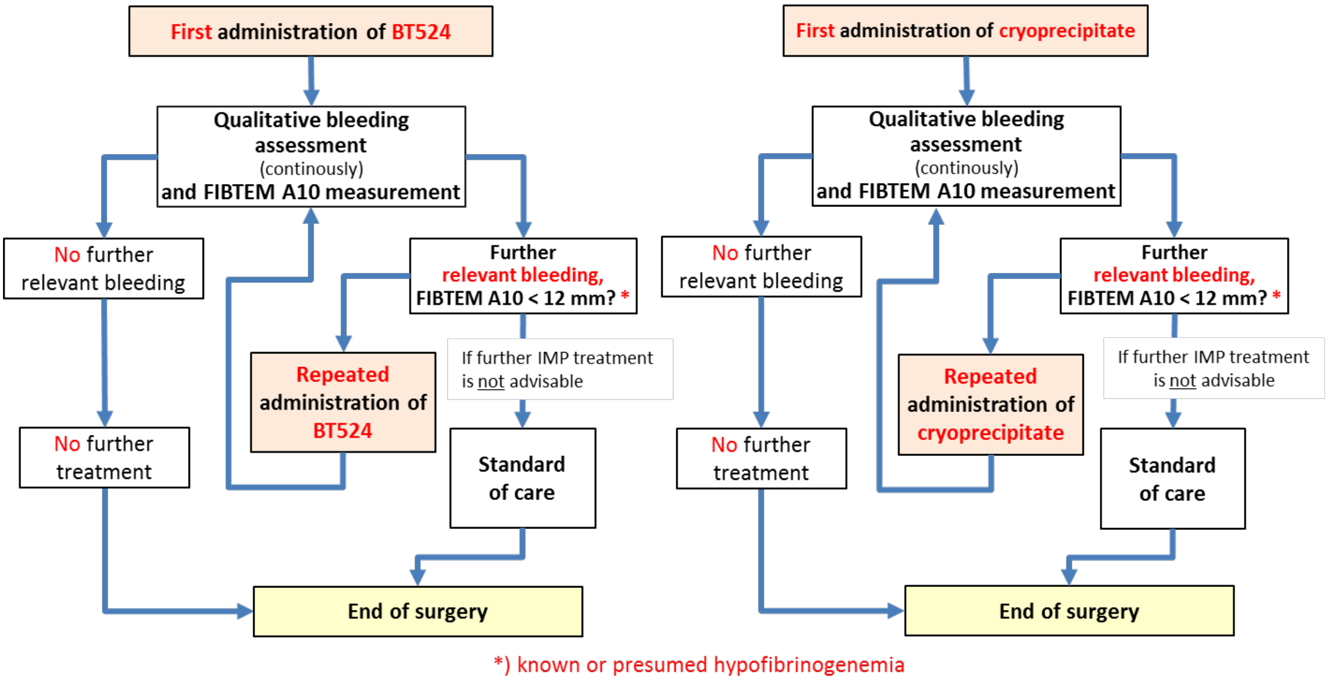

The decision to transfuse allogeneic blood products in the intra-operative period depends on the concentration of hemoglobin, the amount and speed of the blood loss and the clinical condition of the subject at the discretion of the surgeon and/or the anaesthesiologist.

The allogeneic blood components that can be used are RBCs, platelet concentrates, cryoprecipitate and FFP.

Furthermore, plasma-derived drugs (e.g. albumin) can be used according to local standards. The total volume (number of units and volume per unit) of all blood

components (e.g. RBCs, platelets, cryoprecipitate, FFP) or plasma derivatives (such as albumin and coagulation factors) given intra-operatively will be recorded.

4 STUDY POPULATION

4.1 Study Population, Diagnosis and Number of Subject

A total of at least 200 adult subjects (≥ 18 years) of both gender undergoing elective spinal (not applicable for subjects in the UK) or abdominal surgery with expected major blood loss are planned to be treated within this study. Eligibility is defined by the inclusion and exclusion criteria as described below.

In the UK the type of surgery is restricted by the eligibility criteria to cytoreductive surgery for PMP with an expected major blood loss.

Pseudomyxoma peritonei (PMP) is a rare clinically entity that usually results from a perforated primary appendiceal tumor. The sequence of events culminating in PMP is thought to involve growth of an appendiceal adenoma with distension of the appendix by mucus and mucinous tumor cells. The appendix eventually ruptures and mucinous deposits develop throughout the abdomen. The established intervention for PMP is cytoreductive surgery with hyperthermic intraperitoneal chemotherapy (Sugarbaker procedure) (Mohamed and Moran, 2011). Clinically relevant bleeding and acquired fibrinogen deficiency are common complications of this surgery. Patients undergoing the PMP surgery may develop a coagulopathy, predominantly characterised by reduced levels of fibrinogen, resulting in reduced whole blood clot firmness (Sargant et al., 2016).

4.1.1 Gender Distribution

There are no gender-based enrolment restrictions applicable for the study i.e., male and female subjects are intended to be included.

Since men and women undergoing elective surgery might suffer from acquired hypofibrinogenaemia, subjects of both genders should be included into the study. Equitable inclusion of both genders in research is important to ensure that both receive a proportionate share of benefits of research and that neither bears a disproportionate burden.

Women of childbearing potential are allowed to participate when using reliable/ effective contraceptive method(s) during the study and at least one month after the last administration of study drug (see section 9.3.8.1). However, pregnant women are to be excluded (see general exclusion criterion 1).

4.2 Inclusion Criteria

Only subjects meeting all of the following inclusion criteria will be considered for study inclusion:

Inclusion Criteria	Rationale	Screening	Intra-operatively
1. Written informed consent obtained from subjects indicating that they understand the purpose of and procedures required for the study and are willing to participate in it	Ethical aspects	X	
2. Subjects scheduled for **elective cyto-reductive PMP surgery** with expected major blood loss	Disease requirement	X	
3. Male or female, aged ≥ 18	Study population requirement	X	
4. No increased bleeding risk as assessed by standard coagulation tests and medical history *	Pre-treatment requirement	X	
5. Intra-operative trigger for treatment: ** Intra-operative prediction of clinically relevant bleeding of > 2 L, requiring hemostatic treatment during surgery	Pre-treatment requirement		X

* Inclusion criterion no. 4 aims to ensure that only subjects without hereditary bleeding disorders are to be included in this study.

Subjects with continuous 'aspirin intake', subjects under Direct Oral Anti-Coagulants (DOACs), or with a hepatic disease are at higher bleeding risk. Nevertheless, these subjects can be included at the discretion of the investigator.

** Approximately 60 minutes after the start of cytoreductive PMP surgery.

4.3 Exclusion Criteria

Subjects having any of the following criteria, either at screening and/or at baseline will not be included in the study:

Exclusion Criteria	Rationale	Screening	Baseline (prior start of surgery)
1. Pregnancy or unreliable contraceptive measures or breast feeding (women only)	Lack of suitability due to not yet established safety in a clinical study	X	
2. Hypersensitivity to proteins of human origin or known hypersensitivity reactions to components of the IMP	Lack of suitability for study due to safety reasons	X	
3. Participation in another clinical study within 30 days before entering the study or during the study and/or previous participation in this study *	Lack of suitability for study	X	
4. Treatment with any fibrinogen concentrate and/or fibrinogen-containing product within 30 days prior to infusion of IMP	Lack of suitability for study	X	
5. Employee or direct relative of an employee of the CRO, the study site, or Biotest	Ethical aspects	X	
6. Inability or lacking motivation to participate in the study	Subject compliance	X	

Exclusion Criteria	Rationale	Screening	Baseline (prior start of surgery)
7. Medical condition, laboratory finding (e.g. clinically relevant biochemical or hematological findings outside the normal range), or physical exam finding that in the opinion of the investigator precludes participation	Lack of suitability for study due to safety reasons	X	
8. Presence or history of venous/arterial thrombosis or TEE in the preceding 6 months	Lack of suitability for study due to safety reasons	X	

* Exclusion criterion no. 3 aims to ensure that only subjects without participation in another **interventional** clinical study are to be included in this study.

4.4 Subjects Withdrawal Criteria and Replacements

The participation of an individual subject may be terminated prematurely for reasons such as:

a. Withdrawal of written informed consent
b. Study discontinuation due to subject's own request (e.g. personal reasons)
c. Required treatment with any medication known or suspected to interfere with the IMP
d. Life threatening thrombosis or TEE or life threatening hypersensitivity or any AE, laboratory abnormality, or other medical condition or situation occurs suggesting that continued participation in the study would not be in the best interest of the subject.
e. Protocol deviation requiring discontinuation of study treatment
f. Evidence of exclusion criteria or inclusion criteria not met
g. Lack of study compliance
h. Recommendation of the Data Safety Monitoring Board (DSMB)

A subject is entitled to discontinue participation in the clinical study at their own request at any time without stating a reason.

The investigator can terminate a subject's participation in the study at any time if continuation could lead to disadvantages for the subject which cannot be justified by the investigator.

The reason for withdrawal of the subject must be documented by the investigator together with all data collected until the day of premature study termination including laboratory results and assessment of AE. All examinations foreseen for the subject's last study visit (closing visit) should be performed. Afterwards, the subject will be treated according to local standards at the discretion of the investigator.

In case a subject withdraws due to an AE or SAE please follow the instructions given in section 15 Appendix 2: Reporting Procedures of this protocol.

Withdrawn subjects will not be replaced.

For screening failures occurring during the screening period the following data need to be documented in eCRF only:

- Informed consent

- Demographic data
- In-/Exclusion criteria
- End of Study (day and reason)
- AE information

For intra-operative screening failures (not randomized subjects) the following data need to be documented in eCRF only:

- Informed consent
- Demographic data
- In-/Exclusion criteria
- Classification of type of planned PMP surgery (due to underlying cause of the condition, that is the location and extent of the original tumor, including the inability to achieve complete cytoreduction)
- Estimated blood loss
- FIBTEM A10 at Baseline
- Duration of surgery
- End of Study (day and reason)
- AE information

4.5 Subjects Information

The subject will be informed about the clinical study according to the requirements of GCP and the legal requirements of the country in which the subject is recruited.

The clinical study, its objectives, possible benefits and risks, and its consequences will be verbally explained to the subject. Moreover, the subject is provided with written information about the clinical study. Sufficient time will be allowed for the information to be read and for questions to be asked. Attention should be paid to signs of undue distress in subjects who are unable to clearly articulate their distress. The subject must be told that refusal to participate in the clinical study does not cause any disadvantages to their treatment; similarly, withdrawal of written informed consent is possible at any time, without stating a reason and without prejudice to further medical management.

Subjects should be informed and should agree that medical data may be reviewed by authorized persons during monitoring and during an audit or an inspection by the appointed regulatory authority or ethics committee, but that personal data will be treated with absolute confidentiality.

Upon request, the subject must be granted access to the insurance terms and conditions.

Any new and relevant information that evolves during the course of the clinical study concerning the IMP, alternative treatments, or the benefit/risk ratio will be communicated to the subject.

4.6 Declaration of Informed Consent

The subject must have given written consent to participate in the clinical study by signing and personally dating the Informed Consent Form (ICF). Informed consent to the proposed data handling and to data inspection must also be documented in written form. Written informed consent must be obtained from each subject before any study-related procedures are performed. The subject's written informed consent will be filed at the investigator's site.

A duplicate of the signed and dated written ICF must be handed over to the subject.

5 INVESTIGATIONAL MEDICINAL PRODUCTS

5.1 Investigational Medicinal Product BT524

BT524 is a lyophilized, heat-treated, virus and prion safe human fibrinogen concentrate manufactured from human plasma. Fibrinogen conversion to fibrin strands during blood clot formation is one of the major steps in the coagulation cascade to stop bleeding. In subjects with fibrinogen deficiencies, therapeutic substitution with human fibrinogen concentrate will help correct the hemostatic defect and arrest or prevent bleeding.

The manufacturing process of BT524 contains [redacted] steps that were shown to be effective for removal/inactivation of enveloped viruses such as HIV, HBV and HCV, and for the non-enveloped viruses such as Reo, HAV and parvovirus B19. Moreover, [redacted] are effectively removed during the production process.

Thus, BT524 is a virus and prion safe plasma-derived product fulfilling the requirements of the national German and European (CHMP) guidelines on a virus and prion safe pharmaceutical product.

BT524 is presented as a single-use vial with a nominal content of 1 g fibrinogen (lyophilized powder for solution for injection/infusion) to be reconstituted under aseptic conditions with 50 mL of water for injections, resulting in a final concentration of 20 mg/mL for infusion.

5.1.1 Description of Investigational Medicinal Product BT524

Substance code:	BT524
Active ingredients:	Fibrinogen concentrate from human plasma
Composition:	Lyophilized powder for solution for injection/infusion
Dosage form:	1 g
Concentration:	20 mg/mL after reconstitution with 50 mL water for injections
Container:	100 mL glass vial with rubber stopper
Manufacturer:	Biotest AG, D-63303 Dreieich, Germany

Batch number and expiry date are given in the applicable certificates of analysis.

5.1.2 Formulation, Packaging and Labelling

BT524 is a lyophilized, heat-treated fibrinogen concentrate manufactured from human plasma according to the description of Ph. Eur. monograph 0024 on Human Fibrinogen.

BT524 drug product is presented as a single-use 100 mL Type I glass vial with a nominal content of 1 g fibrinogen (lyophilized powder for solution for injection/infusion).

The labelling of BT524 will be performed according to local requirements. A sample label will be filed in the Trial Master File (TMF).

Batch number and vial number must be documented in the eCRF and the drug accountability log.

5.1.3 Storage Conditions and Stability

BT524 is to be stored in a cabinet or other enclosure which is security locked. Generally access should be restricted to the investigator and authorized personnel.

BT524 is to be stored at a temperature CCI [redacted]. Continuous temperature recording should be documented on a temperature log.

5.1.4 Preparation for Use

BT524 is presented as a single-use glass vial of 100 mL with a nominal content of 1 g of lyophilisate (powder for solution for injection/ infusion). The lyophilisate is to be reconstituted under aseptic conditions with 50 mL of water for injections using an appropriate transfer device or syringe.

The vial is to be swirled gently until the product is fully dissolved. After reconstitution, the solution should be almost colorless and clear to slightly opalescent. Reconstituted products should be inspected visually for particulates and discoloration prior to administration. Do not use solutions that are cloudy or contain deposits.

BT524 will be delivered to the operating room by the pharmacy according to local practice and immediately after reconstitution administered by the unblinded anaesthesiologist.

5.2 Investigational Medicinal Product Cryoprecipitate

Cryoprecipitate will be used as active comparator to BT524 in subjects undergoing cytoreductive PMP surgery at one site in the UK.

Cryoprecipitate is produced in the UK, USA, Canada, Australia, and New Zealand, where it is mainly used as a concentrated source of fibrinogen for treatment of acquired hypofibrinogenaemia.

Cryoprecipitate consists of the cryoglobulin fraction of plasma containing the major portion of Factor VIII and fibrinogen. It is obtained by thawing a single donation of FFP at 4°C ± 2°C resulting in the formation of the cryoprecipitate. Following centrifugation, the supernatant plasma is removed. The cryoprecipitate is then rapidly frozen to ≤ -25°C. It is available as single units or as pools of five. A single unit contains a mean of approximately 400 to 460 mg fibrinogen. The adult therapeutic dose is two pools of five units (NHS Blood and Transplant, May 2018. Access Date: 05-Feb-2020.; NHSBT, 2016; Walters, 2016).

5.2.1 Description of Investigational Medicinal Product Cryoprecipitate

Substance code:	Cryoprecipitate
Active ingredients:	Human plasma proteins (fibrinogen, vWF, factor VIII, factor XIII, fibronectin and platelet microparticles
Dosage form:	AB0-blood group specific solution for infusion, which appears (slightly) yellow
Concentration:	400-460 mg (per unit)
Container	Bags with pooled cryoprecipitate containing approximately 100 - 300 mL frozen solution (mean 236 mL)

5.2.2 Formulation, Packaging and Labelling

The labelling of cryoprecipitate will be performed according to local requirements.

Bag number must be documented in the eCRF and the drug accountability log.

5.2.3 Storage Conditions and Stability

In general, cryoprecipitate will be stored protected from light, at a temperature at ≤ -25°C. Continuous temperature recording should be documented on a temperature log.

5.2.4 Preparation for Use

Cryoprecipitate is to be thawed and prepared for administration by the blood bank according to local standards, delivered to the operating room, and administered upon arrival.

Cryoprecipitate should be inspected visually for particulate matter and discoloration prior to administration, whenever solution and container permit. Do not use if turbid. Avoid shaking.

Once thawed, cryoprecipitate should be used immediately. If delay is unavoidable, cryoprecipitate should be stored at ambient temperature and used within four hours. Thawed plasma components must not be re-frozen. The infusion must be completed as soon as possible and within 4 hours of thawing (Walters, 2016). ABO group identical cryoprecipitate should be given whenever possible; if not possible cryoprecipitate of a different ABO group may be acceptable according to local transfusion standards.

6 STUDY TREATMENT

6.1 Dosage Regimen

After a bleeding risk assessment, which takes place approximately 60 minutes after the start of cytoreductive PMP surgery, only subjects with the prediction of an intra-operative clinically relevant bleeding of more than 2 L, requiring hemostatic treatment (high risk for the need of fibrinogen supplementation with BT524 or cryoprecipitate) during surgery, will be treated with BT524 or cryoprecipitate according to the predefined treatment algorithm.

6.2 Dosage and Administration

The first IMP (BT524 or cryoprecipitate) dose will be administered pre-emptively, based on clinical judgement (prediction of clinically relevant bleeding of more than 2 L, requiring hemostatic treatment during surgery).

Further IMP administration depends on the extent of bleeding and the subject's clinical condition, and will be guided by FIBTEM A10 results. Therefore, the functional fibrinogen level should be determined intra-operatively on an individual subject basis by FIBTEM thromboelastometry prior to IMP administration.

A FIBTEM A10 < 12 mm may trigger the administration of IMP in case a further clinically relevant bleeding occurs. Nevertheless, IMP may be administered prior to the determination of FIBTEM A10 in a subject who is bleeding if deemed appropriate as per current clinical standards (that is suspected acquired hypofibrinogenaemia with rapid bleeding precluding waiting for laboratory results).

Blinding

Study 995 will be partially blinded; surgeon, surgical staff and subjects will be blinded to treatment allocation throughout the entire surgery. The anaesthesiologist who will administer the IMP could not be blinded to treatment allocation because of the inherent characteristics of the IMPs BT524 and cryoprecipitate. Whereas BT524 is administered by syringe with a small volume and fast infusion to rapidly supplement missing fibrinogen to restore hemostasis, cryoprecipitate is administered as infusion bag with larger volume and longer infusion time. Due to the different methods of application, the different volume, resulting in different infusion times of the two IMPs a blinding technique at the level of the anaesthesiologist is considered not feasible (either impossible or heavily impractical) to allow the study to be conducted successfully.

The basis for the partial blinding in the operating room is the spatial separation between the anaesthetic field and the surgical, sterile field (operating field). As a basic principle, the anaesthesiologist remains outside of the operating field. By using a sterile drape with non-transparent material between these two fields, the surgeon will not be able to see which IMP is being administered by the anaesthesiologist. The sterile drape will especially cover up the infusion stands and therefore the administration of cryoprecipitate. The anaesthesiologist has a professional and organizational responsibility regarding the maintenance of blinding of the surgeon and the surgical staff during the entire surgery. In general, the anaesthesiologist is responsible for administering all medications including IMP, and for monitoring and maintenance of vital functions (including e.g. heart rate, oxygen saturation) and laboratory values of the subject.

BT524

The dose of BT524 to be infused will be a fixed dose of 4 g with the aim of restoring the fibrinogen plasma levels according to guidelines. The first BT524 dose will be administered pre-emptively. In case of repeated dosing, regular monitoring of the plasma level of fibrinogen (FIBTEM A10) during therapy is indicated. Subjects randomized to the BT524 group will receive 4 g BT524 each time fibrinogen supplementation is ordered.

BT524 will be administered as IV infusion with a maximum infusion rate of [redacted]/min (1 g fibrinogen concentrate [redacted]). BT524 is to be administered preferably in the forearm vein. Alternatively, BT524 can be administered through a central venous line or a peripherally inserted central catheter (PICC). Other administration routes are only allowed after approval from the sponsor.

Cryoprecipitate

Cryoprecipitate will be administered by IV infusion following hospital transfusion policies at the participating study site. A single unit of cryoprecipitate contains approximately 400 to 460 mg fibrinogen. The adult therapeutic cryoprecipitate dose is 2 pools, each pool consisting of 5 units/donations (10 units, dose-equivalent to 4 g fibrinogen concentrate), which should increase plasma fibrinogen levels by 1 g/L depending on the clinical setting.

The first cryoprecipitate dose will be given pre-emptively. Further cryoprecipitate administration intra-operatively will be guided by the FIBTEM A10 test. Subjects randomized to the cryoprecipitate group will receive 2 pools of cryoprecipitate each time IMP (fibrinogen supplementation) is ordered. If cryoprecipitate will be administered with

a common infusion rate of 10 - 20 mL/kg/hr, 2 pools can be infused in approximately 30 minutes.

6.3 Compliance with Dosage Regimens

As the IMP (BT524 or cryoprecipitate) will be administered IV to each subject under the supervision of the unblinded anaesthesiologist, the compliance is expected to be 100%. In addition, the assessment of plasma fibrinogen concentrations may also serve as an adherence measure.

If a subject's treatment deviates from the dosage regimen (e.g., a dosing interruption occurs due to the occurrence of an AE), this will be recorded in the eCRF.

6.4 Dose Justification

In general, the dosage of IMP (BT524 or cryoprecipitate) depends on the clinical situation and the extent of bleeding during surgery.

Subjects undergoing cytoreductive surgery for PMP will be randomized to one of the two treatment arms, when clinical judgement approximately 60 minutes after the start of cytoreductive PMP surgery predicts an intra-operative bleeding of > 2 L (without targeted fibrinogen replacement), requiring hemostatic treatment during surgery. Subjects will be treated pre-emptively with BT524 or cryoprecipitate as the primary hemostatic therapy.

Cryoprecipitate is available as a single unit, or as a pooled product made up of five single units. A commonly used adult dose is 2 pools, each pool consisting of 5 units/donations (10 single units), and containing approximately 4 g fibrinogen. Therefore, the corresponding BT524 dose is 4 g. The repeated further IMP administration (4 g BT524 or 2 pools cryoprecipitate) will be dependent on the clinical condition (i.e. clinically relevant bleeding) and may be triggered by FIBTEM A10 results of <12 mm.

Fibrinogen is the first coagulation factor to become critically reduced during major surgical blood loss and the observation that patients with higher fibrinogen levels experience fewer bleeding complications than those with low levels highlights the importance of fibrinogen in the maintenance of hemostasis (Charbit et al., 2007; Ucar et al., 2007). Consequently, fibrinogen replacement therapy targeting a high-normal level of plasma fibrinogen and fibrin-based clot formation may be an important first step in restoring hemostasis during major bleeding (Rahe-Meyer et al., 2013a).

The normal blood fibrinogen concentration is between 2.0 and 4.5 g/L although this range can vary (Levy and Goodnough, 2015). Restoring the individual physiological fibrinogen level seems reasonable to ensure sufficient fibrinogen supplementation without risk of overdosing.

Therefore, the repeated IMP dosing in this study will be guided by determination of the functional fibrinogen level via ROTEM/FIBTEM thromboelastometry intra-operatively with the aim to restore patient's fibrinogen level. A FIBTEM A10 < 12 mm and evidence of clinically relevant bleeding may trigger the administration of IMP (4 g BT524 or 2 pools cryoprecipitate). In line with current clinical standards, subjects with relevant bleeding may also be treated if acquired hypofibrinogenaemia is suspected but plasma FIBTEM A10 results are still pending at the time of required treatment.

Rotational thromboelastometry (ROTEM) is an established viscoelastic method for hemostasis testing in whole blood, and ROTEM/FIBTEM that measures the fibrin or fibrinogen contribution to clot strength can be used to determine the most appropriate therapeutic dose of fibrinogen concentrate (Levy et al., 2014).

The ROTEM/FIBTEM test will be used in the intra-operative setting to quickly identify deficits in fibrin quality, and to guide hemostatic therapy. In contrast to conventional laboratory tests (measurement of fibrinogen concentration via Clauss assay), ROTEM/FIBTEM can measure early variables describing the clot firmness, such as clot amplitude obtained after 10 minutes (A10), and provide a forecast on the expected Maximum Clot Firmness (MCF) value at an earlier stage already. This early variable allows for a more rapid decision about therapeutic interventions. Owing to its rapid assessment of fibrinogen, ROTEM is frequently used to guide transfusion therapy (Schochl et al., 2011), and the data from Schochl and colleagues revealed the effective use of ROTEM-guided coagulation management in trauma patients by reducing the amount of allogeneic blood product transfusion (Schochl et al., 2010).

Accordingly, the IMP treatment in this clinical study will be guided by ROTEM/FIBTEM to obtain results more quickly and to initiate repeated dosing if FIBTEM A10 is less than 12 mm in case a clinically relevant bleeding occurs..

6.5 Treatment of Overdose

In order to avoid overdosage in subjects with acquired hypofibrinogenaemia undergoing major surgery, point-of-care FIBTEM guided dosing according to fibrinogen plasma levels will be used. Monitoring of the plasma level of fibrinogen intra-operatively will be performed as defined in section III.

In case of overdosage, the risk of development of thromboembolic complications is enhanced (EMA (Committee for Medicinal Products for Human Use), 2015).

6.6 Randomization Code

There will be a stratified randomization per surgery type. Subjects undergoing cytoreductive PMP surgery are to be randomized on a 1:1 basis to receive either BT524 or cryoprecipitate.

Randomization of PMP subjects to treatment will occur intra-operatively (pre-dose) when eligibility for the clinical study has been confirmed. Immediately after the prediction of clinically relevant bleeding > 2 L, requiring hemostatic treatment (high risk for the need of fibrinogen supplementation with BT524 or cryoprecipitate) during surgery, the person responsible for randomization will be informed and the randomization request will be sent. The person responsible for randomization retrieves the randomization code via Interactive Web Response System (IWRS) and informs the blood bank/pharmacy.

BT524 will be delivered to the operating room by the pharmacy. After randomization BT524 will be prepared in the operating room in accordance with the manufacturer's instructions and administered immediately after reconstitution by the unblinded anaesthesiologist.

After randomization, cryoprecipitate will be thawed and prepared for administration by the local blood bank, delivered to the operating room, and administered upon arrival.

In case the anaesthesiologist retrieves the randomization code via IWRS, the blood bank/ pharmacy will be informed accordingly and can provide the IMP.

Subject Identification

For the coherent assignment of the study documents all subjects having signed the informed consent and having entered the screening period will receive a subject number. The subject number comprises a five digit number of which the first two digits define the

investigational site and the last three digits the subject enrolled at the corresponding site. Subject numbers are assigned consecutively per site. Subject numbers are assigned unique and will not be replaced i.e., in case of a screening failure.

An interactive web/voice response system (IWRS/IVRS) will be implemented and used for randomization and re-supply. Detailed instructions for the use of IWRS systems are provided in a separate document that will be filed in the Investigator Site File.

The random allocation of treatments to subjects will be done using a computerized randomization program. Subjects will receive a randomization number, which will be recorded along with the date of randomization in the eCRF.

6.7 Procedures for Emergency Unblinding

In the event of an emergency, each study site will be able to unblind subject treatment allocation via IWRS (either the principal investigator or a designated medic sub-investigator at the study site) without undue delay.

However, unblinding shall only be carried out if a medical emergency requires the identification of the IMP for that particular participant. If the code is broken for a subject (via the IWRS), Biotest must be informed immediately. The reason for opening the code break must be documented on the appropriate eCRF page along with the date and the initials of the person who broke the code.

Any subject for whom the blind is broken will be discontinued from the clinical study, however will be followed-up.

6.8 Drug Accountability

The IMP BT524 will be supplied to the investigator at the time of site initiation under the assumption that all required regulatory documents are in place. The investigator or his/her designee should maintain records that document adequately that the subjects were provided the doses specified in the protocol and reconcile all IMPs received for the clinical study. The investigator has to ensure that consignments of IMP are received correctly by a dedicated person (e.g. blood bank, pharmacy) and that the IMP is safely and appropriately handled and stored.

The investigator or designee is obliged to keep sufficient documentation of the delivery, use, and destruction or return of unused, used or partially used packages of IMP. The investigator must allow the monitor to perform drug reconciliation before any IMP is returned or destroyed. The documentation must include dates, quantities, subject numbers, batch numbers (if applicable), bag numbers (if applicable) and expiry dates.

The entries in the eCRF as well as the documentation kept in the Investigator Site File will be compared with the returned and residual IMPs, with clarification of any discrepancies or inconsistencies.

6.9 Previous and Concomitant Medication or Treatment

All previous medication and treatment in the previous 4 weeks prior to the elective surgery and the administration of IMP are to be recorded in the eCRF.

Concomitant medication therapeutically required is allowed during the study. If a change in concomitant medication is necessary during the study, it is the responsibility of the investigator to ensure that details regarding the medication are recorded in full in the

eCRF (i.e. identity of all medications, dosage and route of administration, frequency, duration of administration, and indication for use at each visit).

6.10 Prohibited Medication or Treatment

The administration of allogeneic or autologous blood products interacting relevantly with the coagulation system (e.g. platelet concentrates, cryoprecipitate, FFP) and of hemostatic agents (including coagulation factor concentrates) is not allowed prior to start of or during the first IMP administration within this study. In such instances, the subject can not be randomized or will be withdrawn from the study.

RBCs can be infused according to institutional practice.

6.11 Warnings and Precautions

Brief Summary

A Guideline for Core SmPC for Human Fibrinogen Products (EU Core SmPC) is in place which also describes the well established benefit-risk profile of the authorized fibrinogen concentrates [Guideline on Core SmPC for Human Fibrinogen Products (EMA (Committee for Medicinal Products for Human Use), 2015)].

It provides the following AEs as undesirable effects considered established for all authorized and marketed human fibrinogen concentrate formulations:

In the MedDRA System Organ Class (SOC) "Immune system disorders": Allergic or anaphylactic-type reactions; in the MedDRA SOC "Vascular disorders": Thromboembolic episodes (including myocardial infarction and pulmonary embolism), and in the MedDRA SOC "General disorders and administration site conditions": Increase in body temperature.

Description of Potential Risks

There is a risk of thrombosis when subjects, with either congenital or acquired deficiency, are treated with human fibrinogen particularly with high dose or repeated dosing. Subjects given human fibrinogen should be observed closely for signs or symptoms of thrombosis. In subjects with a history of coronary heart disease or myocardial infarction, in subjects with liver disease, in peri- or post-operative subjects, in neonates, or in subjects at risk of TEEs or DIC, the potential benefit of treatment with human plasma fibrinogen should be weighed against the risk of thromboembolic complications. Caution and close monitoring should also be performed.

Acquired hypofibrinogenaemia is associated with low plasma concentrations of all coagulation factors (not only fibrinogen) and inhibitors and so treatment with blood products containing coagulation factors should be considered. Careful monitoring of the coagulation system is necessary.

If allergic or anaphylactic-type reactions occur, the injection/infusion should be stopped immediately. In case of anaphylactic shock, standard medical treatment for shock should be implemented.

There is currently no data on fibrinogen inhibitors available with human fibrinogen.

Standard measures to prevent infections resulting from the use of medicinal products prepared from human blood or plasma include selection of donors, screening of individual donations and plasma pools for specific markers of infection and the inclusion of effective manufacturing steps for the inactivation/removal of viruses. Despite this, when medicinal

products prepared from human blood or plasma are administered, the possibility of transmitting infective agents cannot be totally excluded. This also applies to unknown or emerging viruses and other pathogens.

The measures taken are considered effective for enveloped viruses such as human immunodeficiency virus (HIV), hepatitis B virus (HBV) and hepatitis C virus (HCV) and for the non-enveloped hepatitis A and parvovirus B19 viruses. Every time that fibrinogen is administered to a patient, the name and batch number of the product are to be recorded in order to maintain a link between the patient and the batch of the product.

Special precautions for BT524 administration

All plasma-derived human products may lead to allergic or anaphylactic reactions. Thus, the administration of BT524 will be performed under medical supervision where proper medical care for allergic or anaphylactic reactions can be provided. If allergic or anaphylactic-type reactions occur, the infusion must be stopped immediately. In case of anaphylactic shock, standard medical treatment for shock must be applied.

Prothrombin time PT(INR), activated partial thromboplastin time (aPTT), prothrombin fragments F_{1+2}, thrombin-antithrombin III complex (TAT), D-dimer, PS, PC, antithrombin III (AT III) activity, thrombin time (TT), thrombin generation test (TGT)[2] and further coagulation parameters are monitored regularly to detect a hypercoagulable state.

Changes in vital signs (including pulse [heart rate], blood pressure, respiratory rate, body temperature) are monitored during the surgery and according to the flow chart (see section III).

Incompatibilities

Fibrinogen must not be mixed with other medicinal products and should be administered by a separate injection/infusion line.

[2] Only applicable for subjects undergoing cytoreductive surgery for PMP

7 COURSE OF THE CLINICAL STUDY

7.1 Visit Schedule for PMP subjects

Screening Visit Day -42 through Day -1:

All subjects will attend a screening visit (if required more than one visit) between Day -42 and Day -1 prior to elective major cytoreductive PMP surgery, where the following procedures will be performed:

- **Written informed consent** will be obtained.
- In general, eligibility to take part in the study will be assessed against the **inclusion and exclusion criteria**.
- **Demographic data** (including sex, year of birth, race) will be recorded.
- Classification of **type of the planned PMP surgery** will be recorded.
- **Physical examination** (including body weight and body height) will be performed.
- **Body weight** will be recorded.
- A serum **pregnancy test** (human chorionic gonadotropin) will be performed for all females of child-bearing potential (a woman of child bearing potential is one that has NOT had a hysterectomy and/or a bilateral oophorectomy, or has NOT been naturally postmenopausal for at least 24 consecutive months).
- **Medical and surgical history** with regard to the subjects' drug history, previous medication and treatment, disease history, and other medical and surgical history will be recorded. Previous medication taken up to 4 weeks before enrollment will be recorded. The type of previous medication, dose schedule, duration, and the indication the previous medication was given for will be documented.
- **Vital signs** (including pulse [heart rate], blood pressure, respiratory rate, body temperature) will be recorded.
- Samples for **clinical laboratory** parameters (hematology, clinical chemistry, urinalysis) will be taken:
  - **Hematology** (RBC, WBC, platelet count, hemoglobin, hematocrit)
  - **Clinical chemistry** (ALAT, ASAT, γ-GT, AP, total bilirubin, creatinine, creatinine clearance, BUN/Urea, potassium, sodium, calcium, chloride)
  - **Urinalysis** (pH, blood, WBC, protein, glucose, ketone bodies, nitrite, bilirubin, urobilinogen)

  Some of these tests for clinical laboratory parameters may have been performed by the investigator as standard of care prior to the subject signing the ICF. If test(s) were done within 42 days before the first scheduled treatment with IMP (BT524 or cryoprecipitate), the investigator may use the results obtained from standard of care for the purpose of this study.
- Sample for **coagulation activation tests** (PT(INR), aPTT, TAT, F_{1+2}, D-dimer, PS, PC, AT III, TT, TGT) will be taken.
- Sample for **plasma activity of fibrinogen** (Clauss assay) will be taken.
- **FIBTEM A10 and Maximum clot firmness (MCF)** (ROTEM) will be performed.
- **Retention samples for viral safety** laboratory parameters will be taken.

- **Samples for virus serology laboratory parameters** (hepatitis B, hepatitis C, HIV) will be taken.
- **AEs** will be documented.

If a subject does not fulfill the eligibility criteria, re-assessment is allowed within the 42 days screening period.

Baseline Visit Day 1 (or Day -2 or Day -1, prior to surgery):

All subjects will attend a baseline visit on Day 1, the day of PMP surgery. The baseline visit can be scheduled one or two days prior to surgery (Day -2 or Day -1) if required due to local hospital procedures.

The following assessments will be performed **prior to start of surgery, before** the potential IMP administration (BT524 or cryoprecipitate):

- **Re-check of inclusion and exclusion criteria**: Eligibility to take part in the study will be confirmed against the inclusion and exclusion criteria, as appropriate (i.e., diagnostic tests will be repeated at the investigator's discretion).
- Any **changes in medical and surgical history** since the screening visit will be recorded. The type of previous medication, dose schedule, duration, and the indication the previous medication was given for, will be documented.
- **Expected blood loss** will be recorded.
- **Physical examination** will be performed.
- **Pregnancy test** (urine or serum) will be performed for all females of child-bearing potential (a woman of child bearing potential is one that has NOT had a hysterectomy and/or a bilateral oophorectomy, or has NOT been naturally postmenopausal for at least 24 consecutive months).
- **Body weight** will be recorded to calculate subject dose.
- **Vital signs** (including pulse [heart rate], blood pressure, respiratory rate, body temperature) will be recorded.
- Samples for **clinical laboratory** parameters (hematology, clinical chemistry, urinalysis) will be taken:
  - **Hematology** (RBC, WBC, platelet count, hemoglobin, hematocrit)
  - **Clinical chemistry** (ALAT, ASAT, γ-GT, AP, total bilirubin, creatinine, creatinine clearance, BUN/Urea, potassium, sodium, calcium, chloride)
  - **Urinalysis** (pH, blood, WBC, protein, glucose, ketone bodies, nitrite, bilirubin, urobilinogen)
- Sample for **coagulation activation tests** (PT(INR), aPTT, TAT, F_{1+2}, D-dimer, PS, PC, AT III, TT, TGT) will be taken.
- Sample for **coagulation factors** (FII, FV, FVII, FVIII, FIX, FX, FXI, FXIII, and vWF) will be taken.
- Sample for **plasma activity of fibrinogen** (Clauss assay) will be taken and
- **FIBTEM A10 and MCF** (ROTEM) will be performed.
- **AEs** will be documented.

***Please note:** The following tests and assessments have to be done prior to surgery. In case of a short time-period between screening and baseline (≤ 2 days) these tests have only to be repeated based on medical judgment of the investigator. If not repeated, screening results will serve as baseline.*

- Physical examination
- Pregnancy test
- Body weight
- Hematology
- Clinical chemistry
- Urinalysis
- Coagulation activation tests
- Plasma activity of fibrinogen (Clauss assay)
- FIBTEM A10 and MCF (ROTEM)

Surgery Day 1 (pre-dose):

- **Time of start of surgery** will be recorded.
- Continuous **collection and measurement of blood loss** from start of surgery.
- Amount of blood in blood suction unit and swabs, surgical cloths and compresses will be estimated.
- **Approximately 60 minutes after the start of cytoreductive PMP surgery, eligibility** to take part in the study will be assessed against the intra-operative inclusion criteria:

  an intra-operative prediction of clinically relevant bleeding of > 2 L, requiring hemostatic treatment (high risk for the need of fibrinogen supplementation with BT524 or cryoprecipitate) during surgery.

In subjects who do not meet the intra-operative inclusion criteria no IMP administration will take place and no further assessments intra-operatively will be performed. These subjects will be considered as screening failures and will be treated with standard of care.

Only for subjects considered to be eligible for IMP administration with BT524 or cryoprecipitate the following assessments will be performed:

- **Time of decision** to treat the subject with IMP will be recorded.
- **Amount of blood** from blood suction unit and from swabs, surgical cloths and compresses after decision to treat the subject with IMP until end of surgery will be measured.
- Sample for plasma **activity of fibrinogen** (Clauss assay) will be taken.
- **FIBTEM A10 and MCF** (ROTEM) will be performed.
- **Randomization** will be initiated **with order of IMP.**
- Sample for **coagulation activation tests** (PT(INR), aPTT, TAT, F_{1+2}, D-dimer, PS, PC, AT III, TT, TGT) will be taken prior start of first IMP administration.

- Sample for **coagulation factors** (FII, FV, FVII, FVIII, FIX, FX, FXI, FXIII, and vWF) will be taken prior start of first IMP administration.
- Samples for **clinical laboratory parameters** (hematology, clinical chemistry) will be taken prior start of first IMP administration:
    - **Hematology** (RBC, WBC, platelet count, hemoglobin, hematocrit)
    - **Clinical chemistry** (ASAT, ALAT, creatinine, creatinine clearance, BUN/Urea, γ-GT, AP, total bilirubin, potassium, sodium, calcium, chloride)
- **Vital signs** (pulse [heart rate], blood pressure, respiratory rate, body temperature) will be recorded prior start of IMP administration.
- **Transfusion products** (allogenic blood products) given intra-operatively will be documented.
- **Concomitant medication or treatment** will be recorded. The type of concomitant medication/treatment, dose/schedule, duration, and the indication the concomitant medication was given for will be documented.
- **AEs** will be documented.
- **IMP** (4 g BT524 or 2 pools cryoprecipitate) will be infused IV.

  The total volume and the total infusion time of each IMP administration (start and end of infusion) will be recorded.

Surgery Day 1 (post-dose):

- Continuous **collection and measurement of blood loss**
- **Vital signs** (pulse [heart rate], blood pressure, respiratory rate, body temperature) will be recorded **15 minutes** and **90 minutes** after start of IMP administration.
- Sample for **coagulation activation tests** (PT(INR), aPTT, TAT, F_{1+2}, D-dimer, PS, PC, AT III, TT, TGT) will be taken only **15 minutes** and **90 minutes** after start of **first IMP** administration.
- Sample for **coagulation factors** (FII, FV, FVII, FVIII, FIX, FX, FXI, FXIII, and vWF) will be taken only **90 minutes** after start of **first IMP** administration.
- Sample for plasma **activity of fibrinogen** (Clauss assay) will be taken only **15 minutes** and **90 minutes** after start of **first IMP** administration.
- **FIBTEM A10 and MCF** (ROTEM) will be performed only **15 minutes** and **90 minutes** after start of **first IMP** administration.
- **Transfusion products** (allogenic blood products) given intra-operatively will be documented.
- **Concomitant medication or treatment** will be recorded. The type of concomitant medication/treatment, dose/schedule, duration, and the indication the concomitant medication/treatment was given for will be documented.
- **AEs** will be documented.

Please note: *The following tests have to be done 90 minutes after start of **first IMP** treatment. In case this time point is within a short timeframe (< 30 min) after the 'end of surgery', these tests have only to be repeated based on medical judgment of the investigator.*

- Markers of coagulation (Coagulation activation tests)
- Plasma activity of fibrinogen (Clauss assay)
- FIBTEM A10 and MCF (ROTEM)
- Coagulation factors

Surgery Day 1 (prior to repeated IMP administration):

- **Vital signs** (pulse [heart rate], blood pressure, respiratory rate, body temperature) will be recorded prior start of each IMP administration.
- **FIBTEM A10 and MCF** (ROTEM) will be performed.
- **IMP** will be ordered: 4 g BT524 or 2 pools cryoprecipitate, according to the previously assigned treatment arm.
- **IMP** will be administered. The total volume and the total infusion time of each IMP administration (start and end of infusion) will be recorded.
- **Amount of blood** from blood suction unit and from swabs, surgical cloths and compresses will be collected and measured.
- **Transfusion products** (allogenic blood products) given intra-operatively will be documented.
- **Concomitant medication or treatment** will be recorded.
- **AEs** will be documented.

Surgery Day 1 (end of surgery):

- **Vital signs** (including pulse [heart rate], blood pressure, respiratory rate, body temperature) will be recorded at the end of the surgery.
- Samples for **clinical laboratory parameters** (hematology, clinical chemistry) will be taken at the end of the surgery:
    - **Hematology** (RBC, WBC, platelet count, hemoglobin, hematocrit)
    - **Clinical chemistry** (ASAT, ALAT, creatinine, creatinine clearance, BUN/Urea, γ-GT, AP, total bilirubin, potassium, sodium, calcium, chloride)
- Sample for **coagulation activation tests** (PT(INR), aPTT, TAT, F_{1+2}, D-dimer, PS, PC, AT III, TT, TGT) will be taken at the end of the surgery.
- Sample for coagulation factors (FII, FV, FVII, FVIII, FIX, FX, FXI, FXIII, and vWF) will be taken at the end of the surgery.
- Sample for plasma **activity of fibrinogen** (Clauss assay) will be taken at the end of the surgery.
- **FIBTEM A10 and MCF** (ROTEM) will be performed at the end of the surgery.
- Start continuous **measurement of blood loss** until 24 hours post-operative.
- **Amount of blood** from blood suction unit and from swabs, surgical cloths and compresses will be calculated and recorded (blood loss after the decision to treat the subject with IMP until end of surgery).

- **Transfusion products** (allogenic blood products) given intra-operatively will be documented.
- **Concomitant medication or treatment** will be recorded. The type of concomitant medication/treatment, dose/schedule, duration, and the indication the concomitant medication//treatment was given for will be documented.
- **AEs** will be documented.
- **Time of end of surgery** (time of last suture) will be recorded.
- **Rebleeding episodes** will be recorded.

Please note: *The following tests have to be done at the 'end of surgery'. In case the end of surgery is within a short timeframe (< 30 min) after the blood draw '90 minutes after start of first IMP treatment', these tests have only to be repeated based on medical judgment of the investigator.*

- Markers of coagulation (Coagulation activation tests)
- Plasma activity of fibrinogen (Clauss assay)
- FIBTEM A10 and MCF (ROTEM)
- Coagulation factors

Follow-up Visit Day 2:

All subjects treated with IMP will attend the follow-up visit one day after surgery (Day 2). The following procedures will be performed:

- Continuous measurement of post-operative blood loss (drainage volume) and recording until 24 hours after end of surgery.
- **Physical examination** will be performed.
- **Vital signs** (including pulse [heart rate], blood pressure, respiratory rate, body temperature) will be recorded.
- Sample for **coagulation activation tests** (PT(INR), aPTT, TAT, F_{1+2}, D-dimer, PS, PC, AT III, TT, TGT) will be taken.
- Samples for **clinical laboratory parameters** (hematology, clinical chemistry, urinalysis) will be taken:
  - **Hematology** (RBC, WBC, platelet count, hemoglobin, hematocrit)
  - **Clinical chemistry** (ASAT, ALAT, creatinine, creatinine clearance, BUN/Urea, γ-GT, AP, total bilirubin, potassium, sodium, calcium, chloride)
  - **Urinalysis** (pH, blood, WBC, protein, glucose, ketone bodies, nitrite, bilirubin, urobilinogen)
- Sample for plasma **activity of fibrinogen** (Clauss assay) will be taken and
- **FIBTEM A10 and MCF** (ROTEM) will be performed.
- **Transfusion products** (allogenic blood products) will be documented.
- **Concomitant medication or treatment** will be documented. The type of concomitant medication/treatment, dose/schedule, duration, and the indication the concomitant medication/treatment was given for will be documented.
- **AEs** will be documented.
- **Rebleeding episodes** will be recorded.

Follow-up Visits Day 3, Day 5 and Day 8:

All subjects treated with IMP will attend the follow-up visits 2, 4 and 7 days after surgery (Day 3, 5 and 8):

The following procedures will be performed:

- **Physical examination** will be performed.
- **Vital signs** (including pulse [heart rate], blood pressure, respiratory rate, body temperature) will be recorded.
- Sample for **coagulation activation tests** (PT(INR), aPTT, TAT, F_{1+2}, D-dimer, PS, PC, AT III, TT, TGT) will be taken.
- Samples for **clinical laboratory parameters** (hematology, clinical chemistry, urinalysis) will be taken:
  - **Hematology** (RBC, WBC, platelet count, hemoglobin, hematocrit)
  - **Clinical chemistry** (ASAT, ALAT, creatinine, creatinine clearance, BUN/Urea, γ-GT, AP, total bilirubin, potassium, sodium, calcium, chloride)
  - **Urinalysis** (pH, blood, WBC, protein, glucose, ketone bodies, nitrite, bilirubin, urobilinogen)
- **Transfusion products** (allogenic blood products) will be documented.
- **Concomitant medication or treatment** will be documented. The type of concomitant medication/treatment, dose/schedule, duration, and the indication the concomitant medication/treatment was given for will be documented.
- **AEs** will be documented.
- **Rebleeding episodes** will be recorded.

Discharge from Hospital:

- The day of the discharge from hospital will be recorded at the **closing visit Day 36**.
- If the closing visit Day 36 (+35, up to Day 71 if required) is scheduled as a phone call, all tests and procedures requiring an in-person visit (physical examination, vital signs, samples for laboratory tests) must be performed on the day of hospital discharge:
  - ***Physical examination***
  - ***Vital signs*** *(including pulse [heart rate], blood pressure, respiratory rate, body temperature)*
  - *Sample for* ***coagulation activation tests*** *(PT(INR), aPTT, TAT,* F_{1+2}*, D-dimer, PS, PC, AT III, TT, TGT)*
  - *Samples for* ***clinical laboratory parameters****:*
    - ***Hematology*** *(RBC, WBC, platelet count, hemoglobin, hematocrit)*
    - ***Clinical chemistry*** *(ASAT, ALAT, creatinine, creatinine clearance, BUN/Urea, γ-GT, AP, total bilirubin, potassium, sodium, calcium, chloride)*
    - ***Urinalysis*** *(pH, blood, WBC, protein, glucose, ketone bodies, nitrite, bilirubin, urobilinogen)*
  - ***Retention samples for viral safety laboratory parameters***

- *Samples for virus serology laboratory parameters (hepatitis B, hepatitis C, HIV)*

Closing Visit Day 36 (+35, up to Day 71 if required):

All subjects treated with IMP will attend a closing visit including the final safety examination, and a safety follow-up at least 5 weeks after the surgery, scheduled on Day 36 (+35, up to Day 71 if required).

Using a phone call instead of a protocol-directed in-person study visit is acceptable if all tests and procedures scheduled for Day 36 (+35) requiring an in-person visit (physical examination, vital signs, samples for laboratory tests), were performed on the day of hospital discharge. These results are to be entered in the eCRF in the fields provided for the closing visit.

The documentation of transfusion products, concomitant medication or treatment, and the recording of AEs can be performed by using a phone call. In addition, the follow-up of AEs can be completed with the subject by telephone (for the follow-up of AEs please refer to section 9.3.4).

An appropriate documentation of the phone call is kept.

The following procedures will be performed:

- A **physical examination** will be performed.
- **Vital signs** (including pulse [heart rate], blood pressure, respiratory rate, body temperature) will be recorded.
- Sample for **coagulation activation tests** (PT(INR), aPTT, TAT, F_{1+2}, D-dimer, PS, PC, AT III, TT, TGT) will be taken.
- Samples for **clinical laboratory parameters** (hematology, clinical chemistry, urinalysis) will be taken:
    - **Hematology** (RBC, WBC, platelet count, hemoglobin, hematocrit)
    - **Clinical chemistry** (ASAT, ALAT, creatinine, creatinine clearance, BUN/Urea, γ-GT, AP, total bilirubin, potassium, sodium, calcium, chloride)
    - **Urinalysis** (pH, blood, WBC, protein, glucose, ketone bodies, nitrite, bilirubin, urobilinogen)
- **Retention samples for viral safety laboratory parameters** will be taken.
- **Samples for virus serology laboratory parameters** (hepatitis B, hepatitis C, HIV) will be taken.

- **Transfusion products** (allogenic blood products) will be documented.
- **Concomitant medication or treatment**: type of concomitant medication/treatment, dose/schedule, duration, and the indication the concomitant medication/treatment was given for will be documented continuously up to Day 36 (+35).
- **AEs** will be recorded continuously up to Day 36 (+35).

Further details on the visit schedule that will be used for the assessment of the efficacy and safety parameters in this study are presented in section III, Flowchart of Study.

7.2 Duration of the Clinical Study

First Subject in (planned)	Q1 2018
Last Subject Last Visit *(planned)*	tbd

Individual Subject

Regular duration of individual study participation for eligible screened subjects is at least 5 weeks plus time between screening (signed ICF) and surgery.

Each subject fulfilling the intra-operative inclusion criteria will be administered BT524 or cryoprecipitate during surgery. The usual stay in hospital after elective surgery varies depending on the type of surgery and subjects overall conditions. Each subject will have follow-up visits 1, 2, 4 and 7 days after surgery (Day 2, 3, 5 and 8) and the day of discharge from hospital will be recorded.

Subsequent, each subject has to attend a closing visit including the final safety examination, and a safety follow-up at least 5 weeks after surgery, scheduled on Day 36. Using a phone call for the closing visit Day 36 is acceptable if all tests and procedures scheduled for Day 36 requiring an in-person visit, were performed on the day of hospital discharge.

The closing visit can be postponed up to Day 71 (Day 36 +35) if required due to subject's availability (e.g., stay in rehabilitation center).

Subjects not meeting inclusion criteria or meeting exclusion criteria are screen failures and will end study participation at the day of screen failure. Subjects that do not meet the intra-operatively inclusion criterion are also considered as screen failure and will end study participation on the day of the surgery.

A subject is considered to have completed the study when he/she is presumed to have followed the protocol (i.e., completed visits approximately 5 weeks after surgery). If for any reason, a subject discontinues involvement in the study early, every effort should be made to ensure the subject attends a closing visit (section 4.4).

For each subject date and reason for the end of the study participation will be recorded.

7.2.1 End of Study

The end of clinical study will be defined as the Last Visit of the Last Subject.

7.3 Criteria for Premature Termination

7.3.1 Premature Termination of the Entire Clinical Study

The clinical study as a whole may be stopped by Biotest after consultation with the Coordinating Investigator if there are reasons for which continuation of clinical study is no longer justified, such as:

a) Unacceptable delay of study completion
b) Low recruitment rate
c) A large number of subjects with premature termination
d) Changed benefit-risk ratio according to the efficacy and/or safety results from this or parallel studies
e) Lack of efficacy

f) Recently emerged information suggest that the study population can be offered a more advantageous study design

In case of premature termination of the entire clinical study, the sponsor has to notify the appropriate Ethics Committees and Regulatory Authorities as soon as possible but at the latest within 15 days.

7.3.2 Premature Termination of an Individual Study Site

The clinical study may be stopped at an individual study site for reasons such as:

a) Determination of unexpected, significant, or unacceptable risk to subjects.
b) Low recruitment rate,
c) Lack of co-operation,
d) Severe deviations from study protocol,
e) Manipulation of study data,
f) Violation of other ethical or legal principles.

7.4 Treatment and Care after the End of the Study

For subjects who have finished the clinical study and for all subjects who drop out prematurely it is the responsibility of the investigator to choose adequate therapeutic measurements.

After termination of the clinical study, any unexpected safety issue that changes the benefit-risk evaluation and is likely to have an impact on the subjects who have participated in it, should be reported as soon as possible to the sponsor. Sponsor expeditiously reports the event to the competent authority(ies) concerned (see also section 9.3.4).

8 BENEFIT-RISK EVALUATION

8.1 Benefit of BT524

Fibrinogen plays a critical role in achieving and maintaining hemostasis in patients undergoing major surgeries and the observation that patients with higher fibrinogen levels experience fewer bleeding complications than those with low levels highlights the importance of fibrinogen in the maintenance of hemostasis. Consequently, fibrinogen replacement therapy targeting a high-normal level of plasma fibrinogen and fibrin-based clot formation may be an important first step in restoring hemostasis during major bleeding.

Major spinal and abdominal surgeries represent elective surgeries often associated with significant intra- and post-operative blood loss resulting in decreased fibrinogen levels. An accurate and rapid determination of (functional) fibrinogen level is important during hemorrhage to establish a timely hemostatic intervention. Individualized dosing of fibrinogen concentrate using a target ROTEM/FIBTEM value integrates rapid diagnostic testing with appropriate therapeutic dosing of fibrinogen concentrate according to patients' needs.

The principal benefit for the participating subjects randomized to the BT524 treatment arm will be to receive the required individualized fibrinogen replacement therapy in a goal-directed treatment algorithm as a timely hemostatic intervention if significant bleeding is accompanied by acquired hypofibrinogenaemia. Moreover, fibrinogen concentrate seems to have certain advantages over the standard replacement therapy with FFP/cryoprecipitate, such as precisely determined, high amount of purified fibrinogen dissolved in a small volume, low risk of pathogen transmission and instant administration without need for thawing or testing AB0 blood group compatibility.

8.2 Foreseeable Risk and Discomfort Related to BT524

At that point of time the pre-clinical and clinical study data are still considered too scarce to finally define risks as "identified" in the context of BT524. Therefore at present all signals as derived from class-labelling, currently remain to be regarded as potential risks. Please also refer to section 6.11 Warnings and Precautions.

8.3 Other Sources of Possible Risk and Discomfort

Please refer to section 6.11 Warnings and Precautions.

8.4 Summary of Possible Risk and Discomfort

Human fibrinogen concentrate is a well-known substance, established for decades in the treatment of congenital fibrinogen deficiency as well as of various indications of acquired fibrinogen deficiency. Fibrinogen concentrates have shown to be safe and well-tolerated (Fenger-Eriksen et al., 2009; Henselmans et al., 1999).

A Guideline for Core SmPC for Human Fibrinogen Products (EU Core SmPC) is in place which also describes the well established benefit-risk profile of the authorized fibrinogen concentrates (EMA (Committee for Medicinal Products for Human Use), 2015).

It provides the following AEs as undesirable effects considered established for all authorized and marketed human fibrinogen concentrate formulations: In the MedDRA SOC "Immune system disorders": Allergic or anaphylactic-type reactions; in the MedDRA SOC "Vascular disorders": Thromboembolic episodes (including myocardial infarction

and pulmonary embolism); and in the MedDRA SOC "General disorders and administration site conditions": Increase in body temperature.

Currently, there is no data with human fibrinogen products in regard to inhibitor formation.

With regard to transmissible agents refer to section 6.11 Warnings and Precautions.

The safety profile of BT524 is anticipated to be in line with the marketed fibrinogen concentrates and the content of the EU Core SmPC [Guideline on Core SmPC for Human Fibrinogen Products (EMA/CHMP/BPWP/691754/2013 Rev 1)].

The safety and tolerability and the benefit-risk profile of BT524 (fibrinogen concentrate from human plasma) is considered favorable.

9 ASSESSMENT OF OBJECTIVES / CRITERIA FOR EVALUATION

9.1 Efficacy

9.1.1 Specification of Efficacy Parameters

9.1.1.1 Primary Efficacy Parameter

The primary efficacy parameter is determined from the intra-operative blood loss after the decision to treat the subject with IMP until the end of the surgery as measured by the amount of blood from the blood suction unit and the amount of blood from swabs, surgical cloths and compresses.

9.1.1.2 Secondary Efficacy Parameter

Secondary efficacy will be determined using the following parameters:

- Proportion (%) of subjects with successful correction of fibrinogen level (FIBTEM A10) 15 minutes after start of first IMP administration
- Time to first successful correction of fibrinogen level (15 minutes or 90 minutes after start of first IMP administration, end of surgery, not within surgery)
- Total amount (volume in mL and number of units) of transfusion products (allogenic blood products) or autologous blood transfusion infused after start of first IMP administration until end of surgery
- Amount (volume in mL and number of units) of RBCs (allogenic and autologous) infused after start of first IMP administration until end of surgery
- Post-operative blood loss in the first 24 hours
- Proportion (%) of subjects with rebleeds after the end of surgery until Day 8
- Hospital length of stay after surgery
- In-hospital mortality

9.1.2 Methods for Assessing and Recording Efficacy Parameters

9.1.2.1 Methods for Assessing Primary Efficacy Parameter

Amount of Blood Loss

The blood loss will be quantified by measuring the continuous bleeding mass with a **blood suction unit** and by calculation of the amount of blood from **swabs**, **surgical cloths and compresses** (see section 3).

The **blood suction unit** will be used to remove fluid and blood from the area being operated. Allowance must be made also for the presence of **mucinous ascites.** After opening and inspection of the abdomen the inside of the abdominal cavity will be washed out with a certain amount of **irrigation solution** (physiological saline solution) to remove mucinous ascites.

Prior to the start of surgery the weight of **dry swabs, surgical cloths and compresses** will be recorded (weighing of swabs is required if weight is not specified on the packaging). In case a **manual compression** is necessary during surgery, dry swabs, surgical cloths and compresses will be applied to the surgical field, afterwards removed and wrung out until almost dry. By wringing out surgical cloths, the blood can be caught in a kidney dish and finally collected in the suction container. Subsequently, all cloths and compresses will be weighed (before they dry out) and the weight will be recorded.

For the primary endpoint the amount of blood loss will be measured from the time of decision to treat the subject with IMP until the end of the surgery:

Immediately after the prediction of an intra-operative clinically relevant bleeding of more than 2 L, and the assessment that hemostatic treatment will be required during surgery (high risk for the need of fibrinogen supplementation, either with cryoprecipitate or BT524 during surgery), the person responsible for randomization will be informed about the decision to treat the subject with IMP. At the same time, the **suction container will be emptied and the blood measurement for the primary endpoint is initiated** with collection of blood in the empty blood suction unit.

In addition, in case a manual compression is necessary after decision to treat the subject with IMP, **new** swabs, surgical cloths and compresses will be applied to the surgical field.

At the end of the surgery, all **swabs, surgical cloths and compresses** will be removed from the surgical field, wrung out (blood will be collected in the suction container) until almost dry and weighed. The weight will be recorded. The end of surgery is defined as time of last suture.

The **fluid volume** collected in the blood suction container will be measured and documented.

The amount of **irrigation solution** suctioned into the container through irrigation of the wound must be calculated. This can be done by knowing the capacity of the irrigation syringe in use and keeping track of the number of times it is used. The calculated amount of irrigation solution must also be recorded.

The amount of irrigation solution will be subtracted **from the entire fluid volume in the suction container** to determine the actual amount of blood collected in the suction container.

In a final step the **total blood loss will be calculated**. The calculation of blood loss based on the volume of blood removed from the surgical field by the blood suction unit and that absorbed by swabs, surgical cloths and compresses.

9.1.2.2 Methods for Assessing Secondary Efficacy Parameter

Correction of Fibrinogen Level via FIBTEM A10 (mm)

The correction of the fibrinogen level will be measured via thromboelastometry (ROTEM/FIBTEM A10). ROTEM is an established viscoelastic method for hemostasis testing in whole blood and ROTEM/FIBTEM that measures the fibrin or fibrinogen contribution to clot strength, can be used to determine the most appropriate therapeutic dose of fibrinogen concentrate (Levy et al., 2014).

Whole blood viscoelastic tests such as the fibrin-based thromboelastometry (ROTEM) test FIBTEM will be used in the intra-operative setting to quickly identify deficits in fibrin quality, and to guide hemostatic therapy. In contrast to conventional laboratory tests (measurement of fibrinogen concentration via Clauss assay), ROTEM/FIBTEM can measure early variables describing the clot firmness, such as clot amplitude obtained after 5 minutes (A5) or 10 minutes (A10), and provide a forecast on the expected MCF value at an earlier stage already. These early variables allow for a more rapid decision about therapeutic interventions.

The fibrinogen activity will be measured by FIBTEM A10, MCF and Clauss assay at screening, prior surgery (baseline), pre-dose, 15 and 90 minutes after the start of first IMP administration, at the end of the surgery and one day after surgery.

For the evaluation of the secondary efficacy endpoint successful correction of the fibrinogen level is defined as restoring fibrinogen FIBTEM A10 baseline levels measured by ROTEM 15 minutes after start of first IMP administration.

Accordingly, the time to first successful correction of fibrinogen level is defined as the time point (15 minutes or 90 minutes after start of first IMP administration, end of surgery, not within surgery) the fibrinogen FIBTEM A10 baseline levels measured by rotational thromboelastometry are restored the first time.

However, correction of fibrinogen will also be analysed based on results obtained from MCF and Clauss assay to perform sensitivity analyses.

Clauss Assay

The most widely used technique for determination of fibrinogen concentration is the Clauss (FIBClauss) assay (Clauss, 1957). In this method, dilutions of a plasma standard of known fibrinogen concentration are clotted by addition of a high concentration of thrombin, and a standard curve is prepared. Because the clotting time is inversely proportional to the fibrinogen concentration, the clotting time of diluted patient plasma is used to read the fibrinogen concentration from the standard curve. This method is reliable, accurate, and precise, and easily adapted to automated coagulation analyzers.

Maximum Clot Firmness (MCF, mm)

MCF addresses the clot integrity and will be measured by ROTEM® (ROTEM delta or ROTEM sigma).

MCF will be determined locally from whole blood or at the central laboratory by means of the Fib-tem S assay (tissue factor activation and platelet inhibition), a ready-to-use ROTEM system reagent (PPD, Mar 2011) for the ROTEM delta/sigma which allows the assessment of the fibrinogen level and the quality of the fibrin polymerization in citrated blood by inhibiting the platelets. For ROTEM sigma the FIBTEM C system reagent will be used accordingly.

Fibtem measures the viscoelastic properties of the clot and provides information on the speed of coagulation initiation, kinetics of clot growth (MCF, mm), clot strength, and breakdown(Lang et al., 2009). **Figure 4** shows an example of the ROTEM readout of citrated normal blood.

Figure 4: ROTEM Readout of Citrated Normal Blood

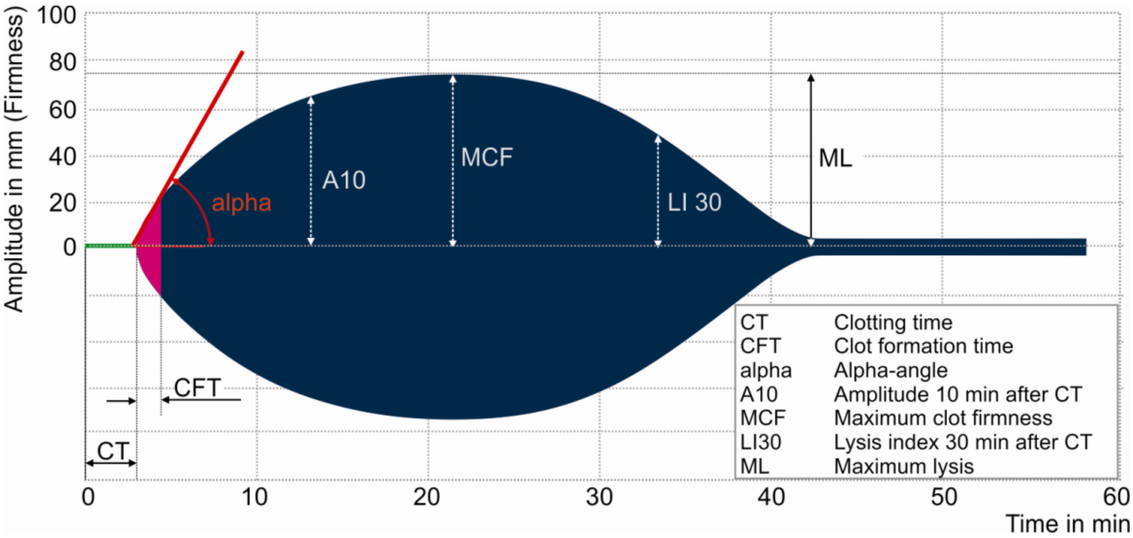

The ROTEM® analyses: FibTEM® test (fibrin clot obtained by platelet inhibition with cytochalasin D). The clotting time (CT (seconds)) represents the time from the start of the test until a clot firmness of 2 mm is detected; maximum clot firmness (MCF (mm)) represents the total amplitude of the clot (Schöchl et al., 2010).

In the ROTEM read out of Fib-tem S test expected reference (normal range) values for the amplitude of MCF are 9-25 mm for fibrinogen levels (Fib-tem S 2011).

Consumption of Transfusion Products

The **amount of transfusion products** (allogenic blood products or autologous blood transfusion) given after start of first IMP administration until end of surgery to counteract hemodynamic instability will be documented (volume in mL and number of units if applicable) and evaluated.

The **amount of RBCs** (allogenic and autologous) required intra-operatively after start of first IMP administration until end of surgery will be documented (volume in mL and number of units if applicable) and evaluated.

Postoperative Blood Loss (Drainage Volume)

The plasma drainage tube and disposable drainage bag will be emptied 24 hours after the end of the surgery. The end of surgery is defined as time of last suture. The drainage volume will be measured and postoperative blood loss will be recorded.

Rebleeds

Any bleed judged by the surgeon and/or the anaesthesiologist as requiring hemostatic treatment (including reoperation) after the end of the surgery until follow-up Day 8 will be recorded. The end of surgery is defined as time of last suture.

Hospital Length of Stay after Surgery

The day of the surgery and the day of the discharge from hospital will be recorded.

The hospital length of stay after surgery is used to compare the number of days patients stayed in the hospital after surgery. The hospital length of stay after surgery is calculated by the following formula:

Length of stay after surgery = date of discharge - date of surgery

In-hospital Mortality

In-hospital mortality is defined as death occurring during the hospital stay. A death during hospitalization will be recorded as an SAE.

9.1.3 Specification of Efficacy Endpoints

9.1.3.1 Specification of Primary Efficacy Endpoint

Intra-operative blood loss as measured by amount of blood from the blood suction unit and amount of blood from swabs, surgical cloths and compresses after the decision to treat the subject with IMP until end of surgery. The end of surgery is defined as time of last suture.

9.1.3.2 Specification of Secondary Efficacy Endpoints

- Proportion (%) of subjects with successful correction of fibrinogen level 15 minutes after start of first IMP administration.

  Successful correction of fibrinogen level in a subject is defined as restoring fibrinogen FIBTEM A10 baseline level to at least 95% measured by ROTEM 15 minutes after start of first IMP administration. A correction of at least 95% is considered successful, as measurement methods has a coefficient of variation of about 5% (Solomon et al., 2015).
- Time to first successful correction of fibrinogen level (15 minutes or 90 minutes after start of first IMP administration, end of surgery, not within surgery).
- Total amount of transfusion products (allogenic blood products) or autologous blood transfusion infused after start of first IMP administration until end of surgery. The end of surgery is defined as time of last suture.

- Amount of RBCs (allogenic and autologous) infused after start of first IMP administration until end of surgery. The end of surgery is defined as time of last suture.
- Post-operative blood loss in the first 24 hours.
- Proportion (%) of subjects with rebleeds.
  Rebleeds are defined as any bleed requiring hemostatic treatment (including reoperation) after the end of surgery until Day 8.
- Hospital length of stay after surgery, defined as the date of discharge minus the date of surgery.
- In-hospital mortality.

9.2 Safety

9.2.1 Specification of Safety Parameters

Safety and tolerability in this clinical study will be addressed by the following safety parameters:

- AEs
- Changes in vital signs (including pulse [heart rate], blood pressure, respiratory rate, body temperature)
- Change in clinical laboratory assessments of hematology, clinical chemistry and urinalysis.
- Change in clinical laboratory assessments of markers of coagulation:
  PT(INR), aPTT, TAT, F_{1+2},D-dimer, PS, PC, AT III, TT, TGT[3]
- Change in clinical laboratory assessments of coagulation factors:
  FII, FV, FVII, FVIII, FIX, FX, FXI, FXIII, and vWF[2]
- Frequency and severity of thrombosis and of TEEs
- Viral status.

For the timing of individual safety parameters refer to section 7.1 Visit Schedule and section III FLOWCHART OF STUDY. Any abnormal observation or finding detected during the screening procedures after subject's informed consent that is considered clinically relevant must be documented as concomitant disease (ongoing medical history) or as past medical history. All medications other than the study medications should also be documented.

9.2.1.1 Adverse Events

After informed consent has been signed, AE data are obtained by the investigator through observation of the subject (including examinations and investigations) before, during and after surgery, from any information volunteered by the subject, and through active questioning. At each visit, subjects will be asked about AE that occurred since the last visit, by questioning them with regard to their well-being by ‘non-leading’ questions. All AEs should be recorded and reported to safety department (see section 9.3.2). This includes AEs occurring after informed consent has been signed (before, during or after surgery), as well as changes in concomitant diseases (i.e., ongoing medical history).

[3] Only applicable for subjects undergoing cytoreductive surgery for PMP

Occurrence, frequency, nature and severity of AEs will be recorded. This includes observations or abnormalities in physical examination, vital signs, laboratory or other investigations reported as AEs. Any treatments for AEs will also be recorded.

For further details regarding AEs including definitions and reporting procedures refer to Appendix 1: Safety Definitions.

9.2.1.2 Physical Examination

For each subject, a complete physical examination will be performed at the time points specified in III Flowchart of Study (before and after surgery). Physical examination includes inspection of general appearance, skin, neck (including thyroid) eyes, ears, nose, throat, heart, lungs, abdomen, lymph nodes, vascular system, extremities, musculoskeletal system and nervous system. Clinical findings and existing diseases at screening are to be documented as medical and surgical history. A new appearance of an abnormal finding or worsening of a concomitant disease that occurs after signature of informed consent must be documented as AE.

- BW is measured in kilograms (kg) only at screening visit and at baseline.
- Body height is measured in cm once at screening visit for the purpose of calculation of BMI.

9.2.1.3 Vital Signs

Before and after surgery (Screening, baseline, follow-up Day 2 to Day 36) vital signs are measured with the following methods/units:

- Blood pressure is measured in mmHg according to the Riva Rocci method while the subject has been resting in supine position for at least 15 minutes. The same arm should be used for blood pressure measurements throughout the study. The size of the cuff has to be chosen appropriately in relation to the subject's arm circumference.
- Heart rate and pulse is measured in beats per minute (either electronically and/or by palpation for 1 minute) while the subject is supine and has been resting for at least 15 minutes. When heart rate is of concern, cardiac monitors are used to determine not only rate, but also rhythm.
- Respiratory rate per minute.
- Body temperature is measured in °C.

<u>During surgery (Day 1):</u> Surgery/anaesthesia standard methods should be used for monitoring of vital parameters including standard measurement methods for blood pressure, pulse, heart rate and rhythm, respiratory rate and temperature. These monitoring results are to be reviewed by the investigator, under consultation with a specialist if necessary.

Results must be summarized in writing and classified as 'normal' or 'abnormal'. Abnormal monitoring results must in addition be classified as 'abnormal, clinically not relevant' or 'abnormal, clinically relevant'. Abnormal, clinically relevant findings have to be reported as AE (unless already pre-existing at baseline with the same severity).

In addition, vital signs will be assessed and recorded in the eCRF at the following time-points: prior IMP treatment, 15 and 90 min after start of first IMP administration and end of surgery.

Measurements outside the normal range (according to the age and gender of the subject) or even changed values within the normal range showing a trend have to be assessed for clinical relevance by the investigator, and reported as AE if considered to represent a clinically significant change as compared to pre-treatment values.

9.2.1.4 Laboratory Parameters

All laboratory results have to be evaluated either in the eCRF or in the web-base tool of the central laboratory by the investigator/subinvestigator (anaesthesiologist) according to the following pattern:

a) Within reference range (normal range)
b) Outside reference range but not clinically relevant (e.g. marginal deviation only, due to underlying medical history diseases in the study population)
c) Outside reference range and clinically relevant

Laboratory values occurring (by date of blood sampling) after signature of informed consent, which are outside the reference range and assessed as clinically relevant (as determined by investigator), have to be documented as AE.

Postoperative elevation of liver function tests (LFTs) in patients undergoing cytoreductive PMP surgery are expected due to the specific surgical intervention and are therefore not assessed as clinically relevant and not documented as an AE. The exception to this is if a therapy is required, in which case an AE should be recorded. LFTs include the liver enzymes alanine aminotransferase [ALT], aspartate aminotransferase [AST], gamma-glutamyltransferase [γ-GT], alkaline phosphatase [AP], and Bilirubin.

An abnormal laboratory value that is a sign of an AE (e.g. increased leukocytes due to bacterial infection) that has already been reported during the present clinical study, has to be documented as a symptom together with the diagnosis (bacterial infection) and does not constitute a separate AE.

For the routine laboratory parameters, the total volume of blood that will be drawn from a subject who completes the study is defined in the Laboratory Manual.

The laboratory parameters to be assessed in subjects undergoing cytoreductive PMP surgery are summarized in Table 1:

Table 1: Clinical Laboratory Parameters

Clinical Laboratory Parameters	Assessment to be done locally or centrally
**Clinical Chemistry**	
• Alanine aminotransferase (ALAT)<br>• Aspartate aminotransferase (ASAT)<br>• Gamma-glutamyltransferase (ɣ-GT)<br>• Alkaline phosphatase (AP)<br>• Bilirubin (direct and indirect if total bilirubin is elevated)<br>• Creatinine / creatinine clearance<br>• Blood Urea Nitrogen (BUN) or Urea (UREA)<br>• Sodium<br>• Chloride<br>• Potassium<br>• Calcium	• locally<br>• locally<br>• locally<br>• locally<br>• locally<br>• locally<br>• locally<br>• locally<br>• locally<br>• locally<br>• locally
**Hematology**	
• Hematocrit<br>• Hemoglobin<br>• Red blood cells (RBCs)<br>• White blood cells (WBC)<br>• Differential white blood cells<br>• Platelets	• locally<br>• locally<br>• locally<br>• locally<br>• locally<br>• locally
**Coagulation**	
**Markers of Coagulation**<br>• Prothrombin time (PT) / international normalized ratio (INR)<br>• Activated partial thromboplastin time (aPTT)<br>• Thrombin-antithrombin III complex (TAT)<br>• Prothrombin fragments (F_{1+2})<br>• D-Dimer<br>• Protein S (PS)<br>• Protein C (PC)<br>• Antithrombin III (AT III) activity<br>• Thrombin Time (TT)<br>• Thrombin Generation Test (TGT)<br><br>**Coagulation Factors (including vWF)**<br>• von Willebrand factor (vWF)[3]<br>• Factor I (activity of fibrinogen, Clauss assay)<br>• Factor I (ROTEM: FIBTEM A10, Maximum clot firmness)<br>• Factor II (FII)<br>• Factor V (FV)<br>• Factor VII (FVII)<br>• Factor VIII (FVIII)<br>• Factor IX (FIX)<br>• Factor X (FX)<br>• Factor XI<br>• Factor XIII (FXIII)	 • locally<br>• locally<br>• centrally<br>• centrally<br>• centrally<br>• centrally<br>• centrally<br>• centrally<br>• centrally<br>• locally<br><br><br>• centrally<br>• centrally<br>• locally<br>• centrally<br>• centrally<br>• centrally<br>• centrally<br>• centrally<br>• centrally<br>• centrally<br>• centrally

Clinical Laboratory Parameters	Assessment to be done locally or centrally
**Urinalysis**	
• pH<br>• Qualitative for blood<br>• White blood cells (WBC)<br>• Protein<br>• Glucose<br>• Ketone bodies<br>• Bilirubin<br>• Urobilinogen<br>• Nitrites	• locally<br>• locally<br>• locally<br>• locally<br>• locally<br>• locally<br>• locally<br>• locally<br>• locally
**Pregnancy Test**	
• Human chorionic gonadotropin (hCG)	• locally
**Virus Serology**	
• HIV<br>• Hepatitis B<br>• Hepatitis C	• locally<br>• locally<br>• locally
**Viral Safety**	
• Retention sample for viral safety laboratory parameters	• centrally

The following **markers of coagulation** will be assessed at the time points specified in section III, Flowchart of clinical study:

- Prothrombin Time (PT) / International Normalized Ratio (INR)

  PT is a measure of the integrity of the extrinsic and final common pathways of the procoagulant cascade. PT presents the time for patient plasma to clot after the addition of calcium and thromboplastin as an activator of the extrinsic pathway. Therefore, deficiencies or inhibitors of clotting factors within the extrinsic (factor VII) and final common pathways (factors V, X, II, I [fibrinogen]) result in prolongation of the PT.

  The INR is a mathematical conversion of a patient's PT that accounts for the sensitivity of thromboplastin used by factoring in the international sensitivity index (ISI) value supplied by its manufacturer (Kamal et al., 2007).

- Activated Partial Thromboplastin Time (aPTT)

  aPTT measures the integrity of the intrinsic and final common pathways of the coagulation cascade and represents the time for patient plasma to clot after the addition of phospholipid (intrinsic pathway activator) and calcium (Kamal et al., 2007).

- Thrombin-Antithrombin III Complex (TAT)

  TATs develop during the inactivation of thrombin – the central enzyme of the coagulation system – via complexion with anti-thrombin. TATs are also an indirect measure of thrombin generation. In combination with F_{1+2} a hypercoagulable state can be detected (Wagner C, 2008).

- Prothrombin Fragments 1 and 2 (F1+2)

  During the activation process of prothrombin to thrombin F_{1+2} are split off and represent an indirect measure of thrombin generation. They are useful to detect

and follow-up a hypocoagulable state (Wagner C, 2008).

- D-dimer

  D-dimer is a fibrin degradation product, a small protein fragment present in the blood after a blood clot is degraded by fibrinolysis. D-dimer contains two crosslinked D fragments of the fibrinogen protein. D-dimers are produced when fibrin is cleaved by plasmin. The presence of D-dimer may be used to assist with the diagnosis of DIC, Deep Venous Thrombosis (DVT) or Pulmonary Embolism (PE).

- Antithrombin III (AT III) Activity (functional ATIII level)

  Antithrombin is a glycoprotein and is a natural anticoagulant that inhibits the activated coagulation factors thrombin (factor IIa), factor Xa, and, to a lesser extent, factor XIa and factor IXa. Heparin significantly increases the inhibition rate. The antithrombin level does not influence the results of screening coagulation tests such as partial thromboplastin time (PTT), PT, and TT (Teruya and Kostousov, Updated: Jan 30, 2014. Access Date: 15-Jun-2016.).

- Thrombin Time (TT)

  TT is a screening coagulation test designed to assess fibrin formation from fibrinogen in plasma. TT is performed as the next step in the evaluation of abnormally prolonged aPTT or PT (Rodgers and Lehman, 2007).

- Protein C (PC)

  PC is an inactive protein. When activated, it plays a significant part in blood clot, inflammation, and cell death regulation, as well as in maintenance of blood vessel cell wall permeability. The inactive form of PC circulates in blood plasma and is a vitamin K–dependent glycoprotein. When inactive PC binds to thrombin, it becomes activated (Beckmann et al., 1985; Foster et al., 1985; Mather et al., 1996).

  PC has a crucial role as an anticoagulant, and individuals with a PC deficiency or with some type of P activation dysfunction are at much greater risk of thrombosis (Beckmann et al., 1985).

- Protein S (PS)

  PS is a vitamin K-dependent plasma glycoprotein. The two forms of PS are a free form that is active and a complex form (65%) that is inactive (Lundwall et al., 1986).

  PS plays a significant role in the anticoagulation cascade, where it functions as a cofactor to the serine protease activated PC in the inactivation of factors Va and VIIIa. Through direct binding to factors Va, Xa, and VIII, PS exerts activated PC–independent anticoagulant activity (Francis, 1988). If the amount of PC or PS is inadequate or if either one is not functioning properly, thrombin generation essentially remains undeterred, which may promote inappropriate or excessive clotting, with resultant blockage of blood flow in the veins and, rarely, the arteries (thrombosis) (Esmon, 2003; Mosnier et al., 2007). (Bonhomme and Fontana, 2015; Castoldi and Rosing, 2011)

- Thrombin Generation Test (TGT)

  The thrombin generation test is a global assay that measures the overall tendency of a plasma sample to form thrombin after initiation of coagulation. TGT measures the quantity of thrombin produced in response to a calibrated stimulus. It is performed on plasma, with or without platelets. The amount of thrombin reflects

the overall functioning of the hemostatic system (activators and inhibitors), without assessing fibrinolysis (Bonhomme and Fontana, 2015). Although several studies have shown a correlation between thrombin generation and the risk of bleeding or venous thrombosis, the application of thrombin generation assays to clinical decision-making is still hampered by standardisation problems (Castoldi and Rosing, 2011).

The following **coagulation factors** (including von vWF) will be assessed at the time points specified in section III, Flowchart of clinical study:

- Factor II (FII)

  Clotting factor II, or prothrombin, is a vitamin K–dependent proenzyme that functions in the blood coagulation cascade (Harel et al., 2016).

- Factor V (FV)

  Factor V is an essential component in the blood coagulation cascade. The factor V protein is a catalyst, accelerating the process by which prothrombin is converted to thrombin, the initial step in clot formation.

- Factor VII (FVII)

  Factor VII is a vitamin K-dependent serine protease glycoprotein. The physiological activator of factor VII is thought to be factor Xa. The factor VIIa/tissue factor complex activates both factors IX and X (Roberts et al., 2001).

- Factor VIII (FVIII)

  Factor VIII (antihemophilic factor) is a key factor of the intrinsic clotting cascade. Normal hemostasis requires at least a quarter (25%) of factor VIII activity (Bishnu Prasad Devkota, Updated: Jan 17, 2014. Access Date: Jun 15, 2016.).

- Factor IX (FIX)

  Factor IX, or Christmas factor, is one of the serine proteases of the coagulation system. Factor IX can be activated either by factor XIa or by the factor VIIa/tissue factor complex. In complex with its cofactor, factor VIIIa, factor IXa activates factor X (Roberts et al., 2001).

- Factor X (FX)

  Clotting factor X, or Stuart-Prower factor, is a vitamin K–dependent serine protease that serves as the first enzyme in the common pathway of thrombus formation (Schwartz, Updated: Mar 01, 2017. Access Date: 15-Mar-2017.).

- Factor XI (FXI)

  Factor XI or plasma thromboplastin antecedent is the zymogen form of factor XIa, one of the enzymes of the coagulation cascade. It is a serine protease. Deficiencies of factor XI may lead to a bleeding tendency reflecting the significant role of factor XI in hemostasis (Roberts et al., 2001).

- Factor XIII (FXIII)

  Factor XIII (FXIII), which was initially termed fibrin stabilizing factor, is involved in clot preservation (Shanbhag et al., 2016). Thrombin, generated by reactions initiated by activated tissue factor VII/factor IX pathways, leads to clot formation. Fibrin monomers polymerize spontaneously; this is followed by development of a complex branching clot as a result of the actions of activated FXIII (FXIIIa) (Andersen et al., 2009; Casadio et al., 1999; Fox et al., 1999). Several controls in

the complex activation process focus the actions of FXIIIa on fibrin rather than on fibrinogen. Cross-linking of polymerized soluble fibrin by FXIIIa is the final step in hemostasis (Lorand, 2000; 2001). (Sadler, 1998; Shahidi, 2017; Taylor, 2015).

- Von Willebrand factor (vWF)

  von Willebrand factor is a blood glycoprotein involved in hemostasis. vWF performs two essential functions in hemostasis: it mediates the adhesion of platelets to subendothelial connective tissue, and it binds blood clotting factor VIII (the protein missing in hemophilia A). In the absence of vWF, factor VIII is rapidly removed from the circulation. Consequently, patients who lack vWF have a severe bleeding disorder (von Willebrand disease) because they have profound defects both in blood clotting and in the formation of platelet plugs at sites of vascular injury. von Willebrand factor (vWF) is also involved in fibrin clot formation through its role as a carrier protein for factor VIII (Sadler, 1998; Taylor, 2015).

  Increased plasma levels in many cardiovascular, neoplastic, and connective tissue diseases are presumed to arise from adverse changes to the endothelium, and may predict an increased risk of thrombosis (Shahidi, 2017).

In addition, the following specific safety laboratory parameters will be assessed at the time points specified in section III, Flowchart of clinical study:

- Pregnancy Test

  In females of childbearing potential, a pregnancy human chorionic gonadotropin test in serum will be performed at screening as specified in section III, Flowchart of clinical study. In addition, a second pregnancy test in urine (or serum) will be performed at baseline as specified in section III, Flowchart of clinical study. Pregnancy tests will be performed in the **local laboratory**.

- Virus Serology

  The immunological status of viral infections will be assessed at screening and closing visits for HIV, HBV and HCV, section III, Flowchart of clinical study. The following tests will be performed in the **local laboratory**:

  - **HIV** (anti HIV1 and anti HIV2)
  - **hepatitis B** (total hepatitis B core antibody [anti HBc], hepatitis B surface antibody [anti HBs], hepatitis B surface antigen [HBsAg])
  - **hepatitis C** (anti HCV)

9.2.1.5 Retention Samples

In order to respond rapidly to any reports on additional viral infections, a pre-treatment serum sample (5 mL) from each subject included in the study must be taken pre-dose and stored at -70 °C for possible future testing (screening visit).
At closing visit (Day 36) an additional serum sample (5 mL) must be taken and stored up to 6 months after study end.
Viral safety retention samples will be analysed in the **central laboratory** (if applicable).

9.2.2 Methods for Assessing and Recording Safety Parameter(s)

The following safety parameters are recorded in the eCRF: AEs, vital signs (including pulse [heart rate], blood pressure, respiratory rate, body temperature), clinical laboratory values of hematology, clinical chemistry and urinalysis. Clinical laboratory assessments of markers of coagulation and coagulation factors: PT(INR), aPTT, TAT, F1+2, D-dimer, PS, PC, AT III, TT, TGT, FII, FV, FVII, FVIII, FIX, FX, FXI, FXIII, vWF. Virus status.

For documentation of abnormal, clinically relevant findings refer to the respective sections above: section 9.2.1.4.

The results of all analyses performed by the central laboratory PPD , PPD , will be transferred to the investigational site, CRO's Data Management, and PPD Medical Advisor in a timely manner. All laboratory analyses performed by the local laboratory using standard assay methods will be transcribed to the eCRF by the investigator also in a timely manner.

For alert processes in the situation of abnormal results refer to Laboratory Manual.

9.2.3 Safety Endpoints

The following variables are defined as secondary safety endpoints:

- AEs
- Changes in vital signs
- Changes in clinical laboratory assessments of hematology, clinical chemistry, and urinalysis
- Changes in clinical laboratory assessments of markers of coagulation
- Changes in clinical laboratory assessments of coagulation factors
- Frequency and severity of thrombosis and of TEEs
- Virus status

9.3 Adverse Events

9.3.1 Definitions

(also refer to Appendix 1: Safety Definitions)

- **Adverse Event (AE)**
  Any untoward medical occurrence in a patient or clinical study subject administered an IMP and which does not necessarily have a causal relationship with this treatment. An AE may be any aggravation or new unfavorable and unintended sign, symptom, or disease temporally associated with the use of an IMP, whether or not considered related to the IMP (ICH Guideline for Good Clinical Practice E6(R2)).

- **Adverse drug reaction of an investigational medicinal product (ADR):**
  All untoward and unintended responses to an IMP related to any dose administered. All AEs judged by either the reporting investigator or the sponsor as having a reasonable causal relationship to a medicinal product qualify as adverse reactions. The expression reasonable causal relationship means to convey in

general that there is evidence or argument to suggest a causal relationship. All non-serious AE (related and not related) should also be entered in eCRF as soon as possible but not later than one month after occurrence.

- **Serious Adverse Event (SAE)**
  An SAE is any untoward medical occurrence or effect that at any dose*:
    - results in death
    - is life-threatening
    - requires hospitalization or prolongation of existing hospitalization
    - results in persistent or significant disability / incapacity
    - is a congenital anomaly / birth defect
    - is another important medical event

  * "At any dose" does not necessarily imply that the subject is receiving the study drug at the time of the event.

  Reporting requirements are detailed in Appendix 2.

- **Adverse Event of Special Interest (AESI)**
  An AE of special interest (serious or non-serious) is one of scientific and medical concern specific to the sponsor's IMP or development program, for which ongoing monitoring and immediate communication by the investigator to the sponsor may be appropriate. Such events may require further investigation in order to characterize and understand them. Reporting requirements are detailed in Appendix 2.

  The following AEs have been defined as AESI for this study:
    - Thrombosis or TEE
    - Relevant bleeding complication:
      Relevant changes of a vital sign or laboratory value (e.g. severe tachycardia, severe hypotension, hypovolemic shock, severe anemia) occurring after IMP administration, during and/or after surgery that require immediate corrective action/treatment and are caused by bleeding.
      Note: Corrective treatment includes e.g. the administration of unplanned blood products or other drugs.
    - Suspicion of transmission of infective agents (viral safety).

- **Immediately Reportable Adverse Event (IRAE)**
  An AE that must be reported to the sponsor **within 24 hours** of the study site being aware of the AE. Reporting requirements are detailed in Appendix 2.

  For this clinical study, IRAEs include
    - all SAEs
    - all AESIs (serious and **non-serious**)
    - all AEs that result in a subject's withdrawal from the study (including suspected allergic reaction)
    - Medication error (incl. overdose)
    - Pregnancy

- **Adverse Events Leading to Subject's Withdrawal from the Clinical Study**
  An AE, serious or non-serious, resulting in subject's withdrawal from the clinical study, i.e., permanent treatment discontinuation (see section 4.4). Reporting requirements by the investigator are detailed in section Appendix 2.

- **Medication Error**
  A medication error is an unintended failure in the drug treatment process that leads to, or has the potential to harm the patient (EMA Good Practice Guide definition).
  - A 'failure in the drug treatment process' does not refer to lack of efficacy of the drug, rather to human or process mediated failures.
  - The error is unintended. The concepts of intentional overdose, off-label use, misuse and abuse as defined in GVP (good pharmacovigilance practices) Module VI.A.2.1.2 are outside scope and should be clearly distinguished from medication errors.
  - 'Drug treatment process' includes prescribing, storing, dispensing, preparation for administration and administration of a medicine in clinical practice.

  The dose and administration of the study medication is described in section 6 Study Treatment. Any deviation from the study medication (wrong medication, wrong dose, wrong route of administration, wrong patient) is a medication error. Any higher administered dose of the study medication, than described in section 6 Study Treatment, is an overdose.

9.3.2 Recording Adverse Events

All AEs, serious and non-serious, that occur during the period of observation defined for the clinical study (section III, Flowchart of Study) have to be fully documented in the eCRF according to the provisions given in this section of the study protocol and eCRF completion guidelines, as well as in the subject's source data. This applies also to AEs in subjects who signed the informed consent but never received the study drug. AEs considered not related to study medication observed after Day 36 (defined closing visit) will not be recorded in the eCRF.

In addition, for a subset of AEs (SAE, AESI, AE leading to withdrawal) immediate reporting from investigator to sponsor is required (Immediately Reportable Adverse Events, IRAE). This is further detailed in Appendix 2. The following information is necessary:

- **Diagnosis vs. Signs/Symptoms**
  The investigator should provide a diagnosis rather than individual signs and symptoms, wherever possible and appropriate. However, if there is not enough information to provide a diagnosis, individual signs and symptoms are to be recorded. If a diagnosis is accompanied by unusual symptoms, the diagnosis itself and the unusual symptoms have to be reported separately. For serious and other IRAEs the investigator shall provide any other supporting information that may be required for the assessment of the events.

  A complication of an AE constitutes another AE. For example in diarrhea leading to dehydration, diarrhea and dehydration would be captured as separate AEs.

  The eCRF provides for a number of items to be completed for each AE. This includes the onset date, end date, intensity/severity, seriousness, action taken with

study medication, treatment for the AE, outcome, and causal relationship of the AE with the study medication, other drugs, or study procedures (Appendix 2: Reporting Procedures).

Severe vs. Serious: The severity is used to describe the intensity of an event. This is not the same as seriousness, which is based on subject/event outcome or action criteria usually associated with events that pose a threat to subject's life or functioning. Seriousness, not severity, serves as the guide for defining regulatory reporting obligations.

- **Causal Relationship of AE**

  The causal relationship with the study medication has to be reported for each AE. It refers to the presence or absence of a reasonable possibility of a causal relationship between the study medication and the AE.

  The investigator is asked to use medical judgment and take into account the nature of the AE, subject's medical and surgical history, temporal relation, response to withdrawal or interruption of study drug (dechallenge), response to re-introduction of study drug (rechallenge), any alternative explanations such as underlying or concomitant diseases, concomitant drugs, study procedures.

  The investigator should also provide the causality assessment with the NIMP (e.g. background medication, concomitant medication - see Appendix 1 for definition).

  The following categories are used:

  - Related: There is a reasonable possibility of a causal relationship between the study medication and the AE.
  - Not related: There is no reasonable possibility of a causal relationship between the study medication and the AE.

  For serious and other immediately reportable AEs the investigator is asked to specify if there are alternative and/or additional explanations for the occurrence of the event, e.g. concomitant drugs, study procedures, or concomitant/underlying disease and should provide this information already with the initial case report.

9.3.3 Period of Observation

The period of observation for collection of AEs extends from the time the subject signs the ICF until the last study visit (Day 36), which is scheduled 5 weeks after the day of the surgery (day of IMP administration).

Abnormal, clinically relevant findings or observations made prior to signature of informed consent are to be recorded as medical history/concomitant disease but not as AEs.

AEs (including any change in severity or trait of the concomitant disease/ medical history) occurring in the pre-treatment period between signature of informed consent until first administration of study medication are non-treatment emergent AEs (NTEAEs).

AEs (including any change in severity or trait of the concomitant disease/ medical history) occurring from the administration of study medication until the subject's last study visit are treatment-emergent AEs (TEAEs).

If an SAE occurs in a subject after the period of observation, i.e., after the last study visit, which is considered by the investigator to be related to the study medication, this should be recorded as SAE and follow the immediate reporting process for SAEs as described

in Appendix 2. If the eCRF has been closed for the subject, the investigator should contact the sponsor to determine how to report the SAE (see also section 7.4).

9.3.4 Assessment of Adverse Events

- **Responsibilities of Investigator**

  AEs are assessed by the investigator in a standardized manner including, but not limited to the seriousness, severity, outcome, and causality. This has to be performed in line with the definitions and provisions given in Appendix 1.

  Laboratory values outside the reference range have to be assessed for clinical relevance taking into account the pre-treatment values. For reporting of abnormal laboratory values as AEs refer to section 9.2.1.4.

  If an AE meets the definition of any of the mandatory AE related stopping rules (section 9.3.1), the investigator must withdraw the subject and report the AE as IRAE according to section Appendix 2. If no mandatory AE related stopping rules defined for the study, delete this sentence.

  For all AEs the causality assessment has to be provided in the eCRF, even if based on preliminary data. Once more information is available, the investigator may change a preliminary causality assessment.

  During and after participation of a subject in a clinical study the investigator/institution has to ensure that adequate medical care is provided to the subject for any ongoing AEs including clinically significant abnormal laboratory values. The investigator has to inform the subject when medical care is needed for any intercurrent disease of which the investigator becomes aware.

- **Responsibilities of Sponsor**

  AEs are reviewed and assessed by the sponsor during ongoing safety monitoring activities throughout the study, as well as medical evaluation and regulatory assessment for reportability of SAEs and IRAEs. For the purpose of regulatory reporting, the causality assessment given by the investigator will not been downgraded by the sponsor. If the sponsor disagrees with the investigator's causality assessment, both the opinion of the investigator and the sponsor will be recorded.

  Regulatory assessment of an AE by the sponsor comprises further the assessment of expectedness. For BT524, Fibrinogen Concentrate from Human Plasma, it is based on section 7.9 of the Investigator's Brochure (IB). For cryoprecipitate it is based on the Summary of Product Characteristics of a reference product: Reference Safety Information (RSI) for the cryoprecipitate is the section 4.8 of the SmPC of PPD solution for infusion (PPD

- **Follow-up of Adverse Events**

  AEs should be followed up to determine the outcome.

  AEs that are serious or severe or considered related to the study medication or study procedures must be followed up by the investigator until the AE is resolved or resolved with sequelae, and until all queries related to the AE have been

clarified. If the subject had an AE with fatal outcome, an autopsy report should be provided if possible.

If AEs that are serious or severe or considered related to study medication or study procedures are ongoing at the time of the subject's last study visit, or if the subject has clinically relevant laboratory parameter abnormalities at the last study visit, one or more safety follow-up visit should be scheduled for those subjects. The investigator should set the interval to the additional safety follow-up visit according to his/her medical judgment. Follow-up activities should be continued until the investigator considers it medically justifiable to stop further follow-up.

All other AEs must be followed up by the investigator until the AE is resolved or resolved with sequelae or the end of the period of observation (= last study visit), whichever comes first.

The investigator should respond to any queries raised by the sponsor in relation to AEs, including provision of supporting documentation for SAEs or other IRAEs (e.g. ECG data, laboratory results, hospital summary, autopsy report) within the requested timeline. In case of fatal or life-threatening SAEs the sponsor may request urgent clarification within one calendar day. In general, if for AEs requiring immediate reporting from investigator to sponsor (IRAE/SAE) follow-up information becomes available, this must be reported to the sponsor **within 24 h** of becoming aware of this information (i.e., the same timeframe as for initial IRAE/SAE reports). Any supporting documents have to be identified by the subject ID, and personal data (e.g. subject name, address or phone number) obliterated prior to sending to the sponsor. For details on reporting IRAE/SAE see Appendix 2.

AE data in the eCRF must be updated accordingly when follow-up information is received.

All efforts to collect follow-up information must be documented in the subject's source data.

Subjects who were treated with the study medication but did not complete the study as per protocol, should receive all the examinations and investigations scheduled for the last study visit. The investigator should make all efforts to contact subjects lost to follow-up and document the attempts in the subject's source data.

9.3.5 Immediate Reporting by Investigator to Sponsor

(also refer to Appendix 2: Reporting Procedures)

9.3.6 Use of IMP outside the Specifications of the Clinical Study Protocol

Situations may occur where the IMP is used outside the specifications of the protocol, which may or may not be associated with an AE. These special situations comprise

- Medication errors (including overdose)
- Abuse/ misuse of the IMP.

Such situations, whether or not associated with an AE, are documented in the eCRF on dedicated pages. Any AE that occurred in association with such a special situation has to be cross-referenced on the dedicated eCRF page. An IRAE/SAE occurring in conjunction with a medication error or abuse/ misuse of the IMP has to follow the

immediate reporting process for IRAE/SAE as described in Appendix 2 in addition to its documentation in the eCRF (also refer to sections 9.3.8 and 10.2).

9.3.7 Investigational Medicinal Product Complaints

IMP complaints must be recorded in the eCRF and in addition reported to the sponsor on the "Investigational Medicinal Product Complaint Report Form" **within 24 hours** of the investigator becoming aware of the IMP complaint. If the IMP complaint is associated with an AE, the AE must be entered in the eCRF also.

Any complaint samples should be provided to the sponsor upon request.

9.3.8 Special Situations Requiring Immediate Reporting

Special situations may occur that may or may not be associated with AEs. For these situations special reporting provisions apply.

Special situations comprise:

- Pregnancy in a female study subject or the partner of a male study subject
- Investigational Medicinal Product Complaint.
- Use of IMP outside the specifications of the CSP (e.g. Medication errors, overdose, misuse and abuse (also refer to sections 9.3.6 and 10.2).
- Protocol deviations (refer to section 10.2)

If such a situation occurs, the investigator should contact the sponsor immediately. A special paper report form has to be completed always in these situations and sent to the sponsor immediately, but not later **than 24 hours** after the investigator becoming aware of the situation.

9.3.8.1 Pregnancy

Pregnant women are excluded from the study, and female study subjects of child-bearing potential undergo pregnancy testing at screening and regularly during the study (section III, Flowchart of study). If pregnancy is suspected in a study subject during treatment with the IMP, the IMP must be immediately withheld until the result of a confirmatory test is available. If confirmed, the subject must be withdrawn from the study. Furthermore, the reason and the contraception methods may need to be reviewed and documented as a preventative measure for other participants.

Reliable methods of contraception are

- combined (estrogen and progestogen containing) hormonal contraception associated with inhibition of ovulation,
- progestogen-only hormonal contraception associated with inhibition of ovulation,
- intrauterine device (IUD),
- intrauterine hormone-releasing system (IUS),
- bilateral tubal occlusion,
- vasectomised partner,
- sexual abstinence - only when abstinence is the usual and preferred lifestyle of the subject.

Although not an AE per se, pregnancy in a female study subject or the partner of a male study subject must be recorded if it occurs during the period of observation of the study (see definition in section 9.3.3). The investigator must contact the sponsor immediately in such a situation.

The pregnancy must be documented on a “Drug Exposure Via Parent" (DEVP) Report Form and reported to the sponsor **within 24 hours** of the investigator becoming aware of the pregnancy. If an AE occurs in relation to the pregnancy, it has to be noted on the DEVP form and recorded in the eCRF. If an IRAE/SAE occurs in relation to the pregnancy, it has to be noted on the DEVP form and recorded in the eCRF and follow the immediate reporting process for IRAE including SAE as described in Appendix 2.

The investigator must make all reasonable efforts to follow up the pregnancy until its end and will report all outcomes associated with the pregnancy to the sponsor. In the situation of pregnancy of the female partner of a male study subject, consent for the release of medical data should be obtained from the female partner to allow collection of information on the outcome of the pregnancy.

9.4 Data Safety Monitoring Board

A DSMB will independently monitor the study.

The DSMB will independently review and assess the unblinded safety data throughout the entire study at regular intervals. The DSMB consists of three voting members: an expert in pharmacovigilance, an expert in the field of hematology/hemostaseology and an expert in anaesthesiology. Two members of the DSMB constitute a quorum.

In addition, a statistician without a vote will be responsible for adequate data supply. Prior to the data safety monitoring phase, a meeting will be held to familiarize the DSMB with all relevant procedures. The DSMB members are unblinded during both evaluation periods and will be provided with the following information: reports of SAEs and AEs, data on markers of coagulation and coagulation factors, clinical laboratory assessments of hematology, clinical chemistry and urinalysis, and vital signs.

DSMB meetings will take place at regular intervals. The DSMB will be provided with data covering the screening visit, the day of surgery plus 4 additional follow-up visits (Day 2, 3, 5 and 8) and will evaluate the subjects’ risks at a formal DSMB meeting with regards to the relevant parameters and outcome criteria. In addition, subject's data from the closing visit will also be evaluated if data already available at the time of the DSMB meeting. After each meeting treatment of the following subjects can continue unless the DSMB has not actively disapproved it.

Minutes of the DSMB will describe the proceedings from all sessions of the DSMB meeting, and will summarize all recommendations, which will also be reported to Biotest and the principal investigator.

The DSMB members can propose to stop the study at any time after a scheduled or unscheduled meeting in case of major safety concerns related to study treatment.

Further details will be provided in the DSMB Charter.

10 STATISTICS

The statistical planning and evaluation of the clinical study will be carried out by a qualified biostatistician in accordance with the ICH-guidelines and adequate biostatistical SOPs in SAS version 9.4 or later. A detailed Statistical Analysis Plan (SAP), providing details about the statistical methods for the analyses, will be finalized before unblinding. This ensures that the integrity of the analyses is maintained.

Any deviations from the planned analyses will be described and justified in the Clinical Study Report (CSR).

10.1 Analysis Sets

The following analysis sets will be defined:

All Subjects Enrolled Set:
The All Subjects Enrolled Set includes all subjects who have given informed consent to this study.

Safety Analysis Set (SAF):
The SAF comprises all subjects who have received at least one dose of IMP. Subjects will be analyzed according to the treatment received.

Full Analysis Set (FAS):
The FAS comprises all subjects who received at least one dose of IMP prior to the ‘end of surgery’ and have at least one post dose efficacy assessment.

Subjects will be analyzed as randomized.

Per-protocol Set (PPS):
The PPS includes all subjects who are compliant with the study protocol without any major protocol deviations thought to have the potential to impact the results of the efficacy analysis, e.g. no treatment with IMP, treatment with IMP after the ‘end of surgery’, no post dose efficacy assessment. Classification of protocol deviations as major or minor will be agreed upon at the Blind Data Review Meeting (BDRM) prior to database lock.

Subjects will be analyzed according to the treatment received.

10.2 Protocol Deviations

Deviations from the protocol will be documented on an on-going basis during conduct of the clinical study based on monitoring reports (e.g. failure of eligibility criteria), data management checks and statistical programming (e.g. prohibited medications based on drug codes). Protocol deviations will be discussed and agreed in the BDRM to find protocol deviations with major impact on subject safety or the validity of the study data. Subjects with major protocol deviations will be excluded from the PPS under the assumption that the deviation may have an impact on the efficacy analysis.

The investigator should not implement any deviation from, or changes of the protocol without agreement by the sponsor and prior review and documented approval/ favorable opinion from the IRB/IEC of an amendment, except where necessary to eliminate an immediate hazard(s) to study subjects. The investigator, or person designated by the investigator, should document and explain any deviation from the approved protocol.

10.3 General Considerations

BT524 will be compared with **standard treatment (FFP in the EU and Switzerland / cryoprecipitate in the UK)** whereas FFP and cryoprecipitate will be considered as one treatment group.

The global significance level will be 2.5% (one-sided), confidence intervals will be 95% (two-sided). All statistical tests will be two-sided, unless otherwise stated.

Quantitative (continuous) data - absolute values and differences from baseline, where appropriate - will be summarized with number of observations (n), arithmetic mean, standard deviation, median, minimum, and maximum.

Qualitative (categorical) data will be summarized using number of observations (n), frequency and percentages of subjects. Unless stated otherwise the calculation of percentages will be based on the total number of subjects in the population of interest. Thus counts of missing observations will be included in the denominator and presented as a separate category.

Definition of Baseline
If not stated otherwise, the last non-missing valid observation prior to surgery will serve as the baseline measurement.

Missing Data Conventions
In this short study, not many missing values are expected. Therefore, data will not be imputed for safety analyses or continuous efficacy endpoints.

For binary endpoints, an observed case analysis (excluding missing data) will be considered to be the primary analysis method and a non-responder analysis (treating missing values as non-responders or the worst case) may be performed as a sensitivity analysis if deemed necessary. Details will be given in the SAP.

Pooling of Centers
In case of low number of subjects per center, summaries of data by center would be unlikely to be informative. Therefore, data from all centers per country/region (if applicable) and in total will be pooled prior to analysis.

Subgroups
A subgroup analysis according to the predictive blood loss is planned at least for the primary efficacy variable blood loss.

Disposition
The number of subjects screened and who failed screening prior to surgery or during surgery will be summarized. The number of subjects randomized; number and percentage of subjects treated with IMP; and number and percentage of subjects who prematurely withdrew from the study with the reason for withdrawal will be summarized by treatment arm. The number and percentage of subjects in each of the analysis sets will also be summarized by treatment arm.

Demographic and Baseline Data
Demographic and baseline data will be summarized descriptively.

10.4 Efficacy Analyses

Analyses of the efficacy parameters will be based on the PPS and FAS as appropriate and defined in the SAP.

10.5 Primary Efficacy Analysis

The primary endpoint / efficacy variable is the intra-operative blood loss after decision to treat the subject with IMP until the end of surgery as measured and calculated by amount of blood from the blood suction unit and amount of blood from surgical cloths and compresses.

The primary analysis of this endpoint will test for non-inferiority in the PPS.

The null hypothesis for the primary analysis is that the degree of inferiority of BT524 compared to standard treatment (FFP/cryoprecipitate) is greater than or equal to the non-inferiority margin. The alternative hypothesis is that the degree of inferiority of BT524 compared to FFP/cryoprecipitate is less than the non-inferiority margin.

$H_0: \mu_1 - \mu_2 \geq \delta$

$H_1: \mu_1 - \mu_2 < \delta$

where

μ_1 = mean intra-operative blood loss after the decision to treat the subject with IMP in the BT524 treatment arm

μ_2 = mean intra-operative blood loss after the decision to treat the subject with IMP in the FFP-/cryoprecipitate-treatment arm

δ = non-inferiority margin = 150 mL

The final analysis will be performed using ANCOVA with the intra-operative blood loss after the decision to treat the subject with IMP until the end of surgery as the dependent variable and the predictive blood loss (>1,000 mL to ≤ 2,000 mL and > 2,000 mL) as a covariate. The least square means and treatment difference (BT524 versus standard treatment) in least square means will be presented with the corresponding 95% confidence intervals and 2-sided p-value. Non-inferiority will be demonstrated if the upper confidence limit of the 2-sided 95% confidence interval for the difference in the least square means is less than the non-inferiority margin (150 mL).

This analysis will also be performed in the FAS as a sensitivity analysis.

If non-inferiority is demonstrated, then superiority will be assessed in the FAS with superiority demonstrated if the 2-sided p-value is less than 0.05 (i.e., the upper confidence interval is less than 0 mL) using the analysis performed to assess Non-Inferiority.

No imputation for missing values will be applied.

10.6 Secondary Efficacy Analyses

All secondary efficacy analyses will be conducted with the FAS. The secondary endpoints / efficacy variables are:

- Proportion (%) of subjects with successful correction of fibrinogen level 15 minutes after start of first IMP administration. Successful correction of fibrinogen level in a subject is defined as restoring fibrinogen FIBTEM A10 baseline levels measured by ROTEM 15 minutes after start of first IMP administration. A correction of at least 95% is considered successful.
- Time to first successful correction of fibrinogen level (15 minutes or 90 minutes after start of first IMP administration, end of surgery, not within surgery).

- Total amount of transfusion products (allogenic blood products) or autologous blood transfusion infused after start of first IMP administration until end of surgery.
- Amount of RBCs (allogenic and autologous) infused after start of first IMP administration until end of surgery.
- Post-operative blood loss in the first 24 hours.
- Proportion (%) of subjects with rebleeds after the end of the surgery until Day 8.
- Hospital length of stay after surgery.
- In-hospital mortality.

10.6.1 Correction of Fibrinogen Level

The proportion of subjects with a successful correction of fibrinogen level will be compared between the treatment arms using a CMH approach stratified by predictive blood loss (> 1,000 mL to ≤ 2,000 mL and > 2,000 mL). The number and percentage of subjects with a successful correction of fibrinogen level will be presented with corresponding 95% confidence intervals. The estimated treatment effect (i.e., the difference in correction rate between the treatment arms), corresponding 95% confidence interval, and 2-sided p-value for the difference will be presented.

The time to first successful correction of fibrinogen level will be compared between the treatment arms using a Chi-Square test. The number and percentage of subjects reaching a successful correction at each time point (15 minutes or 90 minutes after start of first IMP administration, end of surgery, not within surgery) will be presented together with the p-value for differences between treatment arms.

Absolute values and change from baseline in fibrinogen levels will be presented descriptively over time by treatment arm.

10.6.2 Consumption of Transfusion Products

The total amount of transfusion products (allogenic blood products or autologous blood transfusion or cell salvage) infused until end of surgery will be descriptively summarized by type of transfusion product and treatment arm.

10.6.3 Amount of Red Blood Cells

The amount of RBCs required intra-operatively will be descriptively summarized by treatment arm.

An ANCOVA analysis will be performed with the amount of RBCs required as the dependent variable and the predictive blood loss (> 1,000 mL to ≤ 2,000 mL and > 2,000 mL) as a covariate. The least square means and difference in least square means will be presented with the corresponding 95% confidence intervals and 2-sided p-value.

10.6.4 Post-operative Blood Loss

The post-operative blood loss in the first 24 hours will be descriptively summarized by treatment arm.

An ANCOVA analysis will be performed with the post-operative blood loss in the first 24 hours as the dependent variable and the predictive blood loss (> 1,000 mL to ≤ 2,000 mL and > 2,000 mL) as a covariate. The least square means and difference in least square

means will be presented with the corresponding 95% confidence intervals and 2-sided p-value.

10.6.5 Proportion of Subjects with Rebleeds

The proportion of subjects with rebleeds after the end of surgery (until Day 8) will be compared between the treatment arms using a CMH approach stratified by predictive blood loss (> 1,000 mL to ≤ 2,000 mL and > 2,000 mL). The number and percentage of patients with a rebleed will be presented with corresponding 95% confidence intervals. The estimated treatment effect (i.e., the difference in rebleed rate between the treatment arms), corresponding 95% confidence interval, and 2-sided p-value for the difference will be presented.

10.6.6 Hospital Length of Stay after Surgery

The hospital length of stay after surgery will be descriptively summarized by treatment arm.

10.6.7 In-hospital Mortality

The number and percentage of subjects died during hospital stay will be presented by treatment arm.

10.7 Safety Analysis

AEs will be coded using the most current version of Medical Dictionary for Regulatory Activities (MedDRA®). The version used will be defined in the SAP. Incidence rates (i.e., number and percentage of affected subjects) will be calculated for the coding levels *SOC* and *preferred term* and will be presented by treatment arm. Further analyses of AEs will focus on seriousness, intensity, causal relationship to IMP, and outcome. IRAE including SAEs etc. will be displayed in detail.

Safety laboratory assessments (hematology, clinical chemistry, urinalysis and coagulation parameters) will be categorized with respect to the laboratory specific reference ranges as normal/abnormal. Abnormal values will be further classified with respect to clinical relevance. Changes over time will be described by means of "shift-tables" by treatment arm as well as summarized with descriptive statistics by time point and treatment arm.

Vital signs data will be summarized descriptively before and after surgery by treatment arm.

The frequency of thrombosis and of TEEs, virus status, exposure data and concomitant medication will be summarized by treatment arm.

All safety analyses will be based on the SAF.

10.8 Interim Analyses

In this study, 3 interim analyses of the observed blood losses are planned to have the option of adjusting the sample size needed (primary: PPS, secondary: FAS).

For this purpose, an alpha-adjustment according to Haybittle/Peto (Haybittle, 1971; Peto et al., 1976; Schulz and Grimes, 2005) is planned. This leads to local alpha levels of 0.001 for each interim analysis and a significance level of 0.05 for the final analysis to reach a global significance level of 5%.

All interim analyses will be based on the PPS.

The first interim analysis is planned with approximately 50 spine subjects, the second one with at least 40 PMP subjects and all other evaluable spine subjects at that time-point. The third interim analysis is planned after approximately 80% of subjects of the total sample size.

Aim of all interim analyses is to adapt the sample size according to the observed blood losses and the standard deviations:

a.) Early termination due to non-inferiority of BT524 in comparison with the used standard therapies.
b.) Continuation with the sample size as initially planned.
c.) Adjustment of sample size to take into account changes from the previous assumptions on the additional blood loss.
d.) Stopping the study early due to futility if the sample size re-estimation indicates a much higher number than planned before.

10.9 Determination of Sample Size

The non-inferiority margin is defined as 150 mL blood loss, as such difference in blood loss after the decision to treat the subjects with IMP is considered as clinically not relevant.

The intra-operative trigger for treatment, “a clinically relevant bleeding or the prediction of a clinically relevant bleeding, requiring hemostatic treatment during surgery”, is defined as the time of decision to treat the subjects with IMP. Based on the assumption of an additional blood loss of about 500 mL in the FFP-/cryoprecipitate-treatment arm after the decision to treat, a further intra-operative blood loss of approximately 150 mL would not lead to a further transfusion of ≥ 1 unit of packed RBCs, ≥ 1 unit of FFP/cryoprecipitate, or ≥ 1 unit of whole blood.

Whole blood contains RBCs and plasma components of circulating blood. A single whole blood donation contains approximately 500 mL of blood with a minimum hematocrit of 38%. When the plasma is removed, RBCs remain and have a hematocrit of > 80% and a volume of 225-350 mL. Additive solutions mixed with the red cells result in a hematocrit of 55-65% and a volume of 300-400 mL. One unit of whole blood or one unit of RBCs can be expected to result in a hemoglobin increase of 1 g/dL or a hematocrit increase of 3% in a typical adult. Therefore, one unit of RBCs can replace a blood loss of 500 mL (Avery and Avery, Spring 2010; Liumbruno et al., 2009). Accordingly, a volume of 150 mL (after an assumed blood loss of 500 mL) is still below a clinically relevant blood loss and would not trigger an additional administration of transfusion products.

It is assumed that BT524 is non-inferior that means not worse than FFP/cryoprecipitate with a non-inferiority margin of 150 mL in reducing intra-operative blood loss. Assuming a blood loss of about 500 mL in the FFP-/cryoprecipitate-treatment arm after the decision to treat the subject with IMP until end of surgery, a standard deviation of 375 mL, a non-inferiority margin of 150 mL, an alpha-level of 2.5% (1-sided) 100 evaluable subjects per treatment arm are needed to demonstrate the non-Inferiority of BT524 by using a t-test (equivalence) with 80% power.

The sample size will be recalculated at the interim analyses as defined in section 10.8. Sample size estimations will be performed by using nQuery Advisor Version 4.0 or higher.

With 100 subjects per treatment arm superiority of BT524 can also be tested with a power of >80% (t-test, alpha=0.05 2-sided, effect size Δ=0.5).

10.9.1 Data Monitoring

After 40 subjects have completed the study, the overall mean and standard deviation for the primary efficacy variable intra-surgery blood loss after decision to treat the subject with IMP will be derived using blinded aggregate data of all 40 subjects without separating according to treatment.

If the assumed mean and standard deviation blood loss are not reflected in these subjects, then a sample size adjustment will be considered to ensure that a sufficient number of subjects has been randomized to maintain a power of 90%.

If an adaption of the sample size is intended, this will be documented in a protocol amendment.

This data monitoring is not an interim analysis because the analysis is performed with all subjects without separating the subjects according to treatment. Therefore, no alpha-adjustment is necessary.

11 DATA MANAGEMENT

11.1 Data Collection

Electronic Case report form (eCRF)

The eCRF is the primary data collection instrument for the clinical study. All data to be recorded according to this CSP must be documented. Entries in the eCRF must only be made by the investigator or persons authorized by the investigator. A list of all persons who are allowed to make entries in the eCRF must be available in each study site.

eCRF completion guidelines will be provided as electronic version with the eCRF as a link on the dashboard.

Clinical study data will be directly entered via eCRF into the study database on a central server by authorized investigator and/or study personnel.

It is ensured that the *electronic data capture (*EDC) system (including the eCRF) is built up with following requirements: validated system, functionality of different user roles and access administration, password protection, given traceability, record keeping, and availability of audit trail functionality as well appropriate standard operation procedures are maintained.

The investigator and/or assigned study personnel at each site will enter data from source documents corresponding to a subject’s visit into the protocol-specific eCRF. Subjects will be identified by a unique study specific number and/or code in any database. The subject's name and any other identifying detail will not be included in any study data-electronic file.

Laboratory samples (e.g. safety lab, clinical immunology, pharmacokinetic, other study specific laboratory data) will be collected and analyzed in local laboratories at each site or shipped to central laboratory for analysis (section 9.2.1.4). The results of local laboratory samples will be sent back to the investigator to be entered into the eCRF. The central laboratory data will be available for evaluation by the investigator via the web-

based tool provided by the vendor. Data will be sent to the CRO on a regular basis to join with the clinical data entered via eCRF.

The complete data management activities (data entry, data validation, query handling, data editing after entry, coding, data base closure, etc.) will be defined in advance within a data management plan together with a description of the personnel responsible for data correction, performance, and controlling as well as specific data handling procedures.

MedDRA® dictionary will be used for coding of AE, concomitant diseases and medical history. Concomitant medication will be coded using the Word Health Drug Dictionary (WHO-DD). Details are provided in the data management plan and the study specific safety manual.

11.2 Correction of Data

After data have been entered into the clinical study database, a system of computerized data validation checks will be implemented and applied to the database on a regular basis.

Definition and details are provided in the data validation specifications.

Queries are entered, tracked, and resolved through the eCRF system directly. If a correction is required for an eCRF, the time and date stamps tracking function creates an electronic audit trail for the person entering/updating the eCRF data.

11.3 Data Handling

The data will be entered into a validated database. The Data Management personnel will be responsible for data processing, in accordance with procedural documentation. Database lock will occur once all data entered are clean and quality assurance procedures have been completed.

All procedures for the handling and analysis of data will be conducted according to available ICH-GCP guidelines for the handling and analysis of data for clinical studies.

12 QUALITY CONTROL AND QUALITY ASSURANCE

12.1 Study Initiation Activities

The investigator(s) is/are informed about objectives and methods of the study, the inclusion and exclusion criteria, the time-schedule, and the applied procedures by means of a Pre-Study Visit by the monitor (if necessary), an investigators' meeting prior to start of the study, and during the Site Initiation Visit by the monitor.

12.2 Training of site staff

The Principal Investigator needs to ensure that all persons assisting with the clinical study are adequately informed about the protocol, the investigational product(s) and their study related duties and functions. Furthermore the Principal Investigator is requested to maintain a list of appropriately qualified persons to whom the investigator has delegated significant study-related duties.

12.3 Documentation and Filing

List of Subjects (subject identification log)

The investigator is asked to keep a confidential list of names of all subjects participating in the study, giving reference to the subjects' records.

With the help of this list it must be possible to identify the subjects and their medical records. In addition, the investigator is asked to keep a list of all subjects screened on a screening log to document identification of subjects who entered pre-study screening. In case of non-eligibility a reason is to be provided.

Source Data

Source data is all information in original records and certified copies of original records of clinical findings, observations, or other activities in a clinical study necessary for the reconstruction and evaluation of the study. Source data are contained in source documents which comprise clinical documentation, data, and records (e.g. hospital records, clinical and office charts, laboratory notes, memoranda, subjects' diaries or evaluation checklists, pharmacy dispensing records, recorded data from automated instruments, copies or transcriptions certified after verification as being accurate copies, microfiches, photographic negatives, microfilm or magnetic media, x-rays, subject files, and records kept at the pharmacy or blood bank, at the laboratories and at medico-technical departments involved in the clinical study). Any data recorded directly in the eCRFs (i.e., no prior written or electronic record of data) will also be considered to be source data.

Investigator Site File / Regulatory Binder

Before site initiation the CRO will provide an Investigator Site File / Regulatory Binder to each study site. The Investigator Site File will include essential documents as defined by the ICH GCP guideline and applicable local requirements.

The investigator will be responsible for the continual update and maintenance of the investigator site file, which will be periodically reviewed by the monitor(s). In case of an audit by the sponsor or an inspection by the Regulatory Authorities these documents will be reviewed.

All study related documents are to be archived and stored according to legal requirements, but at **least for 25 years** after completion of the study.

Prior to destruction the investigator will contact Biotest AG for approval and conformation of such.

12.4 Monitoring

The monitor is responsible for checking the quality of data and adherence to the study protocol and to legal and ethical requirements according to local laws and GCP.

The interval between monitoring visits will be dependent on the recruitment rate and the complexity of the study.

Source data verification is an essential part of the monitoring process and the investigator must grant direct access to the subjects' source data.

The extent and nature of monitoring will be described in detail within the monitoring plan.

12.5 Audits and Inspections

Audits will be performed according to the corresponding audit program, including the possibility that a member of the sponsor's quality assurance function may arrange to visit the investigator in order to audit the performance of the study at the study site, as well as all study documents originating there. Audits may also be performed by contract auditors. In this case, the sponsor's quality assurance function will agree with the contract auditor regarding the timing and extent of the audit(s). In case of audits at the investigational site, the monitor, PM-CRO (Project Manager CRO) or CCR (Clinical Manager (cM) Biotest) will usually accompany the auditor(s).

Inspections by regulatory authority representatives and IECs/IRBs are possible at any time, even after the end of study. The investigator has to notify the sponsor immediately of any such inspection. The investigator and institution will permit and support study-related monitoring, audits, reviews by the IEC/IRB and/or Regulatory Authorities, and will allow direct access to source data and source documents for monitoring, audits, and inspections. The principal investigator shall personally participate in all audits and inspections.

12.6 Archiving

After evaluation and reporting of the study data, all documents relating to the clinical study will be kept in the archives of the sponsor or of a contracted service provider and the study site(s) according to applicable regulatory requirements.

13 GENERAL REGULATIONS, AGREEMENTS AND ORGANISATIONAL PROCEDURES

13.1 Study Administrative Structure

Details for the study administrative structure are kept as a separate list filed in the Trial Master File.

13.2 Ethical and Regulatory Considerations

This CSP and any amendments will be submitted to a properly constituted Independent Ethics Committee (IEC) / Institutional Review Board (IRB) and/or Regulatory Authorities (RA), in agreement with applicable regulatory requirements, for formal approval of the study conduct. A copy of these approvals must be submitted to Biotest before initiation of the clinical study and each site needs to keep a copy of these documents.

Changes to the CSP must be made in the form of an amendment that has the prior written approval of Biotest. Substantial CSP amendments need to be notified to/approved by IEC/IRB and/or Competent Authorities (CA) / Regulatory Authorities (RA) prior to implementation as required by applicable regulations.

The clinical study will be performed according to the applicable regulatory requirements taking into account the principles of GCP and the latest version of the Declaration of Helsinki.

13.3 Committees / Monitoring Boards

Safety data from the clinical study will be evaluated by a DSMB at regular intervals during the study, to ensure that the continuation of the study is appropriate and to make recommendations to the sponsor. The DSMB will consist of permanent members who are not associated with the sponsor or with the operative conduct of the study. A description of the scope of work and operating procedures for the DSMB is provided in the DSMB Charter. The composition of the DSMB will also be outlined in the DSMB Charter.

13.4 Written Agreements

A written agreement will be set up between Biotest and each investigator setting out any arrangements on delegation and distribution of tasks and obligations and, if appropriate, on financial matters.

13.5 Insurance/Liability

In accordance with the relevant national regulations, the sponsor has taken out a subject liability insurance for all subjects who have given their consent to the clinical study. The subjects are insured against injury caused by study medication or participation. The subjects will be informed about the insurance and their own responsibilities and duties.

The insurance company issuing the policy is defined by the Insurance Certificate for Clinical Trials for the respective county. This certificate will comply with the country-specific legal requirements.

13.6 Investigator’s Brochure (IB)

The investigator will be informed about current knowledge concerning the study medication BT524 through an Investigator´s Brochure (IB). All investigators will be informed immediately about relevant new information available.

13.7 Amendments to the Protocol

Changes to the CSP must be made in the form of a CSP Amendment that has the prior written approval of Biotest. Substantial changes to the protocol need to be notified to/approved by IEC/IRB and/or Regulatory Authorities prior to implementation, as required by applicable regulations.

Amendments in order to eliminate immediate hazard to subjects may be implemented before the approval of the IEC/IRB and/or Regulatory Authorities after consultation with Biotest.

In the event that a significant deviation from the protocol is anticipated based on the subjects status, or occurs due to an accident or mistake, the investigator or his/her designee must contact Biotest or PPD (CRO) at the earliest possible time. This will allow an early joint decision to be made as to whether or not the subject should continue in the study. This decision will be documented by both the investigator and Biotest or PPD (CRO).

13.8 Confidentiality

The objectives and contents of this clinical study as well as its results are to be treated as confidential and may not be made accessible to third parties.

Information about study subjects will be kept confidential and managed according to the requirements of the Health Insurance Portability and Accountability Act of 1996. Those regulations require a signed subject authorization informing the subject of the following:

- What protected health information (PHI) will be collected from subjects in this study
- Who will have access to that information and why
- Who will use or disclose that information
- The rights of a research subject to revoke their authorization for use of their PHI.

In the event that a subject revokes authorization to collect or use PHI, the investigator, by regulation, retains the ability to use all information collected prior to the revocation of subject authorization. For subjects that have revoked authorization to collect or use PHI, attempts should be made to obtain permission to collect at least vital status (i.e. that the subject is alive) at the end of their scheduled study period.

13.9 Final Report and Publication

For each study an integrated final report according to ICH-requirements will be produced. At the end of the study the sponsor will provide the competent authority and IEC/IRB with a summary of the CSR **within < 1 year** after the end of the study, where required.

It is generally recommended that the results of clinical studies be presented at congresses and symposia and/or published in scientific journals. Prior to their publication, all results of medical tests with the sponsor's products, and/or publications or lecture manuscripts concerning such results, are to be reviewed and discussed by the coordinating investigator and the sponsor by mutual agreement.

Each investigator is obligated to keep data pertaining to the study secret. He/she must consult with the sponsor before any study data are published.

The legitimate interests of the sponsor, such as acquiring optimum patent protection, coordinating submissions to the health authorities or coordination with other studies in the same field that are underway, protection of confidential data and information, etc. will be given due consideration by all partners involved.

14 LIST OF REFERENCES

PPD [redacted]

[redacted]

[redacted]

[redacted]

[redacted]

[redacted]

[redacted]

[redacted]

[redacted]

[redacted]

[redacted]

[redacted]

PPD [redacted]

PPD [redacted]

[redacted]

[redacted]

[redacted]

[redacted]

[redacted]

[redacted]

[redacted]

[redacted]

[redacted]

[redacted]

[redacted]

[redacted]

PPD [redacted]

PPD [redacted]

[redacted]

[redacted]

[redacted]

[redacted]

[redacted]

[redacted]

[redacted]

[redacted]

[redacted]

[redacted]

[redacted]

PPD [redacted]

PPD [redacted]

[redacted]

[redacted]

[redacted]

[redacted]

[redacted]

[redacted]

[redacted]

[redacted]

[redacted]

[redacted]

[redacted]

PPD [redacted]

PPD [redacted]

[redacted]

[redacted]

[redacted]

[redacted]

[redacted]

[redacted]

[redacted]

[redacted]

[redacted]

PPD [redacted]

[redacted]

[redacted]

[redacted]

[redacted]

[redacted]

[redacted]

[redacted]

[redacted]

[redacted]

[redacted]

[redacted]

[redacted]

PPD [redacted]

Diagnose - Indikation und Bewertung von Laborbefunden für die medizinische PPD [redacted]

[redacted]

[redacted]

15 APPENDICES

Appendix 1: Safety Definitions

Appendix 2: Reporting Procedures

Appendix 1: Safety Definitions

- **Adverse Event (AE)**

  Any untoward medical occurrence in a patient or clinical study subject administered an IMP and which does not necessarily have a causal relationship with this treatment. An AE may be any aggravation or new unfavorable and unintended sign, symptom, or disease temporally associated with the use of an IMP, whether or not considered related to the IMP.

  This includes abnormal laboratory and other investigation results which are considered clinically relevant by the investigator (unless already pre-existing at baseline). However, if an abnormal laboratory value is a sign of an already reported AE (e.g. infection), the respective abnormal laboratory value does not constitute a separate AE.

  A surgical or invasive procedure is not an AE in itself. Instead, the condition for which the surgical or invasive procedure is performed may be an AE. Planned or elective surgery or procedures (i.e. planned prior to signature of informed consent) for a pre-existing condition and the pre-existing condition leading to surgery or procedure are not AEs. However, if the pre-existing condition worsened after signature of informed consent, the worsening of the condition constitutes an AE.

  Worsening of the disease under study (underlying disease): This will be captured by efficacy parameters and should not usually be recorded as AE, unless one or more of the following criteria are met:

  - The worsening of the disease under study constitutes a serious AE
  - A deterioration exceeding the usual fluctuations of the disease under study has occurred in the opinion of the investigator
  - The worsening leads to discontinuation of the study medication
  - Additional treatment is required for the worsening, e.g. concomitant medication is added or changed.

  No causal relationship with the investigational drug, or comparator drug, or study procedures is implied by the use of the term “Adverse Event”.

- **Adverse reaction of an investigational medicinal product:**

  All untoward and unintended responses to an IMP related to any dose administered. All AEs judged by either the reporting investigator or the sponsor as having a reasonable causal relationship to a medicinal product qualify as adverse reactions. The expression reasonable causal relationship means to convey in general that there are facts, evidence or arguments to suggest a causal association with the drug.

- **Serious Adverse Event (SAE)**

  An SAE is any untoward medical occurrence or effect that at any dose*:

  - results in death
    - *Death is an outcome of an AE and not an AE in itself. All deaths, regardless of cause or relationship must be reported for study subjects.*
  - is life-threatening

        - *"Life-threatening" refers to an event in which the subject was at risk of death at the time of the event; it does not refer to an event which hypothetically might have caused death if it were more severe.*
    - requires hospitalization or prolongation of existing hospitalization
        - *In-subject hospitalization means that the subject has been formally admitted to a hospital for medical reasons, for any length of time, which may or may not be overnight. It does not include presentation and care within an emergency department.*
        - *A complication that occurs during hospitalization and prolongs the existing hospitalization is an SAE. Complications that occur during hospitalization but do not prolong the existing hospitalization and do not meet any other seriousness criteria are non-serious AEs.*
        - *Elective or pre-planned (prior to signature of informed consent) hospitalization for investigations, medical or surgical treatment does not meet this seriousness criterion. However, if the underlying condition for which hospital treatment or surgery had been planned worsened during the study, the worsening of the condition is to be reported as SAE.*
    - results in persistent or significant disability / incapacity
    - is a congenital anomaly / birth defect
    - is another important medical event
        - *Adverse events that may not be immediately life-threatening or result in death or hospitalization but my jeopardize the subject or may require medical or surgical intervention to prevent one of the other outcomes listed above, should be reported as serious. Medical and scientific judgment must be exercised in deciding whether an event is serious.*

* "At any dose" does not necessarily imply that the subject is receiving the study drug at the time of the event.

- **Diagnosis vs. Signs/Symptoms**

The investigator should provide a diagnosis rather than individual signs and symptoms, wherever possible and appropriate. However, if there is not enough information to provide a diagnosis, individual signs and symptoms are to be recorded. If a diagnosis is accompanied by unusual symptoms, the diagnosis itself and the unusual symptoms have to be reported separately. For serious and other IRAEs the investigator shall provide any other supporting information that may be required for the assessment of the events, specifically in the free text narrative description of the case. This is of particular importance in situations where a diagnosis cannot (yet) be made. Any subject identifying data on supporting documents (e.g. name, address, phone number) have to be obliterated prior to sending them to the sponsor.

A complication of an AE constitutes another AE. For example in diarrhea leading to dehydration, diarrhea and dehydration would be captured as separate AEs.

The eCRF provides for a number of items to be completed for each AE. This includes the onset date, end date, intensity/severity, seriousness, action taken with study medication, treatment for the AE, outcome, and causal relationship of the AE with the study medication, other drugs, or study procedures.

- **Onset date, end date**

If an AE started during the study but did not end before the final follow-up visit, the investigator must make a reasonable effort to establish the outcome and the end date. If this is not possible, e.g. because the AE is still ongoing, or the subject is lost to follow-up, there will be no end date for the AE.

For all AEs that resolve, resolve with sequelae, or have a fatal outcome, an end date must be provided.

If an AE stops and restarts later, all occurrences have to be recorded separately.

If an AE starts as non-serious AE and becomes serious at a later point in time, the following applies in regard to onset and end dates:

- **Intensity/Severity**

Refers to the extent to which an AE affects the subject's daily activities. Severity will be categorized according to the following criteria:

Table: Adverse Event Severity

**Mild**	The AE does not interfere with the subject's routine activities.
**Moderate**	The AE interferes with the subject's daily routine, but usual routine activities can still be carried out.
**Severe**	The AE results in inability to perform routine activities.

Severe vs. Serious: The severity is used to describe the intensity of an event. This is not the same as seriousness, which is based on subject/event outcome or action criteria usually associated with events that pose a threat to subject's life or functioning. Seriousness, not severity, serves as the guide for defining regulatory reporting obligations.

Example: While an event may be of "severe" intensity, it may be of relatively minor medical significance, such as severe headache. On the other hand, a myocardial infarction would be usually regarded as serious, even if its intensity is "mild".

If formal severity classifications are used, such as the NCI CTC classification used in oncology studies, provisions should be made regarding the reporting of events of defined grading (e.g. ≥ 3) as serious.

AEs with changes in severity: If an AE changes in severity, this will be captured as one AE with the highest severity grade recorded.

- **Seriousness**

  For definition of seriousness criteria refer to section 9.3.1. For a serious AE all seriousness criteria that apply have to be reported. Reporting requirements by the investigator are detailed in Appendix 2.

  If a list of always serious terms is to be used in the study, this should be mentioned here, as well as its use by the investigator. For example a PT-based auto-seriousness in the eCRF, or an urgent query process once a verbatim is coded to a PT on the list.

- **Action Taken with Study Medication**

  The action taken with study medication as a result of the AE has to be documented. In the situation that the AE leads to permanent discontinuation of the study medication, this meets the definition of an AE leading to subject's withdrawal from the study, which is an immediately reportable AE (IRAE). Reporting requirements by the investigator are detailed in section Appendix 2.

- **Treatment for the AE**

  It has to be specified in the eCRF if counteractive treatment was given for the AE. Any treatment for an AE, whether pharmacological or other (e.g. surgical) treatment, has to be recorded in the eCRF.

- **Outcome**

  The following categories are used:

  - Resolved
    - Indicates that the event has fully resolved.
  - Resolving
    - Indicates that the event is in the process of recovery but has not yet fully resolved.
  - Not resolved
    - Indicates that the event is ongoing and there has been no recovery.
  - Resolved with sequelae
    - Indicates that there is a residual, possibly permanent consequence of the event (e.g. residual hemiparesis subsequent to stroke).
  - Fatal
    - Indicates that the subject died due to the event. The outcome "fatal" applies only to the event(s) that were the cause(s) of death. For other AEs that were ongoing at the time of death, the outcome must not be "fatal" but "not resolved".

- **Causal Relationship of AE**

  The causal relationship with the study medication has to be reported for each AE. It refers to the presence or absence of a reasonable possibility of a causal relationship between the study medication and the AE. The investigator is asked to use medical judgment and take into account the nature of the AE, subject's

medical history, temporal relation, response to withdrawal or interruption of study drug (dechallenge), response to re-introduction of study drug (rechallenge), any alternative explanations such as underlying or concomitant diseases, concomitant drugs, study procedures.

The following categories are used:

- Related: There is a reasonable possibility of a causal relationship between the study medication and the AE.
- Not related: There is no reasonable possibility of a causal relationship between the study medication and the AE.

For serious and other immediately reportable AEs the investigator is asked to specify if there are alternative and/or additional explanations for the occurrence of the event, e.g. concomitant drugs, study procedures, or concomitant/underlying disease and should provide this information already with the initial case report.

- **Concomitant medication**

Concomitant medication will be documented according to categories of medicinal products which are normally used in clinical studies as NIMPs (European Commission, 18/03/2011):

(1) Rescue medication
(2) Challenge agents
(3) Concomitant medicinal products systematically prescribed to the study patients
(4) Background treatment

Definitions adapted from the Guidance on IMPs and NIMPs (European Commission, 18/03/2011):

- **Rescue medication**

Rescue medications are medicines identified as those that may be administered to the patients when the efficacy of the IMP is not satisfactory, or the effect of the IMP is too great and is likely to cause a hazard to the patient, or to manage an emergency situation. Rescue medication allows patients to receive effective treatment, e.g. where a standard treatment is available.

- **Challenge agents**

Challenge agents are usually given to study subjects to produce a physiological response that is necessary before the pharmacological action of the IMP can be assessed.

- **Concomitant medicinal products systematically prescribed to the study patients**

This type of NIMP is given to clinical study participants as required in the protocol as part of their standard care for a condition which is not the indication for which the IMP is being tested, and is therefore not the object of the study.

- **Background treatment**

This type of medicinal product is administered to each of the clinical study subjects, regardless of randomization arm, to treat the indication which is the object of the study. Background treatment is generally considered to be the current standard care for the particular indication. In these studies, the IMP is given in addition to the background treatment and safety and efficacy are assessed. The protocol may require that the IMP plus the background treatment is compared to an active comparator or to placebo plus background treatment.

Appendix 2: Reporting Procedures

Reporting procedure:

All IRAE information has to be recorded in the IRAE/SAE form and reported to PPD Corporate Drug Safety (CDS) immediately (i. e. within 24 hours) by e-mail or fax (see addresses below in the box) after the investigational site becoming aware of the IRAE.

In addition, this IRAE has to be recorded on the AE page of the eCRF and the following eCRF pages have to be updated or completed at the same time as necessary: Study drug documentation, subject demographics, medical history, concomitant medication, and study completion/termination (in case of an AE leading to withdrawal).

Entry of an IRAE/SAE into the eCRF will trigger an alert message to PPD and PPD CDS.

PPD	[redacted]
[redacted]	[redacted]
[redacted]	[redacted]
[redacted]	[redacted]

For questions regarding IRAEs including SAEs or to notify the sponsor of an IRAE including SAE in the event of technical failure of the e-mail or fax system, the investigator should contact PPD [redacted].

Whenever follow-up information becomes available to a previously recorded IRAE/SAE, this has to be captured in the IRAE/SAE form and should be send by e-mail or fax together with any supporting documents (e.g. medical records, autopsy report, ECG or laboratory reports) as part of the follow-up information accompanied by a cover page within max. 24 hours of the investigator becoming aware of the follow-up information to the reporting contact above.

In addition, the new information should be captured in the eCRF on the AE page within max. 24 hours after becoming aware of the follow-up information.

The investigator has to undertake active follow-up for subjects with IRAE/SAEs. The investigator shall respond to queries raised by the sponsor with regard to IRAE/SAEs within the timelines stipulated in the query, and provide all necessary information as requested. In case of a fatal or life-threatening SAE, the sponsor will contact the investigator urgently to obtain required additional information within one business day. If supporting documents are requested by the sponsor (e.g. copies of medical records, laboratory reports, ECG tracings, autopsy report), the investigator must ensure that subject identifying data are obliterated prior to sending to the sponsor. The supporting documents should carry the subject ID for identification.

If required the investigator is responsible to inform local IECs/IRBs of safety reports in compliance with applicable regulatory requirements. Copies of all correspondence

relating to reporting of safety reports to IEC/IRB should be maintained in the Investigator Site File/ Regulatory Binder.

The sponsor is responsible for fulfilling all obligations regarding notification of regulatory authorities, ethics committees according to applicable regulatory requirements, in regard to expedited reporting (e.g. serious unexpected suspected adverse reactions) and periodic reporting (e.g. development Safety Update Report). In addition, the sponsor is responsible for information of investigators according to the current legislation.

Document S3: Study 995 Statistical analysis plan—(995_Statistical Analysis Plan_V6_19-Jun-2023_redacted)

PPD [redacted]

Statistical Analysis Plan

PPD [redacted]

1.0 Approvals

Sponsor

PPD [redacted]

Strictly Confidential © 2023, ICON, all rights reserved

PPD

Statistical Analysis Plan

**Sponsor:**	Biotest AG
**Protocol No:**	Biotest 995
PPD **Project Id:**	PPD
**Version Date:**	19-JUN-2023
**Version Status/No.:**	Final 6.0

**Title:**	A randomized, active-controlled, multicenter, phase III study investigating efficacy and safety of intra-operative use of BT524 (human fibrinogen concentrate) in subjects undergoing major spinal or abdominal surgery (AdFIrst)
**CRF Version No./Date:**	3.0 / 15-Oct-2020
**Protocol No./Date:**	Final 4.0 / 04-Dec-2019 and Final 4.3 UK / 06-Sep-2021

1.0 Approvals

**Sponsor**	
**Sponsor Name:**	Biotest AG
**Representative/ Title:**	PPD
**Signature /Date:**	
**Representative/ Title:**	PPD
**Signature /Date:**	
PPD	
**Project Manager/Title:**	PPD
**Signature /Date:**	
**Biostatistician / Title (Owner):**	PPD
**Signature /Date:**	

(NOTE: Electronic Signatures should only be used if all parties have the ability to eSign.)

PPD

Change History

Version	Change Log
1.0	Created as new
2.0	Added further rules for imputation of missing date and time<br><br>Updated definition for relative day<br><br>Updated definition for prior and concomitant medication<br><br>Updated analysis for transfusion products and laboratory data
3.0	Updates which are editorial in nature and do not impact the analyses are not listed.<br><br>Updated version of protocol and electronic Case Report Form (eCRF).<br><br>Added new treatment with cryoprecipitate for subjects undergoing cytoreductive pseudomyxoma peritonei (PMP) surgery.<br><br>Added interim analyses.<br><br>Added visit Surgery Day 1 (Prior 1st dose).<br><br>Updated inclusion criteria.<br><br>Updated calculation of sample size.<br><br>Updated randomization dependent on surgery (spine vs. cytoreductive PMP).<br><br>Updated definitions of full analysis set and per-protocol set (PPS).<br><br>Updated protocol deviation categories based on PD Guidance Template v3.0.<br><br>Added TFL specification documents to Appendix 1
4.0	Added new UK protocol version.
5.0	Update to section 6.2 Sample Size Considerations. Clarification of total sample size taking randomized subjects who do not meet the PPS criteria into consideration.<br><br>Update to section 8.12 Correction of Fibrinogen Level. Clarification of “15 minutes after start of IMP administration” added.<br><br>Update to section 9.3 Full Analysis Set. Definition supplement for an unambiguous assignment of subjects to the analysis set.<br><br>Update to section 9.4 Per-Protocol Set. Definition supplement for an unambiguous assignment of subjects to the analysis set.

PPD

	Update to section 12.3.1, adding number of IMP administrations in grams.<br><br>Update to safety section:<br><br>12.6.1 Adverse Events.<br>Clarification and update to the list of AESIs and the respective definitions. Update to the AE table section, including analysis by type of surgery (spinal and abdominal surgery)<br><br>12.6.3 Laboratory Data. Analysis by type of surgery (spinal and abdominal surgery).
6.0	New exploratory efficacy endpoint "Overall Mortality" added to section 5.1 and section 7.2.1<br><br>Update to section 8.6. New subgroups added. These subgroups are described in more detail in section 12.<br><br>Update to the definition of correction of fibrinogen level in section 8.12<br><br>Definition of overall mortality added to section 8.19<br><br>Update to section 9.3 describing the full analysis set to be in line with the ICH E9 guidelines. The previous full analysis set is now described in section 9.3.1.<br><br>Tabulation of prohibited medication added to section 12.3.2<br><br>In section 12.5 new subgroups are added and the word ANCOVA is now replaced with ANOVA,<br><br>Section 12.5.1.3 and 12.5.1.4 describe checking of ANOVA assumptions and alternative strategies in case the assumptions are significantly violated.<br><br>Section 12.5.3.1 updated to include Pearson's correlation between fibrinogen level and MCF Clauss assay.<br><br>Plasma derived drugs removed from section 12.5.3.2 describing consumption of transfusion products after IMP until end of surgery. Summary of subjects with total avoidance of transfusion products post IMP administration added.<br><br>Section 12.5.3.7. Method to produce the confidence internal added.<br><br>Section 12.5.3.8 added to describe the analysis of overall mortality.<br><br>Section 12.6.1 updated to accommodate new adverse event analyses and subgroup analyses.<br><br>In section 12.6.3 a shift summary of hematology for number of subjects with change from normal or not clinical significant to clinical significant has been added.

PPD

Table of Contents

1.0 Approvals ... 1
Change History ... 2
Table of Contents ... 4
Abbreviations ... 6
2.0 Purpose ... 9
3.0 Scope ... 9
4.0 Introduction ... 9
4.1 Changes from Protocol ... 9
5.0 Study Objectives ... 10
5.1 Endpoints ... 11
6.0 Study Design ... 11
6.1 Inclusion/Exclusion Criteria ... 15
6.2 Sample Size Considerations ... 15
6.2.1 Sample Size Recalculation ... 16
6.3 Data Monitoring ... 16
6.4 Randomization ... 16
7.0 Study Parameters and Covariates ... 17
7.1 Primary Efficacy Parameter ... 17
7.2 Secondary Parameters ... 17
7.2.1 Efficacy ... 17
7.2.2 Exploratory Efficacy ... 18
7.2.3 Safety ... 18
7.3 Predetermined Covariates and Prognostic Factors ... 18
8.0 Definitions ... 19
8.1 Age ... 19
8.2 Baseline ... 19
8.3 Change from Baseline ... 19
8.4 Missing Data Conventions ... 19
8.4.1 Missing Severity Assessment for Adverse Events ... 19
8.4.2 Missing Relationship to Investigation Product for Adverse Events ... 19
8.4.3 Missing Seriousness Assessment for Adverse Events ... 19
8.4.4 Missing/under detection limit Laboratory values ... 19
8.5 Handling of partial dates and missing/incomplete times ... 20
8.5.1 Incomplete Start Date and Time ... 20
8.5.2 Incomplete Stop Date and Time ... 21
8.6 Subgroups ... 21
8.7 Pooling of Centers ... 22
8.8 Completed Study ... 22
8.9 End of Surgery ... 22
8.10 Study Visits ... 22
8.11 Amount of Blood Loss ... 22
8.12 Correction of Fibrinogen Level ... 23
8.13 Amount of Transfusion Products ... 24
8.14 Amount of Red Blood Cells ... 24
8.15 Post-operative Blood Loss in the First 24 hours ... 24
8.16 Rebleeds after the End of Surgery until Day 8 ... 24
8.17 Hospital Length of Stay after Surgery ... 24

PPD

8.18 In-hospital Mortality 24
8.19 Overall Mortality 24
8.20 Relative Day 24
8.21 Time to Start of First Investigational Medicinal Product Infusion 24
8.22 Total planned Investigational Medicinal Product Volume 25
8.23 Total actual Investigational Medicinal Product Administered 25
8.24 Duration of Investigational Medicinal Product Infusion 25
8.25 Relative Time in Surgery 25
9.0 Analysis Sets 25
9.1 All Subjects Enrolled Set 25
9.2 Safety Analysis Set 25
9.3 Full Analysis Set 25
9.3.1 Modified Full Analysis Set 25
9.4 Per-Protocol Set 26
10.0 Interim Analyses and Data Monitoring 26
11.0 Data Review 27
11.1 Data Handling and Transfer 27
11.2 Data Screening 27
12.0 Statistical Methods 27
12.1 Subject Disposition 27
12.2 Protocol Deviations 28
12.3 Treatments 28
12.3.1 Extent of Study Drug Exposure 28
12.3.2 Prior and Concomitant Medications or Treatment 29
12.3.3 Medical and Surgical History, and Concomitant Disease 29
12.3.4 Transfusion Products 29
12.4 Demographic and Baseline Characteristics 30
12.4.1 Surgery 30
12.5 Efficacy Analyses 30
12.5.1 Primary Efficacy Parameter 30
12.5.2 Multiplicity 32
12.5.3 Secondary Efficacy Parameters 32
12.6 Safety Analyses 34
12.6.1 Adverse Events 34
12.6.2 Deaths and Serious Adverse Events 37
12.6.3 Laboratory Data 37
12.6.4 Vital Signs 38
12.6.5 Physical Examinations 38
12.6.6 Viral Status 38
13.0 Validation 38
14.0 References 38
Appendix 1 Tables, Figures, Listings, and Supportive SAS Output Appendices 39

PPD

Abbreviations

AE	Adverse event
AESI(s)	Adverse event(s) of special interest
ANCOVA	Analysis of Covariance
ANOVA	Analysis of Variance
aPTT	Activated partial thromboplastin time
ATC	Anatomic Therapeutic Classification
AT III	Antithrombin III
BDRM	Blind Data Review Meeting
CMH	Cochran-Mantel-Haenszel
CSP	Clinical Study Protocol
DSMB	Data Safety Monitoring Board
ETP	Endogenous Thrombin Potential
eCRF	electronic Case Report Form
EU	European Union
FAS	Full Analysis Set
FFP	Fresh frozen plasma
FII	Factor II
FV	Factor V
FVII	Factor VII
FVIII	Factor VIII
FIX	Factor IX
FX	Factor X
FXI	Factor XI
FXIII	Factor XIII
F_{1+2}	Prothrombin Fragments 1+2
IMP	Investigational medicinal product
INR	International Normalized Ratio
IV	Intravenous
IWRS	Interactive Web Response System
PC	Protein C
PD	Protocol Deviation
PPS	Per-Protocol Set
PMP	Pseudomyxoma peritonei

PPD

PS	Protein S
PT	Prothrombin Time
RBC	Red Blood Cells
SAF	Safety Set
SAE	Serious Adverse Event
SAP	Statistical Analysis Plan
SD	Standard deviation
SMQ	Standard MedDRA Queries
SOC	System Organ Class
TAT	Thrombin-antithrombin III complex
TEAE	Treatment-emergent adverse event
TEE	Thromboembolic event
TFL	Tables, Figures and Listings
TGT	Thrombin Generation Test
TT	Thrombin Time
vWF	von Willebrand Factor
WBC	White Blood Cells
AE	Adverse event
AESI(s)	Adverse event(s) of special interest
ANCOVA	Analysis of Covariance
ANOVA	Analysis of Variance
aPTT	Activated partial thromboplastin time
AT III	Antithrombin III
ATC	Anatomic Therapeutic Classification
BDRM	Blind Data Review Meeting
CMH	Cochran-Mantel-Haenszel
CSP	Clinical Study Protocol
CTR	Clinical Trial Report
DSMB	Data Safety Monitoring Board
eCRF	electronic Case Report Form
ETP	Endogenous Thrombin Potential
EU	European Union
F_{1+2}	Prothrombin Fragments 1+2
FAS	Full Analysis Set
FFP	Fresh frozen plasma

PPD

FII	Factor II
FIX	Factor IX
FV	Factor V
FVII	Factor VII
FVIII	Factor VIII
FX	Factor X
FXI	Factor XI
FXIII	Factor XIII
IMP	Investigational medicinal product
INR	International Normalized Ratio
IV	Intravenous
IWRS	Interactive Web Response System
mFAS	modified Full Analysis Set
NTEAE	Non-treatment emergent adverse event
PC	Protein C
PD	Protocol Deviation
PMP	Pseudomyxoma peritonei
PPS	Per-Protocol Set
PS	Protein S
PT	Prothrombin Time
RBC	Red Blood Cells
SAE	Serious Adverse Event
SAF	Safety Set
SAP	Statistical Analysis Plan
SD	Standard deviation
SMQ	Standard MedDRA Queries
SOC	System Organ Class
TAT	Thrombin-antithrombin III complex
TEAE	Treatment-emergent adverse event
TEE	Thromboembolic event
TFL	Tables, Figures and Listings
TGT	Thrombin Generation Test
TT	Thrombin Time
vWF	von Willebrand Factor
WBC	White Blood Cells

PPD

2.0 Purpose

The statistical analysis plan (SAP) describes the statistical methods to be used during the reporting and analyses of data collected under Biotest AG Clinical Study Protocol 995.

3.0 Scope

This plan is a living document that will be created during the study start-up. The first version of the SAP will be drafted within three months of the final electronic Case Report Form (eCRF), and maintained throughout the lifecycle of the study. If any updates of the SAP are required before an interim analysis, then this version has to be approved and signed before locking any data for the interim analysis. The SAP will be finalized prior to database lock. The SAP will require sign off from the Project Manager, Biostatistician and representative of the sponsor.

The SAP outlines the following:

- Study objectives
- Study design
- Variables analyzed and analysis sets
- Applicable study definitions
- Statistical methods regarding major protocol deviations (PDs), study drug exposure, efficacy analysis, concomitant medications, adverse events (AEs) handling, laboratory data, vital signs and physical examinations
- Tables, Figures, Listings (TFLs) (defined in a separate document)

4.0 Introduction

This SAP describes the statistical methods to be used during the reporting and analyses of data collected under Biotest AG Clinical Study Protocol 995.

This SAP should be read in conjunction with the Clinical Study Protocol (CSP) and eCRF. This version of the plan has been developed using the CSP version 4.0, dated 04DEC2019 and version 4.2 UK, dated 04AUG2020 and eCRF version 3.0, dated 15OCT2020. Any further changes to the CSP or eCRF may necessitate updates to the SAP.

The purpose is to finalize a SAP so that programming can start. Versions of the SAP up to initial sponsor approval will be known as a draft SAP. If any updates of the SAP are required, for example due to changes of the CSP or eCRF, then this version has to be approved and signed before locking any data for the interim or final analysis.

4.1 Changes from Protocol

An additional mFAS population has been defined to follow ICH guidance.

The full analysis set (FAS) as defined in section 10.1 in the protocol "The FAS comprises all subjects who received at least one dose of IMP prior to the 'end of surgery' and have at least one post dose efficacy assessment", has been altered in section 9.03 of this SAP version to "All randomized subjects receiving IMP post randomization and with data collected post randomization will be included in the FAS. Exclusion from the FAS can be considered in special cases as described in ICH E9, section 5.2.1.". In addition, the previous FAS definition stemming from the protocol and defined in Section 9.0 of SAP version 5.0 is renamed to modified FAS (mFAS). These changes are done to be compatible with ICH E9 section 5.2.1.

Additional steps have been added for the efficacy section to allow for the potential of non-normal data. These are data transformations and a non-parametric Van Elteren's method as specified in Section 12.5.1. This is an extension to Section 10.5 of the protocol.

Overall Mortality has been added to the SAP in Section 12.5.3.8 as a secondary efficacy endpoint.

Per Section 10.5 of the protocol, there will be no imputation of missing values. Imputation has since been deemed necessary from SAP version 2.0. These rules are now defined in Section 8.4.

5.0 Study Objectives

The main purpose of this phase III study is to demonstrate the efficacy of BT524 as a complementary therapy to management of uncontrolled severe hemorrhage in acquired hypofibrinogenemia in subjects undergoing elective major spinal or abdominal surgery.

The primary objective of this study is to demonstrate that BT524 is non-inferior that means not worse than fresh frozen plasma (FFP)/cryoprecipitate with a non-inferiority margin of 150 mL in reducing intra-operative blood loss by intravenous (IV) administration in subjects with acquired hypofibrinogenemia undergoing elective major spinal or abdominal surgery.

If therapeutic equivalence (non-inferiority) has been demonstrated, therapeutic superiority of BT524 compared with FFP/cryoprecipitate will also be assessed.

Secondary objectives are to demonstrate the efficacy of BT524 by assessing the correction of the fibrinogen level intra-operatively, the transfusion requirements, post-operative blood loss in the first 24 hours, the number of subjects with rebleeds, the hospital length of stay and in-hospital mortality. Secondary objectives also comprise the safety of BT524 by documenting the number of AEs including changes in laboratory parameters, the viral status, and the frequency and severity of thrombosis and of thromboembolic events (TEEs).

PPD

5.1 Endpoints

**- *Efficacy***	***Primary Endpoint:***<br>• Intra-operative blood loss after decision to treat the subject with IMP until the end of surgery as measured by amount of blood from the blood suction unit and amount of blood from swabs, surgical cloths and compresses.<br><br>***Secondary Endpoints:***<br>• Proportion (%) of subjects with successful correction of fibrinogen level 15 minutes after start of first IMP administration<br>• Time to first successful correction of fibrinogen level<br>• Total amount of transfusion products (allogenic blood products) or autologous blood transfusion infused after start of first IMP administration until end of surgery<br>• Amount of red blood cells (allogenic and autologous RBCs) infused after start of first IMP administration until end of surgery<br>• Post-operative blood loss in the first 24 hours<br>• Proportion (%) of subjects with rebleeds after the end of surgery until Day 8<br>• Hospital length of stay after surgery<br>• In-hospital mortality<br><br>***Exploratory Endpoints:***<br>• Overall Mortality
**- *Safety***	***Secondary Endpoints:***<br>• AEs<br>• Changes in vital signs<br>• Changes in clinical laboratory assessments of hematology, clinical chemistry, and urinalysis<br>• Changes in clinical laboratory assessments of markers of coagulation<br>• Changes in clinical laboratory assessments of coagulation factors<br>• Frequency and severity of thrombosis and of TEEs<br>• Viral status

6.0 Study Design

This is a phase III, prospective, randomized, active-controlled, multicenter, non-inferiority clinical study in adult subjects (≥ 18 years) undergoing major spinal or abdominal surgery to demonstrate the efficacy and the safety of intra-operative use of BT524 as a complementary therapy to management of uncontrolled severe hemorrhage in acquired hypofibrinogenemia.

PPD

At least 200 subjects will be enrolled to ensure data are available for at least 100 evaluable subjects per treatment arm (BT524 or FFP/cryoprecipitate). The multicenter, multinational study will be conducted at approximately 15-20 sites in the EU and Switzerland with subjects undergoing major spinal surgery, and at one site in the United Kingdom with subjects undergoing cytoreductive pseudomyxoma peritonei (PMP) surgery.

This study will be partially blinded; surgeon, surgical staff and subjects will be blinded to treatment allocation throughout the entire surgery. The anesthesiologist who will administer the investigational medicinal products (IMP) could not be blinded to treatment allocation because of the inherent characteristics of the IMP BT524 and FFP/ cryoprecipitate.

Eligibility of subjects is assessed during screening with the exception of the inclusion criterion #5, which is assessed intra-operatively. The intraoperative inclusion criterion is different in subjects undergoing major spinal surgery (EU and Switzerland) and in subjects undergoing cytoreductive PMP surgery (United Kingdom).

Randomization of subjects to treatment will occur intra-operatively (predose) when eligibility for the clinical study has been confirmed. Subjects undergoing spine surgery will be randomized on a 1:1 basis to receive either BT524 or FFP, and subjects undergoing cytoreductive PMP surgery are to be randomized separately on a 1:1 basis to receive either BT524 or cryoprecipitate. For subjects undergoing spine surgery there will be a stratified randomization according to the predictive blood loss: > 1,000 mL to ≤ 2,000 mL and > 2,000 mL. The predictive blood loss will be recorded prior to surgery. Measurement of intra-operative blood loss will continue after decision to treat until end of surgery, and will serve as the primary efficacy parameter.

The study comprises a screening visit within 42 days prior to surgery to assess subjects eligibility, a baseline visit on the day of the surgery prior anesthesia (Day 1), the surgery phase (including randomization and single or repeated intra-operative administration of IMP, Day 1) and the follow-up phase of at least 5 weeks with 4 follow-up visits on Days 2, 3, 5 and 8 and the closing visit, including the final safety examination, scheduled on Day 36* after the day of surgery (*+35, up to Day 71 if required). The duration of individual study participation for eligible screened subjects is at least 5 weeks.

Subjects are entitled to discontinue participation in the clinical study at their own request at any time without stating a reason.

The investigator can terminate a subject's participation in the study at any time if continuation could lead to disadvantages for the subject, which cannot be justified by the investigator. Withdrawn subjects will not be replaced.

After 40 subjects have completed the study, a data monitoring of blinded aggregate data of all 40 subjects without separating the subjects according to treatment will be performed to recalculate the sample size. See section 6.2.1 for details.

In this study, three interim analyses with an alpha-adjustment according to Haybittle/Peto (Haybittle, 1971; Peto et al., 1976; Schulz and Grimes, 2005) are planned for assessment of adjusting the sample size. See section 10.0 for details.

A Data Safety Monitoring Board (DSMB) will independently review and assess the unblinded safety data throughout the entire study at regular intervals. The DSMB members can propose to stop the study at any time after a scheduled or unscheduled meeting in case of major safety concerns related to study treatment.

Further details on the assessment schedule (including the follow-up period) that will be used for assessment of the efficacy and safety parameters in this study are presented in the following flowchart of study.

PPD

Study Schedule — Day	D-42 to D-1	D-2 to D1	D1	D1	D1	D1	D2	D3	D5	D8	D36 (+35)
**Assessments — Visit**	**Screening**	**Prior surgery** (Baseline)	**Surgery**				**Follow-up**	**Follow-up**	**Follow-up**	**Follow-up**	**Closing**
			**prior 1st dose**	**pre-dose**	**post-dose**	**end of surgery**					
Informed Consent	•										
Check/re-check of inclusion / exclusion criteria	•	•[1]									
Demographic data	•										
Classification of type of surgery	•										
Recording expected blood loss		•									
Body weight	•	•[2]									
Physical examination	•	•[2]					•	•	•	•	•
Pregnancy test, only in females of childbearing potential	•	•[2]									
Medical and surgical history	•	•[3]									
Viral safety: Collection of retention sample	•										•
Virus serology (hepatitis B, hepatitis C, HIV)	•										•
Vital signs	•	•	•	•	•	•	•	•	•	•	•
Hematology and clinical chemistry	•	•[2]	•			•	•	•	•	•	•
Urinalysis	•	•[2]					•	•		•	•
Markers of coagulation (coagulation activation tests)	•	•[2]	•		•[4]	•[5]	•	•	•	•	•
Plasma concentration of fibrinogen activity (Clauss assay)	•	•[2]	•		•[4]	•[5]	•				
FIBTEM A10 (ROTEM)	•	•[2]	•	•	•[4]	•[5]	•				

[1] Diagnostic tests will be repeated at the investigator's discretion.
[2] Diagnostic tests have to be done prior to surgery. In case of short time-period between screening and baseline (≤ 2 days) these tests have only to be repeated based on medical judgment of the investigator. If not repeated, screening results will serve as baseline.
[3] Previous medication: change from screening.
[4] Tests have to be done only 15 and 90 minutes after start of 1st IMP administration.
[5] Tests for *markers of coagulation and plasma activity of fibrinogen (Clauss assay, FIBTEM A10, MCF)* have to be done '90 min after start of 1st IMP administration' and at the 'end of surgery'. In case of a short time-period between these two time-points (<30 min) these tests have only to be repeated based on medical judgment of the investigator.

PPD

**Study Schedule** **Day**	**D-42 to D-1**	**D-2 to D1**	**D1**	**D1**	**D1**	**D1**	**D2**	**D3**	**D5**	**D8**	**D36 (+35)**
**Assessments** **Visit**	**Screening**	**Prior surgery** (Baseline)	**Surgery**				**Follow-up**	**Follow-up**	**Follow-up**	**Follow-up**	**Closing**
			**prior 1st dose**	**pre-dose**	**post-dose**	**end of surgery**					
Maximum clot firmness (MCF) (ROTEM)	•	•[2]	•	•	•[4]	•[5]	•				
Coagulation factors (including vWF)		•	•		•[6]						
Intra-operative inclusion criteria			•								
**Intravenous infusion(s) of IMP (BT524 or FFP/cryoprecipitate)**			•[7]								
Recording start/end of surgery			•			•					
Continuous measurement of blood loss from start of surgery			•			•	•				
Calculation and recording of blood loss						•[8]	•[9]				
Recording time of decision to treat the subject with IMP			•								
Order of IMP			•	•							
Randomization			•								
Rebleeding episodes						•	•	•	•	•	
Concomitant medication or treatment			•			•	•	•	•	•	•
Transfusion products			•			•	•	•	•	•	•
Adverse events	•	•	•			•	•	•	•	•	•

[6] Test has to be done only 90 minutes after start of 1st IMP administration.
[7] Total volume and total infusion time (start and end of each infusion) to be recorded.
[8] Intra-operative blood loss from time-point of decision to treat the patient with IMP until end of surgery.
[9] Recording of blood loss (drainage volume) until 24 hours after end of surgery.

PPD

6.1 Inclusion/Exclusion Criteria

Only subjects meeting all of the following **inclusion criteria** will be considered for study inclusion (will be considered for randomization to study treatment):

1. Written informed consent obtained from subjects indicating that they understand the purpose of and procedures required for the study and are willing to participate in it
2. Subjects scheduled for elective major spinal or cytoreductive PMP surgery with expected major blood loss
3. Male or female, aged ≥ 18
4. No increased bleeding risk as assessed by standard coagulation tests and medical history
5. Intra-operative trigger for treatment
    a. Subjects undergoing spinal surgery: Intra-operative clinically relevant bleeding of approximately 1 L, requiring hemostatic treatment during surgery.
    b. Subjects undergoing cytoreductive PMP surgery: Intra-operative prediction of clinically relevant bleeding of > 2 L (approximately 60 minutes after the start of cytoreductive PMP surgery), requiring hemostatic treatment during surgery.

Subjects having any of the following **exclusion criteria**, either at screening and/or at baseline will not be included in the study (will not be randomized to study treatment):

1. Pregnancy or unreliable contraceptive measures or breast feeding (women only)
2. Hypersensitivity to proteins of human origin or known hypersensitivity reactions to components of the IMP
3. Participation in another clinical study within 30 days before entering the study or during the study and/or previous participation in this study
4. Treatment with any fibrinogen concentrate and/or fibrinogen-containing product within 30 days prior to infusion of BT524
5. Employee or direct relative of an employee of the Contract Research Organization, the study site, or Biotest
6. Inability or lacking motivation to participate in the study
7. Medical condition, laboratory finding (e.g., clinically relevant biochemical or hematological findings outside the normal range), or physical exam finding that in the opinion of the investigator precludes participation
8. Presence or history of venous/arterial thrombosis or TEE in the preceding 6 months

6.2 Sample Size Considerations

The non-inferiority margin is defined as 150 mL blood loss, as such difference in blood loss after the decision to treat the subjects with IMP is considered as clinically not relevant.

It is assumed that BT524 is non-inferior that means not worse than FFP/cryoprecipitate with a non-inferiority margin of 150 mL in reducing intra-operative blood loss. Assuming a blood loss of about 500 mL in the FFP/cryoprecipitate treatment arm after the decision to treat the subject with IMP until end of surgery, a standard deviation (SD) of 375 mL, a non-inferiority margin of 150 mL, an alpha-level of 2.5% (1-sided) 100 evaluable subjects per treatment arm are needed to demonstrate the non-inferiority of BT524 by using a t-test (equivalence) with 80% power.

PPD

Based on the results of the blinded sample size recalculation (see section 6.2.1) the power was reduced from 90% to 80% to allow an increase of the originally assumed SD of 325 mL to 375 mL.

The sample size will be recalculated at the interim analyses as defined in section 10.0. Sample size estimations will be performed using nQuery Advisor Version 4.0 or higher.

With 100 subjects per treatment arm superiority of BT524 can also be tested with a power of > 80% (t-test, alpha=0.05 2-sided, effect size Δ=0.5).

It is expected that approximately 10% of the randomized subjects will not fulfil the criteria to be included in the PPS. Therefore, approx. 220 randomized subjects are required to reach 200 evaluable subjects in the PPS.

6.2.1 Sample Size Recalculation

After 40 subjects had completed the study, the overall mean and SD for the primary efficacy variable intra-surgery blood loss after decision to treat the subject with IMP was derived using blinded aggregate data of all 40 subjects without separating according to treatment.

If the assumed mean and SD blood loss are not reflected in these subjects, then a sample size adjustment had to be considered to ensure that a sufficient number of subjects will be randomized to maintain a power of 90%.

If an adaption of the sample size is intended, this had to be documented in a protocol amendment.

The sample size recalculation was performed. The CSP (version 4.0) and the sample size considerations were updated (see section 6.2). The power was reduced to 80%.

6.3 Data Monitoring

This data monitoring is not an interim analysis because the analysis is performed with all subjects without separating the subjects according to treatment. Therefore, no alpha-adjustment is necessary.

6.4 Randomization

There will be a stratified randomization per surgery type.

Subjects undergoing spine surgery are to be randomized on a 1:1 basis to receive either BT524 or FFP, and subjects undergoing cytoreductive PMP surgery are to be randomized separately on a 1:1 basis to receive either BT524 or cryoprecipitate.

For subjects undergoing spine surgery the randomization will be stratified according to the predictive blood loss: > 1,000 mL to ≤ 2,000 mL and > 2,000 mL. The predictive blood loss will be recorded prior surgery.

Randomization of subjects to treatment will occur intra-operatively (predose) when eligibility for the clinical study has been confirmed.

<u>For subjects undergoing spine surgery the following applies:</u>

> After an intra-operative blood loss of approximately 1 L, requiring hemostatic treatment (high risk for the need of fibrinogen supplementation with BT524 or FFP) during surgery, the randomization request will be sent to the pharmacy (if applicable). The pharmacy retrieves the randomization code via Interactive Web Response System (IWRS) and provides the prepared IMP to the unblinded anesthesiologist. In case the anesthesiologist retrieves the randomization code via IWRS, the pharmacy will be informed accordingly and can provide the IMP.

<u>For subjects undergoing cytoreductive PMP surgery the following applies:</u>

> Randomization of PMP subjects to treatment will occur intra-operatively (pre-dose) when eligibility for the clinical study has been confirmed. Immediately after the prediction of clinically relevant bleeding > 2 L, requiring hemostatic treatment (high risk for the need of fibrinogen supplementation with BT524 or cryoprecipitate) during surgery, the person responsible for randomization will be informed and the randomization request will be sent. The person responsible for randomization retrieves the

PPD

randomization code via IWRS and informs the blood bank/pharmacy. BT524 will be delivered to the operating room by the pharmacy. After randomization, BT524 will be prepared in the operating room in accordance with the manufacturer's instructions and administered immediately after reconstitution by the unblinded anesthesiologist.

After randomization, cryoprecipitate will be thawed and prepared for administration by the local blood bank, delivered to the operating room, and administered upon arrival.

In case the anesthesiologist retrieves the randomization code via IWRS, the blood bank/ pharmacy will be informed accordingly and can provide the IMP.

Subject Identification

For the coherent assignment of the study, documents all subjects having signed the informed consent and having entered the screening period will receive a subject number. The subject number comprises a five-digit number of which the first two digits define the investigational site and the last three digits the subject enrolled at the corresponding site. Subject numbers are assigned consecutively per site. Subject numbers are assigned unique and will not be replaced i.e., in case of a screening failure.

An interactive web/voice response system will be implemented and used for randomization and re-supply. Detailed instructions for the use of IWRS systems are provided in a separate document that will be filed in the Investigator Site File.

The random allocation of treatments to subjects will be done using a computerized randomization program. Subjects will receive a randomization number, which will be recorded along with the date of randomization in the eCRF.

7.0 Study Parameters and Covariates

7.1 Primary Efficacy Parameter

The primary efficacy parameter is determined from the intra-operative blood loss after the decision to treat the subject with IMP until the end of the surgery as measured by the amount of blood from the blood suction unit and the amount of blood from swabs, surgical cloths and compresses. The end of surgery is defined as time of last suture.

7.2 Secondary Parameters

7.2.1 Efficacy

Secondary efficacy will be determined using the following parameters:

- Proportion (%) of subjects with successful correction of fibrinogen level (FIBTEM A10) 15 minutes after start of first IMP administration.
  Successful correction of fibrinogen level in a subject is defined as restoring fibrinogen FIBTEM A10 baseline level to at least 95% measured by ROTEM 15 minutes after start of first IMP administration. A correction of at least 95% is considered successful, as measurement methods has a coefficient of variation of about 5% (Solomon et al., 2015).
- Time to first successful correction of fibrinogen level (15 minutes or 90 minutes after start of first IMP administration, end of surgery, not within surgery).
- Total amount (volume in mL and number of units [bags]) of transfusion products (allogeneic blood products) or autologous blood transfusion infused after start of first IMP administration until end of surgery. The end of surgery is defined as time of last suture.
- Amount (volume in mL and number of units [bags]) of red blood cells (RBCs) (allogeneic and autologous) infused after start of first IMP administration until end of surgery. The end of surgery is defined as time of last suture.

- Post-operative blood loss in the first 24 hours.
- Proportion (%) of subjects with rebleeds after the end of surgery until Day 8. Rebleeds are defined as any bleed requiring hemostatic treatment (including reoperation) after the end of surgery until Day 8.
- Hospital length of stay after surgery, defined as the date of discharge minus the date of surgery.
- In-hospital mortality.

7.2.2 Exploratory Efficacy

Exploratory efficacy endpoints:

- Overall Mortality

7.2.3 Safety

Safety and tolerability in this clinical study will be addressed by the following safety parameters:

- Frequency, severity, seriousness and causality of AEs.
- Changes in vital signs (including pulse [heart rate], blood pressure, respiratory rate, body temperature).
- Change in clinical laboratory assessments of hematology, clinical chemistry and urinalysis.
- Change in clinical laboratory assessments of markers of coagulation:

  Prothrombin time (PT)/International Normalized Ratio (INR), activated partial thromboplastin time (aPTT), thrombin-antithrombin III complex (TAT), prothrombin fragments 1+2 (F1+2), D-dimer, protein S (PS), protein C (PC), antithrombin III (AT III), thrombin time (TT) and Endogenous Thrombin Potential (ETP) measured by Thrombin Generation Test (TGT) (TGT only for subjects undergoing cytoreductive PMP surgery).

- Change in clinical laboratory assessments of coagulation factors:

  Factor II (FII), factor V (FV), factor VII (FVII), factor VIII (FVIII), factor IX (FIX), factor X (FX), factor XI (FXI), factor XIII (FXIII) and von Willebrand factor (vWF) (vWF only for subjects undergoing cytoreductive PMP surgery).

- Frequency, severity, seriousness and causality of thromboses and TEEs, of relevant bleeding complications, of Hypersensitivity/ anaphylactic reactions, and of bleeding related ischaemic events.
- Change in viral status.

7.3 Predetermined Covariates and Prognostic Factors

In addition to treatment as a prognostic factor, there is a predetermined covariate for subjects undergoing spinal surgery only. The stratification factor predicted blood loss (> 1,000 mL to ≤ 2,000 mL and > 2,000 mL) is a covariate for the analysis of the primary efficacy variable and two secondary efficacy variables: amount of RBCs infused after IMP, post-operative blood loss in the first 24 hours.

Predicted blood loss will also be used as stratification factor in the analyses of two secondary efficacy variables: proportion of subjects with successful correction of fibrinogen level 15 minutes after start of first IMP administration, proportion of subjects with rebleeds after the end of surgery until Day 8.

8.0 Definitions

8.1 Age

Age will be calculated as:

Age = (date of informed consent – date of birth) / 365.25 rounded to lowest integer

Only year of birth will be recorded. The date will be imputed as 01 January. The imputed date of birth will be used for age calculation.

8.2 Baseline

If not stated otherwise, the last non-missing valid observation prior to surgery will serve as the baseline measurement.

8.3 Change from Baseline

Change from baseline will be calculated by statistical programming as follows:

Change from baseline = Post-baseline measurement – baseline measurement

8.4 Missing Data Conventions

In this short study, not many missing values are expected. Therefore, in general, data will not be imputed.

In case there will be at least 10% missing data, these data will be checked at the Blind Data Review Meeting (BDRM) and imputation methods will be considered. It is assumed that these missing data will be at random and an imputation method which is based on the worst case of the respective treatment group will be used as a sensitivity analysis.

For binary endpoints, an observed case analysis (excluding missing data) will be considered to be the primary analysis method and a non-responder analysis (treating missing values as non-responders or the worst case) may be performed as a sensitivity analysis if deemed necessary.

Imputed values will be flagged in listings.

8.4.1 Missing Severity Assessment for Adverse Events

If severity is missing for an AE starting prior to the administration of IMP, then a severity of “Mild” will be assigned. If the severity is missing for an AE starting on or after administration of IMP, then a severity of “Severe” will be assigned. The imputed values for severity assessment will be used for incidence summaries, while the actual values will be used in data listings.

8.4.2 Missing Relationship to Investigation Product for Adverse Events

If the relationship to IMP is missing for an AE starting on or after the administration of IMP, a causality of “Related” will be assigned. The imputed values for relationship to IMP will be used for incidence summaries, while the actual values will be presented in data listings.

8.4.3 Missing Seriousness Assessment for Adverse Events

If no information about seriousness is available, the AE will be considered serious. The imputed values for seriousness assessment will be used for incidence summaries, while the actual values will be used in data listings.

8.4.4 Missing/under detection limit Laboratory values

Laboratory values that are "not measurable" or "under detection limit" should not be replaced and should not be included in the summary tables. Quantitative laboratory parameters reported as “< x” or “> y” will be imputed with x/2 and y, respectively, for inclusion in summary statistics. The reported value will be presented in listings.

PPD

8.5 Handling of partial dates and missing/incomplete times

For prior or concomitant medications, incomplete (i.e., partially missing) start date and/or stop date will be imputed. When the start date and the stop date are both incomplete for a subject, impute the start date first.

For AEs, incomplete (i.e., partially missing) start dates and times and/or incomplete stop dates and times will be imputed.

For transfusion products, incomplete start and/or stop times will be imputed.

Imputation rules for incomplete dates for prior or concomitant medications, incomplete dates and times for AEs, and incomplete times for transfusion products are described in sections 8.5.1 and 8.5.2.

Imputed dates will be used for all further derivations (e.g., treatment-emergent adverse event [TEAE] definition, classification of medications as prior or concomitant, and study day calculation). Imputed dates will also be used and flagged in listings.

Imputed dates will be indicated in the listings by using 'D' if only day is imputed, and 'M' if day and month are imputed, Imputed times will be indicated in listings by using 'm' if only minutes are imputed and 'h' if minutes and hours are imputed. It is not expected that dates for any other parameter are missing. Should any other dates be missing or partially missing, then statistical programming will place a warning in the log file and inform the statistical lead should these dates be required for derivations.

8.5.1 Incomplete Start Date and Time

The following rules will be applied to impute the missing numerical fields. If the stop date is complete and the imputed start date is after the stop date, then the start date will be imputed using the stop date.

Missing Day and Month

- If the year of the incomplete start date is the same as the year of the date of the first dose of IMP, then the day and month of the date of the first dose of IMP will be assigned to the missing fields.
- If the year of the incomplete start date is before the year of the date of the first dose of IMP, then December 31 will be assigned to the missing fields.
- If the year of the incomplete start date is after the year of the date of the first dose of IMP, then 01 January will be assigned to the missing fields.
- If the subject was not treated (no IMP start date), then 01 January will be assigned to the missing fields.

Missing Month Only

- The day will be treated as missing and both month and day will be replaced according to the above procedure.

Missing Day Only

- If the month and year of the incomplete start date are the same as the month and year of the date of the first dose of IMP, then the day of the date of the first dose of IMP will be assigned to the missing day.
- If either the year is before the year of the date of the first dose of IMP or if both years are the same but the month is before the month of the date of the first dose of IMP, then the last day of the month will be assigned to the missing day.
- If either the year is after the year of the date of the first dose of IMP or if both years are the same but the month is after the month of the date of the first dose of IMP, then the first day of the month will be assigned to the missing day.
- If the subject was not treated (no IMP start date), then the first of the month will be assigned to the missing field.

Missing/Incomplete Time

- If the time is missing and the date is complete and is the same as the date of the first dose of IMP, or the date is imputed to be this date, then the time will be set to the time of the first dose of IMP. Otherwise, missing times will be imputed as 00:00.
- If the minutes are given but the hour is not, then the time will be regarded as completely missing and handled as above.
- If the hour is given but the minutes are not, then if the hour is the same as the hour of the first dose of IMP, then the minutes of that dose of IMP will be assigned to the missing fields. Otherwise 00 will be assigned to the missing minutes.
- If the subject was not treated (no IMP start date), then missing times will be imputed as 00:00.

8.5.2 Incomplete Stop Date and Time

The following rules will be applied to impute the missing numerical fields. If the imputed stop date is before the start date (imputed or non-imputed start date), then the imputed stop date will be equal to the start date.

Missing Day and Month

- If the year of the incomplete stop date is the same as the year of the date of the study discontinuation/completion, the stop day and month will be set to the maximum of the date of study discontinuation/completion, as appropriate, or the date equivalent to 35 days after the last dose of IMP.
- If the year of the incomplete stop date is before the year of the date of study discontinuation/ completion, as appropriate, or the date equivalent to 35 days after the last dose of IMP, the day and month will be set to 31 December.
- If the year of the incomplete stop date is after the year of the date of study discontinuation/ completion, as appropriate, or the date equivalent to 35 days after the last dose of IMP, the day and month will be set to 01 January.

Missing Month Only

- The stop day will be treated as missing and both month and day will be replaced according to the above procedure.

Missing Day Only

- The stop day will be set to the last day of the month.

Totally Missing Stop Date

- The concomitant medication will be considered ongoing.

Missing /Incomplete Time

- If the time is missing, it will be imputed as 23:59.
- If the minutes are given, but not the hour, then the time will be regarded as completely missing and handled as above.
- If hours are given, but minutes are not, minutes will be imputed with 59.

8.6 Subgroups

The subgroup type of surgery (spinal and abdominal) is analyzed for the primary as well as for all secondary endpoints. Subgroups sex and race are analyzed for the primary endpoint.

Further subgroup analysis according to the predictive blood loss is planned for two secondary efficacy variables: total amount of transfusion products after IMP administration until end of surgery, total amount of RBC after IMP administration until end of surgery.

PPD

For specific adverse events and other safety parameters, subgroup analyses are being conducted based on type of surgery, sex and race. For more information, please refer to section 12.6.1.

8.7 Pooling of Centers

In case of low number of subjects per center, summaries of data by center would be unlikely to be informative. Therefore, data from all centers per country/region (if applicable) and in total will be pooled prior to analysis. Only enrollment will be summarized by country and center.

8.8 Completed Study

A subject is considered to have completed the study when he/she is presumed to have followed the CSP (i.e., completed visits approximately 5 weeks after surgery).

8.9 End of Surgery

End of surgery is defined as time of last suture.

8.10 Study Visits

The labels of study visits in this study are as following:

- Screening
- Baseline
- Surgery Day 1 (Prior 1st dose)
- Surgery Day 1 (Pre-dose)*
- Surgery Day 1 (15 min after IMP start)
- Surgery Day 1 (90 min after IMP start)
- Surgery Day 1 (End of Surgery)
- Day 2, Follow-up
- Day 3, Follow-up
- Day 5, Follow-up
- Day 8, Follow-up
- Closing Visit
- Early Discontinuation

*only applicable in case of repeated intra-operative IMP administration

For a subject who did not complete the study, date of early discontinuation is the last visit, when the subject discontinued from the study. As subjects are expected to attend a Closing Visit whether they complete or discontinue early from the study, subjects who complete the study will have their Closing Visit data summarized under ‘Closing Visit’, while subjects who discontinue early from the study will have their Closing Visit data summarized under ‘Early Discontinuation’. If a subject terminates the study prematurely without having a closing visit, the data from the last regular visit will be summarized under this visit.

8.11 Amount of Blood Loss

During surgery, the blood loss will be quantified by measuring the continuous bleeding mass with a blood suction unit (and/or a cell saver) and by calculation of the amount of blood from swabs, surgical cloths and compresses. For the primary endpoint, the amount of blood will be measured for the time between decision to treat and end of surgery.

Calculation of blood loss:

Blood loss (mL) = Volume of blood on swabs, surgical cloths and compresses (mL) + Volume of blood in the suction container (mL) + Total volume of blood loss from other sources (mL)

Volume of blood on swabs, surgical cloths and compresses (mL) = Weight of used/unused surgical cloths and compresses (g) - Weight of dry swabs, surgical cloths and compresses (g). 1 g weight represents 1mL volume of blood.

Volume of blood in the suction container (mL) = Total volume of fluid collected in suction container (mL) - Total volume of anticoagulant solution in blood suction container (mL)* - Total volume of irrigation solution (mL)

* Not applicable for abdominal surgery

Total volume of irrigation solution = Capacity of irrigation syringe (mL) * Number of times irrigation syringe used

<u>Handling of missing data:</u>

For the calculation of **Blood loss**, Volume of blood on swabs, surgical cloths and compresses and Volume of blood removed from the surgical field by the blood suction is required. If there is at least one missing value (see above for single items needed) the blood loss cannot be calculated, and the volume will be missing.

For the calculation of blood in the suction container: Total volume of anticoagulant solution in blood suction container is not applicable for abdominal surgery and will not be considered for the calculation.

For the calculation of Total volume of irrigation solution: If Capacity of irrigation syringe is missing and Number of times irrigation syringe used > 0 then volume is missing. If Capacity of irrigation syringe is provided and Number of times irrigation syringe used is missing, then volume is missing. If Capacity of irrigation syringe is missing and Number of times irrigation syringe used = 0 then volume is 0.

8.12 Correction of Fibrinogen Level

The fibrinogen level is assessed by FIBTEM A10 test, MCF and Clauss assay at the following visits: screening, prior surgery (baseline), Surgery Day 1 (Prior 1st dose), Surgery Day 1 (Predose)*, Surgery Day 1 (15 minutes after start of first IMP administration), Surgery Day 1 (90 minutes after start of first IMP administration), Surgery Day 1 (End of surgery), and Day 2, Follow-up Visit. (* only applicable in case of repeated intra-operative IMP administration).

Rate of fibrinogen restoring = fibrinogen level at a post-baseline visit / fibrinogen level at baseline*100

If the rate of fibrinogen restoring is ≥ 95%, then the fibrinogen level is considered to be successfully corrected at that post-baseline visit for the subject.

The above rate will be calculated for the following visits: Surgery Day 1 (15 minutes after start of first IMP administration), Surgery Day 1 (90 minutes after start of first IMP administration), Surgery Day 1 (End of surgery), and Day 2, Follow-up.

The time to first successful correction of fibrinogen level is classified into the following categories: "Within 15 minutes after IMP start", "Greater than 15 and less than or equal to 90 minutes after IMP start", “Greater than 90 minutes after IMP start” and “Unsuccessful correction”.

At every visit, the time point relative to the start time of IMP is calculated in minutes as Collection Time of sample minus Start Time of IMP. Any corrections <15 minutes after IMP start are not considered for classification.

The earliest time point, when the rate is ≥ 95%, is the time to first successful correction of fibrinogen level and will be used to categorize the subjects into the respective categories.

Subjects with time to first successful fibrinogen correction on Follow-up Visit Day 2 will be assigned to the category "Greater than 90 minutes after IMP start". If the rate of fibrinogen restoring is < 95% at all the time points, correction of fibrinogen level is reported as “Unsuccessful correction”.

A sensitivity analysis adds a time window of ±3 minutes to the category “Within 15 minutes after IMP start”, so that time points ≥12 and ≤18 minutes fall into this category. To the category “Greater than 15 and less

than or equal to 90 minutes after IMP start" a time window of +3 minutes is added, so that time points between >18 and ≤93 minutes fall into this category. Corrections >93 minutes are categorized as "Greater than 90 minutes after IMP start".

In addition, and independent from the above categories, the time to first successful correction is classified into "Until end of surgery" or "Not within surgery". Subjects with time to first successful correction at the time point Surgery Day 1 (End of surgery) are assigned to "Until end of surgery". Subjects with time to first successful correction on Follow-up Visit Day 2 are assigned to "Not within surgery". For the visits Surgery Day 1 (15 minutes after start of first IMP administration) and Surgery Day 1 (90 minutes after start of first IMP administration) the time point is compared with the End time of Surgery and subjects are assigned to "Until end of surgery" or "Not within surgery" accordingly. If the rate of fibrinogen restoring is < 95% at all the time points, correction of fibrinogen level is reported as "Unsuccessful correction".

The whole analysis for the correction of fibrinogen levels is also done based on results obtained from MCF and Clauss assay to perform sensitivity analyses.

8.13 Amount of Transfusion Products

All types of transfusion products administered, including RBCs will be counted. The volumes and units (bags) of all these products will be summarized by type of transfusion product, and by treatment arm.

8.14 Amount of Red Blood Cells

The volumes and units (bags) of allogeneic RBCs and autologous blood transfusion/cell salvage will be counted separately.

8.15 Post-operative Blood Loss in the First 24 hours

The plasma drainage tube and disposable drainage bag will be emptied 24 hours after the end of surgery. The post-operative drainage volume in the first 24 hours is collected in eCRF.

8.16 Rebleeds after the End of Surgery until Day 8

Rebleeds are defined as any bleed requiring hemostatic treatment (including reoperation) after the end of surgery until Day 8, which are recorded in eCRF.

8.17 Hospital Length of Stay after Surgery

Length of stay after surgery (days) = date of discharge – date of surgery

Where date of discharge is the date of discharge following the IMP treated surgery.

8.18 In-hospital Mortality

In-hospital mortality is defined as death occurring during the hospital stay.

8.19 Overall Mortality

Overall mortality is defined as death occurring during study duration.

8.20 Relative Day

If the assessment date is prior to the date of IMP administration, the relative day is date of assessment minus date of IMP administration. If the assessment date is on the date of IMP administration or after that, then the relative day is the date of assessment minus date of IMP administration plus 1.

8.21 Time to Start of First Investigational Medicinal Product Infusion

Time to start of first IMP infusion = Start time of first IMP administration - Time of decision to treat subject with IMP.

PPD

8.22 Total planned Investigational Medicinal Product Volume

Total planned IMP volume = Sum of all planned IMP volume.

8.23 Total actual Investigational Medicinal Product Administered

Total actual IMP administered = Sum of all actual IMP administered.

8.24 Duration of Investigational Medicinal Product Infusion

Duration of XXX IMP administration = End time of XXX IMP administration - Start time of XXX IMP administration

Where XXX is the first, second … administration.

8.25 Relative Time in Surgery

Duration of Surgery = End time of surgery - Start time of surgery

Time until decision to treat = Time of decision to treat subject with IMP - Start time of surgery

Time to end of surgery after decision to treat = End time of surgery - Time of decision to treat subject with IMP

Time of IMP exposure during surgery = End time of surgery - Time of start of first IMP administration

9.0 **Analysis Sets**

9.1 All Subjects Enrolled Set

The All Subjects Enrolled Set includes all subjects who have given informed consent to this study.

9.2 Safety Analysis Set

The Safety Analysis Set (SAF) comprises all subjects who have received at least one dose of IMP. Subjects will be analyzed according to the treatment received.

9.3 Full Analysis Set

All randomized subjects receiving IMP post randomization and with data collected post randomization will be included in the FAS. Exclusion from the FAS can be considered in special cases as described in ICH E9, section 5.2.1. For the purpose of this trial, there are two FAS: FAS and mFAS. All subjects will by default be included and analyzed according to the randomized treatment allocation in both definitions.

The decision to exclude subjects from FAS will be done at a data review meeting after reviewing the data as described in the Data Review Meeting Plan.

9.3.1 Modified Full Analysis Set

As per the section above, all randomized subjects will be included in the modified full analysis set (mFAS) who meet the following conditions.

1. Subjects who received at least one dose of IMP prior to the ‘end of surgery’ and
2. have at least one post-dose efficacy assessment.
3. This includes all subjects whose IMP infusion started prior to the end of surgery, irrespective of the amount of IMP infused.

Subjects will be analyzed as randomized. The decision to exclude subjects from mFAS will be done at a data review meeting after reviewing the data as described in the Data Review Meeting Plan.

PPD

9.4 Per-Protocol Set

The Per-Protocol Set (PPS) is a subset of FAS and includes all subjects who are compliant with the CSP without any major PDs thought to have the potential to impact the results of the efficacy analysis, e.g., no treatment with IMP, incomplete treatment with IMP (administration of the 1st IMP dose is not completed if the end of the 1st IMP administration is after the end of surgery), treatment with IMP after the 'end of surgery', no post-dose efficacy assessment for the primary endpoint. Classification of PDs as major or minor will be agreed upon at the BDRM prior to database lock and also prior to each Interim Analysis for eligible subjects.

Subjects will be analyzed according to the treatment received.

10.0 Interim Analyses and Data Monitoring

To ensure subject safety, the safety data will be evaluated by a DSMB at regular intervals. A DSMB charter and list of required tables and listings is available (see Appendix 1).

After 40 subjects have completed the study, a blinded sample size recalculation will be performed. See section 6.2.1 for details. A specification of required table and listing is available (see Appendix 1).

In this study, three interim analyses of the observed blood losses are planned to have the option of adjusting the sample size needed.

For this purpose, an alpha-adjustment according to Haybittle/Peto (Haybittle, 1971; Peto et al., 1976; Schulz and Grimes, 2005) is planned. This leads to local alpha levels of 0.001 for each interim analysis and a significance level of 0.05 for the final analysis to reach a global significance level of 5%.

Past interim analyses were based on the PPS, and past sensitivity analyses were based on the mFAS.

The first interim analysis is planned with approximately 50 spine subjects, the second one with at least 40 cytoreductive PMP subjects and all other evaluable spine subjects at that time point. The third interim analysis is planned after approximately 80% of subjects of the total sample size.

Aim of all interim analyses is to adapt the sample size according to the observed blood losses and the SDs:

a) Early termination due to non-inferiority of BT524 in comparison with the used standard therapies.
b) Continuation with the sample size as initially planned.
c) Adjustment of sample size to take into account changes from the previous assumptions on the additional blood loss.
d) Stopping the study early due to futility if the sample size re-estimation indicates a much higher number than planned before.

As soon as the approximate required number of subjects for an interim analysis has received at least one dose of IMP prior to the end of surgery, completed treatment, the relevant data points (including measurement and documentation of blood loss between decision to treat and end of surgery) have been satisfactorily reviewed and the corresponding items/forms have been frozen, a data snapshot will be taken. Further details are specified in the Planned Data Deliverables section of the Data Management Plan. Classification of PDs as major or minor will be agreed PD review meeting and then reviewed during the BDRM meeting based on the snapshot data.

At each interim analysis, the tables and listings which are specified in the document: Biotest 995 TFL Specifications for Interim Analysis of Sample Size (see Appendix 1) will be produced.

For the interim analysis, the sample size will be recalculated according to the observed blood losses and the SDs using nQuery Advisor version 4.0 or higher.

PPD

The interim analysis will be based on unblinded data. The analysis will be performed by an Independent Interim Analysis Team. The interim analysis outputs will be created in a secure, restricted-access storage area on the PPD SAS file-server. All derived datasets, analysis outputs, email communications, etc. created as part of an interim analysis and containing the potential to unblind or result in operational bias, must be handled in a manner such that the Project Team has no access to them. The Independent Reporting Statistician files the final datasets, and any email communication in a secure, restricted-access storage area.

The Independent Reporting Statistician shares only the newly calculated sample size and the recommendation (see rules above) from the interim analysis results with the Sponsor and PPD study team.

In the final BDRM meeting the need for additional subgroup definitions related to length between decision to treat and end of surgery will be evaluated. If required, additional subgroups will be added to the SAP and shells in a new SAP version prior to database lock and the final evaluation.

11.0 Data Review

11.1 Data Handling and Transfer

Please see the Data Management Plan.

11.2 Data Screening

Beyond the data screening built into the PPD Data Management Plan, the PPD programming of analysis datasets and TFLs provides additional data screening. Presumed data issues will be output into SAS logs identified by the word “Problem” and extracted from the logs by a SAS macro and sent to Data Management.

Review of dry run TFL allow for further data screening prior to lock. The PPD statistician and the sponsor must approve database lock.

12.0 Statistical Methods

All analyses will use SAS® version 9.4 or higher.

Subject data listings will be ordered by randomized or actual treatment (‘Not randomized’ / ‘Not treated’, ‘BT524’, ‘FFP’, ‘Cryoprecipitate‘), subject number (numerically, ascending) and visit of assessment respectively where applicable. All tables will be presented by treatment arm (‘BT524’, ‘FFP/Cryoprecipitate‘) and overall. Where applicable tables will also be presented by treatment (‘BT524’, ‘FFP’, ‘Cryoprecipitate‘) or by surgery (spinal, abdominal).

Unless otherwise noted, categorical variables will be summarized using number of observations (n), counts and percentages within category. Percentages will be rounded to one decimal place; except 100% will be displayed without any decimal places and percentages will not be displayed for zero counts.

Unless otherwise noted, continuous variables will be summarized using the number of observations (n), arithmetic mean, SD, minimum, Q1, median, Q3 and maximum. The minimum and maximum values will be displayed to the same level of precision as the raw data, the mean, median, Q1 and Q3 to a further decimal place and the SD to two additional decimal places. Unless stated otherwise the calculation of percentages will be based on the total number of subjects in the population of interest. Thus counts of missing observations will be included in the denominator and presented as a separate category.

The significance level will be 2.5% (one-sided), confidence intervals will be 95% (two-sided). All statistical tests (p-values) will be two-sided, unless otherwise stated.

12.1 Subject Disposition

The number of subjects screened and, the number and percentage who failed screening prior to surgery or during surgery, together with the main reasons, will be summarized for the All Subjects Enrolled Set. The number of subjects randomized, number and percentage of subjects treated with IMP, number and

percentage of subjects whose treatment was unblinded during the surgery will be summarized by treatment, treatment arm and overall for the All Subjects Enrolled Set. The subject disposition summary will be repeated for spinal and abdominal surgery.

The number of subjects included in each subject analysis set will be summarized by treatment arm and overall for the SAF. The number and percentage of subjects who completed the study or prematurely withdrew/discontinued from the study with the reason for withdrawal/discontinuation will be summarized by treatment, treatment arm and overall for the SAF.

The number and percentage of subjects treated at each country and center will be summarized by treatment, treatment arm and overall for the SAF.

A listing of details of whether the patients completed or discontinued study prematurely will be presented for the SAF. The informed consent and eligibility will be listed for the All Subjects Enrolled Set. The randomization data will be listed for the All Subjects Enrolled Set. A listing of analysis set allocation will also be created for the All Subjects Enrolled Set.

12.2 Protocol Deviations

Deviations from the CSP will be documented on an on-going basis during conduct of the clinical study based on monitoring reports (e.g., failure of eligibility criteria), data management checks and statistical programming (e.g., prohibited medications based on drug codes). Protocol deviations will be discussed and agreed in the BDRM to find PDs with major impact on subject safety or the validity of the study data. Subjects with major PDs will be excluded from the PPS under the assumption that the deviation may have an impact on the efficacy analysis.

In general, PDs will be considered according to the following general categories:

- Inclusion criteria
- Exclusion criteria
- Study drug
- Assessment – safety
- Laboratory/ endpoint data
- Visit window
- Prohibited concomitant medications
- Overdose/ misuse
- Informed consent
- Other

Protocol deviations will be summarized by treatment arm and overall and listed for the FAS. The protocol deviation summary will be repeated for spinal and abdominal surgery.

12.3 Treatments

12.3.1 Extent of Study Drug Exposure

After a bleeding (risk) assessment only subjects requiring hemostatic treatment (high risk for the need of fibrinogen supplementation with BT524 or FFP/cryoprecipitate) during surgery, will be randomized and treated with BT524 or FFP/cryoprecipitate. If the bleeding remained unchanged and an ongoing relevant blood loss will be confirmed or the hemostatic control is not considered sufficient and requires further intervention or a new major blood loss occurred in the course of the surgery, subjects can be treated with repeated IMP administration according to their randomized treatment group.

As the IMP (BT524 or FFP/cryoprecipitate) will be administered intravenous (IV) to each subject under the supervision of the unblinded anesthesiologist, the compliance is expected to be 100%. In addition, the assessment of plasma fibrinogen concentrations may also serve as an adherence measure.

Exposure of IMP will be summarized by treatment separately for the SAF and repeated by surgery type (spinal or abdominal).

Descriptive statistics will be provided by treatment for the

- number and frequency of IMP administration(s) during surgery
- planned volume of IMP for each (1st, 2nd, …) infusion
- actual volume of IMP administered for each (1st, 2nd, …) infusion
- total planned volume of IMP administered during surgery
- total actual volume of IMP administered during surgery
- time to start of first IMP infusion
- duration of each (1st, 2nd, …) IMP infusion
- number of interruption(s) during surgery for those subjects whose treatment administration was interrupted for the SAF
- actual gram of IMP (BT524) received by type of surgery and total

A listing will be created by subject number for infusion administration of IMP for the SAF.

12.3.2 Prior and Concomitant Medications or Treatment

The most updated version of the World Health Organization drug dictionary will be used to classify prior and concomitant medications by preferred term. Medications will also be coded with the lowest available Anatomical Therapeutic Chemical (ATC) classification system (level 4 to level 2).

Prior medication is defined as any medication with start date prior to IMP start date, irrespective of the stop date. Prior medications will be summarized by ATC classification and preferred term in treatment arms and overall for the SAF. The number and percentage of subjects using each medication will be displayed together with the number and percentage of subjects using at least one prior medication within each medication group and subgroup. This will be repeated by surgery type.

Concomitant medication is defined as any medication taken on or after the start date of IMP, irrespective of start date. Any concomitant treatment (surgeries or procedures) will be coded as “All Other Therapeutic”. Concomitant medications/treatment categorized by ATC classification and preferred term will be summarized by treatment arm and overall for the SAF. The number and percentage of subjects using each medication will be displayed together with the number and percentage of subjects using at least one medication within each medication group and subgroup. This will be repeated by surgery type.

A medication can be both prior and concomitant if it was taken both prior and on or after the date of IMP.

In addition, prohibited medications will be tabulated.

All prior and concomitant medications will be listed separately for the SAF.

12.3.3 Medical and Surgical History, and Concomitant Disease

Medical and surgical history, and concomitant disease will be coded using the most updated version of the MedDRA® dictionary. Medical and surgical history until signature of informed consent, and concomitant disease will be summarized separately by MedDRA system organ class and preferred term in treatment arms and overall for the SAF. These will also be repeated by surgery type.

A listing of medical and surgical history, and concomitant disease will be created for the SAF.

12.3.4 Transfusion Products

The type of transfusion products includes autologous blood transfusion/cell salvage, allogenic platelet concentrates, allogeneic RBCs, allogeneic FFP, cryoprecipitate and other.

PPD

The number and percentage of subjects, using any transfusion products will be summarized by treatment and treatment arm for the SAF. This will be repeated by surgery type. The number and percentage of subjects will also be presented for each type of transfusion product. The volume (mL) of administration for each type of transfusion product will be summarized as well. This will also be repeated by surgery type. These summaries will also be performed separately for RBCs taken before start of first IMP (SAF) and for each type of transfusion product after start of first IMP (SAF). Transfusion products will be listed for the All Subjects Enrolled Set.

12.4 Demographic and Baseline Characteristics

Descriptive summaries of demographic and baseline characteristics will be presented by treatment, treatment group and overall, for FAS, mFAS, SAF and PPS. The following demographic characteristics will be summarized in the following order in the tables: sex, race, ethnicity, age, weight, height, body mass index, childbearing potential, and predictive blood loss. The summary will also be repeated for surgery type.

A listing will be created to show all the demographic and baseline characteristics for the All Subjects Enrolled Set.

A listing will be created to show results for pregnancy test of female subjects with childbearing potential for the SAF.

12.4.1 Surgery

The detailed information of surgery, including e.g., type of surgery (categorized by spinal and abdominal), predictive blood loss, duration of surgery, time until decision to treat subject, time to end of surgery after decision to treat, and overall vital signs monitoring during surgery will be listed for the All Subjects Enrolled Set. Number and percentage of subjects for each type of surgery, descriptive statistics for duration of surgery, time until decision to treat (relative to start of surgery), time to end of surgery (relative to time of decision to treat) and time of IMP exposure during surgery will be presented by treatment group and overall for FAS. The surgery summary, without type of surgery will be repeated for spinal and abdominal surgery.

12.5 Efficacy Analyses

12.5.1 Primary Efficacy Parameter

The primary efficacy parameter is the intra-operative blood loss after decision to treat the subject with IMP until the end of surgery as measured and calculated by amount of blood from the blood suction unit and amount of blood from swabs, surgical cloths and compresses.

The primary objective of this study is to demonstrate that BT524 is non-inferior that means not worse than FFP/cryoprecipitate with a non-inferiority margin of 150 mL in reducing intra-operative blood loss by intravenous (IV) administration in subjects with acquired hypofibrinogenemia undergoing elective major spinal or abdominal surgery. The primary analysis of this endpoint is a non-inferiority test in the PPS. The null hypothesis for the primary analysis is that the degree of inferiority of BT524 compared to standard treatment (FFP/cryoprecipitate) is greater than or equal to the non-inferiority margin. The alternative hypothesis is that the degree of inferiority of BT524 compared to FFP/cryoprecipitate is less than the non-inferiority margin.

H0: $\mu 1 - \mu 2 \geq \delta$
H1: $\mu 1 - \mu 2 < \delta$

where

$\mu 1$ = mean intra-operative blood loss after the decision to treat the subject with IMP in the BT524 treatment arm
$\mu 2$ = mean intra-operative blood loss after the decision to treat the subject with IMP in the FFP/cryoprecipitate treatment arm
δ = non-inferiority margin = 150 mL

The final analysis will be performed using a two-way analysis of variance (ANOVA) with the intra-operative blood loss after the decision to treat the subject with IMP until the end of surgery as the dependent variable

PPD

and the predictive blood loss (> 1,000 mL to ≤ 2,000 mL and > 2,000 mL) as a covariate. The least square means and treatment difference (BT524 versus FFP/cryoprecipitate) will be presented with the corresponding 2-sided 95% confidence intervals and p-value. Non-inferiority will be demonstrated if the upper confidence limit of the 95% confidence interval for the difference in the least square means is less than the non-inferiority margin (150 mL). Assumptions for the ANOVA model will be evaluated by diagnostic (e.g., Shapiro-Wilk test for normality) and graphical methods such as residual plots, including distribution of residuals, and Q-Q plots of residuals. The ANOVA assumptions are reviewed during the BDRM and in case the assumptions are not fulfilled, data transformation and alternative methods are discussed (see sections 12.5.1.3 and 12.5.1.4).

In addition, the following analyses will be presented:

- Sensitivity analysis of the same analysis repeated for the FAS and mFAS
- Sensitivity analysis of spinal surgery subjects with no tumors.

12.5.1.1 Assessment of Superiority

If the non-inferiority is demonstrated by the primary analysis, then superiority will be assessed in the FAS and mFAS and repeated for surgery type, sex and race. For superiority analysis, the null hypothesis is that the blood loss in BT524 treatment arm is greater than that in FFP/cryoprecipitate treatment arm. The alternative hypothesis is that the blood loss in BT524 treatment arm is less than that in FFP/cryoprecipitate treatment arm.

$H0: \mu 1 - \mu 2 \geq 0$
$H1: \mu 1 - \mu 2 < 0$
where μ1 and μ2 are the same as above.

The same ANOVA model as the non-inferiority analysis will be used. The least square means and difference of least square means will be presented together with the 95% confidence intervals and 2-sided p-value. The superiority of BT524 is demonstrated if the 2-sided p-value is less than 0.05.

12.5.1.2 Sub-Group Analyses: Predicted Blood Loss

In addition to the primary analysis, a subgroup analysis of the primary endpoint will also be performed in the mFAS. Subgroups are predictive blood loss, as well as spinal and abdominal surgery. These subgroups will be further splitted by sex and race.

Primary endpoint will be analyzed using a one-way ANOVA in each subgroup. The least square means and difference of least square means together with 95% confidence intervals and p-values in each subgroup will be presented.

The intra-operative blood loss after decision to treat with IMP will be summarized descriptively overall and for subgroups predictive blood loss and surgery (spinal, abdominal) and will be listed for the FAS. In addition, intra-operative blood loss after decision to treat with IMP will be summarized as continuous data as a box plot for both treatment arms for the mFAS and the PPS and subgroups predictive blood loss and surgery (spinal, abdominal) for the mFAS.

12.5.1.3 Data Transformation

In the case that the statistical assumptions for the ANOVA model are shown to be violated via the diagnostic tests then the primary efficacy parameter will be log-transformed. The ANOVA will then be re-run and checked via the diagnostic tests. If the ANOVA assumptions now hold this will be used. If they do not hold then the alternative method shall be used on the non-log-transformed data.

12.5.1.4 Alternative Method

The statistical assumptions for the ANOVA model will be tested in regards to the primary endpoint. Primarily the outcome of a Shapiro-Wilk test and graphical quantile-quantile (QQ) plots will used to assess if the

assumptions are significantly violated. If deemed necessary other test may be performed as well. The methods used, and the conclusions will be discussed in the clinical trial report (CTR).

In case there is a significant violation to the assumptions for the ANOVA model for the primary endpoint, a non-parametric alternative method will be implemented, proposed by van Elteren for stratified or blocked continuous response variables.

This alternative method will be used as a sensitivity analysis to the primary non-inferiority analysis for the PPS, FAS and mFAS. It will also be used as a sensitivity to the superiority analysis for the FAS and mFAS.

12.5.2 Multiplicity

The primary variable is only tested for superiority when the non-inferiority is demonstrated by the primary analysis, which will not lead to inflation of the Type-I error.

The statistical analyses on secondary variables are of exploratory nature. No adjustment for multiplicity is needed.

12.5.3 Secondary Efficacy Parameters

All analyses of the secondary efficacy parameters will be performed on the FAS overall and for spinal surgery and abdominal surgery.

12.5.3.1 Correction of Fibrinogen Level

The proportion (%) of subjects with a successful correction of fibrinogen level (by FIBTEM A10) 15 minutes after start of first IMP administration will be compared between the treatment arms using a Cochran-Mantel-Haenszel (CMH) approach stratified by predictive blood loss (> 1,000 mL to ≤ 2,000 mL and > 2,000 mL). The number and percentage of subjects with a successful correction of fibrinogen level 15 minutes after start of first IMP administration will be presented for both treatment arms with corresponding 95% confidence intervals. The estimated treatment effect (i.e., the difference in response rate between the treatment arms, BT524 – FFP/cryoprecipitate), corresponding 95% confidence interval, and 2-sided p-value for the difference will be presented.

The time to first successful correction of fibrinogen level (by FIBTEM A10) is assigned to the following categories: "Within 15 minutes after IMP start", "Greater than 15 and less than or equal to 90 minutes after IMP start", "Greater than 90 minutes after IMP start" and “Unsuccessful correction”. The 4 categories will be compared between the two treatment arms using a Chi-square test.

In an additional analysis the time to first successful correction of fibrinogen level (by FIBTEM A10) is classified relative to the end of surgery into "Until end of surgery", "Not within surgery" or “Unsuccessful correction”. The 3 categories will be compared between the two treatment arms using a Chi-square test.

For the above Chi-square tests, the number and percentage of subjects in each category will be presented for each treatment arm, together with an overall p-value for the difference between the two treatment arms.

The absolute values and change from baseline in fibrinogen levels by FIBTEM A10, together with the rate of fibrinogen restoring will be summarized with descriptive statistics over time by treatment arm. The absolute values at screening and change from screening at baseline will also be summarized for fibrinogen levels by FIBTEM A10.

In addition, the test results of MCF and Clauss assay will be summarized in the same way. The results of these three assays will be listed for the All Subjects Enrolled Set.

Mean with SD, median with interquartile ranges will be presented graphically over time for both treatment arms for FIBTEM A10, MCF and Clauss assay.

Pearson’s Correlation will be tabulated between the FIBTEM A10 and Clauss assay for baseline and prior first dose values in the Fibrinogen FIBTEM A10 summary tables.

Analysis will be repeated for spinal and abdominal surgery.

PPD

12.5.3.2 Consumption of Transfusion Products after IMP until End of Surgery

The type of transfusion products includes autologous blood transfusion/cell salvage, allogenic platelet concentrates, allogeneic RBCs, allogeneic FFP, cryoprecipitate and other. Consumption of transfusion products is measured by the total amount of transfusion product (volume in mL and units [bags]) infused after start of first IMP and until the end of surgery.

This secondary efficacy endpoint will be descriptively summarized for volume (mL) and units (bags) of transfusion product for each product type by treatment arm. The number and percentage of subjects, using any transfusion product, together with the number and percentage of subjects using each transfusion product type will also be summarized by treatment arm.

Analysis will be repeated for spinal and abdominal surgery.

Two box-whisker plots will be created to present the amount of each type of transfusion product after start of first IMP until end of surgery by product type. One plot will show volume in mL and the other will show units (bags). Analysis will be repeated for spinal and abdominal surgery.

All the consumption of transfusion products in this study will be listed for the All Subjects Enrolled Set.

Summaries of subjects will also be provided for subjects with total avoidance of transfusion products post IMP administration. This will be repeated by surgery type.

12.5.3.3 Amount of RBCs infused after IMP until End of Surgery

The amount (volume in mL and units [bags]) of RBCs (allogeneic and autologous) infused after start of first IMP administration until the end of surgery will be summarized by treatment arm.

An ANOVA analysis will be performed with the amount of RBCs required as the dependent variable and the predictive blood loss (> 1,000 mL to ≤ 2,000 mL and > 2,000 mL) as a covariate. The least square means and difference in least square means will be presented with the corresponding 95% confidence intervals and 2-sided p-value. This will be repeated by surgery type.

In addition, a subgroup analysis of the amount of RBCs will be performed for the FAS. Subgroups are predictive blood loss as well as spinal and abdominal surgery. The amount of RBCs will be analyzed using an ANOVA for each subgroup. The least square means and difference of least square means together with 95% confidence intervals and p-values for each subgroup will be presented.

12.5.3.4 Post-operative Blood Loss

The post-operative blood loss in the first 24 hours is the blood loss from end of surgery until 24 hours after end of surgery. This secondary efficacy endpoint will be descriptively summarized by treatment arm.

An ANOVA analysis will be performed with the post-operative blood loss in the first 24 hours as the dependent variable and the predictive blood loss (> 1,000 mL to ≤ 2,000 mL and > 2,000 mL) as a covariate. The least square means and difference (BT524 versus FFP/cryoprecipitate) in least square means will be presented with the corresponding 95% confidence intervals and 2-sided p-value. This will be repeated by surgery type.

A box-whisker plot for post-operative blood loss will be generated for both treatment arms.

In addition, a subgroup analysis of the post-operative blood loss in the first 24 hours will be performed for the FAS. Subgroups are spinal and abdominal surgery. The post-operative blood loss will be analyzed using an ANOVA for each subgroup. The least square means and difference of least square means together with 95% confidence intervals and p-values for each subgroup will be presented.

Post-operative blood loss will be listed for the FAS.

12.5.3.5 Proportion of Subjects with Rebleeds

The proportion of subjects with rebleeds will be compared between the treatment arms using a CMH approach stratified by predictive blood loss (> 1,000 mL to ≤ 2,000 mL and > 2,000 mL). The number and

PPD

percentage of subjects with a rebleed will be presented with corresponding 95% confidence intervals. The estimated treatment effect (i.e., the difference in rebleed rate between the treatment arms, BT524 – standard treatment (FFP/cryoprecipitate)), corresponding 95% confidence interval, and 2-sided p-value for the difference will be presented. The descriptive summary of the number of rebleeds will also be presented by treatment arm.

Analysis will be repeated for spinal and abdominal surgery.

Post-operative rebleeds will be listed for the FAS.

12.5.3.6 Length of Hospital Stay after Surgery

Subjects without a date of discharge (i.e., hospitalization ongoing) at Day 36 will be classified into > 36 days. The frequency of subjects whose length of hospital stay falls in the following categories are presented: 1-7, 8-14, 15-21, 22-28, 29-36, > 36 days.

The length of hospital stay after surgery will be summarized descriptively by treatment arm for those subjects, who had a day of discharge until closing visit. The details of surgery date, discharge or ongoing hospital stay, as well as length of hospital stay will be listed for the FAS.

Analysis will be repeated for spinal and abdominal surgery.

12.5.3.7 In-hospital Mortality

The number and percentages of subjects who died during their hospital stay will be presented with corresponding 95% confidence intervals of death rate by treatment arm using the Clopper-Pearson Method.

Analysis will be repeated for spinal and abdominal surgery.

The details of mortality during hospital stay will be listed for the FAS.

12.5.3.8 Overall Mortality

The number and percentages of subjects who died will be presented with corresponding 95% confidence intervals of death rate by treatment arm using the Clopper-Pearson Method.

The details of overall mortality will be listed for the FAS.

12.6 Safety Analyses

All safety analyses will be based on the SAF.

12.6.1 Adverse Events

Adverse events will be coded using the most updated version of the MedDRA® dictionary.

Adverse events occurring during or after the administration of study medication are TEAEs. If a partially missing AE start date is imputed, the imputed date will be used to assess if the AE is treatment emergent. If AE start date is completely missing and AE end date/time is after IMP start date/time or if it is completely missing, then it is a TEAE. All other AEs are regarded non-treatment emergent.

The search criteria for the adverse events of special interest are specified in the table below:

PPD

AESI	Search Criteria
Thrombosis or TEE	SMQ: Embolic and thrombotic events (narrow)
Relevant bleeding complication	All AESIs ticked as AESI, but not falling under categories “Thrombosis or TEE” or “Suspicion of transmission of infective agents (viral safety)”
Suspicion of transmission of infective agents (viral safety)	Preferred terms: “transmission of infectious agent via product” and “suspected transmission of infectious agent via product”

Adverse events related to Hypersensitivity/anaphylactic reactions/anaphylactic shock will be selected by using the following criteria: SMQ: Hypersensitivity, (narrow), Anaphylactic reaction (narrow), and Anaphylactic shock conditions (narrow).

Adverse events related to bleeding related ischaemic events will be selected by using the following criteria: SMQ: Ischaemic heart disease: Myocardial infarction (narrow), Other ischaemic heart disease (narrow); Ischaemic colitis (broad); Ischaemic central nervous system vascular conditions (narrow); Embolic and thrombotic events, arterial (narrow); Embolic and thrombotic events, venous (narrow); PT: Peripheral ischaemia; Ischaemia; Gastric ischaemia; Hepatic ischaemia; Ischaemic contracture of the left ventricle; Dry gangrene; Ischaemic gastritis; Ischaemic limb pain; Necrosis ischaemic; Spleen ischaemia; Uterine ischaemia.

If a serious adverse event (SAE) occurs in a subject after the period of observation, i.e., after the last study visit, and is considered by the investigator to be related to the study medication, this should still be recorded as a SAE. If the eCRF has been closed for the subject, the investigator should contact the sponsor to determine how to report the SAE.

The total number and percentage of subjects reporting at least one AE and the absolute count of AEs will be tabulated for each treatment, treatment arm and overall once for the overall trial population and separately by type of surgery (spinal and abdominal surgery), sex and race. The initial summary will also provide a breakdown of the following:

- Any AE
- Any non-severe AE
- Any TEAE
- Any NTEAE
- Any non-serious TEAE
- Any non-severe TEAE
- Any treatment-related TEAE
- Any severe AE
- Any severe TEAE
- Any severe treatment-related TEAE
- Any SAE
- Any serious TEAE
- Any serious treatment-related TEAE
- Any AESI
- Any treatment-emergent AESI
- Any treatment-emergent treatment-related AESI
- Any AE leading to discontinuation from study
- Any TEAE leading to discontinuation from study

PPD

- Any treatment-related TEAE leading to discontinuation from study
- Any AE with outcome of death
- Any TEAE with outcome of death
- Any treatment-related TEAE with outcome of death
- Any TEAE of special interest
    - Thrombosis or TEE
    - Suspicion of transmission of infective agents
    - Relevant bleeding complication
- Any TEAE of Hypersensitivity/ anaphylactic reactions/ anaphylactic shock
- Any TEAE of Bleeding related ischaemic events

The number of events, number and percentage of subjects reporting AEs will be tabulated (as well for causality and maximum severity) in the following way once for the overall population, and separately by type of surgery (spinal and abdominal surgery) and sex in the following:

- Treatment-emergent AEs by system organ class and preferred term
- Serious treatment-emergent AEs by system organ class and preferred term
- Causally related TEAE by system organ class, preferred term and maximum severity
- Not Causally related TEAE by system organ class, preferred term and maximum severity
- Causally related Serious TEAE by system organ class, preferred term and maximum severity
- Not Causally related Serious TEAE by system organ class, preferred term and maximum severity

The number of events, number and percentage of subjects reporting AEs will be tabulated (as well for causality and maximum severity) in the following way once for the overall population, and separately by type of surgery (spinal and abdominal surgery) in the following:

- Non-treatment emergent AEs by system organ class and preferred term (will not be tabulated for maximum severity or causality)
- Treatment-emergent AEs by system organ class and preferred term of Hypersensitivity/ anaphylactic reactions/ anaphylactic shock
- Treatment-emergent AEs by system organ class and preferred term of Bleeding Related Ischaemic Events
- Treatment-emergent AEs by system organ class and preferred term leading to study discontinuation (will not be tabulated for maximum severity, causality or separately by type of surgery)
- Treatment-emergent AEs by system organ class and preferred term leading to death (will not be tabulated for maximum severity or separately by type of surgery)
- Treatment-emergent AEs by system organ class and preferred term leading to hospitalization (will not be tabulated for maximum severity or causality)

The following AESI categories (only treatment-emergent adverse events) will be presented by system organ class and preferred term for the overall population, and separately by type of surgery and sex. They are repeated for maximum severity and causality for overall and surgery type only.

- Adverse events of special interest:
    - Thrombosis or TEE
    - Suspicion of transmission of infective agents (presented only for the overall population and for causality)

PPD

- Relevant bleeding complication

Listings will be produced for all AEs (including non-treatment emergent adverse events) reported in the eCRF for the All Subjects Enrolled Set.

In the listing, time to onset will be presented. Time to onset of the adverse event is defined as start date/time of adverse event – date/time of first study drug administration.

12.6.2 Deaths and Serious Adverse Events

Listings for SAEs and subjects with AEs with the outcome of death will also be provided for the SAF.

12.6.3 Laboratory Data

The following clinical laboratory variables will be summarized for the overall population, and separately by type of surgery (spinal and abdominal surgery):

**Hematology**	Red blood cells (RBC), white blood cells (WBC), platelet count, hemoglobin, hematocrit, neutrophils (absolute and %), lymphocytes (absolute and %), monocytes (absolute and %), eosinophils (absolute and %) and basophils (absolute and %)
**Biochemistry**	Aspartate aminotransferase, alanine aminotransferase, creatinine, creatinine clearance (Cockcroft and Gault), blood urea nitrogen (BUN), urea, gamma-glutamyltransferase (γ-GT), alkaline phosphatase (AP), total bilirubin, direct bilirubin, indirect bilirubin, potassium, sodium, calcium and chloride
**Urinalysis**	pH, blood, WBC, protein, glucose, ketone bodies, nitrite, bilirubin and urobilinogen
**Markers of Coagulation**	PT, INR, aPTT, TAT, F_{1+2}, D-dimer, PS, PC, AT III, TT and ETP(TGT)
**Coagulation factors**	FII, FV, FVII, FVIII, FIX, FX, FXI, FXIII and vWF

Note: Protein C (PC) is also referred to as Factor XIV. Direct bilirubin and indirect bilirubin are only assessed in case of an elevated bilirubin value and are thus not generally included in statistical summaries.

Quantitative laboratory parameters reported as “< x” or “ y” > will be imputed with x/2 and y, respectively, for inclusion in summary statistics. The reported value will be presented in listings.

Descriptive statistics for clinical laboratory values (in SI units) and changes from baseline at each scheduled assessment visit (as stated in Flowchart of study) with the exception of urinalysis parameters and direct/indirect bilirubin will be presented by treatment and treatment arm.

Laboratory results of all hematology, biochemistry, urinalysis parameters and PT/INR, aPTT will be categorized with respect to the laboratory specific reference ranges as normal/abnormal (i.e., high [H], low [L], where applicable). Abnormal values will be further classified with respect to clinical relevance by the investigator. The results and percentages will be summarized by visit and overall for each treatment and treatment arm.

Shifts from baseline to each post-baseline visit for all laboratory variables (except direct and indirect bilirubin) will be summarized by treatment and treatment arm using number and percentage of subjects.

In addition, a shift summary of hematology for number of subjects with change from normal or not clinical significant to clinical significant will be presented.

All the laboratory data will be listed for the SAF.

12.6.4 Vital Signs

Descriptive statistics for vital signs (systolic and diastolic blood pressure, pulse rate, respiratory rate and body temperature) and their changes from baseline at each scheduled study visit will be presented by treatment and treatment arm. This will be repeated by surgery type.

The overall vital signs monitoring results during surgery will be tabulated by number and percentage of subjects in each treatment, treatment arm and overall. This will be repeated by surgery type.

All vital signs will be listed for the SAF.

12.6.5 Physical Examinations

All the physical examination parameters will be listed for the SAF.

12.6.6 Viral Status

A shift table from screening to closing visit for virus serology parameters (hepatitis B, hepatitis C and HIV) will be summarized by treatment and treatment arm using number and percentage of subjects.

Listings for viral status will be created respectively for the SAF.

13.0 Validation

PPD goal is to ensure that each TFL delivery is submitted to the highest level of quality. Our quality control procedures will be documented separately in the study specific programming quality control plans.

14.0 References

PPD [redacted]

[redacted]

[redacted]

[redacted]

[redacted]

[redacted]

[redacted]

PPD

Appendix 1 Tables, Figures, Listings, and Supportive SAS Output Appendices

Clinical Study Report:

Please see the separate document: Biotest 995 TFL Specifications.

DSMB:

Please see the separate documents for the DSMB Charter and a List of DSMB TFL Specifications.

Blinded Sample Size Recalculation:

Please see the separate document: Biotest 995 TFL Specifications for Sample Size Recalculation.

Interim Analyses for Sample Size Assessment:

Please see the separate document: Biotest 995 TFL Specifications for Interim Analysis of Sample Size.

Document S4: Subject information sheet and Master informed consent form—(995 - Informed Consent Form _Master V5.0 Final-clean_ 12 Dec 2019_redacted

Subject Information Sheet and Informed Consent Form

**Sponsor:**	Biotest AG
**Protocol No:**	995
**Protocol Title:**	**A randomized, active-controlled multicenter, phase III study investigating efficacy and safety of intra-operative use of BT524 (human fibrinogen concentrate) in subjects undergoing major spinal or abdominal surgery (AdFIrst)**
**Investigator:**	PPD
**Address:**	PPD
	PPD
	PPD
**Phone:**	PPD
**Fax:**	PPD

1.0 General Information

You are being invited to participate in a research study. It is your decision if you wish to participate. Before you decide whether or not to take part in this study, we would like to explain why the research is being done and what it would involve for you. Your study doctor or member of the study staff will go through this Information Sheet with you and answer any questions you might have. Ask the study doctor or member of the study staff if there is anything you do not understand. You are free to talk to your family and friends about the study. Once you have a good understanding of the study, and if you agree to take part, you will be asked to sign the Consent Form. You will be given a copy to keep. You are free to withdraw at any time without giving a reason and this would not affect the standard of care you receive.

It is important to tell the study doctor everything regarding your health history otherwise you may harm yourself by participating in this study.

This study is being organized by the pharmaceutical company Biotest AG.

2.0 Purpose of Study

You have been asked to participate in this research study because you will undergo major spinal surgery and may experience major blood loss during the surgery.

The purpose of this research study is to find how well and how safe BT524 (human fibrinogen concentrate) is as a complementary treatment of severe blood loss in patients undergoing spinal surgery. Fibrinogen is one of the substances in the blood that helps with normal blood clotting.

BT524 (human fibrinogen concentrate) is an investigational drug. Investigational means that the drug is not currently approved by the regulatory authorities in your country for the treatment of severe blood loss during spinal surgery.

Another product that has been approved by the regulatory authorities in your country and is currently used to treat blood loss may also be administered to you. This product is called Fresh Frozen Plasma (FFP). FFP is the liquid part of blood that helps clotting and stops bleeding.

Approximately 200 adult subjects will participate in this research study in approximately 20 study centres in the EU and Switzerland.

Your participation in this study will last at least 36 days.

3.0 Description of the Procedures

In this study, the effectiveness of BT524 will be compared to the effectiveness of FFP in the treatment of severe blood loss for patients undergoing spinal surgery.

If you agree to participate in this study, you will be assigned to a group to receive either BT524 or FFP. To ensure the groups are comparable, each patient is assigned to a group by chance (randomly). The chances of you receiving BT524 are 50/50, i.e. like flipping a coin.

The study will be partially blinded which means that neither you nor your surgeon or surgical staff will know which treatment you have received (if needed for urgent medical reasons, your surgeon can be informed which treatment you received). The anaesthesiologist who administer the study drug will always know which treatment you received.

You will receive the study drug during surgery if your study doctor decides that you have experienced severe blood loss. In case treatment is needed during surgery you will receive either BT524 or FFP. The study drug will be given to you as an intravenous infusion – through a tube into a vein in your arm or hand.

4.0 Study Visits and Procedures

This section describes what will happen during the study.

Before any tests and exams can be initiated you are asked to sign and date this Informed Consent Form.

The following tests and procedures are performed during the study at the following time points:

a) Screening visit – prior to your spinal surgery to make sure it is safe for you to be in the study. This visit can happen anytime within 6 weeks before your surgery.

b) The day of your surgery (Day 1):
   - before your surgery (this can be done on Day 1 or 1 or 2 days before surgery)
   - during your surgery before the decision is made to treat you with BT524 or FFP for excessive blood loss (Day 1)
   - during your surgery after you have been given either BT524 or FFP (Day 1)
   - at the end of your surgery (Day 1)

c) Follow-up Visits during your hospitalization on Day 2, Day 3, Day 5, and Day 8

d) Closing Visit at least 5 weeks but not later than 10 weeks after your surgery

a) Screening visit

Before being eligible to receive BT524 or FFP as part of this research study you will undergo some tests and procedures. Having these tests and procedures will not guarantee that you will be able to receive BT524 or FFP. Your participation in the treatment part of the study (treatment with BT524 or FFP) will depend on the results of your laboratory tests, study guidelines, and the study doctor's judgment.

At the first visit, called the screening visit, the study doctor will ask some questions about you (including your date of birth, gender, and race), your general health, and your medical and surgical history. There will be a physical examination and assessment of your vital signs where your weight, height, heart rate, blood pressure, respiratory rate and body temperature will be recorded.

Blood (38 mL) and urine samples will be collected. These samples will be used to check your general health, if you are pregnant (if you are female and of child bearing potential) and to check how well your blood will clot.

The blood samples collected at screening visit will also be used to check if you are infected with human immunodeficiency virus (HIV) and hepatitis B and C (HBV and HCV). Depending on the local requirements, if your test is positive for HIV or Hepatitis B or C, these results will be reported to the Public Health Department. Additionally, it is possible that some insurance companies, employers, government agencies, or health care providers might also require you to report these tests results.

Some of the tests above may have been performed by your doctor as standard of care before you sign this informed consent form. If this test was done within 6 weeks before your first scheduled treatment with BT524 or FFP, your doctor may decide to not repeat this test and to use the result obtained from standard of care for the purpose of the study.Blood samples (5 mL) will be collected and stored in case you develop a viral reaction after you have been treated with BT524 or FFP, or for further analysis in case of future suspicion of a new virus not yet identified.

b) The day of your surgery (Day 1)

Before the surgery (Day 1, or 1 or 2 days before surgery), the study doctor will ask some questions about your medical and surgical history as well as your symptoms. There will be a physical examination and assessment of your vital signs where your weight, heart rate, blood pressure, respiratory rate and body temperature will be recorded.

Blood (44 mL) and urine samples will be collected. These samples will be used to assess your general health and to check how well your blood is clotting.

If you are female and of child bearing potential, prior to surgery you will have a urine pregnancy test.

During the surgery (Day1) your blood loss will be measured. Only in case your blood loss will be approximately 1 L and a treatment to control the bleeding is required, the study drug will be given to you. If you do not experience a major blood loss and your blood clotting is normal, a treatment with BT524 or FFP is not necessary. Your care during surgery will not be affected in any way.

During the surgery (Day1) and at the end of the surgery (Day1), the study doctor will monitor blood loss, vital signs including heart rate, blood pressure, respiratory rate and body temperature as well as medication you receive. After surgery you will be asked about your symptoms. Blood samples will be collected during (84 mL) and at the end of the surgery (38 mL). These samples will be used to assess your general health and to check how well your blood is clotting.

The following tests and procedures are only performed if either BT524 or FFP was given to you.

c) During your hospitalization on Day 2, Day 3, Day 5, and Day 8

There will be a physical examination and assessment of your vital signs where your heart rate, blood pressure, respiratory rate and body temperature will be recorded.

Blood (146 mL) and urine samples will be collected. These samples will be used to assess your general health and to check how well your blood is clotting.

You will be asked about your symptoms and the doctor will speak to you about how you feel.

d) Closing Visit at least 5 weeks but not later than 10 weeks after your surgery

There will be a physical examination and assessment of your vital signs where your heart rate, blood pressure, respiratory rate and body temperature will be recorded.

Blood (36 mL) and urine samples will be collected. These samples will be used to assess your general health and to check how well your blood is clotting.

Blood samples (5 mL) will be collected and stored in case you develop a viral reaction after you have been treated with BT524 or FFP or for further analysis in case of future suspicion of a new virus not yet identified. If the retention sample is not taken at the closing visit, your study doctor can schedule a follow-up visit to ensure that a sample for viral safety testing is available.

You will be asked about your symptoms and medication you have taken following your discharge from hospital and the doctor will speak to you about how you feel.

5.0 Subject Responsibilities

As a participant in this study, you have certain responsibilities to help ensure your safety. These responsibilities are listed below. It's your obligation to:

- Complete all required visits to the study centre;
- Report all side effects and medical problems to your study doctor or staff;
- Report if you (or your partner, if you are a man) have become pregnant; and
- Inform the study doctor or staff if you decide to no longer participate in the study. You may be asked to complete a close out visit.

6.0 Potential Benefits

We cannot promise the study will benefit you. The study drug may reduce the level of blood loss during surgery or may have no effect at all. The information we get from this study may help improve the treatment of people with excessive blood loss during major spinal surgery.

7.0 Potential Risks and/or Discomforts

In this study, you will receive either BT524 or FFP. There are benefits and risks associated with treatment in both groups, which your study doctor will discuss with you for both products BT524 and FFP.

You may have side effects from study drugs, or the procedures used in this study.

Side effects usually vary from person to person and can range from mild to very serious. If you have any side-effects as a result of taking part in this study, tell your study doctor right away, even if you do not think they may be due to BT524 or FFP.

Below you are informed about the potential risks (potential side effects) associated with BT524.

Potential General Risks

The study doctor will closely monitor your safety, and at every visit your study doctor and study staff will ask you about any side effects, you have experienced. If you have any problems, or any side effects during this study, you should let the study doctor know at once.

Known Potential Risks Associated with Fibrinogen Concentrate (Human)

The below side effects have been reported for other human fibrinogen concentrate products and therefore they can potentially occur in this study.

- **Allergic reaction**

Allergic reaction or anaphylactic type reactions such as generalised urticaria (hives over the whole body), rash, fever, chills, nausea, vomiting, abdominal or back pain, fall in blood pressure, dyspnoea (shortness of breath) is always possible. Serious allergic reactions that can be life-threatening may occur. If allergic or anaphylactic-type reactions occur, the injection/ infusion has to be stopped immediately. In case of a life-threatening allergic reaction such as difficulty breathing, low blood pressure, and/ or organ failure (anaphylactic shock), your doctor will start with standard medical treatment for shock to treat this reaction.

- **Increase in body temperature**

- **Blood Clot** (thrombosis/ thromboembolic events)

There is a risk of blood clot when patients are treated with human fibrinogen, particularly with high dose or repeated dosing. If you are given human fibrinogen, you will be closely observed for signs or symptoms of blood clots.

The blood clots may results in:

- Heart attack, the warning signs are sudden chest pain or shortness of breath.
- Stroke, the warning signs are sudden onset of muscle weakness, loss of sensation and/or balance, decreased alertness or difficulty in speaking.
- A serious condition called pulmonary embolism, the warning signs are chest pain, difficulty in breathing or coughing up blood.
- Clot in a vein (venous thrombosis), the warning signs are redness, feel warmth, pain, tenderness, or have a swelling of one or both legs.

For your safety please inform your study doctor, who will estimate the risk of having a thromboembolic event based on the individual medical history as mentioned below:

- If you have a history of coronary heart disease or myocardial infarction (heart attack), a liver disease,
- If you underwent or will undergo a surgery,
- If you are at risk of blood clots (thromboembolic events) or disseminated intravascular coagulation (a condition that is associated with uncontrolled clotting and bleeding in the body that can cause serious bleeding and organ damage),
- If you have family history of blood clot (thromboembolic events).

In case of over dosage, the risk of development of blood clots complications is enhanced. Pre-cautionary and close monitoring and appropriate measures will be put in place by your study doctor.

- **Transmission of infectious agents (Virus safety)**

BT524 is a fibrinogen concentrate made from human plasma (collected from blood). Products made from human plasma may potentially contain infectious agents, such as viruses like hepatitis or HIV (the AIDS virus), that can cause disease. The risk that BT524 will transmit an infectious (viral) agent has been reduced by screening plasma donors for prior exposure to certain viruses, by testing for the presence of current viral infections, and by inactivating and/or removing certain viruses during the manufacturing process. The measures taken are considered effective for enveloped viruses such as human immunodeficiency virus (HIV), hepatitis B virus and hepatitis C virus, and for the hepatitis A and parvovirus B19 viruses.

Despite these measures, such products may still potentially contain infectious agents, including those not yet known or identified. Thus, the risk of transmission of infectious agents cannot be totally eliminated.

Known Potential Risks Associated with Fresh Frozen Plasma (FFP)

The side effects for the FFP listed in this Consent Form are derived from the Product Information of a reference FFP product.

Hypersensitivity reactions to this product may rarely be observed and are:

- Usually mild type of allergic reactions consisting of localised or generalised skin rashes, redness, hives, itching, and increased sweating.
- More severe forms can be complicated with a drop in the blood pressure and with swelling of the face, the tongue, and difficulty swallowing.
- Very rarely, some patients may experience severe allergic reaction that might have a rapid onset and is characterised with complications like low blood pressure, increased heart rate, or irregular heartbeats, constriction of the airways and difficulty breathing, wheezing, coughing,

breathlessness, nausea, vomiting, diarrhoea, abdominal or back pain. Such severe reactions may progress to shock, including unconsciousness, collapse of the circulation system, failure of breathing, heart failure, and in very rare occasions even to death.

Other side effects connected to the use of this medicainal product may include the following symptoms (frequencies cannot be estimated from the available data, as these side effects have been mainly observed during post-approval use):

- Reduced sense of touch, dizziness, flushing, chills (shivering with or without fever), local swelling (oedema), fever.
- Furthermore, there may be abnormal symptoms in the lungs with lack of oxygen, anxiety, agitation, restlessness, reactions of the application site, low or high blood pressure, and sometimes very rarely, a generalized predisposition to bleeding.
- Negative effects can be caused by citrate contained in FFP. You may experience symptoms like fatigue, tingling feelings (paraesthesia), tremor and low calcium levels.

FFP may increase the risk of blood clots which may result in:

- Heart attack, the warning signs are sudden chest pain or shortness of breath.
- Stroke, the warning signs are sudden onset of muscle weakness, loss of sensation and/or balance, decreased alertness or difficulty in speaking.
- A serious condition called pulmonary embolism, the warning signs are chest pain, difficulty in breathing or coughing up blood.
- Clot in a vein (venous thrombosis), the warning signs are redness, feeling warmth, pain, tenderness, or have a swelling of one or both legs.

In all patients that are at risk for increased clotting of the blood, special caution will be exercised, and appropriate measures will be considered.

In rare cases, an incompatibility between antibodies (natural chemicals in the body that fight infections) in FFP and antigens (a substance that, when introduced into the body, stimulates the production of antibodies against it) in your blood can result in haemolytic transfusion reactions with destruction of your red blood cells leading to low red blood cell count. The symptoms may include chills; fever; a non-productive cough; difficulty in breathing; rash; and bleeding within the body.

Infusion of FFP may give rise to specific coagulation factor antibodies.

High dosages or infusion rates may induce increased blood volume; oedema (fluid accumulation) in the lungs and/or heart failure.

Depending on type and severity of adverse reactions, the infusion rate must be reduced, or the administration must be stopped. Appropriate action will be taken by your doctor.

Please inform your doctor if you have any type of immune deficiency concerning the secretory IgA antibodies, a severe problem with your liver and possible diagnosed deficiency in the synthesis of Protein S, manifest or hidden heart diseases and decompensation, swelling of the lungs, as well as a previous reaction to this type of product. In these instances, the FFP used in the study should be administered with special caution under these conditions

Transmission of infectious agents (Virus safety)

Products made from human plasma may potentially contain infectious agents, such as viruses like hepatitis or HIV (the AIDS virus), that can cause disease.

Certain measures are put in place to prevent infections being passed on to patients. These include careful selection of the blood and plasma donors to make sure those at risk of carrying infections are excluded, and the testing of each donation and pools of plasma for signs of virus/infections. Manufacturers of these products also include steps in the processing of the blood or plasma that can inactivate or remove the viruses. Despite these measures, when medicines prepared from human blood or plasma are administered, the possibility of passing on infection cannot be totally excluded. This also applies to any unknown or emerging viruses or other types of infections.

Unknown/Unexpected Risks and Discomforts

There are adverse events that are not known or happen rarely when patients receive these study drugs. You will be told of any new information that might cause you to change your mind about continuing to take part in this study.

Other Study Procedures

In addition to the potential side effects listed above, you may also experience some discomfort, bruising, and possibly infection as a result of taking blood samples.

8.0 Pregnancy/Birth Control (Female Participants)

The risks of taking BT524 by pregnant women or to an unborn baby are unknown. For this reason, a negative pregnancy test at screening visit is mandatory. You must not become pregnant during this study. If you are a woman of childbearing potential, you are obliged to use an effective form of birth control during this study and for at least one month after the last administration of study drug. Acceptable methods of birth control include consistent use of an approved oral contraceptive (birth control pill), an implantable contraceptive, an injectable contraceptive, a double-barrier method (diaphragm with spermicide, condom with spermicide), or abstinence. True abstinence is only considered an acceptable method of birth control, if it is in line with your usual lifestyle. Oral, implantable (e.g. vaginal delivery system), or injectable contraceptives are only considered effective if used properly and started at least 30 days prior to the screening visit. Some drugs (e.g., antibiotics) may interact with hormonal contraceptives, making them less effective. Please inform your study doctor of all other medications you are taking. If you suspect that you may have become pregnant during the study, you must contact your study doctor immediately. Your study doctor may want to follow the progress of your pregnancy until the baby is born. The effects of BT524 on a nursing infant are unknown; if you are breastfeeding, you cannot participate in the study.

9.0 Male Reproduction/Birth Control

There are no specific birth control measures required, however the usage of condoms are strongly advised for male participants during the study.

If you suspect your female partner may be pregnant, you must contact the study doctor immediately. Your study doctor may want to follow the progress of your partner's pregnancy until the baby is born.

10.0 Reimbursement/Cost for Participation

There are no anticipated costs for you while participating in the study. The study medication will be provided to you free of charge. You will not be charged for any procedure performed for the purpose of this study.

You will not be paid for your participation in the study, but you will be reimbursed for any reasonable expenses incurred by taking part, for example travel to and from the study site for your visits and/or parking at the hospital.

11.0 Alternative Treatments

If you do not wish to participate in this study, your treatment will be continued by your regular doctor and your care during surgery will not be affected in any way.

Your study doctor will explain the standard treatments available for people who have acquired fibrinogen deficiency (blood clotting problems) caused by major surgery with excessive blood loss. Alternative treatment options are the use of cryoprecipitate, fresh frozen plasma or fibrinogen concentrates that might be available in your country to treat bleeding. There are benefits and risks associated with this medication, which your doctor will discuss with you.

If the treatment with BT524 seems to be insufficient in controlling your bleeding events, the study doctor can use another available alternative treatment.

12.0 Confidentiality and Data Protection

To participate in this study, it is necessary that you read and sign the data protection information and consent form attached in **Appendix 1**.

13.0 Injury Compensation – Information about Insurance

Before participating you should consider if this will affect any insurance you have and seek advice if necessary.

In the event of an illness or injury that is determined to be directly related to the administration of study drug or the properly-performed study procedures, the Biotest AG agrees to pay all reasonable and necessary medical expenses to treat such illness or injury provided that you have followed the directions of the study doctor, and that you have not otherwise been reimbursed by your personal insurance, a government program, or other third party coverage for such medical expenses. No other compensation will be offered by Biotest AG or the Institution. Financial compensation for such things as lost wages, disability, or discomfort due to any research-related injury has not been made available. By signing this form, you are not waiving any legal right to seek additional compensation through the courts.

In accordance with country regulation, Biotest AG has issued an insurance policy.

Name and address of the insurance company: PPD
Policy number: PPD

13.1 Who to Contact to Ask Questions or Report a Possible Research Related Injury or Reaction

If you have any questions concerning your participation in this study, or if you feel you have experienced a research-related injury or a reaction to the study drug, you should contact:

Dr. PPD at PPD

13.2 Who to Contact To Report a Breach of Confidential Information

If you feel that there has been a breach of your confidential information, you should contact the principal investigator for this study:

Dr. PPD at PPD

13.3 Who to Contact to Ask Questions about Your Rights as a Research Subject

This research project has been reviewed by the PPD. This committee or board is a group of individuals from the community responsible for the review and approval of research proposed to be conducted. If you have questions about your rights as a research subject, you may contact:

The PPD at PPD.

14.0 Voluntary Participation and Termination of Participation

Your participation in this research study is voluntary. You can choose not to participate in this study either at the beginning or at any time during the study. Your choice will not have a negative impact on your present or future health care. There will be no disadvantage or loss of benefits to which you are otherwise entitled. To ensure your safety, you will be asked to undergo a final evaluation visit. If you wish to withdraw from the study, you should contact:

PPD or study personnel at PPD.

Your participation in this study may be discontinued without your consent by the investigator or the sponsoring company if you fail to follow the investigator's instructions. You may also be withdrawn from the study if, in the investigator's opinion, the study drug is ineffective, harmful, or has medically unacceptable side effects, or for other reasons at the discretion of the Sponsor or investigator. If you are withdrawn from the study, you may be asked to have the appropriate medical tests and follow-up to evaluate your health and safety.

15.0 **What if new information about the study drug becomes available**

Sometimes new information about the study drug is received. You will be told if any relevant new information becomes available. If the information is available prior to surgery, it may affect your willingness to carry on taking part in the study. If this happens after surgery your study doctor will be available to discuss the impact of new information with you. In either case your study doctor will contact you as soon as possible. If you decide not to carry on in the study, your study doctor will make arrangements for your care to continue. If you decide to continue in the study, you may be asked to sign a new consent form.

Also, if new information becomes available, your study doctor may stop your participation without your consent. If this happens the reasons will be explained, and arrangements made for your care to continue.

Consent Form

**Sponsor:**	Biotest AG
**Protocol No:**	995
**Protocol Title:**	A randomized, active-controlled multicenter, phase III study investigating efficacy and safety of intra-operative use of BT524 (human fibrinogen concentrate) in subjects undergoing major spinal or abdominal surgery (AdFIrst)
**Investigator:**	PPD
**Address:**	PPD
	PPD
	PPD
**Phone:**	PPD
**Fax:**	PPD

- I have read the description of the clinical research study and have had it explained to me in words and terms that I understand. I understand that my participation is voluntary. I know enough about the purpose, methods, risks, and benefits of the research study.
- I voluntarily agree to participate in this study.
- The study doctor will inform me of any new findings developed during the course of this study, which may affect my willingness to continue participation.
- I authorize the release of my study-related medical records to Biotest AG, the regulatory authorities, and the Ethics Committee/IRB.
- I understand that I will be provided a copy of this signed consent.
- I understand that I am free to withdraw my consent and to stop my participation in this study at any time.
- By signing this consent form I understand that I have not waived any of the legal rights as a participant in a research study.

Subject
(or legally authorized representative as applicable)

Subject Printed Name
(or legally authorized representative) Signature Date

Person Obtaining Consent

Printed Name & Title	Signature	Date

Witness (if applicable)

Witness Printed Name	Signature	Date

Appendix 1:
Data protection information and consent form

In connection with the conduct of the research study, certain personal data will be collected and processed. This document aims at informing you on how the collection and processing of your personal data is handled in connection with the research study.

Please read this data protection information carefully. To confirm that you have read and understood its content and that you agree with the processing of your personal data as described herein, please provide your signature at the end of the document.

1.0 Who is responsible for the data processing?

As the sponsor, Biotest AG ("Sponsor") determines the purposes and means of the processing of your personal data in the context of the research study together with study center and/or the study doctor. Therefore, both the Sponsor and the study center/study doctor are considered data controller under applicable data protection laws.

Contact details of the Sponsor:

Biotest AG

PPD

PPD

PPD

PPD

Contact details of [PPD]:

[PPD]

2.0 What types of personal data are processed?

In connection with your participation in the research study, the following personal data, including sensitive personal data, will be processed: your name/initials, sex, year of birth, contact details, information needed for reimbursement purposes, body weight and height, racial and ethnic origin, health data, including medical records and information on how you respond to the treatment, as necessary for the purpose of the research study, including blood and urine samples.

3.0 How and for what purpose will my personal data be used?

Your personal data will be collected by [PPD] and will be recorded on paper and/or on electronic data storage devices in/at [PPD] to run the research study and to monitor your safety as a study participant. In addition, some of your health data may

be obtained from other treating physicians, provided you have released them from their obligation to maintain medical confidentiality.

The personal data collected will be processed in a manner that ensures appropriate security and confidentiality of your data. The Sponsor and its representatives (e.g. Clinical Research Organisation - CRO), as well as the study center and the study doctor will take all reasonable steps to protect your privacy as is required by applicable laws and regulations. These include, e.g., measures to prevent unauthorized access to or use of your personal data and the equipment used for the processing, e.g. by limiting access to the rooms and equipment where your personal data is stored and by pseudonymizing your personal data. Specifically, for the purpose of this research study, your name will be replaced by a code (study patient number) at the beginning of the trial, in order to rule out that you can be directly identified or to make your identification significantly harder. The list with patient names and their respective codes will be kept in a secure place, separate from the research study documentation. Only your study doctor and the study center staff/persons explicitly authorized by the study doctor have access to the list and can link your study patient number to your name in case of emergency in/at [PPD]. The analysis and usage of the data obtained during the research study by the study doctor and the study center staff takes place exclusively in pseudonymized form.

Your pseudonymized personal data may be disclosed to and processed under supervision of PPD , in accordance with applicable laws and the terms of this consent form. PPD and its authorized personnel have signed a non-disclosure agreement or are legally obliged to maintain secrecy. In addition, your personal data, including sensitive personal data, may be:

a) kept available in/at [PPD] and disclosed to competent supervisory authorities for inspection or monitors appointed by the Sponsor to verify the proper conduct of the research study;

b) transferred, in pseudonymized form, to the Sponsor or to an agency commissioned by the latter for scientific evaluation,

c) transferred, in pseudonymized form, to the Sponsor and the competent authority for the marketing authorization if an application for a marketing authorization is filed;

d) transferred, in pseudonymized form, to the Sponsor and the competent authority and subsequently by the latter to the European database in the event of adverse events in connection with the investigational medicinal product,

e) transferred to the responsible health authority in case of a positive test result for hepatitis B and/or C, as far as the test result indicates an acute infection and to the Robert-Koch-Institut Berlin in case of a positive test result for HIV.

The blood and urine samples collected from you during the research study will be pseudonymized and shipped to, temporarily stored and tested by PPD . The samples will only be used for research purposes and no analyses except those specified in the Patient Information for the research study will be performed without your prior consent and the approval of the competent Ethics Committee. Your identity is kept confidential as the laboratory will only receive your study patient number. All data gained through the analysis of the samples will be transferred in pseudonymized form to the Sponsor or to a site commissioned by the Sponsor for the purposes of further analysis within scope of the research study. Following the completion of the research study all samples will be destroyed except for some of your blood, which will be frozen and stored in case you develop a viral reaction after you have been treated with BT524, or for further analysis in case of future suspicion of a new virus not yet identified. The frozen blood samples will be kept up to six months following the completion of the research study, or longer if required by applicable laws. The Sponsor will be responsible for storage and record

keeping after completion of the research study. Subsequent to the six months period, the samples will be destroyed.

Neither the Sponsor, nor its CRO or any other company supporting the Sponsor in conducting the research study will be able to identify you directly. If at all, they will receive pseudonymized personal data (e.g. year of birth, gender, race and health data relevant to the research study). This, however, does not apply to monitors, who are appointed by the Sponsor to ensure the appropriate implementation of the research study and that your rights and well-being is protected. They will verify that you have provided informed consent prior to participating in the research study and that the source documents and other trial records are accurate, complete, kept up-to-date, and maintained. For that purpose, these monitors require direct access to your personal data, including sensitive personal data. Further, your personal data may be disclosed in response to lawful requests by public authorities, including those to meet national security or law enforcement requirements. For this purpose, you have to release the study doctor from the obligation to maintain medical confidentiality. All other individuals or companies acting on behalf of the Sponsor for the purpose of conducting the research study have been sworn to confidentiality.

Your personal data will be processed for the purposes of analyzing and reporting the results of this research study; to develop future study protocols; for product performance monitoring and scientific research investigating new treatments, interventions and management procedures so that patient outcomes are continually improved; and for ensuring compliance with medical, ethical and medical device laws and regulations.

If the Sponsor or the study doctor makes public any study results your identity will remain confidential, as the study results will be published in a form that does not allow your re-identification.

With your permission, the study doctor will tell your family doctor about your participation in this study.

4.0 Will my personal data be transferred to a third party?

Qualified representatives of the Study Sponsor and its worldwide affiliates; and/or PPD and its worldwide affiliates; and/or national and foreign regulatory authorities (including the Paul-Ehrlich-Institute (PEI) and /or the Food and Drug Administration (FDA); and/or independent auditors may look at your medical notes (including un-coded personal data), to check the information collected in this study, to check how the study was conducted and to monitor participant's safety. Further, PPD may be required to disclose your personal data in response to lawful requests by public authorities, including those to meet national security or law enforcement requirements.

The disclosure of your personal data to the above-mentioned third parties, may include transferring your (pseudonymized) personal data to countries other than where you are based and outside the European Union (“EU”) / the European Economic Area (“EEA”), such as the USA. The laws protecting personal data in third countries may not be not as strict as those applicable in Germany. However, the Sponsor will ensure that your personal data is transferred in compliance the provisions of the General Data Protection Regulation (GDPR). You may contact the study doctor to get more information about the safeguards used to protect your personal data transferred to third country. Some ways in which your personal data is kept safe includes having study sites put the appropriate arrangements for the security of your personal data, removing some direct identifiers of your personal data or key-coding it so that it is not identifiable and collecting only the personal data needed. You may also ask the study doctor for a copy of those safeguards.

5.0 On which legal basis is the personal data processed?

The processing of your personal data, including any sensitive personal data, is based on Article 6(1) lit. a) and Article 9(2) lit a) of the GDPR in connection with [insert applicable national law].

6.0 For how long will my personal data be stored?

Your personal data will be kept as long as necessary for the purpose of conducting the research study, unless longer periods are provided by applicable laws. The code to link your study patient number to your name will be deleted by the study doctor as soon as the purpose of the research study allows for it. However, applicable laws may provide for a longer retention period of the personal data. In that regard, your personal data may be stored for at least

- 10 years following the completion or discontinuation of the research study, or
- 2 years following the investigational medicinal product has received its last approval for a marketing authorization, or
- 2 years after the development of the present investigational medicinal product has been stopped,

  depending on whichever period is longer.

Absent other statutory or contractual retention periods, your personal data will be deleted afterwards.

7.0 Do I have to agree to the processing of my personal data?

No, your consent to the processing of your personal data is voluntary. However, you will not be able to participate in the research study without giving the consent for the processing of the above-mentioned personal data.

8.0 Do I have the right to withdraw my consent to the processing of personal data?

No, your consent to the processing of your personal data is irrevocable pursuant [insert applicable national law]. However, you are free to revoke your consent to participate in the research study at any time pursuant to [insert applicable national law]

9.0 What happens with my personal data if I revoke my declaration of consent to participate in the research study?

In case you withdraw your consent to participate in the research study, the Sponsor and its representatives (e.g., CRO), as well as the study center and the study doctor will determine without undue delay which of your personal data collected and stored may still be necessary to:

a) determine effects of the investigational medicinal product,

b) ensure that those of your interests which are worthy of special protection are not prejudiced,

c) satisfy the obligation to provide complete marketing authorization documents.

Personal data that is no longer necessary for the above-mentioned purposes will be deleted immediately or anonymized (e.g., by destroying the key code linking your name with your study patient number).

PPD

10.0 Which rights do I have in connection with my personal data?

You have the right to gain access to your personal data stored by the Sponsor, the study center or the study doctor (Article 15 of the GDPR). You also have the right to request the rectification of inaccurate personal data without undue delay according to Article 16 of the GDPR. Under certain conditions, you can also request deletion of your personal data without undue delay (see Article 17 of the GDPR) or restricted processing of your personal data (see Article 18 of the GDPR). You may also have the right to receive the personal data concerning your person, in a structured, commonly used and machine-readable format and to transmit those data to another controller without hindrance (Article 20 GDPR).

If you believe that the processing of your personal data violates the provisions of the GDPR, you have the right to lodge a complaint with the competent data protection supervisory authority in particular in the Member State of your habitual residence, place of work or place of the alleged infringement. Further information regarding competent supervisory authorities can be found on the website of the Federal Commissioner for Data Protection and Freedom of Information: www.bfdi.bund.de.

11.0 Who can I contact if I have any questions?

You can contact Biotest's data protection officer using the following contact details:

PPD

You can also contact the study center's data protection officer using the following contact details:

[PPD
]

Finally, a description of this research study will be available on http://www.ClinicalTrials.gov as required by U.S. Law and on the European Clinical Trials database at https://www.clinicaltrialsregister.eu as required by European Law. This website will not include information that can identify you. At most, this website will include a summary of the results. You can search this website at any time.

Consent

I have read and understand the above data protection information concerning the processing of my personal data in connection with the conduct of the research study 995 (EudraCT number: 2017-001163-20).

By signing this consent form, I agree that:

(1) My personal data, including sensitive personal, is collected, processed and stored for the purposes of the research study as described above.

(2) My personal data, including sensitive personal data, can be transferred to and shared with third parties both within and outside of the EEA, including countries that may not have the same level of data protection as [insert country name], as described in the data protection information above.

(3) My health data may also be obtained from other treating physicians, in particular, whom I hereby release from the physician-patient confidentiality obligation.

____
Print name of participant

____________________________________ ___________________________
Signature of participant Date (day, month, year)

Furthermore, I agree that my family doctor

____
Print name of family doctor

will be informed of my participation in the researchResearch study.
(If this is not wanted, please leave in blank.)

____
Print name of participant

____________________________________ ___________________________
Signature of participant Date (day, month, year)
