# Supplementary material for: Efficacy and safety of human fibrinogen concentrate (BT524) in patients with major haemorrhage undergoing major orthopaedic or abdominal surgery (AdFIrst): a randomised, active-controlled, multicentre, partially blinded, phase 3 non-inferiority trial
Source: eClinicalMedicine. 2025 Jun 7;85:103264. doi: 10.1016/j.eclinm.2025.103264 (PMC12308311; doi:10.1016/j.eclinm.2025.103264)
Supplement: Supplementary Material 2 [file mmc2.pdf]

Supplementary Material 2

Efficacy and safety of human fibrinogen concentrate (BT524) in patients with major haemorrhage undergoing major orthopaedic or abdominal surgery (AdFIrst): a randomised, active-controlled, multicentre, partially blinded, phase 3 non-inferiority trial.

Prof Niels Rahe-Meyer, MD, Ashok Roy, MD, PD Dr Hans-Heinrich Trouillier, MD, Dr Sonja Schimo, Dr Judith Wessels-Kranz, Salomon Abraha, Dr Alexander Staus, Dr Ümniye Balaban, Dr Thomas Häder, Dr Jörg Schüttrumpf, MD, Dr Silke Aigner, Heike Böhm

CONTENTS

	Page
**Supplementary Methods**	
Statistical Analysis	2
**Supplementary Results**	
Repeated doses of trial drug and FIBTEM A10 results	3
**Supplementary Figures**	
Figure S1: Overview of trial design and trial drug administration for patients undergoing spinal surgery	4
Figure S2: Overview of trial design and trial drug administration for patients undergoing abdominal surgery	5
Figure S3: Functional fibrinogen in patients throughout surgery (modified full analysis set)	6
Figure S4: Thromboembolic events in all patients (safety set)	8
**Supplementary Tables**	
Table S1: Surgical procedures and characteristics of patients in the trial (full analysis set)	9
Table S2: Correction of functional fibrinogen level in patients by three different analysis methods (modified full analysis set)	10
Table S3: Consumption of transfusion products after start of first trial drug administration until end of surgery (modified full analysis set)	11
Table S4: Length of hospital stay after surgery (modified full analysis set)	11
Table S5: Summary of adverse events and other outcomes (safety set)	12
Table S6: Summary of adverse events with a preferred term frequency of ≥5% in the overall trial population (safety set)	13

Supplementary Methods

Statistical Analysis

A two-way analysis of variance (ANOVA) was performed with the intraoperative blood loss after decision to treat until the end of surgery as the dependent variable, and treatment group (FC vs FFP/Cryo) and predictive blood loss (>1 L to =2 L and >2 L) as the two factors, to evaluate the primary endpoint. The primary endpoint was not normally distributed, consequently a pre-specified van Elteren test was performed as a sensitivity analysis. The efficacy analysis was applied for both non-inferiority and superiority analyses using a hierarchical test procedure. A 2-sided p value of <0·05 was considered statistically significant.

Sensitivity analyses were conducted for the subgroups of predictive blood loss and surgery type.

The primary efficacy analysis tested non-inferiority in the per-protocol set (PPS), while all other efficacy endpoints were analysed in the modified full analysis set (mFAS). Safety endpoints were analysed in the safety set. Post-hoc analyses were not pre-specified in the trial's statistical analysis plan and were performed for hypothesis-generating purposes. The results should be interpreted as exploratory as no formal adjustments for type 1 error for multiple comparisons were made.

Post-hoc analyses were conducted to assess group differences. For continuous variables, p values were calculated using the Wilcoxon rank-sum test. Confidence intervals (CIs) for the differences in medians between groups were estimated using a stratified bootstrapping method (1,000 resamples), with 95% CIs based on the 2·5th and 97·5th percentiles of the bootstrap distribution, accounting for stratification factors. For categorical outcomes, p values were calculated using the chi-square or Fisher's exact test, where appropriate. The 95% CIs for the differences in proportions were calculated using the standard error of the risk difference (RD), based on the binomial distribution. For the safety outcome, thromboembolic events (TEEs), a risk ratio (RR) with 95% CIs was calculated. The proportion of TEEs in each group was used to compute the RR, and the Wald log-transformation method was employed to obtain the confidence interval.

Supplementary Results

Repeated doses of trial drug and FIBTEM A10 results

The repeated intraoperative administration of trial drug was dependent on the patient's clinical condition and was triggered by FIBTEM A10 results of less than 12 mm.

In patients that received second or third doses of trial drug, the Surgery day 1 (predose) mean (SD) FIBTEM A10 measurement was approximately 60% lower than the baseline FIBTEM A10 measurement in the FC group (measurements available for 12 patients) and the FFP/Cryo group (measurements available for 15 patients). Overall, the mean FIBTEM A10 Surgery day 1 predose (i.e., prior to the second dose) value was similar in the FC group (7.8 mm [measurements available for 12 patients]) and the FFP/Cryo group (8.3 mm [measurements available for 15 patients]).

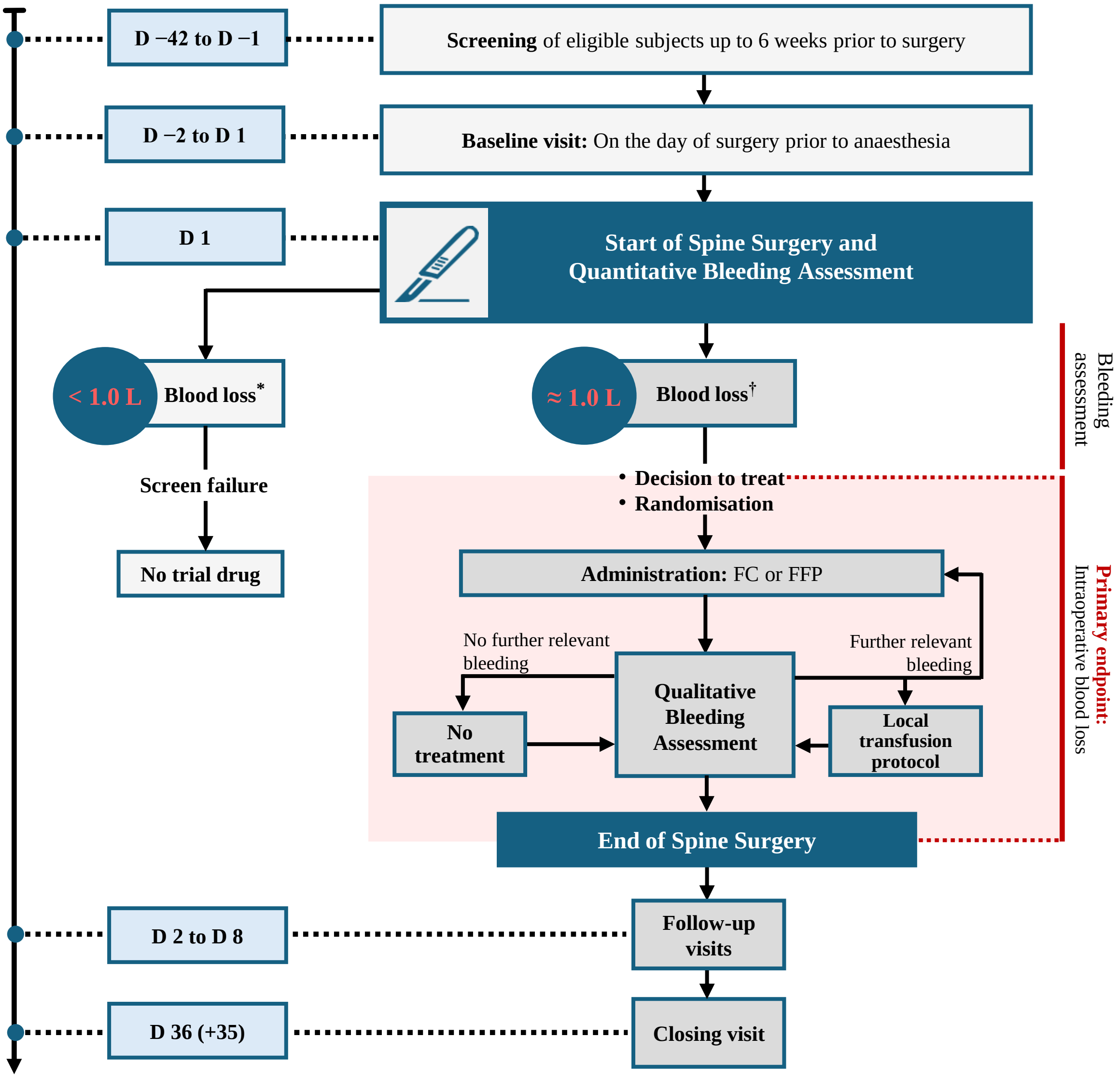

***Figure S*1. Overview of trial design and trial drug administration for patients undergoing spinal surgery**
D=Day. FFP=fresh frozen plasma. FC=human fibrinogen concentrate. *A patient was considered a screen failure if the intraoperative blood loss was <1.0 L and haemostatic treatment was not required. †The decision to treat was made if blood loss was ≈1.0 L or greater and haemostatic treatment was required.

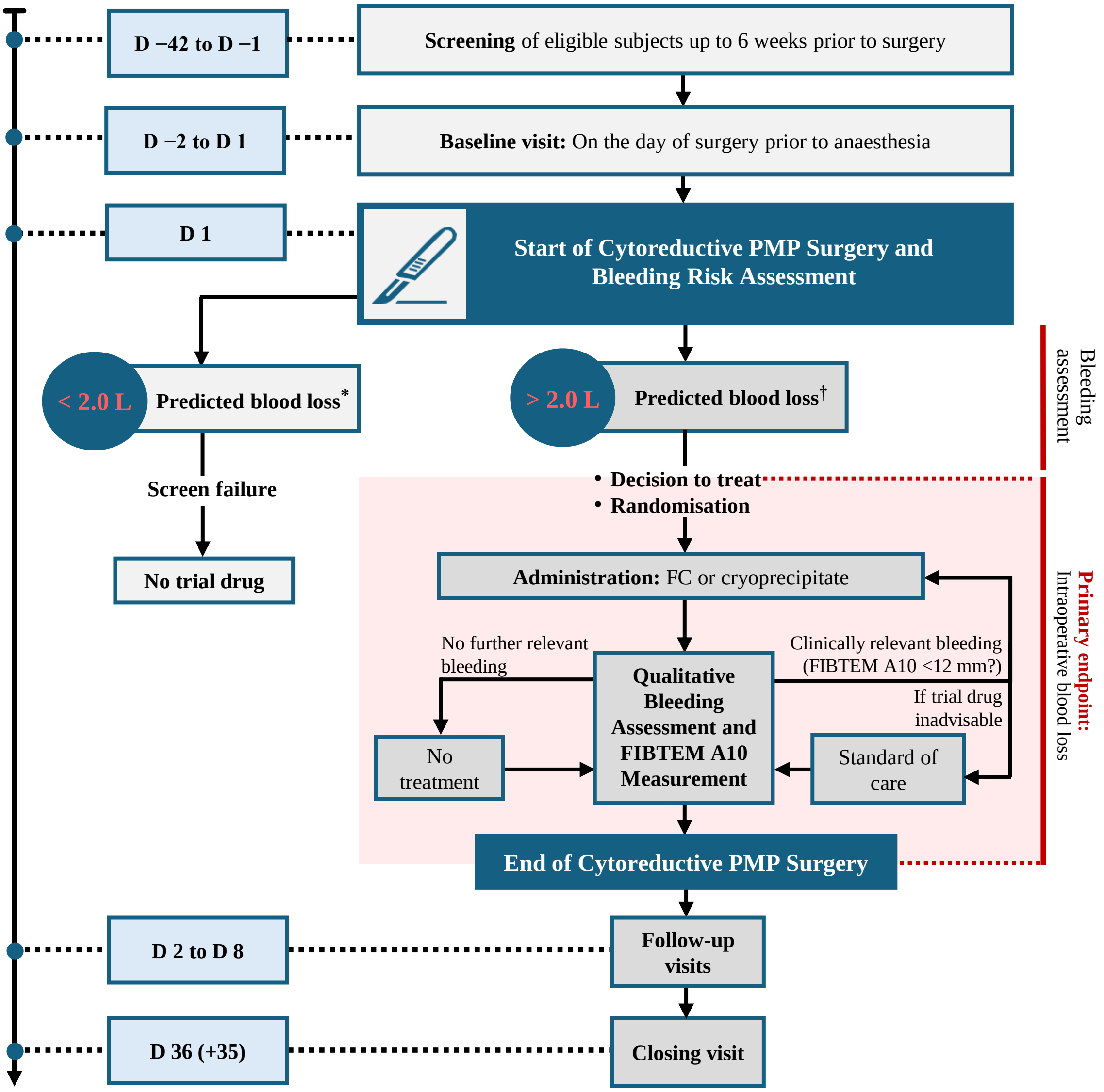

***Figure S*2. Overview of trial design and trial drug administration for patients undergoing abdominal surgery**
D=Day. FIBTEM A10=amplitude after 10 minutes as measured by rotational thromboelastometry. FC=human fibrinogen concentrate. PMP=pseudomyxoma peritonei. *A patient was considered a screen failure if the predicted blood loss at approximately 60 minutes after the start of surgery was <2.0 L and haemostatic treatment was not required. †The decision to treat was made if the predicted blood loss at approximately 60 minutes after start of surgery was >2.0 L and haemostatic treatment was required.

(A)

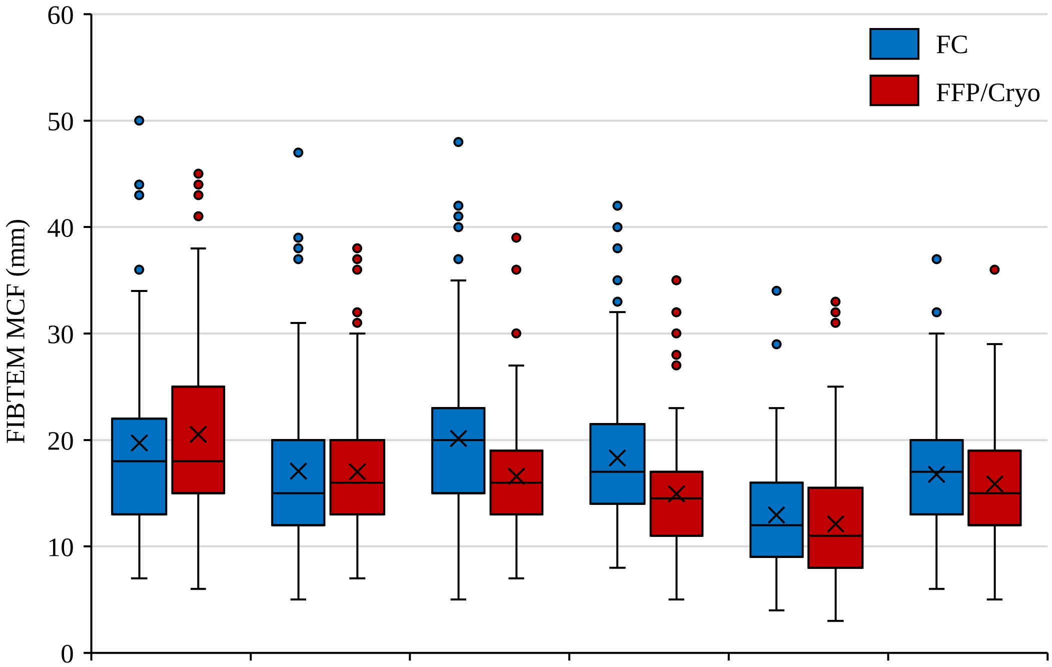

FIBTEM MCF [mm] (number of patients)	Baseline (before surgery)	Prior first dose of trial drug	15 minutes after first trial drug infusion	90 minutes after first trial drug infusion	End of surgery	Follow-up
FC	18 (106)	15 (107)	20 (103)	17 (97)	12 (102)	17 (106)
FFP/Cryo	18 (103)	16 (104)	16 (102)	14·5 (96)	11 (97)	15 (100)
	Day 1					Day 2

(B)

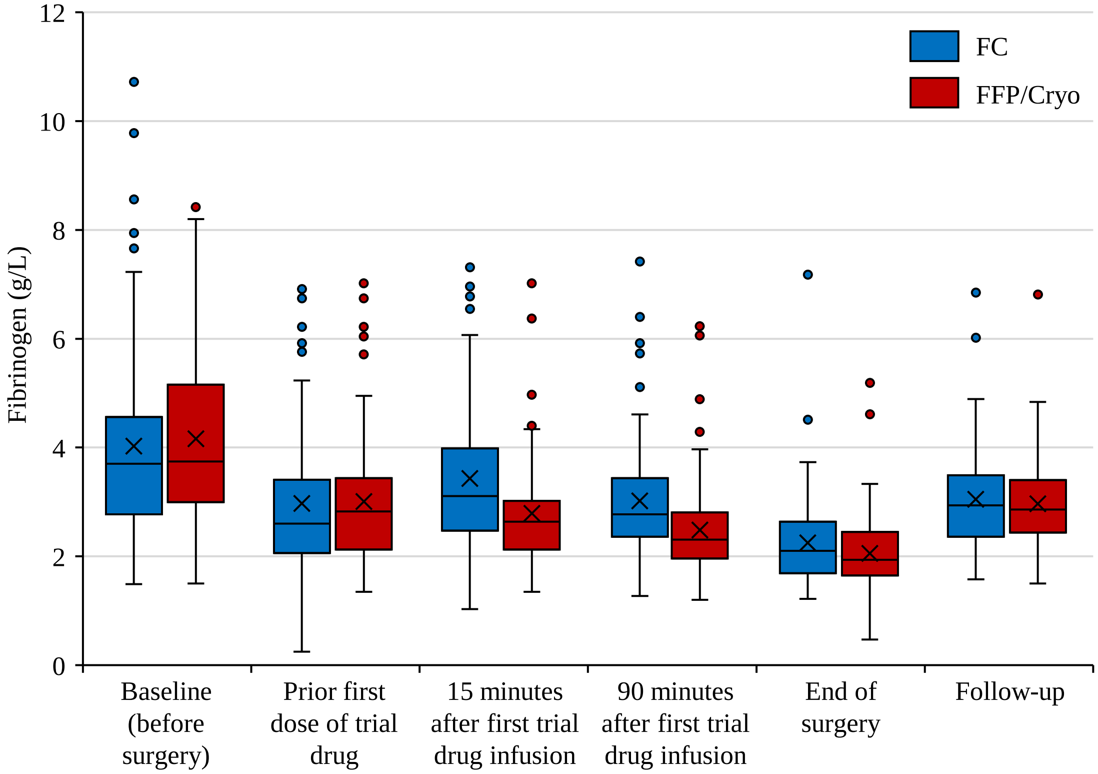

Fibrinogen [g/L] (number of patients)	Baseline (before surgery)	Prior first dose of trial drug	15 minutes after first trial drug infusion	90 minutes after first trial drug infusion	End of surgery	Follow-up
FC	3·7 (106)	2·6 (107)	3·1 (106)	2·8 (99)	2·1 (103)	2·9 (107)
FFP/Cryo	3·7 (104)	2·8 (104)	2·6 (104)	2·3 (96)	1·9 (98)	2·9 (104)
	Day 1					Day 2

***Figure S3*: Functional fibrinogen in patients throughout surgery (modified full analysis set)**
Functional fibrinogen in patients from baseline (Day 1, before surgery) to follow-up (Day 2). (A) Box-and-whisker plot depicting MCF as a measure of the functional fibrinogen level in patients as determined by ROTEM; (B) Box-and-whisker plot depicting fibrinogen concentration in patients as determined by the Clauss assay. End of surgery may have been before the 90-minute time point. Boxes represent the median, lower and upper quartiles (25th and 75th percentile, respectively), with whiskers indicating the minimum and maximum values. The whisker length is restricted to 1.5 times the IQR from the median; data points beyond this range are represented as individual dots. The mean value is indicated by a cross. Cryo=cryoprecipitate. FC=human fibrinogen concentrate. FIBTEM MCF=maximum clot firmness as measured by ROTEM. FFP=fresh frozen plasma. MCF=maximum clot firmness. ROTEM=rotational thromboelastometry.

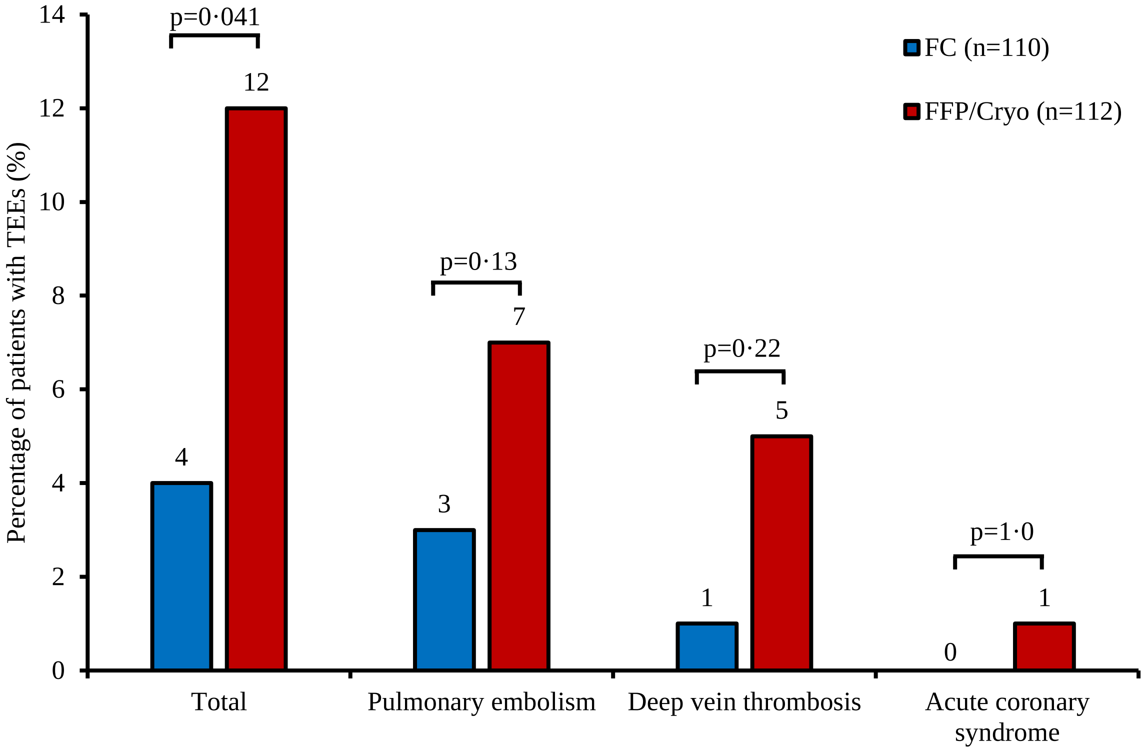

Figure S4: **Thromboembolic events in all patients (safety set)**
Only treatment-emergent adverse events are included. Cryo=cryoprecipitate. FC=human fibrinogen concentrate. FFP=fresh frozen plasma. TEE=thromboembolic event.

	FC (n=110)	FFP/Cryo (n=112)	Percentage difference (95% CI)	p value
Surgery type				
Abdominal surgery	48 (44)	50 (45)	-1 (-14 to 12)	0·88
Pseudomyxoma peritonei	48 (44)	50 (45)	-1 (-14 to 12)	0·88
Spinal surgery	62 (56)	62 (55)	1 (-12 to 14)	0.88
Surgery on spinal meninges and spinal cord	0	1 (1)	-1 (-3 to 1)	1·0
Spondylodesis (spinal fusion)	21 (19)	19 (17)	2 (-8 to 12)	0·68
Vertebral body replacement and complex spine reconstruction	3 (3)	3 (3)	0·1 (-4 to 4)	1·0
Release and scoliosis deformity correction	13 (12)	15 (13)	-2 (-10 to 7)	0·72
Complex 360° – reconstruction with fusion, ventrodorsal procedure	2 (2)	4 (4)	-2 (-6 to 3)	0·68
Complex 360° – reconstruction with fusion, ventrodorsal procedure after tumour resection	6 (6)	2 (2)	4 (-1 to 9)	0·17
Bony decompression of the spinal canal, ≥ 4 vertebral segments	12 (11)	10 (9)	2 (-6 to 10)	0·62
Re-operation	0	1 (1)	-1 (-3 to 1)	1·0
Other	5 (5)	7 (6)	-2 (-8 to 4)	0·57
Duration of surgery (hours)	6·9 (2·32)	6·9 (2·34)	0·0 (-0·6 to 0.6)	0·98
Time until decision to treat (hours)	1·2 (1·0–3·1)	1·3 (1·0–2·9)	-0·1 (-0·7 to 0·8)	0·97
Time to end of surgery after decision to treat (hours)	4·7 (2·3–7·1)	4·9 (2·6–6·3)	-0·2 (-1·6 to 1·6)	0·86

Data are mean (SD), median (IQR) or n (%). CI=confidence interval. Cryo=cryoprecipitate. FC=human fibrinogen concentrate. FFP=fresh frozen plasma. IQR=interquartile range. n=number of patients in the treatment group. SD=standard deviation. Differences presented are for FC – FFP/Cryo; a negative value indicates that the surgery type was done in fewer patients in the FC group or the time for the FC group was shorter.

***Table S1*. Surgical procedures and characteristics of patients in the trial (full analysis set)**

	FIBTEM A10		FIBTEM MCF		FIBRINOGEN [g/L][c]	
	**FC**	**FFP/Cryo**	**FC**	**FFP/Cryo**	**FC**	**FFP/Cryo**
Patients with successful correction of fibrinogen level 15 minutes after start of first trial drug administration[a]	59 (57)	19 (19)	58 (56)	15 (15)	29 (27)	6 (6)
95% CI of response rate	(47–66)	(12–28)	(46–66)	(9–23)	(19–37)	(2–12)
Difference in response rate (95% CI)	38 (26–50)		42 (30–53)		22 (12–31)	
p value	<0·0001		<0·0001		<0·0001	
Patients with first successful correction of fibrinogen level intraoperatively[b]						
≤15 minutes after trial drug start	59 (55)	19 (18)	58 (54)	15 (14)	29 (27)	6 (6)
>15 minutes or ≤90 minutes after trial drug start	17 (16)	11 (11)	18 (17)	14 (13)	10 (9)	2 (2)
>90 minutes after trial drug start	11 (10)	16 (15)	13 (12)	14 (13)	16 (15)	22 (21)
Unsuccessful correction	20 (19)	58 (56)	18 (17)	61 (59)	52 (49)	74 (71)
p value	<0·0001		<0·0001		<0·0001	
Sensitivity analysis: Patients with first successful correction of fibrinogen level intraoperatively[b]						
≤15 minutes after trial drug start	62 (58)	20 (19)	61 (57)	17 (16)	31 (29)	6 (6)
>15 minutes or ≤90 minutes after trial drug start	14 (13)	11 (11)	15 (14)	14 (13)	8 (8)	2 (2)
>90 minutes after trial drug start	11 (10)	15 (14)	13 (12)	13 (13)	16 (15)	22 (21)
Unsuccessful correction	20 (19)	58 (56)	18 (17)	60 (58)	52 (49)	74 (71)
p value	<0·0001		<0·0001		<0·0001	

Data are n (%) unless otherwise specified. Differences presented are for FC – FFP/Cryo. CI=confidence interval. Cryo=cryoprecipitate. FC=human fibrinogen concentrate. FFP=fresh frozen plasma. FIBTEM A10=amplitude after 10 minutes as measured by ROTEM. FIBTEM MCF=maximum clot firmness as measured by ROTEM. IQR=interquartile range. n=number of patients in treatment group. ROTEM=rotational thromboelastometry. The sensitivity analysis considered a time window of ±3 minutes for the first category and of +3 minutes for the additional categories (≥12 and ≤18 minutes, >18 and ≤93 minutes and >93 minutes). [a]n=104 in the FC group and n=102 in the FFP/Cryo group for FIBTEM A10, n=103 in the FC group and n=102 in the FFP/Cryo group for FIBTEM MCF, n=106 in the FC group and n=104 in the FFP/Cryo group for Clauss assay. [b]n=107 in the FC group and n=104 in the FFP/Cryo group. [c]Determined by Clauss assay.

***Table S2*. Correction of functional fibrinogen level in patients by three different analysis methods (modified full analysis set)**

	FC (n=107)	FFP/Cryo (n=104)
Patients receiving any type of transfusion products after start of first trial drug administration until end of surgery[a]	79 (74)	72 (69)
Patients receiving		
Autologous blood transfusion/cell salvage	18 (17)	13 (13)
Volume, mL	0 (0–0)	0 (0–0)
Allogeneic platelet concentrates	1 (1)	1 (1)
Volume, mL	0 (0–0)	0 (0–0)
Allogeneic red blood cells	65 (61)	61 (59)
Volume. mL	329 (0–772)	328 (0–855)
Allogeneic FFP[b,c,d]	10 (9)	2 (2)
Volume, mL	0 (0–0)	0 (0–0)
Cryoprecipitate[b]	0	0
Volume, mL	0 (0–0)	0 (0–0)
Other	0	0
Volume, mL	0 (0–0)	0 (0–0)

Data are n (%) or median (IQR). Patients with no consumption of a particular transfusion product type are included with a result of zero for that type. Cryo=cryoprecipitate. FC=human fibrinogen concentrate. FFP=fresh frozen plasma. IQR=interquartile range. n=number of patients in treatment group. SD=standard deviation. [a]End of surgery is defined as time of last suture. [b]Only additional transfusion products excluding trial drug. [c]Two patients in the FFP group were incorrectly recorded as receiving allogeneic FFP as a transfusion product rather than as trial drug. [d]The longer administration time of FFP compared with FC means that the surgical team has more time to administer FFP in the FC group; thus, the higher intraoperative administration of FFP as coagulation therapy after trial drug is related to the longer period between the end of trial drug administration and the end of surgery.

***Table S*3. Consumption of transfusion products after start of first trial drug administration until end of surgery (modified full analysis set)**

	FC (n=107)	FFP/Cryo (n=104)	Difference (95% CI)	p value
Hospital stay period (days)				
1–7	10 (9)	12 (12)	-2 (-10 to 6)	
8–14	39 (36)	49 (47)	-11 (-24 to 3)	
15–21	34 (32)	17 (16)	15 (4 to 27)	0·065
22–28	13 (12)	12 (12)	1 (-8 to 9)	
29–36	4 (4)	10 (10)	-6 (-13 to 1)	
>36	7 (7)	4 (4)	3 (-3 to 9)	

Data are n (%) or median (IQR) for the modified analysis set or for the number of patients stated. Differences presented are for FC – FFP/Cryo; a negative value indicates shorter hospital stay after surgery in the FC group. Patients without a date of discharge were classified into the >36 days period. For the calculation of the median, only patients who had a date of discharge were included. CI=confidence interval. Cryo=cryoprecipitate. FC=human fibrinogen concentrate. FFP=fresh frozen plasma. n=number of patients.

***Table S4*. Length of hospital stay after surgery (modified full analysis set)**

	Total (n=222)		FC (n=110)		FFP/Cryo (n=112)		p value
	m	n (%)	m	n (%)	m	n (%)	
**Adverse event category**							
Any AEs	769	196 (88)	405	96 (87)	364	100 (89)	<0·001
Any non-serious AE	666	185 (83)	361	92 (84)	305	93 (83)	0·90
Any non-severe AE	719	193 (87)	379	94 (85)	340	99 (88)	0·52
Any adverse reaction	7	6 (3)	3	2 (2)	4	4 (4)	0·68
Any severe AE	50	35 (16)	26	17 (15)	24	18 (16)	0·90
Any severe adverse reaction	2	2 (1)	0	0	2	2 (2)	0·50
Any SAE	103	69 (31)	44	28 (25)	59	41 (37)	0·07
Any serious adverse reaction	5	5 (2)	1	1 (1)	4	4 (4)	0·37
Any AE leading to trial or treatment discontinuation	0	0	0	0	0	0	1·0
Any AE with outcome of death	1	1 (1)	0	0	1	1 (1)	1·0
Any thromboembolic event	18	17 (8)	4	4 (4)	14	13 (12)	0·041
Any suspicion of transmission of infective agents	0	0	0	0	0	0	1·0
Any relevant bleeding complication	41	30 (14)	20	16 (15)	21	14 (13)	0·67

Data are n (%) and number of events. Only treatment-emergent AEs are included. For each category, patients are included only once, even if they experienced multiple events in that category. AE=adverse event. Cryo=cryoprecipitate. FC=human fibrinogen concentrate. FFP=fresh frozen plasma. m=number of events. n=number of patients. SAE=serious adverse event.

***Table S5*. Summary of adverse events and other outcomes (safety set)**

	Total (n=222)		FC (n=110)		FFP/Cryo (n=112)	
	m	n (%)	m	n (%)	m	n (%)
**AEs by SOC/PT (PTs occurring ≥5% in Total)**						
Infections and infestations	83	66 (29·7)	41	32 (29·1)	42	34 (30·4)
Pneumonia	21	21 (9·5)	10	10 (9·1)	11	11 (9·8)
Urinary tract infection	21	21 (9·5)	10	10 (9·1)	11	11 (9·8)
Psychiatric disorders	68	62 (27·9)	36	32 (29·1)	32	30 (26·8)
Hallucination	56	55 (24·8)	30	29 (26·4)	26	26 (23·2)
Injury, poisoning and procedural complications	78	56 (25·2)	48	32 (29·1)	30	24 (21·4)
Anaemia postoperative	24	23 (10·4)	15	15 (13·6)	9	8 (7·1)
Vascular disorders	66	49 (22·1)	40	31 (28·2)	26	18 (16·1)
Hypotension	43	33 (14·9)	29	23 (20·9)	14	10 (8·9)
Cardiac disorders	53	45 (20·3)	29	23 (20·9)	24	22 (19·6)
Tachycardia	36	33 (14·9)	22	19 (17·3)	14	14 (12·5)
Gastrointestinal disorders	62	44 (19·8)	38	23 (20·9)	24	21 (18·8)
Nausea	16	14 (6·3)	12	10 (9·1)	4	4 (3·6)
Constipation	12	12 (5·4)	8	8 (7·3)	4	4 (3·6)
Respiratory, thoracic and mediastinal disorders	63	44 (19·8)	28	17 (15·5)	35	27 (24·1)
Pneumothorax	12	11 (5·0)	6	5 (4·5)	6	6 (5·4)
Pulmonary embolism	11	11 (5·0)	3	3 (2·7)	8	8 (7·1)
Blood and lymphatic system disorders	51	41 (18·5)	18	18 (16·4)	33	23 (20·5)
Anaemia	33	26 (11·7)	12	12 (10·9)	21	14 (12·5)
Investigations	58	41 (18·5)	28	22 (20·0)	30	19 (17·0)
Gamma-glutamyltransferase increased	13	13 (5·9)	5	5 (4·5)	8	8 (7·1)
Hepatobiliary disorders	27	25 (11·3)	14	13 (11·8)	13	12 (10·7)
Hypertransaminasaemia	24	24 (10·8)	13	13 (11·8)	11	11 (9·8)
Metabolism and nutrition disorders	33	23 (10·4)	16	13 (11·8)	17	10 (8·9)
Hypokalaemia	12	11 (5·0)	5	5 (4·5)	7	6 (5·4)

Data are n (%) and number of events. Only treatment-emergent AEs are included. AEs were coded using MedDRA version 26.1 and presented by SOC and PT. For each SOC and PT, patients are included only once, even if they experienced multiple events in that SOC or PT. AE=adverse event. Cryo=cryoprecipitate. FC=human fibrinogen concentrate. FFP=fresh frozen plasma. n=number of patients. m=number of events MedDRA=Medical Dictionary for Regulatory Activities. PT=preferred term. SOC=system organ class.

***Table S6*. Summary of adverse events with a preferred term frequency of ≥5% in the overall trial population (safety set)**
